# Supplementary material for: Design and application of α-ketothioesters as 1,2-dicarbonyl-forming reagents
Source: Nat Commun. 2019 Jun 17;10:2661. doi: 10.1038/s41467-019-10651-w (PMC6572800; doi:10.1038/s41467-019-10651-w)
Supplement: Supplementary file 1 — Supplementary Information [file 41467_2019_10651_MOESM1_ESM.pdf]

Supporting Information

Design and Application of α-Ketothioesters as 1,2-Dicarbonyl-forming Reagents

Wang et al.

Supplementary Methods

General Information.

^1H and ^{13}C NMR spectra were recorded on 400 MHz NMR spectrometers (Bruker AVANCE) using $CDCl_3$. Chemical shifts are reported in parts per million (ppm). Chemical shifts for protons are reported in parts per million relative to chloroform, dichloromethane or DMSO ($CHCl_3$ δ 7.26, $(CH_3)_2CO$ δ 2.05, DMSO δ 2.50). Chemical shifts for carbon are reported in parts per million relative to chloroform, dichloromethane or DMSO ($CHCl_3$ δ 77.0, $(CH_3)_2CO$ δ 29.84, DMSO δ 39.52). Data are represented as follows: chemical shift, multiplicity (br = broad, s = singlet, d = doublet, t = triplet, q = quartet, m = multiplet), coupling constants in Hertz (Hz), integration. Mass spectra were recorded on a Shimadzu GCMS-QP2010 Ultra. IR spectra were recorded on TENSOR (27) Series FT-IR 241.Spectrometers. α-hydroxy ketones **1** were prepared adopting reported procedures.[1-2]

General procedure for the synthesis of 1,2-dicarbonyl-forming reagents

Under a N_2 atmosphere, α-hydroxy ketones **1** (0.5 mmol), S_8 (64.2 mg, 2 mmol), $KHCO_3$ (100 mg, 1 mmol), TBAB (32.3 mg, 0.1 mmol), H_2O (180 mg, 4 mmol) and CPME (4 mL) were added to a Schlenk tube. After stirring for 10 hours at 90 ^oC (detect by TLC), RBr (0.75 mmol, 1.5 equiv) was added to this mixture. The resulting mixture was allowed to stir for 2 hours at 90 ^oC. After completion of the reaction, water (5 mL) was added. The solution was extracted with ethyl acetate and organic layers were combined, dried over sodium sulfate. After evaporation of solvent, the residue was purified by column chromatography to give the corresponding product.

General procedure for the dicarbonylation of amines

α-Ketothioester **2** (0.2 mmol), amine (0.2 mmol) and THF (2 mL) were added to a reaction tube. After stirring for 12 hours at room temperature (detect by TLC), the solvent was removed and the residue was purified by column chromatography to give the corresponding product **3**.

General procedure for the dicarbonylation of aryl borates

Under a N_2 atmosphere, α-ketothioester **2** (0.1 mmol), aryl borate 4 (0.1 mmol), $Pd_2(dba)_3$ (0.0025mmol, 2.5 mol%), 4,4'-dimethoxy-2,2'-bipyridine (0.01 mmol, 10 mol%), CuTc (0.1 mmol, 1 equiv), K_2CO_3 (0.15 mmol), anhydrous Na_2SO_4 (0.15 mmol) and DMF (1 mL) were added to a Schlenk tube. After stirring for 12 hours at 45 ^oC (detect by TLC), the mixture was cooled to room temperature and water (5 mL) was added. Then the mixture was extracted with ethyl acetate and organic layers were combined, dried over sodium sulfate before the organic phase was concentrated under vacuum. The residue was purified by column chromatography to give the corresponding product.

Optimization of the 1,2-dicarbonyl-forming reagent

Supplementary Table 1. The optimization of reaction conditions

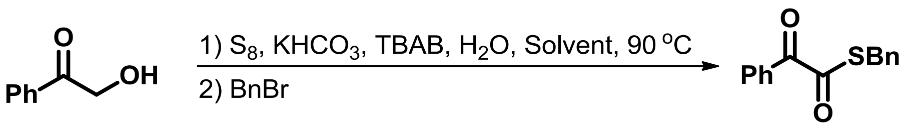

1a 2a

Entry	Solvent	Yield(%)
1	DMSO	25
2	CH_3CN	ND
3	DMAc	62
4	DMF	71
5	1,4-Dioxane	72
6	Toluene	60
7	CPME	86

Characterization of the products

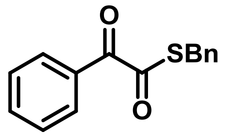

2a

***S*-Benzyl 2-oxo-2-phenylethanethioate (2a)**: Prepared following general procedure using 2-hydroxy-1-phenylethan-1-one **1a** (68.1 mg, 0.5 mmol), S_8 (64.2 mg, 2 mmol), $KHCO_3$ (100 mg, 1 mmol), TBAB (32.3 mg, 0.1 mmol), H_2O (180 mg, 4 mmol), CPME (4 mL) and BnBr (0.75 mmol, 1.5 equiv), the reaction was stirred at 90 ^oC for 12 hours giving **2a** (110.2 mg) in 86% yield as a yellow solid by column chromatography. **^{1}H NMR** (400 MHz, $CDCl_3$) δ 8.14 (d, *J* = 8.2 Hz, 2H), 7.65 (t, *J* = 7.4 Hz, 1H), 7.49 (t, *J* = 7.8 Hz, 2H), 7.40 – 7.27 (m, 5H), 4.28 (s, 2H). **^{13}C NMR** (100 MHz, $CDCl_3$) δ 191.9, 185.7, 136.3, 134.8, 131.5, 130.6, 128.9, 128.7, 128.6, 127.5, 33.1. **HRMS** (EI) Calcd for $C_{15}H_{12}O_2S$ 256.0558, Found 256.0555.

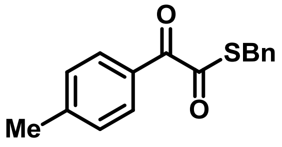

2b

***S*-Benzyl 2-oxo-2-(p-tolyl)ethanethioate** (**2b**): Prepared following general procedure using 2-hydroxy-1-(*p*-tolyl)ethan-1-one **1b** (75.1 mg, 0.5 mmol), S_8 (64.2 mg, 2 mmol), $KHCO_3$ (100 mg, 1 mmol), TBAB (32.3 mg, 0.1 mmol), H_2O (180 mg, 4 mmol), CPME (4 mL) and BnBr (0.75 mmol, 1.5 equiv), the reaction was stirred at 90 ^oC for 12 hours giving **2b** (96.6 mg) in 72% yield as a yellow solid by column chromatography. **^{1}H NMR** (400 MHz, $CDCl_3$) δ 8.06 (d, *J* = 8.3 Hz, 2H), 7.41 – 7.27 (m, 7H), 4.28 (s, 2H), 2.44 (s, 3H). **^{13}C NMR** (100 MHz, $CDCl_3$) δ 192.2, 185.4, 146.2, 136.4, 130.8, 129.5, 129.0, 128.9, 128.6, 127.5, 33.2, 21.8. **HRMS** (EI) Calcd for $C_{16}H_{14}O_2S$ 270.0715, Found 270.0710.

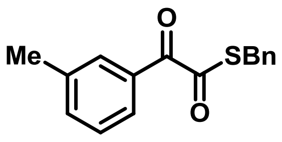

2c

***S*-Benzyl 2-oxo-2-(m-tolyl)ethanethioate** (**2c**): Prepared following general procedure using 2-hydroxy-1-(*m*-tolyl)ethan-1-one **1c** (75.1 mg, 0.5 mmol), S_8 (64.2 mg, 2 mmol), $KHCO_3$ (100 mg, 1 mmol), TBAB (32.3 mg, 0.1 mmol), H_2O (180 mg, 4 mmol), CPME (4 mL) and BnBr (0.75 mmol, 1.5 equiv), the reaction was stirred at 90 ^oC for 12 hours giving **2c** (109.8 mg) in 82% yield as a yellow oil by column chromatography. **^{1}H NMR** (400 MHz, $CDCl_3$) δ 7.94 (d, J = 8.9 Hz, 2H), 7.47 (d, J = 7.6 Hz, 1H), 7.42 – 7.27 (m, 6H), 4.29 (s, 2H), 2.42 (s, 3H). **^{13}C NMR** (100 MHz, $CDCl_3$) δ 192.2, 186.1, 138.7, 136.4, 135.7, 131.5, 131.0, 128.9, 128.7, 128.6, 128.0, 127.5, 33.2, 21.2. **HRMS** (EI) Calcd for $C_{16}H_{14}O_2S$ 270.0715, Found 270.0717.

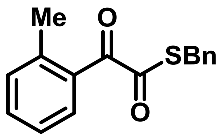

2d

***S*-Benzyl 2-oxo-2-(o-tolyl)ethanethioate** (**2d**): Prepared following general procedure using 2-hydroxy-1-(*o*-tolyl)ethan-1-one **1d** (75.1 mg, 0.5 mmol), S_8 (64.2 mg, 2 mmol), $KHCO_3$ (100 mg, 1 mmol), TBAB (32.3 mg, 0.1 mmol), H_2O (180 mg, 4 mmol), CPME (4 mL) and BnBr (0.75 mmol, 1.5 equiv), the reaction was stirred at 90 ^oC for 12 hours giving **2d** (85.2 mg) in 63% yield as a yellow oil by column chromatography. **^{1}H NMR** (400 MHz, $CDCl_3$) δ 7.76 (d, J = 8.0 Hz, 1H), 7.51 – 7.45 (m, 1H), 7.40 – 7.28 (m, 7H), 4.28 (s, 2H), 2.53 (s, 3H). **^{13}C NMR** (100 MHz, $CDCl_3$) δ 192.5, 188.8, 141.0, 136.4, 133.4, 132.1, 132.0, 130.9, 129.0, 128.7, 127.6, 125.6, 33.3, 21.2. **HRMS** (EI) Calcd for $C_{16}H_{14}O_2S$ 270.0715, Found 270.0718.

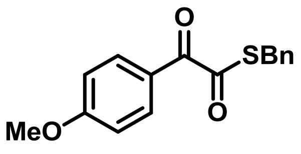

2e

***S*-Benzyl 2-(4-methoxyphenyl)-2-oxoethanethioate (2e)**: Prepared following general procedure using 2-hydroxy-1-(4-methoxyphenyl)ethan-1-one **1e** (83.1 mg, 0.5 mmol), S_8 (64.2 mg, 2 mmol), $KHCO_3$ (100 mg, 1 mmol), TBAB (32.3 mg, 0.1 mmol), H_2O (180 mg, 4 mmol), CPME (4 mL) and BnBr (0.75 mmol, 1.5 equiv), the reaction was stirred at 90 ^oC for 12 hours giving **2e** (86.7 mg) in 61% yield as a yellow oil by column chromatography. **^{1}H NMR** (400 MHz, $CDCl_3$) δ 8.18 (d, *J* = 8.9 Hz, 2H), 7.41 – 7.26 (m, 5H), 6.97 (d, *J* = 8.9 Hz, 2H), 4.26 (s, 2H), 3.88 (s, 3H). **^{13}C NMR** (100 MHz, $CDCl_3$) δ 192.5, 184.0, 165.0, 136.5, 133.3, 128.9, 128.6, 127.4, 124.3, 114.2, 55.5, 33.1. **HRMS** (EI) Calcd for $C_{16}H_{14}O_3S$ 286.0664, Found 286.0667.

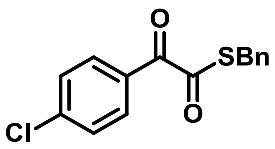

2f

***S*-Benzyl 2-(4-chlorophenyl)-2-oxoethanethioate (2f)**: Prepared following general procedure using 1-(4-chlorophenyl)-2-hydroxyethan-1-one **1f** (42.6 mg, 0.25 mmol), S_8 (24.0 mg, 0.75 mmol), $KHCO_3$ (50 mg, 0.5 mmol), TBAB (16.1 mg, 0.05 mmol), H_2O (180 mg, 4 mmol), CPME (2 mL) and BnBr (0.375 mmol, 1.5 equiv), the reaction was stirred at 90 ^oC for 12 hours giving **2f** (53.6 mg) in 74% yield as a yellow solid by column chromatography. **^{1}H NMR** (400 MHz, $CDCl_3$) δ 8.11 – 8.05 (m, 2H), 7.48 – 7.42 (m, 2H), 7.36 – 7.22 (m, 5H), 4.24 (s, 2H). **^{13}C NMR** (100 MHz, $CDCl_3$) δ 191.7, 184.4, 141.7, 136.3, 132.1, 130.0, 129.2, 128.9, 128.7, 127.6, 33.3. **HRMS** (EI) Calcd for $C_{15}H_{11}O_2SCl$ 290.0168, Found 290.0172.

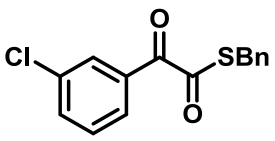

2g

***S*-Benzyl 2-(3-chlorophenyl)-2-oxoethanethioate (2g)**: Prepared following general procedure using 1-(3-chlorophenyl)-2-hydroxyethan-1-one **1g** (42.6 mg, 0.25 mmol), S_8 (24.0 mg, 0.75 mmol), $KHCO_3$ (50 mg, 0.5 mmol), TBAB (16.1 mg, 0.05 mmol), H_2O (180 mg, 4

mmol), CPME (2 mL) and BnBr (0.375 mmol, 1.5 equiv), the reaction was stirred at 90 ^{o}C for 12 hours giving **2g** (55.4 mg) in 76% yield as a yellow solid by column chromatography. **^{1}H NMR** (400 MHz, $CDCl_3$) δ 8.10 (t, *J* = 1.8 Hz, 1H), 8.03 – 7.98 (m, 1H), 7.59 (ddd, *J* = 8.0, 2.1, 1.0 Hz, 1H), 7.41 (t, *J* = 7.9 Hz, 1H), 7.36 – 7.24 (m, 5H), 4.24 (s, 2H). **^{13}C NMR** (100 MHz, $CDCl_3$) δ 191.5, 184.4, 136.2, 135.1, 134.7, 133.2, 130.5, 130.1, 128.9, 128.9, 128.7, 127.7, 33.4. **HRMS** (EI) Calcd for C15$H_{11}O_2$SCl 290.0168, Found 290.0166.

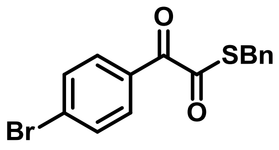

2h

***S*-Benzyl 2-(4-bromophenyl)-2-oxoethanethioate (2h)**: Prepared following general procedure using 1-(4-bromophenyl)-2-hydroxyethan-1-one **1h** (53.8 mg, 0.25 mmol), S_8 (24.0 mg, 0.75 mmol), $KHCO_3$ (50 mg, 0.5 mmol), TBAB (16.1 mg, 0.05 mmol), H_2O (180 mg, 4 mmol), CPME (2 mL) and BnBr (0.375 mmol, 1.5 equiv), the reaction was stirred at 90 ^{o}C for 12 hours giving **2h** (46.1 mg) in 55% yield as a yellow oil by column chromatography. **^{1}H NMR** (400 MHz, $CDCl_3$) δ 8.04 – 7.97 (m, 2H), 7.66 – 7.59 (m, 2H), 7.38 – 7.23 (m, 5H), 4.24 (s, 2H). **^{13}C NMR** (100 MHz, $CDCl_3$) δ 191.7, 184.6, 136.2, 132.2, 132.2, 130.7, 130.3, 128.9, 128.7, 127.6, 33.3. **HRMS** (EI) Calcd for $C_{15}H_{11}O_2SBr$ 333.9663, Found 333.9665.

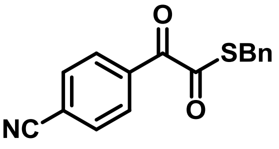

2i

***S*-benzyl 2-(4-cyanophenyl)-2-oxoethanethioate (2i)**: Prepared following general procedure using 1-(4-cyanophenyl)-2-hydroxyethan-1-one **1i** (40.3 mg, 0.25 mmol), S_8 (24.0 mg, 0.75 mmol), $KHCO_3$ (50 mg, 0.5 mmol), TBAB (16.1 mg, 0.05 mmol), H_2O (180 mg, 4 mmol), CPME (2 mL) and BnBr (0.375 mmol, 1.5 equiv), the reaction was stirred at 90 ^{o}C for 12 hours giving **2i** (38.8 mg) in 55% yield as a yellow solid by column chromatography. **^{1}H NMR** (400 MHz, $CDCl_3$) δ 8.28 – 8.22 (m, 2H), 7.83 – 7.76

(m, 2H), 7.39 – 7.27 (m, 5H), 4.27 (s, 2H). **^{13}C NMR** (100 MHz, $CDCl_3$) δ 191.0, 184.2, 136.0, 134.8, 132.4, 131.1, 129.0, 128.8, 127.8, 117.8, 117.6, 33.4. **HRMS** (EI) Calcd for $C_{16}H_{11}NO_2S$ 281.0511, Found 281.0515.

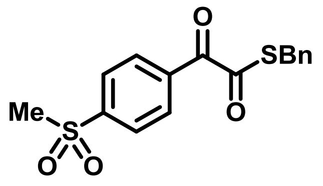

2j

***S*-Benzyl 2-(4-(methylsulfonyl)phenyl)-2-oxoethanethioate (2j)**: Prepared following general procedure using 2-hydroxy-1-(4-(methylsulfonyl)phenyl)ethan-1-one **1j** (53.6 mg, 0.25 mmol), S_8 (24.0 mg, 0.75 mmol), $KHCO_3$ (50 mg, 0.5 mmol), TBAB (16.1 mg, 0.05 mmol), H_2O (180 mg, 4 mmol), CPME (2 mL) and BnBr (0.375 mmol, 1.5 equiv), the reaction was stirred at 90 ^oC for 12 hours giving **2j** (62.4 mg) in 75% yield as a yellow solid by column chromatography. **^{1}H NMR** (400 MHz, $CDCl_3$) δ 8.30 (d, *J* = 8.5 Hz, 2H), 8.06 (d, *J* = 8.5 Hz, 2H), 7.39 – 7.24 (m, 5H), 4.27 (s, 2H), 3.07 (s, 3H). **^{13}C NMR** (100 MHz, $CDCl_3$) δ 191.0, 184.3, 145.4, 135.9, 135.8, 131.5, 128.9, 128.7, 127.7, 127.7, 44.1, 33.4. **HRMS** (EI) Calcd for $C_{16}H_{14}O_4S_2$ 334.0334, Found 334.0336.

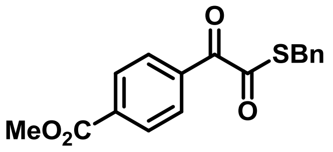

2k

Methyl 4-(2-(benzylthio)-2-oxoacetyl)benzoate (2k): Prepared following general procedure using methyl 4-(2-hydroxyacetyl)benzoate **1k** (48.5 mg, 0.25 mmol), S_8 (24.0 mg, 0.75 mmol), $KHCO_3$ (50 mg, 0.5 mmol), TBAB (16.1 mg, 0.05 mmol), H_2O (180 mg, 4 mmol), CPME (2 mL) and BnBr (0.375 mmol, 1.5 equiv), the reaction was stirred at 90 ^oC for 12 hours giving **2k** (50.2 mg) in 64% yield as a yellow solid by column chromatography. **^{1}H NMR** (400 MHz, $CDCl_3$) δ 8.21 – 8.17 (m, 2H), 8.16 – 8.12 (m, 2H), 7.39 – 7.27 (m, 5H), 4.28 (s, 2H), 3.96 (s, 3H). **^{13}C NMR** (100 MHz, $CDCl_3$) δ 191.5, 185.2, 165.9, 136.2, 135.2, 134.9, 130.7, 129.8, 129.0, 128.8, 127.7, 52.6, 33.4. **HRMS** (EI) Calcd for $C_{17}H_{14}O_4S$

314.0613, Found 314.0614.

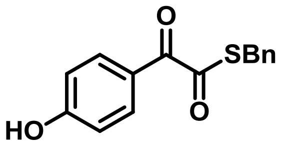

2l

***S*-Benzyl 2-(4-hydroxyphenyl)-2-oxoethane thioate (2l)**: Prepared following general procedure using 2-hydroxy-1-(4-hydroxyphenyl)ethan-1-one **1l** (38.0 mg, 0.25 mmol), S_8 (24.0 mg, 0.75 mmol), $KHCO_3$ (50 mg, 0.5 mmol), TBAB (16.1 mg, 0.05 mmol), H_2O (180 mg, 4 mmol), CPME (2 mL) and BnBr (0.375 mmol, 1.5 equiv), the reaction was stirred at 90 ^oC for 12 hours giving **2l** (30.8 mg) in 45% yield as a yellow solid by column chromatography. **^{1}H NMR** (400 MHz, $CDCl_3$) δ 8.14 – 8.07 (m, 2H), 7.38 – 7.25 (m, 5H), 6.94 – 6.87 (m, 2H), 6.15 (s, 1H), 4.26 (s, 2H). **^{13}C NMR** (100 MHz, $CDCl_3$) δ 192.7, 184.3, 161.9, 136.5, 133.8, 129.0, 128.7, 127.6, 124.5, 115.9, 33.3. **HRMS** (EI) Calcd for $C_{15}H_{12}O_3S$ 272.0507, Found 272.0509.

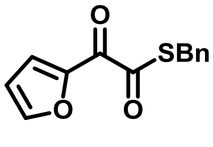

2m

***S*-Benzyl 2-(furan-2-yl)-2-oxoethanethioate (2m)**: Prepared following general procedure using 1-(furan-2-yl)-2-hydroxyethan-1-one **1m** (31.5 mg, 0.25 mmol), S_8 (24.0 mg, 0.75 mmol), $KHCO_3$ (50 mg, 0.5 mmol), TBAB (16.1 mg, 0.05 mmol), H_2O (180 mg, 4 mmol), CPME (2 mL) and BnBr (0.375 mmol, 1.5 equiv), the reaction was stirred at 90 ^oC for 12 hours giving **2m** (39.8 mg) in 65% yield as a yellow oil by column chromatography. **^{1}H NMR** (400 MHz, $CDCl_3$) δ 7.80 (dd, *J* = 3.7, 0.6 Hz, 1H), 7.75 (dd, *J* = 1.6, 0.6 Hz, 1H), 7.32 – 7.18 (m, 5H), 6.58 (dd, *J* = 3.7, 1.7 Hz, 1H), 4.15 (s, 2H). **^{13}C NMR** (100 MHz, $CDCl_3$) δ 190.7, 171.5, 150.1, 147.7, 136.3, 128.9, 128.7, 127.6, 126.1, 113.3, 33.2. **HRMS** (EI) Calcd for $C_{13}H_{10}O_3S$ 246.0351, Found 246.0350.

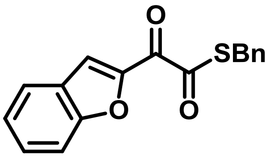

2n

***S*-Benzyl 2-(benzofuran-2-yl)-2-oxoethanethioate (2n)**: Prepared following general procedure using 1-(benzofuran-2-yl)-2-hydroxyethan-1-one **1n** (44.0 mg, 0.25 mmol), S_8 (24.0 mg, 0.75 mmol), $KHCO_3$ (50 mg, 0.5 mmol), TBAB (16.1 mg, 0.05 mmol), H_2O (180 mg, 4 mmol), CPME (2 mL) and BnBr (0.375 mmol, 1.5 equiv), the reaction was stirred at 90 ^oC for 12 hours giving **2n** (48.6 mg) in 66% yield as a yellow solid by column chromatography. **^{1}H NMR** (400 MHz, $CDCl_3$) δ 8.16 (d, *J* = 0.7 Hz, 1H), 7.72 (d, *J* = 7.9 Hz, 1H), 7.56 (d, *J* = 8.4 Hz, 1H), 7.52 – 7.46 (m, 1H), 7.34 – 7.20 (m, 6H), 4.20 (s, 2H). **^{13}C NMR** (100 MHz, $CDCl_3$) δ 190.4, 173.7, 156.7, 147.5, 136.2, 130.1, 129.0, 128.7, 127.6, 127.0, 124.4, 124.2, 122.1, 112.6, 33.3. **HRMS** (EI) Calcd for $C_{17}H_{12}O_3S$ 296.0507, Found 296.0509.

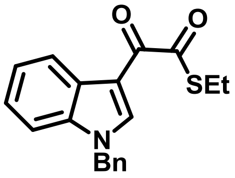

2o

***S*-Ethyl 2-(1-benzyl-1H-indol-3-yl)-2-oxoethanethioate (2o)**: Prepared following general procedure using 1-(1-benzyl-1H-indol-3-yl)-2-hydroxyethan-1-one **1o** (33.0 mg, 0.125 mmol), S_8 (23.0 mg, 0.72 mmol, 5.8 equiv), Cs_2CO_3 (195 mg, 0.6 mmol, 4.8 equiv), TBAB (8.0 mg, 0.025 mmol), DMF (1 mL) and EtBr (28.0 mg, 0.25 mmol, 2 equiv), the reaction was stirred at 90 ^oC for 12 hours giving **2o** (24.9 mg) in 62% yield as a yellow solid by column chromatography. **^{1}H NMR** (400 MHz, $CDCl_3$) δ 8.56 (s, 1H), 8.46 (d, *J* = 7.8 Hz, 1H), 7.39 – 7.27 (m, 6H), 7.21 – 7.15 (m, 2H), 5.37 (s, 2H), 2.97 (d, *J* = 7.4 Hz, 2H), 1.34 (t, *J* = 7.4 Hz, 3H). **^{13}C NMR** (100 MHz, $CDCl_3$) δ 194.6, 178.2, 139.9, 136.8, 135.2, 129.1, 128.3, 127.7, 127.0, 124.3, 123.6, 122.9, 110.6(1), 110.5(9), 51.2, 23.1, 14.2. **HRMS** (EI) Calcd for $C_{19}H_{17}NO_2S$ 323.0980, Found 323.0983.

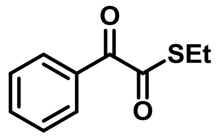

2p

***S*-Ethyl 2-oxo-2-phenylethanethioate (2p)**: Prepared following general procedure using 2-hydroxy-1-phenylethan-1-one **1a** (408.5 mg, 3 mmol), S_8 (12.0 mmol，4.0 equiv), $KHCO_3$ (6.0 mmol，2.0 equiv), TBAB (0.6 mmol，20 mol%), H_2O (180 mg，60 mmol，20 equiv), CPME (20 mL) and EtBr (6.0 mmol, 2equiv), the reaction was stirred at 90 ^{o}C for 12 h giving **2p** (468.3 mg) in 81% yield as a yellow oil by column chromatography. **^{1}H NMR** (400 MHz, $CDCl_3$) δ 8.16 – 8.08 (m, 2H), 7.68 – 7.62 (m, 1H), 7.54 – 7.47 (m, 2H), 3.06 (q, *J* = 7.4 Hz, 2H), 1.37 (t, *J* = 7.4 Hz, 3H). **^{13}C NMR** (100 MHz, $CDCl_3$) δ 193.0, 186.4, 134.8, 131.6, 130.7, 128.8, 23.3, 14.2. **HRMS** (EI) Calcd for $C_{10}H_{10}O_2S$ 194.0402, Found 194.0405.

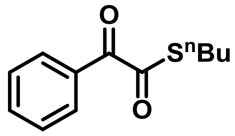

2q

***S*-Butyl 2-oxo-2-phenylethanethioate (2q)**: Prepared following general procedure using 2-hydroxy-1-phenylethan-1-one **1a** (68.1 mg, 0.5 mmol), S_8 (64.2 mg, 2 mmol), $KHCO_3$ (100 mg, 1 mmol), TBAB (32.3 mg, 0.1 mmol), H_2O (180 mg, 4 mmol), CPME (4 mL) and ^{n}BuBr (1.0 mmol, 2 equiv), the reaction was stirred at 90 ^{o}C for 12 hours giving **2q** (92.3 mg) in 83% yield as a yellow oil by column chromatography. **^{1}H NMR** (400 MHz, $CDCl_3$) δ 8.15 – 8.06 (m, 2H), 7.67 – 7.60 (m, 1H), 7.52 – 7.46 (m, 2H), 3.05 (t, *J* = 7.4 Hz, 2H), 1.70 – 1.63 (m, 2H), 1.51 – 1.40 (m, 2H), 0.95 (t, *J* = 7.3 Hz, 3H). **^{13}C NMR** (100 MHz, $CDCl_3$) δ 193.0, 186.4, 134.7, 131.6, 130.7, 128.8, 31.1, 28.5, 22.0, 13.5. **HRMS** (EI) Calcd for $C_{12}H_{14}O_2S$ 222.0715, Found 222.0713.

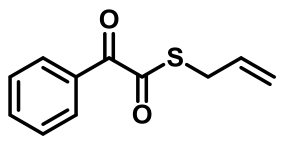

2r

***S*-Allyl 2-oxo-2-phenylethanethioate (2r)**: Prepared following general procedure using 2-hydroxy-1-phenylethan-1-one **1a** (68.1 mg, 0.5 mmol), S_8 (64.2 mg, 2 mmol), $KHCO_3$ (100 mg, 1 mmol), TBAB (32.3 mg, 0.1 mmol), H_2O (180 mg, 4 mmol), CPME (4 mL) and allyl bromide (0.75 mmol, 1.5 equiv), the reaction was stirred at 90 ^oC for 12 hours giving **2r** (57.5 mg) in 56% yield as a yellow oil by column chromatography. **^{1}H NMR** (400 MHz, $CDCl_3$) δ 8.15 – 8.06 (m, 2H), 7.68 – 7.59 (m, 1H), 7.48 (dd, *J* = 11.0, 4.7 Hz, 2H), 5.86 (ddt, *J* = 16.9, 10.0, 6.9 Hz, 1H), 5.38 – 5.30 (m, 1H), 5.18 (dd, *J* = 10.0, 0.9 Hz, 1H), 3.68 (dd, *J* = 7.0, 0.9 Hz, 2H). **^{13}C NMR** (100 MHz, $CDCl_3$) δ 192.0, 185.9, 134.8, 131.9, 131.5, 130.7, 128.7, 118.8, 31.6. **HRMS** (EI) Calcd for $C_{11}H_{10}O_2S$ 206.0402, Found 206.0399.

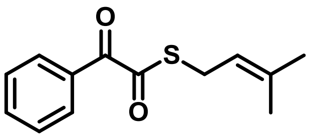

2s

S-(3-Methylbut-2-en-1-yl) 2-oxo-2-phenylethanethioate (2s): Prepared following general procedure using 2-hydroxy-1-phenylethan-1-one **1a** (34.1 mg, 0.25 mmol), S_8 (24.0 mg, 0.75 mmol), $KHCO_3$ (50 mg, 0.5 mmol), TBAC (13.9 mg, 0.05 mmol), DMF (2 mL) and 1-bromo-3-methylbut-2-ene (0.375 mmol, 1.5 equiv), the reaction was stirred at 90 ^oC for 12 hours giving **2s** (38.5 mg) in 66% yield as a yellow oil by column chromatography. **^{1}H NMR** (400 MHz, $CDCl_3$) δ 8.15 – 8.09 (m, 2H), 7.67 – 7.62 (m, 1H), 7.53 – 7.46 (m, 2H), 5.33 – 5.25 (m, 1H), 3.69 (d, *J* = 7.9 Hz, 2H), 1.74 (s, 6H). **^{13}C NMR** (100 MHz, $CDCl_3$) δ 193.0, 186.4, 137.9, 134.7, 131.7, 130.7, 128.8, 117.6, 27.2, 25.6, 17.9. **HRMS** (EI) Calcd for $C_{13}H_{14}O_2S$ 234.0715, Found 234.0719.

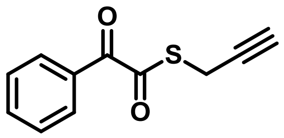

2t

***S*-Prop-2-yn-1-yl 2-oxo-2-phenylethanethioate** (**2t**): Prepared following general procedure using 2-hydroxy-1-phenylethan-1-one **1a** (34.1 mg, 0.25 mmol), S_8 (24.0 mg, 0.75 mmol), $KHCO_3$ (50 mg, 0.5 mmol), TBAB (16.1 mg, 0.05 mmol), DMF (2 mL) and 3-bromopropyne (0.375 mmol, 1.5 equiv), the reaction was stirred at 90 ^{o}C for 12 hours giving **2t** (36.1 mg) in 71% yield as a yellow oil by column chromatography. **^{1}H NMR** (400 MHz, $CDCl_3$) δ 8.21 – 8.14 (m, 2H), 7.70 – 7.62 (m, 1H), 7.54 – 7.46 (m, 2H), 3.76 (d, J = 2.7 Hz, 2H), 2.23 (t, J = 2.7 Hz, 1H). **^{13}C NMR** (100 MHz, $CDCl_3$) δ 190.9, 184.7, 135.1, 131.4, 130.9, 128.9, 77.9, 71.5, 17.4. **HRMS** (EI) Calcd for $C_{11}H_8O_2S$ 204.0245, Found 204.0246.

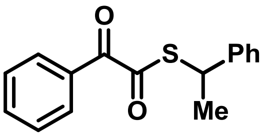

2u

***S*-(1-Phenylethyl) 2-oxo-2-phenylethanethioate** (**2u**): Prepared following general procedure using 2-hydroxy-1-phenylethan-1-one **1a** (34.1 mg, 0.25 mmol), S_8 (24.0 mg, 0.75 mmol), $KHCO_3$ (50 mg, 0.5 mmol), TBAC (13.9 mg, 0.05 mmol), DMF (2 mL) and (1-bromoethyl)benzene (0.375 mmol, 1.5 equiv), the reaction was stirred at 90 ^{o}C for 12 hours giving **2u** (46.2 mg) in 68% yield as a yellow oil by column chromatography. **^{1}H NMR** (400 MHz, $CDCl_3$) δ 8.12 – 8.05 (m, 2H), 7.65 – 7.59 (m, 1H), 7.50 – 7.43 (m, 2H), 7.43 – 7.38 (m, 2H), 7.36 – 7.30 (m, 2H), 7.30 – 7.23 (m, 1H), 4.87 (q, J = 7.2 Hz, 1H), 1.76 (d, J = 7.2 Hz, 3H). **^{13}C NMR** (100 MHz, $CDCl_3$) δ 191.8, 186.1, 141.8, 134.7, 131.7, 130.7, 128.7, 128.6, 127.6, 127.3, 43.2, 22.1. **HRMS** (EI) Calcd for $C_{16}H_{14}O_2S$ 270.0715, Found 270.0718.

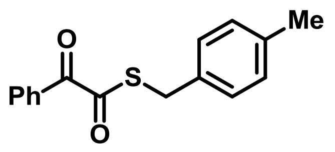

2v

***S*-4-Methylbenzyl 2-oxo-2-phenylethanethioate** (**2v**): Prepared following general procedure using 2-hydroxy-1-phenylethan-1-one **1a** (0.5 mmol, 68.1 mg), S_8 (2.0 mmol, 4.0 equiv), $KHCO_3$ (1.0 mmol, 2.0 equiv)，TBAB (32.3 mg, 0.1 mmol), H_2O (4 mmol, 20 equiv), CPME (4 mL) and 1-(bromomethyl)-4-methylbenzene (0.75 mmol, 1.5 equiv), the reaction was stirred at 90 ^{o}C for 12 hours giving **2v** (112.5 mg) in 84% yield as a yellow solid by column chromatography. **^{1}H NMR** (400 MHz, $CDCl_3$) δ 8.18 – 8.13 (m, 2H), 7.69 – 7.63 (m, 1H), 7.54 – 7.48 (m, 2H), 7.28 (d, *J* = 8.0 Hz, 2H), 7.15 (d, *J* = 7.9 Hz, 2H), 4.27 (s, 2H), 2.35 (s, 3H). **^{13}C NMR** (100 MHz, $CDCl_3$) δ 192.1, 185.9, 137.3, 134.8, 133.2, 131.6, 130.7, 129.4, 128.8, 128.7, 33.0, 21.0. **HRMS** (EI) Calcd for $C_{16}H_{14}O_2S$ 270.0715, Found 270.0717.

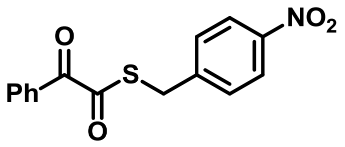

2w

***S*-4-Nitrobenzyl 2-oxo-2-phenylethanethioate (2w)**: Prepared following general procedure using 2-hydroxy-1-phenylethan-1-one **1a** (0.5 mmol, 68.1 mg), S_8 (2.0 mmol, 4.0 equiv), $KHCO_3$ (1.0 mmol, 2.0 equiv)，TBAB (32.3 mg, 0.1 mmol), H_2O (4 mmol, 20 equiv), CPME (4 mL) and 1-(bromomethyl)-4-nitrobenzene (1.25 mmol, 2.5 equiv), the reaction was stirred at 90 ^{o}C for 12 hours giving **2w** (81.9 mg) in 55% yield as a yellow solid by column chromatography. **^{1}H NMR** (400 MHz, $CDCl_3$) δ 8.19 – 8.09 (m, 4H), 7.69 – 7.62 (m, 1H), 7.57 – 7.46 (m, 4H), 4.30 (s, 2H). **^{13}C NMR** (100 MHz, $CDCl_3$) δ 191.2, 185.0, 147.2, 144.3, 135.1, 131.3, 130.7, 129.8, 128.8, 123.8, 32.4. **HRMS** (ESI) Calcd for $C_{15}H_{11}NNaO_4S$ $[M+Na]^+$ 324.0301, Found 324.0305.

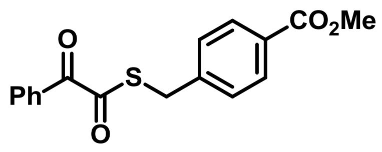

2x

Methyl 4-(((2-oxo-2-phenylacetyl)thio)methyl)benzoate (2x): Prepared following general procedure using 2-hydroxy-1-phenylethan-1-one **1a** (0.5 mmol, 68.1 mg), S_8 (2.0 mmol, 4.0 equiv), $KHCO_3$ (1.0 mmol, 2.0 equiv)，TBAB (32.3 mg, 0.1 mmol), H_2O (4 mmol, 20 equiv), CPME (4 mL) and methyl 4-(bromomethyl)benzoate (1.25 mmol, 2.5 equiv), the reaction was stirred at 90 ^oC for 12 hours giving **2x** (120.7 mg) in 77% yield as a yellow solid by column chromatography. **^{1}H NMR** (400 MHz, $CDCl_3$) δ 8.11 (d, *J* = 7.8 Hz, 2H), 7.98 (d, *J* = 8.2 Hz, 2H), 7.64 (t, *J* = 7.4 Hz, 1H), 7.52 – 7.39 (m, 4H), 4.27 (s, 2H), 3.89 (s, 3H). **^{13}C NMR** (100 MHz, $CDCl_3$) δ 191.6, 185.4, 166.6, 141.7, 134.9, 131.4, 130.7, 129.9, 129.3, 128.9, 128.8, 52.0, 32.8. **HRMS** (EI) Calcd for $C_{17}H_{14}O_4S$ 314.0613, Found 314.0608.

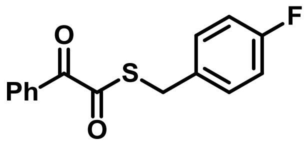

2y

2-Fluoro-9*H*-carbazole (2y): Prepared following general procedure using 2-hydroxy-1-phenylethan-1-one **1a** (0.5 mmol, 68.1 mg), S_8 (2.0 mmol, 4.0 equiv), $KHCO_3$ (1.0 mmol, 2.0 equiv)，TBAB (32.3 mg, 0.1 mmol), H_2O (4 mmol, 20 equiv), CPME (4 mL) and 1-(bromomethyl)-4-fluorobenzene (1.25 mmol, 2.5 equiv), the reaction was stirred at 90 ^oC for 12 hours giving **2y** (91.9 mg) in 67% yield by column chromatography. **^{1}H NMR** (400 MHz, $CDCl_3$) δ 8.16 as a yellow solid– 8.11 (m, 2H), 7.68 – 7.62 (m, 1H), 7.53 – 7.46 (m, 2H), 7.37 – 7.31 (m, 2H), 7.04 – 6.97 (m, 2H), 4.24 (s, 2H). **^{19}F NMR** (376 MHz, $CDCl_3$) δ -114.40. **^{13}C NMR** (100 MHz, $CDCl_3$) δ 191.9, 185.6, 162.1 (d, $^1J_{C\text{-}F}$ = 246.5 Hz), 134.9, 132.3 (d, $^4J_{C\text{-}F}$ = 3.3 Hz), 131.5, 130.7, 130.6 (d, $^3J_{C\text{-}F}$ = 8.2 Hz), 128.8, 115.5 (d, $^2J_{C\text{-}F}$ = 21.6 Hz), 32.4. **HRMS** (EI) Calcd for $C_{15}H_{11}O_2SF$ 274.0464, Found 274.0461.

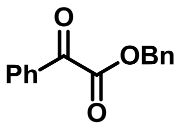

2aa

Benzyl 2-oxo-2-phenylacetate (2aa): Prepared following general procedure using 2-hydroxy-1-phenylethan-1-one **1a'** (67.1 mg, 0.5 mmol), S_8 (64.2 mg, 2 mmol), $KHCO_3$ (100 mg, 1 mmol), TBAB (32.3 mg, 0.1 mmol), H_2O (180 mg, 4 mmol), CPME (4 mL) and BnBr (0.75 mmol, 1.5 equiv), the reaction was stirred at 90 ^{o}C for 12 hours giving **2aa** (48.0 mg) in 40% yield as a yellow oil by column chromatography. **^{1}H NMR** (400 MHz, $CDCl_3$) δ 8.01-7.93 (m, 2H), 7.65 (t, J = 7.4 Hz, 1H), 7.53-7.35 (m, 7H), 5.42 (s, 2H). **^{13}C NMR** (100 MHz, $CDCl_3$) δ 186.0, 163.6, 134.9, 134.5, 132.4, 130.0, 128.9, 128.8, 128.7, 128.6, 67.7. **MS** (EI) m/z 240 (M^+).

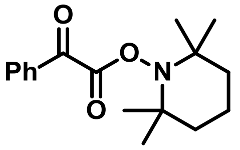

2ab

2,2,6,6-tetramethylpiperidin-1-yl 2-oxo-2-phenyl acetate (2ab): Prepared following general procedure using 2-hydroxy-1-phenylethan-1-one **1a** (68.1 mg, 0.5 mmol), S_8 (64.2 mg, 2 mmol), $KHCO_3$ (100 mg, 1 mmol), TBAB (32.3 mg, 0.1 mmol), H_2O (180 mg, 4 mmol), TEMPO (156.2 mg, 1.0 mmol), CPME (4 mL) and BnBr (0.75 mmol, 1.5 equiv), the reaction was stirred at 90 ^{o}C for 12 hours giving **2ab** (50.6 mg) in 35% yield as a yellow oil by column chromatography. **^{1}H NMR** (400 MHz, $CDCl_3$) δ 8.02 (d, J = 7.4 Hz, 2H), 7.67 (t, J = 7.4 Hz, 1H), 7.53 (t, J = 7.7 Hz, 2H), 1.79 – 1.57 (m, 6H), 1.23 (s, 6H), 1.21 (s, 6H). **^{13}C NMR** (100 MHz, $CDCl_3$) δ 186.6, 164.2, 134.9, 132.5, 129.9, 129.0, 77.0, 60.9, 39.3, 32.0, 20.6, 16.9. **HRMS** (EI) Calcd for $C_{17}H_{23}NO_3$ 289.1678, Found 289.1680.

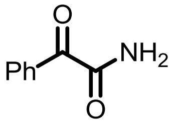

3a

2-Oxo-2-phenylacetamide (3a): Prepared following general procedure using α-ketothioester **2a** (51.3 mg, 0.2 mmol), ammonia (bubbling gas for 15 min), and THF (2 mL), the reaction was stirred at room temperature for 12 h giving **3a** (23.5 mg) in 79% yield as a yellow solid by column chromatography.

^{1}H NMR (400 MHz, $CDCl_3$) δ 8.43 – 8.15 (m, 2H), 7.79 – 7.58 (m, 1H), 7.47 (t, *J* = 7.7 Hz, 2H), 7.08 (s, 1H), 6.48 (s, 1H). **^{13}C NMR** (100 MHz, $CDCl_3$) δ 187.5, 164.2, 134.5, 132.9, 131.0, 128.5. **MS** (EI) m/z 149 (M^+).

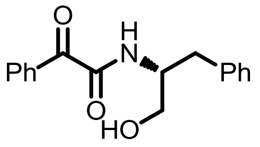

3b

(*S*)-*N*-(1-Hydroxy-3-phenylpropan-2-yl)-2-oxo-2-phenylacetamide (3b): Prepared following general procedure using α-ketothioester **2a** (51.3 mg, 0.2 mmol), D-phenylalaninol (30.3 mg, 0.2 mmol) and THF (2 mL), the reaction was stirred at room temperature for 12 h giving **3b** (48.0 mg) in 85% yield as a white solid by column chromatography. **^{1}H NMR** (400 MHz, $CDCl_3$) δ 8.18 (d, *J* = 7.4 Hz, 2H), 7.60 (t, *J* = 7.4 Hz, 1H), 7.48 – 7.35 (m, 3H), 7.34 – 7.21 (m, 5H), 4.37 – 4.25 (m, 1H), 3.72 (ddd, *J* = 16.1, 11.2, 4.3 Hz, 2H), 3.03 – 2.89 (m, 2H), 2.65 (s, 1H). **^{13}C NMR** (100 MHz, $CDCl_3$) δ 187.9, 162.1, 137.2, 134.4, 133.1, 131.1, 129.2, 128.7, 128.4, 126.7, 63.5, 52.8, 37.0. **MS** (EI) m/z 283 (M^+).

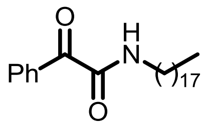

3c

***N*-Octadecyl-2-oxo-2-phenylacetamide (3c)**: Prepared following general procedure using α-ketothioester **2a** (51.3 mg, 0.2 mmol), octadecan-1-amine (53.9 mg, 0.2 mmol) and THF (2 mL), the reaction was stirred at room temperature for 12 h giving **3c** (52.6 mg) in 65% yield as a white solid by column chromatography. **^{1}H NMR** (400 MHz, $CDCl_3$) δ 8.37 – 8.29 (m, 2H), 7.61 (t, *J* = 7.4 Hz, 1H), 7.46 (t, *J* = 7.8 Hz, 2H), 7.12 (s, 1H), 3.37 (dd, *J* = 13.6, 6.9 Hz, 2H), 1.65 – 1.53 (m, 2H), 1.36 – 1.17 (m, 30H), 0.87 (t, *J* = 6.8 Hz, 3H). **^{13}C NMR** (100 MHz, $CDCl_3$) δ 187.9, 161.7, 134.3, 133.4, 131.2, 128.4, 39.4, 31.9, 29.7, 29.6(3), 29.6(0), 29.5(4), 29.5, 29.3(3), 29.3, 29.2, 26.9, 22.7, 14.1. **HRMS** (EI) Calcd for $C_{26}H_{43}NO_2$ 401.3294, Found 401.3296.

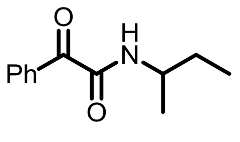

3d

***N*-(*sec*-Butyl)-2-oxo-2-phenylacetamide (3d)**: Prepared following general procedure using α-ketothioester **2a** (51.3 mg, 0.2 mmol), butan-2-amine (14.6 mg, 0.2 mmol) and THF (2 mL), the reaction was stirred at room temperature for 12 h giving **3d** (30.1 mg) in 74% yield as a colorless oil by column chromatography. **^{1}H NMR** (400 MHz, $CDCl_3$) δ 8.35 – 8.29 (m, 2H), 7.63 – 7.57 (m, 1H), 7.46 (t, *J* = 7.6 Hz, 2H), 6.91 (s, 1H), 4.04 – 3.93 (m, 1H), 1.57 (p, *J* = 7.3 Hz, 2H), 1.24 – 1.19 (m, 3H), 0.95 (t, *J* = 7.4 Hz, 3H). **^{13}C NMR** (100 MHz, $CDCl_3$) δ 188.1, 161.2, 134.2, 133.4, 131.1, 128.4, 46.9, 29.4, 20.1, 10.3. **MS** (EI) m/z 205 (M^+).

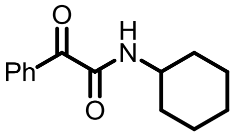

3e

***N*-Cyclohexyl-2-oxo-2-phenylacetamide (3e)**: Prepared following general procedure using α-ketothioester **2a** (51.3 mg, 0.2 mmol), cyclohexylamine (19.8 mg, 0.2 mmol) and THF (2 mL), the reaction was stirred at room temperature for 12 h giving **3e** (36.7 mg) in 80% yield as a yellow solid by column chromatography. **^{1}H NMR** (400 MHz, $CDCl_3$) δ 8.32 (d, *J* = 7.3 Hz, 2H), 7.59 (d, *J* = 7.4 Hz, 1H), 7.46 (t, *J* = 7.8 Hz, 2H), 6.98 (s, 1H), 3.90 – 3.78 (m, 1H), 2.03 – 1.92 (m, 2H), 1.80 – 1.71 (m, 2H), 1.68 – 1.58 (m, 1H), 1.47 – 1.33 (m, 2H), 1.32 – 1.18 (m, 3H). **^{13}C NMR** (100 MHz, $CDCl_3$) δ 188.1, 160.8, 134.2, 133.4, 131.1, 128.4, 48.4, 32.6, 25.4, 24.7. **MS** (EI) m/z 231(M^+).

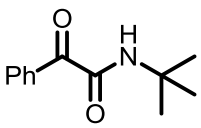

3f

***N*-(*tert*-Butyl)-2-oxo-2-phenylacetamide (3f)**: Prepared following general procedure using α-ketothioester **2a** (51.3 mg, 0.2 mmol), tert-butylamine (14.6 mg, 0.2 mmol), DMAP (7.4 mg, 0.06 mmol, 30 mol%) and THF (2 mL), the reaction was stirred at room

temperature for 34 h giving **3f** (23.6 mg) in 57% yield as a yellow solid by column chromatography. **^{1}H NMR** (400 MHz, $CDCl_3$) δ 8.29 (d, *J* = 7.7 Hz, 2H), 7.60 (t, *J* = 7.4 Hz, 1H), 7.46 (t, *J* = 7.7 Hz, 2H), 6.93 (s, 1H), 1.45 (s, 9H). **^{13}C NMR** (100 MHz, $CDCl_3$) δ 188.6, 161.1, 134.1, 133.4, 131.2, 128.3, 51.6, 28.4. **MS** (EI) m/z 205 (M^+).

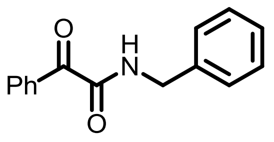

3g

***N*-Benzyl-2-oxo-2-phenylacetamide (3g)**: Prepared following general procedure using α-ketothioester **2a** (51.3 mg, 0.2 mmol), benzylamine (21.4 mg, 0.2 mmol) and THF (2 mL), the reaction was stirred at room temperature for 12 h giving **3g** (42.8 mg) in 89% yield as a white solid by column chromatography. **^{1}H NMR** (400 MHz, $CDCl_3$) δ 8.39 – 8.32 (m, 2H), 7.66 – 7.59 (m, 1H), 7.51 – 7.41 (m, 3H), 7.39 – 7.28 (m, 5H), 4.57 (d, *J* = 6.1 Hz, 2H). **^{13}C NMR** (100 MHz, $CDCl_3$) δ 187.5, 161.6, 137.1, 134.4, 133.3, 131.2, 128.8, 128.5, 127.8(4), 127.8, 43.4. **MS** (EI) m/z 239 (M^+).

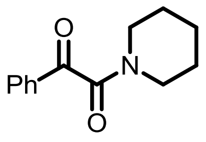

3h

1-Phenyl-2-(piperidin-1-yl)ethane-1,2-dione (3h): Prepared following general procedure using α-ketothioester **2a** (51.3 mg, 0.2 mmol), piperidine (17.0 mg, 0.2 mmol) and THF (2 mL), the reaction was stirred at room temperature for 12 h giving **3h** (39.4 mg) in 91% yield as a colorless oil by column chromatography. **^{1}H NMR** (400 MHz, $CDCl_3$) δ 7.97 – 7.88 (m, 2H), 7.66 – 7.57 (m, 1H), 7.49 (t, *J* = 7.7 Hz, 2H), 3.68 (s, 2H), 3.31 – 3.22 (m, 2H), 1.72 – 1.61 (m, 4H), 1.52 (s, 2H). **^{13}C NMR** (100 MHz, $CDCl_3$) δ 191.9, 165.4, 134.6, 133.2, 129.5, 128.9, 46.9, 42.0, 26.1, 25.3, 24.3. **MS** (EI) m/z 217 (M^+).

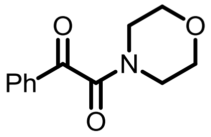

3i

1-Morpholino-2-phenylethane-1,2-dione (3i): Prepared following general procedure using α-ketothioester **2a** (51.3 mg, 0.2 mmol), morpholine (17.4 mg, 0.2 mmol) and THF (2 mL), the reaction was stirred at room temperature for 12 h giving **3i** (36.8 mg) in 84% yield as a yellow oil by column chromatography. **^{1}H NMR** (400 MHz, $CDCl_3$) δ 7.98 – 7.91 (m, 2H), 7.64 (t, J = 7.4 Hz, 1H), 7.51 (t, J = 7.7 Hz, 2H), 3.78 (s, 4H), 3.66 – 3.61 (m, 2H), 3.40 – 3.34 (m, 2H). **^{13}C NMR** (100 MHz, $CDCl_3$) δ 191.1, 165.4, 134.9, 133.0, 129.6, 129.0, 66.7, 66.6, 46.2, 41.6. **MS** (EI) m/z 219 (M^+).

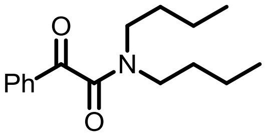

3j

***N*,*N*-Dibutyl-2-oxo-2-phenylacetamide (3j)**: Prepared following general procedure using α-ketothioester **2a** (51.3 mg, 0.2 mmol), dibutylamine (25.8 mg, 0.2 mmol) and THF (2 mL), the reaction was stirred at room temperature for 12 h giving **3j** (44.8 mg) in 86% yield as a yellow oil by column chromatography. **^{1}H NMR** (400 MHz, $CDCl_3$) δ 7.94 – 7.88 (m, 2H), 7.61 (t, J = 7.4 Hz, 1H), 7.48 (t, J = 7.8 Hz, 2H), 3.52 – 3.44 (m, 2H), 3.16 – 3.09 (m, 2H), 1.65 (tt, J = 7.8, 6.6 Hz, 2H), 1.52 (tt, J = 7.8, 6.7 Hz, 2H), 1.45 – 1.35 (m, 2H), 1.22 – 1.11 (m, 2H), 0.97 (t, J = 7.4 Hz, 3H), 0.79 (t, J = 7.4 Hz, 3H). **^{13}C NMR** (100 MHz, $CDCl_3$) δ 191.5, 167.0, 134.4, 133.3, 129.5, 128.8, 47.4, 44.0, 30. 6, 29.4, 20.2, 19.7, 13.8, 13.5. **MS** (EI) m/z 261(M^+).

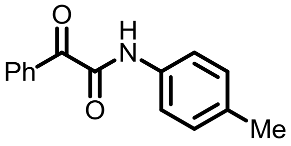

3k

2-Oxo-2-phenyl-*N*-(*p*-tolyl)acetamide (3k): Prepared following general procedure using α-ketothioester **2a** (51.3 mg, 0.2 mmol), *p*-toluidine (21.4 mg, 0.2 mmol) and THF (2 mL), the reaction

was stirred at room temperature for 34 h giving **3k** (38.2 mg) in 80% yield as a yellow solid by column chromatography. **^{1}H NMR** (400 MHz, $CDCl_3$) δ 8.95 (s, 1H), 8.44 – 8.38 (m, 2H), 7.68 – 7.62 (m, 1H), 7.59 (d, *J* = 8.4 Hz, 2H), 7.53 – 7.46 (m, 2H), 7.19 (d, *J* = 8.2 Hz, 2H), 2.35 (s, 3H). **^{13}C NMR** (100 MHz, $CDCl_3$) δ 187.5, 158.8, 135.0, 134.5, 134.1, 133.1, 131.4, 129.6, 128.5, 119.9, 20.9. **MS** (EI) m/z 239 (M^+).

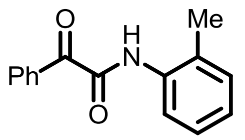

3l

2-Oxo-2-phenyl-*N*-(*o*-tolyl)acetamide (3l): Prepared following general procedure using α-ketothioester **2a** (25.6 mg, 0.1 mmol), *o*-toluidine (10.7 mg, 0.1 mmol), DMAP (12.2 mg, 0.1 mmol) and toluene (1 mL), the reaction was stirred at 80 ^{o}C for 48 h giving **3l** (21.5 mg) in 90% yield as a yellow solid by column chromatography. **^{1}H NMR** (400 MHz, $CDCl_3$) δ 8.93 (s, 1H), 8.49 – 8.40 (m, 2H), 8.11 (d, *J* = 8.0 Hz, 1H), 7.70 – 7.63 (m, 1H), 7.52 (t, *J* = 7.8 Hz, 2H), 7.32 – 7.22 (m, 2H), 7.14 (td, *J* = 7.5, 0.8 Hz, 1H), 2.38 (s, 3H). **^{13}C NMR** (100 MHz, $CDCl_3$) δ 187.5, 158.9, 134.6(2), 134.6(0), 133.1, 131.5, 130.7, 128.7, 128.6, 126.9, 125.7, 121.7, 17.6. **MS** (EI) m/z 239 (M^+).

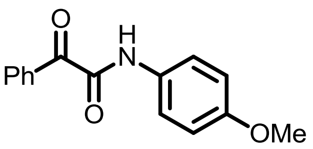

3m

***N*-(4-Methoxyphenyl)-2-oxo-2-phenylacetamide (3m)**: Prepared following general procedure usingα-ketothioester **2a** (51.3 mg, 0.2 mmol), 4-methoxyaniline (24.6 mg, 0.2 mmol) and THF (2 mL), the reaction was stirred at room temperature for 34 h giving **3m** (49.0 mg) in 96% yield as a yellow solid by column chromatography. **^{1}H NMR** (400 MHz, $CDCl_3$) δ 8.91 (s, 1H), 8.45 – 8.36 (m, 2H), 7.71 – 7.58 (m, 3H), 7.54 – 7.46 (m 2H), 6.97 – 6.88 (m, 2H), 3.82 (s, 3H). **^{13}C NMR** (100 MHz, $CDCl_3$) δ 187.6, 158.6, 157.0, 134.5, 133.1,

131.4, 129.7, 128.5, 121.4, 114.3), 55.4. **MS** (EI) m/z 255 (M^+).

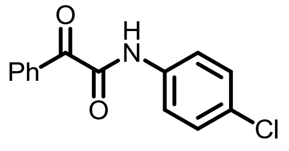
3n

***N*-(4-Chlorophenyl)-2-oxo-2-phenylacetamide (3n)**: Prepared following general procedure using α-ketothioester **2a** (25.6 mg, 0.1 mmol), 4-chloroaniline (12.8 mg, 0.1 mmol), DMAP (3.7 mg, 0.03 mmol, 30 mol%), K_2CO_3 (13.8 mg, 0.1 mmol) and toluene (1 mL), the reaction was stirred at 80 ^oC for 48 h giving **3n** (21.6 mg) in 83% yield as a yellow solid by column chromatography. **^{1}H NMR** (400 MHz, $CDCl_3$) δ 8.99 (s, 1H), 8.44 – 8.37 (m, 2H), 7.71 – 7.63 (m, 3H), 7.52 (t, *J* = 7.8 Hz, 2H), 7.39 – 7.33 (m, 2H). **^{13}C NMR** (100 MHz, $CDCl_3$) δ 187.0, 158.7, 135.2, 134.8, 132.9, 131.5, 130.4, 129.3 128.6, 121.1. **MS** (EI) m/z 259 (M^+).

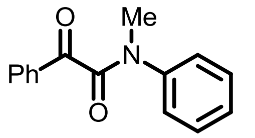
3o

***N*-Methyl-2-oxo-N,2-diphenylacetamide (3o)**: Prepared following general procedure using α-ketothioester **2p** (19.4 mg, 0.1 mmol), *N*-methylaniline (10.7 mg, 0.1 mmol), DMAP (3.7 mg, 0.03 mmol, 30 mol%) and toluene (1 mL), the reaction was stirred at 80 ^oC for 48 h giving **3o** (8.0 mg) in 33% yield as a yellow oil by column chromatography. **^{1}H NMR** (400 MHz, $CDCl_3$) δ 7.89 – 7.83 (m, 2H), 7.57 (t, *J* = 7.4 Hz, 1H), 7.44 (t, *J* = 7.8 Hz, 2H), 7.22 (t, *J* = 7.7 Hz, 3H), 7.16 – 7.11 (m, 2H), 3.49 (s, 3H). **^{13}C NMR** (100 MHz, $CDCl_3$) δ 190.8, 167.1, 141.20, 134.2, 133.6, 129.5, 129.4, 128.7, 128.1, 126.8, 36.2. **MS** (EI) m/z 239 (M^+).

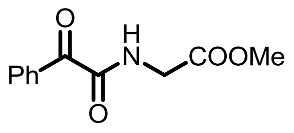

3p

Methyl 2-(2-oxo-2-phenylacetamido)acetate (3p): Prepared following general procedure using α-ketothioester **2a** (51.3 mg, 0.2 mmol), glycine hydrochloride (22.3 mg, 0.2 mmol), $KHCO_3$ (20 mg, 0.2 mmol), and THF (2 mL), the reaction was stirred at room temperature for 12 h giving **3p** (34.0 mg) in 77% yield as a yellow oil by column chromatography. **^{1}H NMR** (400 MHz, $CDCl_3$) δ 7.89 – 7.83 (m, 2H), 7.57 (t, *J* = 7.4 Hz, 1H), 7.44 (t, *J* = 7.8 Hz, 2H), 7.22 (t, *J* = 7.7 Hz, 3H), 7.16 – 7.11 (m, 2H), 3.49 (s, 3H). **^{13}C NMR** (100 MHz, $CDCl_3$) δ 8.32 – 8.26 (m, 2H), 7.68 – 7.57 (m, 2H), 7.49 – 7.43 (m, 2H), 4.16 (d, *J* = 5.7 Hz, 2H), 3.77 (s, 3H). **^{13}C NMR (100 MHz, $CDCl_3$)** δ 186.8, 169.4, 161.9, 134.5, 133.0, 131.1, 128.5, 52.5, 41.0. **MS (EI)** m/z 221 (M^+).

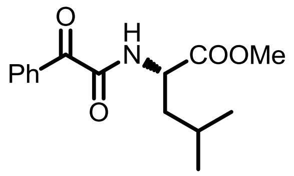

3q

(*S*)-Methyl 4-methyl-2-(2-oxo-2-phenylacetamido)pentanoate (3q): Prepared following general procedure using α-ketothioester **2a** (51.3 mg, 0.2 mmol), *L*-leucine hydrochloride (36.3 mg, 0.2 mmol), $KHCO_3$ (20 mg, 0.2 mmol), and THF (2 mL), the reaction was stirred at 80 ^{o}C for 48 h giving **3q** (45.9 mg) in 83% yield as a yellow oil by column chromatography. **^{1}H NMR** (400 MHz, $CDCl_3$) δ 8.35 – 8.25 (m, 2H), 7.60 (t, *J* = 7.4 Hz, 1H), 7.51 – 7.41 (m, 3H), 4.70 (td, *J* = 8.8, 5.0 Hz, 1H), 3.75 (s, 3H), 1.78 – 1.62 (m, 3H), 0.96 (t, *J* = 5.4 Hz, 6H). **^{13}C NMR** (100 MHz, $CDCl_3$) δ 187.0, 172.4, 161.4, 134.4, 133.1(3), 131.1(1), 128.4, 52.4, 50.7, 41.3, 24.8, 22.7, 21.8. **MS (EI)** m/z 277 (M^+).

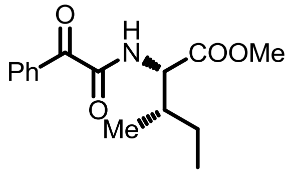

3r

(2*S*,3*S*)-Methyl 3-methyl-2-(2-oxo-2-phenylacetamido)pentanoate (3r): Prepared following general procedure using α-ketothioester **2a** (51.3 mg, 0.2 mmol), *L*-isoleucine hydrochloride (36.3 mg, 0.2 mmol), $KHCO_3$ (20 mg, 0.2 mmol), and THF (2 mL), the reaction was stirred at 80 °C for 48 h giving **3r** (41.5 mg) in 75% yield as a white solid by column chromatography. **^{1}H NMR** (400 MHz, $CDCl_3$) δ 8.31 (dt, *J* = 8.5, 1.5 Hz, 2H), 7.64 – 7.58 (m, 1H), 7.54 (d, *J* = 8.5 Hz, 1H), 7.49 – 7.43 (m, 1H), 4.64 (dd, *J* = 8.9, 5.0 Hz, 1H), 3.76 (s, 3H), 2.06 – 1.94 (m, 1H), 1.53-1.47 (m, 1H), 1.28-1.22 (m, 1H), 0.99 – 0.91 (m, 6H). **^{13}C NMR** (100 MHz, $CDCl_3$) δ 187.0, 171.4, 161.4, 134.4, 133.2, 131.1, 128.5, 56.5, 52.2, 37.9, 25.1, 15.5, 11.5. **MS** (EI) m/z 277 (M^+).

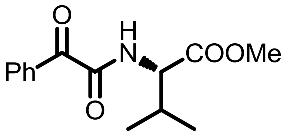

3s

(*S*)-Methyl 3-methyl-2-(2-oxo-2-phenylacetamido)butanoate (3s): Prepared following general procedure using α-ketothioester **2a** (51.3 mg, 0.2 mmol), *L*-Valine methyl ester hydrochloride (33.6 mg, 0.2 mmol), $KHCO_3$ (20 mg, 0.2 mmol), and THF (2 mL), the reaction was stirred at 80 °C for 48 h giving **3s** (40.4 mg) in 77% yield as a white solid by column chromatography. **^{1}H NMR** (400 MHz, $CDCl_3$) δ 8.34 – 8.28 (m, 2H), 7.64 – 7.58 (m, 1H), 7.52 (d, *J* = 8.4 Hz, 1H), 7.49 – 7.43 (m, 2H), 4.60 (dd, *J* = 9.1, 5.0 Hz, 1H), 3.77 (s, 3H), 2.33 – 2.23 (m, 1H), 0.99 (dd, *J* = 11.9, 6.9 Hz, 6H). **^{13}C NMR (100 MHz, $CDCl_3$)** δ 187.0, 171.4, 161.5, 134.4, 133.2, 131.1, 128.5, 57.3, 52.3, 31.4, 19.0, 17.7. **MS (EI)** m/z 263 (M^+).

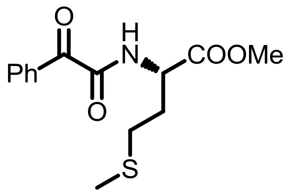

3t

(*S*)-Methyl 4-(methylthio)-2-(2-oxo-2-phenylacetamido)butanoate (3t): Prepared following general procedure using α-ketothioester **2a** (51.3 mg, 0.2 mmol), *L*-Methionine methyl ester hydrochloride (40.0 mg, 0.2 mmol), $KHCO_3$ (20 mg, 0.2 mmol), and THF (2 mL), the reaction was stirred at 80 ^oC for 48 h giving **3t** (43.7 mg) in 74% yield as a yellow oil by column chromatography. **^{1}H NMR** (400 MHz, $CDCl_3$) δ 8.30 (d, *J* = 7.5 Hz, 2H), 7.71 (d, *J* = 7.9 Hz, 1H), 7.61 (t, *J* = 7.4 Hz, 1H), 7.46 (t, *J* = 7.8 Hz, 2H), 4.80 (td, *J* = 7.8, 5.2 Hz, 1H), 3.78 (s, 3H), 2.56 (t, *J* = 7.4 Hz, 2H), 2.25 (dtd, *J* = 12.8, 7.5, 5.2 Hz, 1H), 2.15 – 2.04 (m, 4H). **^{13}C NMR** (100 MHz, $CDCl_3$) δ 186.8, 171.4, 161.5, 134.5, 133.0, 131.1, 128.5, 52.6, 51.5, 31.4, 29.9, 15.4. **HRMS** (EI) Calcd for $C_{14}H_{17}NO_4S$ 295.0878, Found 295.0876.

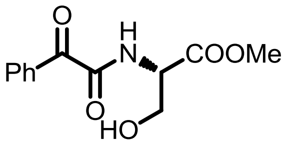

3u

(*S*)-Methyl 3-hydroxy-2-(2-oxo-2-phenylacetamido)propanoate (3u): Prepared following general procedure using α-ketothioester **2a** (51.3 mg, 0.2 mmol), *L*-Serine methyl ester hydrochloride (31.1 mg, 0.2 mmol), $KHCO_3$ (20 mg, 0.2 mmol), and THF (2 mL), the reaction was stirred at 80 ^oC for 48 h giving **3u** (43.9 mg) in 74% yield as a yellow oil by column chromatography. **^{1}H NMR** (400 MHz, $CDCl_3$) δ 8.27 (dd, *J* = 8.3, 1.2 Hz, 2H), 7.93 (d, *J* = 7.6 Hz, 1H), 7.65 – 7.58 (m, 1H), 7.46 (t, *J* = 7.8 Hz, 2H), 4.78 – 4.71 (m, 1H), 4.10 (dd, *J* = 11.4, 3.8 Hz, 1H), 3.99 (dd, *J* = 11.4, 3.5 Hz, 1H), 3.80 (s, 3H), 2.69 (s, 1H). **^{13}C NMR** (100 MHz, $CDCl_3$) δ 187.2, 170.1, 162.1, 134.6, 133.0, 131.1, 128.5, 62.7, 54.6, 52.9. **HRMS** (EI) Calcd for $C_{12}H_{13}NO_5$ 251.0794, Found 251.0791.

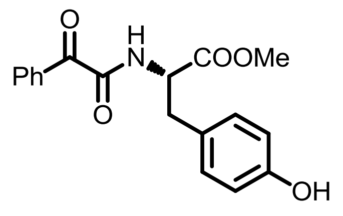

3v

(*S*)-Methyl 3-(4-hydroxyphenyl)-2-(2-oxo-2-phenylacetamido)propanoate (3v): Prepared following general procedure using α-ketothioester **2a** (51.3 mg, 0.2 mmol), *L*-Tyrosine methyl ester hydrochloride (46.3 mg, 0.2 mmol), $KHCO_3$ (20 mg, 0.2 mmol), and THF (2 mL), the reaction was stirred at 80 ^{o}C for 48 h giving **3v** (58.1 mg) in 89% yield as a yellow oil by column chromatography. **^{1}H NMR** (400 MHz, $CDCl_3$) δ 8.17 (d, *J* = 8.2 Hz, 2H), 7.59 (t, *J* = 7.4 Hz, 1H), 7.53 (d, *J* = 8.0 Hz, 1H), 7.43 (t, *J* = 7.7 Hz, 2H), 6.98 (d, *J* = 8.4 Hz, 2H), 6.71 (d, *J* = 8.4 Hz, 2H), 6.56 (s, 1H), 4.94 – 4.86 (m, 1H), 3.74 (s, 3H), 3.10 (ddd, *J* = 20.8, 14.1, 6.1 Hz, 2H). **^{13}C NMR (100 MHz, $CDCl_3$)** δ 187.1, 171.3, 161.7, 155.3, 134.6, 132.8, 131.0, 130.3, 128.5, 126.7, 115.7, 53.4, 52.6, 37.1. **HRMS (EI)** Calcd for $C_{18}H_{17}NO_5$ 327.1107, Found 327.1110.

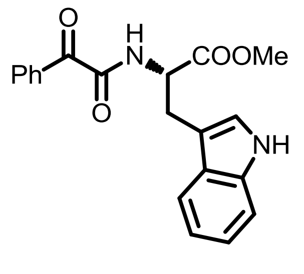

3w

(*S*)-Methyl 3-(1*H*-indol-3-yl)-2-(2-oxo-2-phenyl acetamido)propanoate (3w): Prepared following general procedure using α-ketothioester **2a** (51.3 mg, 0.2 mmol), *L*-Tryptophan methyl ester hydrochloride (50.9 mg, 0.2 mmol), $KHCO_3$ (20 mg, 0.2 mmol), and THF (2 mL), the reaction was stirred at 80 ^{o}C for 48 h giving **3w** (55.5 mg) in 79% yield as a yellow oil by column chromatography. **^{1}H NMR** (400 MHz, $CDCl_3$) δ 8.30 (s, 1H), 8.27 – 8.20 (m, 2H), 7.64 – 7.53 (m, 3H), 7.44 (dd, *J* = 10.8, 4.9 Hz, 2H), 7.32 (d, *J* = 8.1 Hz, 1H), 7.21 – 7.15 (m, 1H), 7.14 – 7.08 (m, 1H), 7.02 (d, *J* = 2.4 Hz, 1H), 5.01 (dt, *J* = 8.2, 5.7 Hz, 1H), 3.71 (s, 3H), 3.42 (d, *J* = 5.7 Hz, 2H). **^{13}C NMR** (100 MHz, $CDCl_3$) δ 187.1, 171.5, 161.5, 136.1, 134.4, 133.0, 131.0, 128.4, 127.2, 123.0, 122.2, 119.6, 118.4, 111.3, 109.3, 52.8, 52.5, 27.6. **HRMS** (EI) Calcd for $C_{20}H_{18}N_2O_4$ 350.1267, Found 350.1271.

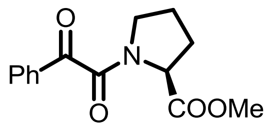

3x

(*S*)-Methyl 1-(2-oxo-2-phenylacetyl)pyrrolidine-2-carboxylate (3x): Prepared following general procedure using α-ketothioester **2a** (51.3 mg, 0.2 mmol), *L*-Proline methyl ester hydrochloride (33.1 mg, 0.2 mmol), $KHCO_3$ (20 mg, 0.2 mmol), and THF (2 mL), the reaction was stirred at 80 ^oC for 48 h giving **3x** (36.4 mg) in 70% yield as a colorless oil by column chromatography. Rotamer A **^{1}H NMR** (400 MHz, $CDCl_3$) δ 8.09 – 8.05 (m, 2H), 7.66 – 7.61 (m, 1H), 7.51 (dd, *J* = 10.7, 4.7 Hz, 2H), 4.66 (dd, *J* = 8.8, 3.9 Hz, 1H), 3.81 (s, 3H), 3.63 – 3.49 (m, 2H), 2.37 – 2.15 (m, 2H), 2.00 – 1.94 (m, 2H). **^{13}C NMR** (100 MHz, $CDCl_3$) δ 190.2, 171.9, 164.4, 134.2, 133.2, 130.4, 128.5, 58.2, 52.3, 46.5, 31.0, 24.6. Rotamer B **^{1}H NMR** (400 MHz, $CDCl_3$) δ 8.04 – 8.00 (m, 2H), 7.60 – 7.57 (m, 1H), 7.46 (dd, *J* = 10.7, 4.8 Hz, 2H), 4.73 (dd, *J* = 8.4, 3.6 Hz, 1H), 3.85 – 3.68 (m, 2H), 3.46 (s, 3H), 2.12 – 2.01 (m, 4H). **^{13}C NMR** (100 MHz, $CDCl_3$) δ 190.2, 171.9, 164.4, 134.2, 133.2, 130.4, 128.5, 59.3, 52.3, 46.5, 31.0, 22.4. **MS** (EI) m/z 261 (M^+).

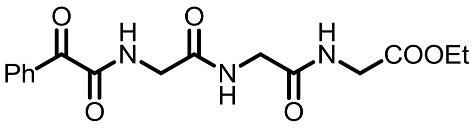

3y

Ethyl 2-(2-(2-(2-oxo-2-phenylacetamido)acetamido)acetamido)acetate (3y): Prepared following general procedure using α-ketothioester **2a** (51.3 mg, 0.2 mmol), thrglycine ethyl ester (43.4 mg, 0.2 mmol), $KHCO_3$ (20 mg, 0.2 mmol), and THF (2 mL), the reaction was stirred at 80 ^oC for 48 h giving **3y** (48.2 mg) in 69% yield as a white solid by column chromatography. **^{1}H NMR** (400 MHz, $CDCl_3$) δ 8.25 (dd, *J* = 8.3, 1.2 Hz, 2H), 8.02 (t, *J* = 5.3 Hz, 1H), 7.64 – 7.58 (m, 1H), 7.45 (dd, *J* = 10.8, 4.9 Hz, 2H), 7.26 (s, 1H), 7.02 (s, 1H), 4.18 – 4.10 (m, 4H), 4.06 – 3.97 (m, 4H), 1.24 (t, *J* = 7.1 Hz, 3H).**^{13}C NMR** (100 MHz, $CDCl_3$) δ 187.1, 169.8, 169.0, 168.8, 162.8, 134.5, 133.1, 131.1, 128.5, 61.6, 42.9, 42.8, 41.3, 14.1.

HRMS (ESI) Calcd for $C_{16}H_{19}N_3NaO_6$ $[M+Na]^+$ 372.1166, Found 372.1162.

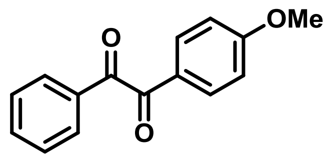

5a

1-(4-Methoxyphenyl)-2-phenylethane-1,2-dione (5a): Prepared following general procedure using α-ketothioesters **2p** (19.4 mg, 0.1 mmol), 4-methoxyphenyl borates (0.1 mmol, 1 equiv), $Pd_2(dba)_3$ (0.0025 mmol, 2.5 mol%), 4,4'-dimethoxy-2,2'-bipyridine (0.01 mmol, 10 mol%), CuTc (0.1 mmol, 1 equiv), K_2CO_3 (0.15 mmol, 1.5 equiv), anhydrous Na_2SO_4 (0.15 mmol, 1.5 equiv) and DMF (1 mL), the reaction was stirred at 45 ^oC for 12 h giving **5a** (20.7 mg) in 86% yield as a yellow oil by column chromatography. **^{1}H NMR** (400 MHz, $CDCl_3$) δ 8.01 – 7.91 (m, 4H), 7.68 – 7.61 (m, 1H), 7.53 – 7.47 (m, 2H), 7.01 – 6.94 (m, 2H), 3.89 (s, 3H). **^{13}C NMR** (100 MHz, $CDCl_3$) δ 194.8, 193.2, 165.0, 134.7, 133.1 132.4, 129.9, 128.9, 126.1, 114.3, 55.6. **MS (EI)** m/z 240 (M^+).

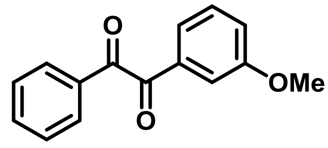

5b

1-(3-Methoxyphenyl)-2-phenylethane-1,2-dione (5b): Prepared following general procedure using α-ketothioesters **2p** (19.4 mg, 0.1 mmol), 3-methoxyphenyl borates (0.1 mmol, 1 equiv), $Pd_2(dba)_3$ (0.0025 mmol, 2.5 mol%), 4,4'-dimethoxy-2,2'-bipyridine (0.01 mmol, 10 mol%), CuTc (0.1 mmol, 1 equiv), K_2CO_3 (0.15 mmol, 1.5 equiv), anhydrous Na_2SO_4 (0.15 mmol, 1.5 equiv) and DMF (1 mL), the reaction was stirred at 45 ^oC for 12 h giving **5b** (18.8 mg) in 78% yield as a yellow solid by column chromatography. **^{1}H NMR** (400 MHz, $CDCl_3$) δ 7.99 – 7.94 (m, 2H), 7.69 – 7.63 (m, 1H), 7.56 – 7.45 (m, 4H), 7.40 (t, J = 7.9 Hz, 1H), 7.23 – 7.18 (m, 1H), 3.87 (s, 3H).**^{13}C NMR** (100 MHz, $CDCl_3$) δ 194.50, 194.48, 160.0, 134.9, 134.2, 132.9, 130.0, 129.9, 129.0, 123.2, 121.9, 112.7, 55.5. **MS (EI)** m/z 240 (M^+).

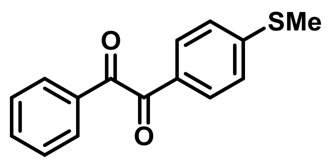

5c

1-(4-(Methylthio)phenyl)-2-phenylethane-1,2-dione (5c) Prepared following general procedure using α-ketothioesters **2p** (19.4 mg, 0.1 mmol), 4-(methylthio)phenyl borates (0.1 mmol, 1 equiv), $Pd_2(dba)_3$ (0.0025 mmol, 2.5 mol%), 4,4'-dimethoxy-2,2'-bipyridine (0.01 mmol, 10 mol%), CuTc (0.1 mmol, 1 equiv), K_2CO_3 (0.15 mmol, 1.5 equiv), anhydrous Na_2SO_4 (0.15 mmol, 1.5 equiv) and DMF (1 mL), the reaction was stirred at 45 ^{o}C for 12 h giving **5c** (18.7 mg) in 73% yield as a yellow solid by column chromatography. **^{1}H NMR** (400 MHz, $CDCl_3$) δ 8.00 – 7.93 (m, 2H), 7.87 (d, *J* = 8.6 Hz, 2H), 7.65 (t, *J* = 7.4 Hz, 1H), 7.51 (t, *J* = 7.8 Hz, 2H), 7.29 (d, *J* = 8.6 Hz, 2H), 2.52 (s, 3H). **^{13}C NMR** (100 MHz, $CDCl_3$) δ 194.5, 193.5, 148.9, 134.8, 133.0, 130.1, 129.9, 129.2, 129.0, 125.0, 14.6. **MS (EI)** m/z 256 (M^+).

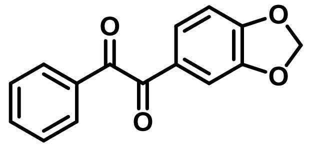

5d

1-(Benzo[d][1,3]dioxol-5-yl)-2-phenylethane-1,2-dione (5d) Prepared following general procedure using α-ketothioesters **2p** (19.4 mg, 0.1 mmol), piperonyl borates (0.1 mmol, 1 equiv), $Pd_2(dba)_3$ (0.0025 mmol, 2.5 mol%), 4,4'-dimethoxy-2,2'-bipyridine (0.01 mmol, 10 mol%), CuTc (0.1 mmol, 1 equiv), K_2CO_3 (0.15 mmol, 1.5 equiv), anhydrous Na_2SO_4 (0.15 mmol, 1.5 equiv) and DMF (1 mL), the reaction was stirred at 45 ^{o}C for 12 h giving **5d** (20.4 mg) in 80% yield as a yellow solid by column chromatography. **^{1}H NMR** (400 MHz, $CDCl_3$) δ 7.96 (d, *J* = 7.3 Hz, 2H), 7.65 (t, *J* = 7.4 Hz, 1H), 7.55 – 7.46 (m, 4H), 6.87 (d, *J* = 8.6 Hz, 1H), 6.09 (s, 2H). **^{13}C NMR** (100 MHz, $CDCl_3$) δ 194.6, 192.8, 153.5, 148.6, 134.8, 133.1, 129.9, 129.0, 127.9, 127.8, 108.4, 108.3, 102.2. **MS (EI)** m/z 254 (M^+).

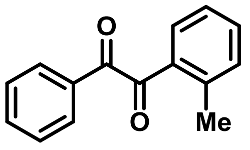

5e

1-Phenyl-2-(o-tolyl)ethane-1,2-dione (5e) Prepared following general procedure using α-ketothioesters **2p** (19.4 mg, 0.1 mmol), 2-tolyl borates (0.1 mmol, 1 equiv), $Pd_2(dba)_3$ (0.0025 mmol, 2.5 mol%), 4,4'-dimethoxy-2,2'-bipyridine (0.01 mmol, 10 mol%), CuTc (0.1 mmol, 1 equiv), K_2CO_3 (0.15 mmol, 1.5 equiv), anhydrous Na_2SO_4 (0.15 mmol, 1.5 equiv) and DMF (1 mL), the reaction was stirred at 45 ^oC for 12 h giving **5e** (12.5 mg) in 56% yield as a yellow oil by column chromatography. **^{1}H NMR** (400 MHz, $CDCl_3$) δ 8.00 – 7.95 (m, 2H), 7.69 – 7.62 (m, 2H), 7.55 – 7.47 (m, 3H), 7.35 (d, *J* = 7.7 Hz, 1H), 7.30 – 7.24 (m, 1H), 2.71 (s, 3H). **^{13}C NMR** (100 MHz, $CDCl_3$) δ 196.8, 194.8, 141.4, 134.7, 133.8, 133.1, 132.6, 131.7, 129.9, 129.0, 126.0, 21.9. **MS (EI)** m/z 224 (M^+).

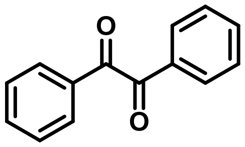

5f

Benzil (5f) Prepared following general procedure using α-ketothioesters **2p** (19.4 mg, 0.1 mmol), phenyl borates (0.1 mmol, 1 equiv), $Pd_2(dba)_3$ (0.0025 mmol, 2.5 mol%), 4,4'-dimethoxy-2,2'-bipyridine (0.01 mmol, 10 mol%), CuTc (0.1 mmol, 1 equiv), K_2CO_3 (0.15 mmol, 1.5 equiv), anhydrous Na_2SO_4 (0.15 mmol, 1.5 equiv) and DMF (1 mL), the reaction was stirred at 45 ^oC for 12 h giving **5f** (15.4 mg) in 73% yield as a yellow solid by column chromatography. **^{1}H NMR** (400 MHz, $CDCl_3$) δ 8.02 – 7.93 (m, 4H), 7.70 – 7.62 (m, 2H), 7.56 – 7.48 (m, 4H). **^{13}C NMR** (100 MHz, $CDCl_3$) δ 194.6, 134.9, 132.9, 129.9, 129.0. **MS (EI)** m/z 210 (M^+).

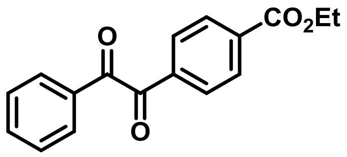

5g

Ethyl 4-(2-oxo-2-phenylacetyl)benzoate (5g) Prepared following general procedure using α-ketothioesters **2p** (19.4 mg, 0.1 mmol), (4-(ethoxycarbonyl)phenyl) borates (0.1 mmol, 1 equiv), $Pd_2(dba)_3$ (0.0025 mmol, 2.5 mol%), 4,4'-dimethoxy-2,2'-bipyridine (0.01 mmol, 10 mol%), CuTc (0.1 mmol, 1 equiv), K_2CO_3 (0.15 mmol, 1.5 equiv), anhydrous Na_2SO_4 (0.15 mmol, 1.5 equiv) and DMF (1 mL), the reaction was stirred at 45 ^oC for 12 h giving **5g** (12.4 mg) in 44% yield as a yellow oil by column chromatography. **^{1}H NMR** (400 MHz, $CDCl_3$) δ 8.17 (d, *J* = 8.5 Hz, 2H), 8.04 (d, *J* = 8.5 Hz, 2H), 7.98 (dd, *J* = 8.2, 1.0 Hz, 2H), 7.68 (t, *J* = 7.4 Hz, 1H), 7.53 (t, *J* = 7.8 Hz, 2H), 4.41 (q, *J* = 7.1 Hz, 2H), 1.41 (t, *J* = 7.1 Hz, 3H). **^{13}C NMR** (100 MHz, $CDCl_3$) δ 193.8, 193.7, 165.4, 135.9, 135.7, 135.1, 132.7, 130.04, 129.95, 129.7, 129.1, 61.7, 14.2. **MS (EI)** m/z 282 (M^+).

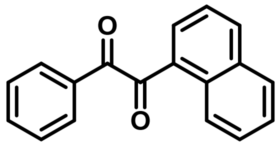

5h

1-(Naphthalen-1-yl)-2-phenylethane-1,2-dione (5h) Prepared following general procedure using α-ketothioesters **2p** (19.4 mg, 0.1 mmol), naphthalen-1-ylborates (0.1 mmol, 1 equiv), $Pd_2(dba)_3$ (0.0025 mmol, 2.5 mol%), 4,4'-dimethoxy-2,2'-bipyridine (0.01 mmol, 10 mol%), CuTc (0.1 mmol, 1 equiv), K_2CO_3 (0.15 mmol, 1.5 equiv), anhydrous Na_2SO_4 (0.15 mmol, 1.5 equiv) and DMF (1 mL), the reaction was stirred at 45 ^oC for 12 h giving **5h** (14.2 mg) in 55% yield as a yellow solid by column chromatography. **^{1}H NMR** (400 MHz, $CDCl_3$) δ 9.32 (d, *J* = 8.6 Hz, 1H), 8.13 (d, *J* = 8.2 Hz, 1H), 8.06 – 8.01 (m, 2H), 7.97 – 7.90 (m, 2H), 7.79 – 7.73 (m, 1H), 7.69 – 7.61 (m, 2H), 7.56 – 7.47 (m, 3H). **^{13}C NMR** (100 MHz, $CDCl_3$) δ 197.1, 194.6, 136.0, 135.1, 134.7, 134.0, 133.3, 130.9, 130.0, 129.5, 129.0, 128.8, 128.5, 127.1, 125.9, 124.4. **MS (EI)** m/z 260 (M^+).

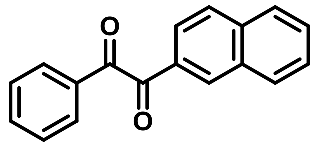

5i

1-(Naphthalen-2-yl)-2-phenylethane-1,2-dione (5i) Prepared following general procedure using α-ketothioesters **2p** (19.4 mg, 0.1 mmol), naphthalen-2-ylborates (0.1 mmol, 1 equiv), $Pd_2(dba)_3$ (0.0025 mmol, 2.5 mol%), 4,4'-dimethoxy-2,2'-bipyridine (0.01 mmol, 10 mol%), CuTc (0.1 mmol, 1 equiv), K_2CO_3 (0.15 mmol, 1.5 equiv), anhydrous Na_2SO_4 (0.15 mmol, 1.5 equiv) and DMF (1 mL), the reaction was stirred at 45 ^oC for 12 h giving **5i** (15.5 mg) in 60% yield as a yellow solid by column chromatography. **^{1}H NMR** (400 MHz, $CDCl_3$) δ 8.41 (s, 1H), 8.11 (dd, *J* = 8.6, 1.6 Hz, 1H), 8.04 (dd, *J* = 8.3, 1.2 Hz, 2H), 7.97 (d, *J* = 8.6 Hz, 1H), 7.91 (t, *J* = 7.4 Hz, 2H), 7.71 – 7.62 (m, 2H), 7.59 – 7.50 (m, 3H). **^{13}C NMR** (100 MHz, $CDCl_3$) δ 194.6, 136.3, 134.9, 133.6, 133.0, 132.3, 130.2, 123.0, 129.9, 129.5, 129.2, 129.0, 127.9, 127.2, 123.6. **MS (EI)** m/z 260 (M^+).

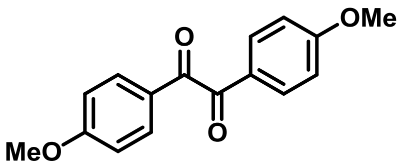

5j

1,2-Bis(4-methoxyphenyl)ethane-1,2-dione (5j) Prepared following general procedure using the corresponding α-ketothioesters (22.4 mg, 0.1 mmol), 4-methoxyphenyl borates (0.1 mmol, 1 equiv), $Pd_2(dba)_3$ (0.0025 mmol, 2.5 mol%), 4,4'-dimethoxy-2,2'-bipyridine (0.01 mmol, 10 mol%), CuTc (0.1 mmol, 1 equiv), K_2CO_3 (0.15 mmol, 1.5 equiv), anhydrous Na_2SO_4 (0.15 mmol, 1.5 equiv) and DMF (1 mL), the reaction was stirred at 45 ^oC for 12 h giving **5j** (17.8 mg) in 66% yield as a yellow solid by column chromatography. **^{1}H NMR** (400 MHz, $CDCl_3$) δ 7.97 – 7.92 (m, 4H), 6.99 – 6.94 (m, 4H), 3.88 (s, 6H). **^{13}C NMR** (100 MHz, $CDCl_3$) δ 193.5, 164.8, 132.4, 126.3, 114.3, 55.6. **MS (EI)** m/z 270 (M^+).

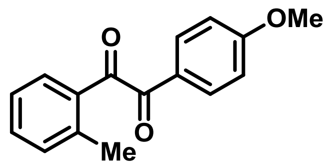

5k

1-(4-Methoxyphenyl)-2-(o-tolyl)ethane-1,2-dione (5k) Prepared following general procedure using the corresponding α-ketothioesters (20.8 mg, 0.1 mmol), 4-methoxyphenyl borates (0.1 mmol, 1 equiv), $Pd_2(dba)_3$ (0.0025 mmol, 2.5 mol%), 4,4'-dimethoxy-2,2'-bipyridine (0.01 mmol, 10 mol%), CuTc (0.1 mmol, 1 equiv), K_2CO_3 (0.15 mmol, 1.5 equiv), anhydrous Na_2SO_4 (0.15 mmol, 1.5 equiv) and DMF (1 mL), the reaction was stirred at 45 ^oC for 12 h giving **5k** (20.0 mg) in 79% yield as a yellow solid by column chromatography. **^{1}H NMR** (400 MHz, $CDCl_3$) δ 7.98 – 7.91 (m, 2H), 7.64 (d, *J* = 7.8 Hz, 1H), 7.51 – 7.45 (m, 1H), 7.33 (d, *J* = 7.6 Hz, 1H), 7.29 – 7.22 (m, 1H), 7.01 – 6.95 (m, 2H), 3.89 (s, 3H), 2.70 (s, 3H). **^{13}C NMR** (100 MHz, $CDCl_3$) δ 197.1, 193.5, 164.8, 141.3, 133.6, 133.0, 132.5, 132.4, 132.0, 126.1, 125.9, 114.3, 55.6, 21.9. **MS (EI)** m/z 254 (M^+).

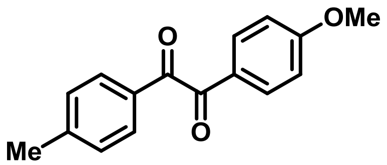

5l

1-(4-Methoxyphenyl)-2-(*p*-tolyl)ethane-1,2-dione (5l) Prepared following general procedure using the corresponding α-ketothioesters (20.8 mg, 0.1 mmol), 4-methoxyphenyl borates (0.1 mmol, 1 equiv), $Pd_2(dba)_3$ (0.0025 mmol, 2.5 mol%), 4,4'-dimethoxy-2,2'-bipyridine (0.01 mmol, 10 mol%), CuTc (0.1 mmol, 1 equiv), K_2CO_3 (0.15 mmol, 1.5 equiv), anhydrous Na_2SO_4 (0.15 mmol, 1.5 equiv) and DMF (1 mL), the reaction was stirred at 45 ^oC for 12 h giving **5l** (20.9 mg) in 82% yield as a yellow solid by column chromatography. **^{1}H NMR** (400 MHz, $CDCl_3$) δ 7.97 – 7.91 (m, 2H), 7.86 (d, *J* = 8.2 Hz, 2H), 7.30 (d, *J* = 8.0 Hz, 2H), 7.00 – 6.94 (m, 2H), 3.88 (s, 3H), 2.43 (s, 3H). **^{13}C NMR** (100 MHz, $CDCl_3$) δ 194.6, 193.4, 164.9, 146.0, 132.4, 130.7, 130.0, 129.7, 126.1, 114.3, 55.6, 21.9. **MS (EI)** m/z 254 (M^+).

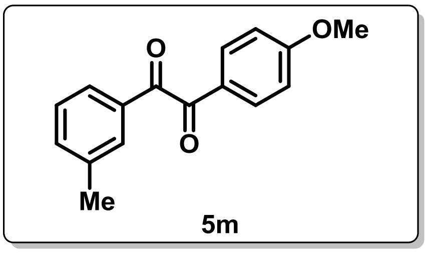

1-(4-Methoxyphenyl)-2-(*m*-tolyl)ethane-1,2-dione (5m) Prepared following general procedure using the corresponding α-ketothioesters (20.8 mg, 0.1 mmol), 4-methoxyphenyl borates (0.1 mmol, 1 equiv), $Pd_2(dba)_3$ (0.0025 mmol, 2.5 mol%), 4,4'-dimethoxy-2,2'-bipyridine (0.01 mmol, 10 mol%), CuTc (0.1 mmol, 1 equiv), K_2CO_3 (0.15 mmol, 1.5 equiv), anhydrous Na_2SO_4 (0.15 mmol, 1.5 equiv) and DMF (1 mL), the reaction was stirred at 45 ^oC for 12 h giving **5m** (19.3 mg) in 76% yield as a yellow oil by column chromatography. **^{1}H NMR** (400 MHz, $CDCl_3$) δ 7.98 – 7.91 (m, 2H), 7.76 (d, *J* = 7.5 Hz, 2H), 7.46 (d, *J* = 7.4 Hz, 1H), 7.38 (t, *J* = 7.8 Hz, 1H), 7.00 – 6.94 (m, 2H), 3.88 (s, 3H), 2.40 (s, 3H). **^{13}C NMR** (100 MHz, $CDCl_3$) δ 197.1, 193.5, 164.8, 141.3, 133.6, 133.0, 132.5, 132.4, 132.0, 126.1, 125.9, 114.3, 55.6, 21.9. **MS (EI)** m/z 254 (M^+).

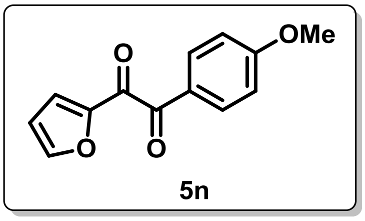

1-(Furan-2-yl)-2-(4-methoxyphenyl)ethane-1,2-dione (5n) Prepared following general procedure using the corresponding α-ketothioesters (18.4 mg, 0.1 mmol), 4-methoxyphenyl borates (0.1 mmol, 1 equiv), $Pd_2(dba)_3$ (0.0025 mmol, 2.5 mol%), 4,4'-dimethoxy-2,2'-bipyridine (0.01 mmol, 10 mol%), CuTc (0.1 mmol, 1 equiv), K_2CO_3 (0.15 mmol, 1.5 equiv), anhydrous Na_2SO_4 (0.15 mmol, 1.5 equiv) and DMF (1 mL), the reaction was stirred at 45 ^oC for 12 h giving **5n** (19.3 mg) in 84% yield as a yellow solid by column chromatography. **^{1}H NMR** (400 MHz, $CDCl_3$) δ 8.05 – 7.97 (m, 2H), 7.77 – 7.72 (m, 1H), 7.37 (d, *J* = 3.6 Hz, 1H), 7.01 – 6.93 (m, 2H), 6.61 (dd, *J* = 3.6, 1.7 Hz, 1H), 3.89 (s, 3H). **^{13}C NMR** (100 MHz, $CDCl_3$) δ 190.1, 180.8, 165.0, 150.0, 149.0, 132.7, 125.5, 123.2, 114.2, 112.9, 55.6. **MS (EI)** m/z 230 (M^+).

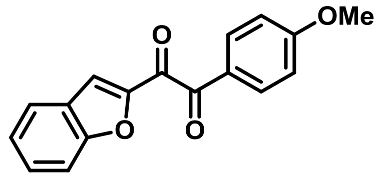

5o

1-(Benzofuran-2-yl)-2-(4-methoxyphenyl)ethane-1,2-dione (5o) Prepared following general procedure using the corresponding α-ketothioesters (23.4 mg, 0.1 mmol), 4-methoxyphenyl borates (0.1 mmol, 1 equiv), $Pd_2(dba)_3$ (0.0025 mmol, 2.5 mol%), 4,4'-dimethoxy-2,2'-bipyridine (0.01 mmol, 10 mol%), CuTc (0.1 mmol, 1 equiv), K_2CO_3 (0.15 mmol, 1.5 equiv), anhydrous Na_2SO_4 (0.15 mmol, 1.5 equiv) and DMF (1 mL), the reaction was stirred at 45 ^oC for 12 h giving **5o** (13.9 mg) in 50% yield as a yellow solid by column chromatography. **^{1}H NMR** (400 MHz, $CDCl_3$) δ 8.10 – 8.03 (m, 2H), 7.73 (d, *J* = 7.9 Hz, 1H), 7.69 (d, *J* = 0.7 Hz, 1H), 7.63 (d, *J* = 8.5 Hz, 1H), 7.57 – 7.50 (m, 1H), 7.37 – 7.31 (m, 1H), 7.02 – 6.96 (m, 2H), 3.90 (s, 3H). **^{13}C NMR** (100 MHz, $CDCl_3$) δ 189.8, 183.0, 165.1, 156.5, 149.8, 132.8, 129.6, 126.8, 125.5, 124.3, 123.9, 119.4, 114.3, 112.7, 55.7. **MS (EI)** m/z 280 (M^+).

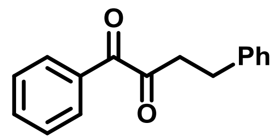

5p

1,4-diphenylbutane-1,2-dione (5p) Prepared following general procedure using α-ketothioesters **2p** (19.4 mg, 0.1 mmol), phenethylborates (0.1 mmol, 1 equiv), $Pd_2(dba)_3$ (0.0025 mmol, 2.5 mol%), 4,4'-dimethoxy-2,2'-bipyridine (0.01 mmol, 10 mol%), CuTc (0.1 mmol, 1 equiv), K_2CO_3 (0.15 mmol, 1.5 equiv), anhydrous Na_2SO_4 (0.15 mmol, 1.5 equiv) and DMF (1 mL), the reaction was stirred at 45 ^oC for 12 h giving **5p** (10.7 mg) in 45% yield as a colorless oil by column chromatography. **^{1}H NMR** (400 MHz, $CDCl_3$) δ 7.88 – 7.81 (m, 2H), 7.67 – 7.59 (m, 1H), 7.48 – 7.40 (m, 2H), 7.34 – 7.24 (m, 5H), 4.61 (t, *J* = 7.0 Hz, 2H), 3.08 (t, *J* = 7.0 Hz, 2H). **^{13}C NMR** (100 MHz, $CDCl_3$) δ 186.3, 163.7, 136.9, 134.9, 132.3, 130.0, 129.0, 128.8, 128.7, 126.9, 66.4, 34.9. **MS (EI)** m/z 238 (M^+).

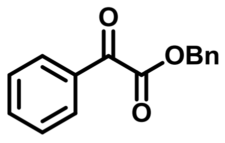

2aa

Benzyl 2-oxo-2-phenylacetate (2aa): α-Ketothioester **2a** (0.2 mmol), benzyl alcohol (0.2 mmol), DMAP (7.4 mg, 0.06 mmol, 30 mol%) and THF (2 mL) were added to a reaction tube. After stirring for 12 hours at 70 ^{o}C (detect by TLC), the solvent was removed and the residue was purified by column chromatography to give the corresponding product **2aa** (35.0 mg) in 73% yield. **^{1}H NMR** (400 MHz, $CDCl_3$) δ 8.01-7.93 (m, 2H), 7.65 (t, *J* = 7.4 Hz, 1H), 7.53-7.35 (m, 7H), 5.42 (s, 2H). **^{13}C NMR** (100 MHz, $CDCl_3$) δ 186.0, 163.6, 134.9, 134.5, 132.4, 130.0, 128.9, 128.8, 128.7, 128.6, 67.7. **MS** (EI) m/z 240 (M^+).

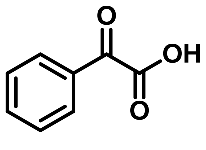

2ac

2-Oxo-2-phenylacetic acid (2ac): α-Ketothioester **2a** (0.2 mmol), 1M NaOH (1 mL) and THF (1 mL) were added to a reaction tube. After stirring for 1 hours at room temperature (detect by TLC), the mixture was extracted with DCM. The solvent was removed and the residue was purified by column chromatography to give the corresponding product **2ac** (28.2 mg) in 94% yield. **^{1}H NMR** (400 MHz, $CDCl_3$) δ 8.04 (d, *J* = 6.7 Hz, 2H), 7.85 (brs, 1H), 7.52 (t, *J* = 7.2 Hz, 1H), 7.34 (t, *J* = 7.1 Hz, 2H). **^{13}C NMR** (100 MHz, $CDCl_3$) δ 187.9, 165.3, 135.0, 132.1, 130.8, 128.7, 77.0.

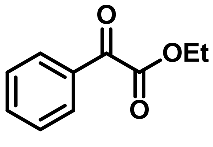

2ad

Ethyl 2-oxo-2-phenylacetate (2ad): α-Ketothioester **2a** (0.2 mmol), EtOH (1 mmol), DMAP (7.4 mg, 0.06 mmol, 30 mol%) and THF (2 mL) were added to a reaction tube. After stirring for 12 hours at 70 ^{o}C (detect by TLC), the solvent was removed and the residue was purified by column chromatography to give the corresponding product **2ad** (29.9 mg) in 84% yield. **^{1}H NMR** (400 MHz, $CDCl_3$) δ 7.99 (d, *J* = 7.3 Hz, 2H), 7.64 (t, *J* = 7.4 Hz, 1H), 7.50 (t, *J* = 7.7

Hz, 2H), 4.44 (q, J = 7.1 Hz, 2H), 1.41 (t, J = 7.2 Hz, 3H). ^{13}**C NMR** (100 MHz, $CDCl_3$) δ 186.4, 163.8, 134.8, 132.4, 129.9, 128.8, 62.2, 15.3.

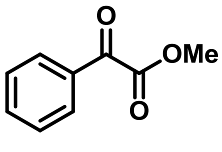

2ae

Methyl 2-oxo-2-phenylacetate (2ae): α-Ketothioester **2a** (0.2 mmol), MeOH (1 mmol), DMAP (7.4 mg, 0.06 mmol, 30 mol%) and THF (2 mL) were added to a reaction tube. After stirring for 12 hours at 60 ^{o}C (detect by TLC), the solvent was removed and the residue was purified by column chromatography to give the corresponding product **2ae** (28.6 mg) in 87% yield. ^{1}**H NMR** (400 MHz, $CDCl_3$) δ 8.00 (dd, J = 8.4, 1.2 Hz, 2H), 7.64 (t, J = 7.4 Hz, 1H), 7.49 (t, J = 7.8 Hz, 2H), 3.96 (s, 3H). ^{13}**C NMR** (100 MHz, $CDCl_3$) δ 186.0, 164.0, 134.9, 132.4, 130.0, 128.8, 52.7.

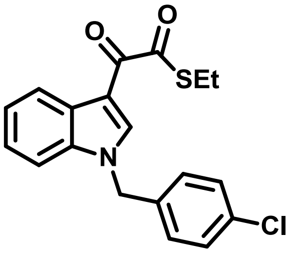

6a

***S*-ethyl 2-(1-(4-chlorobenzyl)-1H-indol-3-yl)-2-oxoethanethioate (6a)**: Prepared following general procedure using **1aa** (37.5 mg, 0.125 mmol), S_8 (23.0 mg, 0.72 mmol), Cs_2CO_3 (195 mg, 0.6 mmol, 4.8 equiv), TBAB (8.0 mg, 0.025 mmol), DMF (1 mL) and EtBr (28.0 mg, 0.25 mmol, 2 equiv), the reaction was stirred at 90 ^{o}C for 12 hours giving **6a** (30.5 mg) in 68% yield as a yellow solid by column chromatography. ^{1}**H NMR** (400 MHz, $CDCl_3$) δ 8.47 (s, 1H), 8.39 (d, J = 7.8 Hz, 1H), 7.32 – 7.16 (m, 5H), 7.03 (d, J = 8.3 Hz, 2H), 5.27 (s, 2H), 2.91 (q, J = 7.4 Hz, 2H), 1.28 (t, J = 7.4 Hz, 3H). ^{13}**C NMR** (100 MHz, $CDCl_3$) δ 194.5, 178.2, 139.7, 136.6, 134.3 133.7, 129.3, 128.3, 127.6, 124.4, 123.7, 123.0, 110.7, 110.5, 50.5, 23.1, 14.2. **HRMS (EI)** Calcd for $C_{19}H_{16}NO_2SCl$ 357.0590, Found 357.0592.

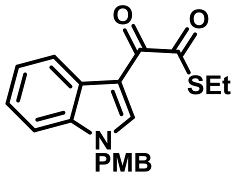

6b

***S*-Ethyl 2-(1-(4-methoxybenzyl)-1H-indol-3-yl)-2-oxoethanethioate (6b)**: Prepared following general procedure using **1ab** (36.9 mg, 0.125 mmol), S_8 (23.0 mg, 0.72 mmol), Cs_2CO_3 (195 mg, 0.6 mmol, 4.8 equiv), TBAB (8.0 mg, 0.025 mmol), DMF (1 mL) and EtBr (28.0 mg, 0.25 mmol, 2 equiv), the reaction was stirred at 90 ^{o}C for 12 hours giving **6b** (32.6 mg) in 74% yield as a yellow solid by column chromatography. **^{1}H NMR** (400 MHz, $CDCl_3$) δ 8.52 (s, 1H), 8.47 – 8.43 (m, 1H), 7.37 – 7.28 (m, 3H), 7.17 – 7.11 (m, 2H), 6.89 – 6.84 (m, 2H), 5.30 (s, 2H), 3.78 (s, 3H), 2.97 (q, *J* = 7.4 Hz, 2H), 1.34 (t, *J* = 7.5 Hz, 3H). **^{13}C NMR** (100 MHz, $CDCl_3$) δ 194.7, 178.2, 159.6, 139.8, 136.8, 128.5, 127.8, 127.1 124.2, 123.6, 122.9, 114.5, 110.6, 110.5, 55.3, 50.8, 23.1, 14.2. **HRMS (EI)** Calcd for $C_{20}H_{19}NO_3S$ 353.1086, Found 353.1090.

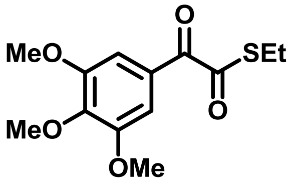

6c

***S*-ethyl 2-oxo-2-(3,4,5-trimethoxyphenyl)ethane thioate (6c)**: Prepared following general procedure using **1ac** (56.6 mg, 0.25 mmol), S_8 (32.0 mg, 1.0 mmol), $KHCO_3$ (50 mg, 0.5 mmol), TBAC (14.0 mg, 0.05 mmol), DMF (2 mL) and EtBr (54.5 mg, 0.5 mmol, 2 equiv), the reaction was stirred at 90 ^{o}C for 12 hours giving **6c** (46.3 mg) in 65% yield as a yellow solid by column chromatography. **^{1}H NMR** (400 MHz, $CDCl_3$) δ 7.45 (s, 2H), 3.95 (s, 3H), 3.90 (s, 6H), 3.08 – 3.00 (m, 2H), 1.39 – 1.33 (m, 3H). **^{13}C NMR** (100 MHz, $CDCl_3$) δ 193.3, 184.7, 153.1, 144.5, 126.4, 108.3, 61.0, 56.3, 23.4, 14.2. **HRMS (EI)** Calcd for $C_{13}H_{16}O_5S$ 284.0718, Found 284.0722.

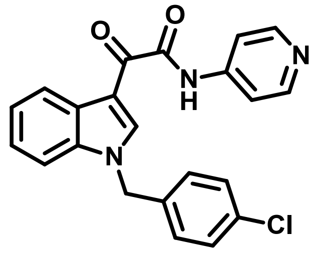

7a, Indibulin

Indibulin (7a): Under a N_2 atmosphere, α-ketothioester **6a** (17.9 mg, 0.05 mmol), pyridin-4-amine (7.1 mg, 0.075 mmol), $Pd(OAc)_2$ (0.6 mg, 0.0025mmol, 5 mol%), IPr·HCl (2.1 mg, 0.005 mmol, 10 mol%), K_2CO_3 (10.4 mg, 0.075 mmol) and toluene (0.3 mL) were added to a Schlenk tube. After stirring for 12 hours at 110 ^oC (detect by TLC), the mixture was cooled to room temperature and water (5 mL) was added. The solution was extracted with ethyl acetate and organic layers were combined, dried over sodium sulfate. After evaporation of solvent, the residue was purified by column chromatography to give the corresponding product **7a** (12.1 mg) in 62% yield as a yellow solid. **^{1}H NMR** (400 MHz, d_6-DMSO) δ 11.10 (s, 1H), 9.00 (s, 1H), 8.52 (d, *J* = 4.6 Hz, 2H), 8.30 (dd, *J* = 6.2, 2.1 Hz, 1H), 7.87 (d, *J* = 4.6 Hz, 2H), 7.60 (dd, *J* = 6.3, 2.3 Hz, 1H), 7.41 (d, *J* = 8.5 Hz, 2H), 7.37 – 7.26 (m, 4H), 5.62 (s, 2H). **^{13}C NMR** (100 MHz, d_6-DMSO) δ 180.7, 163.0, 150.4, 144.9, 141.4, 136.3, 135.6, 132.5, 129.3, 128.8, 126.9, 124.0, 123.4, 121.6, 114.3, 111.7, 111.2, 49.2. **MS (EI)** m/z 389 (M^+).

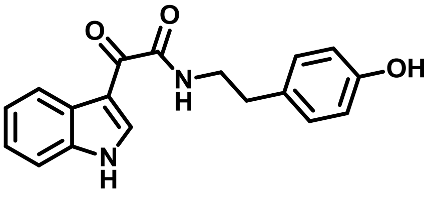

7b, Polyandrocarpamide C

Polyandrocarpamide C (7b): Prepared following general procedure using α-ketothioester **6b** (35.3 mg, 0.1 mmol), tyramine (13.7 mg, 0.1 mmol) and THF (1 mL), the reaction was stirred at room temperature for 12 h. After evaporation of solvent, the residue was added to the mixture of anisole (0.18 mL) in TFA (1.8 mL) at 0 ^oC. The reaction was stirred at 80 ^oC for 19 hours giving **7b** (23.1 mg) in 75% yield as a white solid by column chromatography. **^{1}H NMR** (400 MHz, d_6-acetone) δ 11.25 (s, 1H), 9.04 (d, *J* = 3.2 Hz, 1H), 8.38 – 8.31 (m, 1H), 8.14 (s, 1H), 8.05 (s, 1H), 7.59 – 7.53 (m, 1H), 7.30 – 7.23 (m, 2H), 7.12 (d, *J* = 8.4

Hz, 2H), 6.81 – 6.75 (m, 2H), 3.59 – 3.52 (m, 2H), 2.83 (t, *J* = 7.2 Hz, 2H). **^{13}C NMR** (100 MHz, *d*$_6$-acetone) δ 182.3, 163.6, 156.8, 139.5, 137.3, 130.9, 130.6, 127.8, 124.4, 123.4, 122.8, 116.2, 113.8, 113.1, 41.6, 35.4. **HRMS (EI)** Calcd for $C_{18}H_{16}N_2O_3$ 308.1161, Found 308.1164.

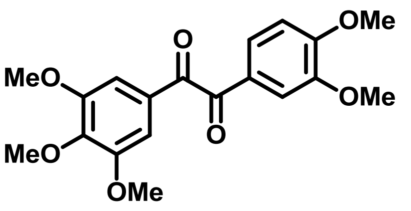

7c

1-(3,4-dimethoxyphenyl)-2-(3,4,5-trimethoxyphenyl)ethane-1,2-dione (7c) Prepared following general procedure using α-ketothioesters **6c** (28.4 mg, 0.1 mmol), 3,4-dimethoxyphenyl borates (0.1 mmol, 1 equiv), $Pd_2(dba)_3$ (0.0025 mmol, 2.5 mol%), 4,4'-dimethoxy-2,2'-bipyridine (0.01 mmol, 10 mol%), CuTc (0.1 mmol, 1 equiv), K_2CO_3 (0.15 mmol, 1.5 equiv), anhydrous Na_2SO_4 (0.15 mmol, 1.5 equiv) and DMF (1 mL), the reaction was stirred at 45 ^{o}C for 12 h giving **7c** (25.2 mg) in 70% yield as a yellow solid by column chromatography. **^{1}H NMR** (400 MHz, $CDCl_3$) δ 7.60 (d, *J* = 1.8 Hz, 1H), 7.48 (dd, *J* = 8.4, 1.9 Hz, 1H), 7.22 (s, 2H), 6.90 (d, *J* = 8.4 Hz, 1H), 3.99 – 3.92 (m, 9H), 3.88 (s, 6H). **^{13}C NMR** (100 MHz, $CDCl_3$) δ 193.5, 193.0, 155.0, 153.4, 149.6, 144.2, 128.2, 126.4, 126.3, 110.4, 107.2, 61.0, 56.4, 56.2, 56.1. **MS (EI)** m/z 360 (M^+).

X-Ray Crystal Structures

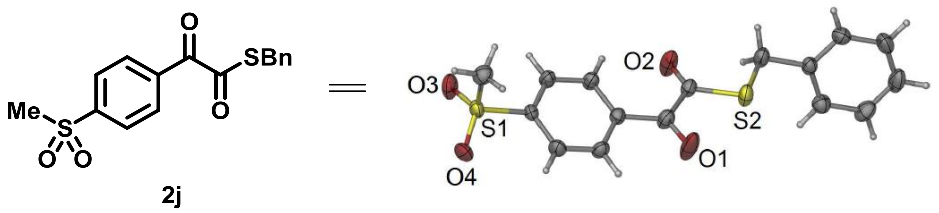

2j

Compound **2j** (CCDC 1895971).

Bond precision	C-C = 0.0032 A
	Wavelength = 1.54184
Cell	a = 5.5727 (3) α = 76.112
	b = 8.9629(4) β = 86.913(4)
	c = 16.3171(7) γ = 76.178(4)
Temperature	293 K
Volume	768.26(7)
Space group	P -1
Sum formula	C16 H14 O4 S2
Mr	334.39
Dx,g cm^{-3}	1.446
Z	2
Mu (mm-1)	3.281
F000	348.0
h,k,lmax	6, 10, 19
Nref	2717
Tmin,Tmax	0.147, 1.000
Correction method= # Reported T Limits	Tmin = 0.147 Tmax = 1.000
AbsCorr = MULTI-SCAN	
Data completeness	0.989
Theta(max)	67.040
R(reflections)	0.0548(2547)
wR2(reflections)	0.1443 (2717)
S	1.053
Npar	201

Supplementary Figure 1. Single-Crystal X-ray Crystallography of **2j**

NMR Spectra

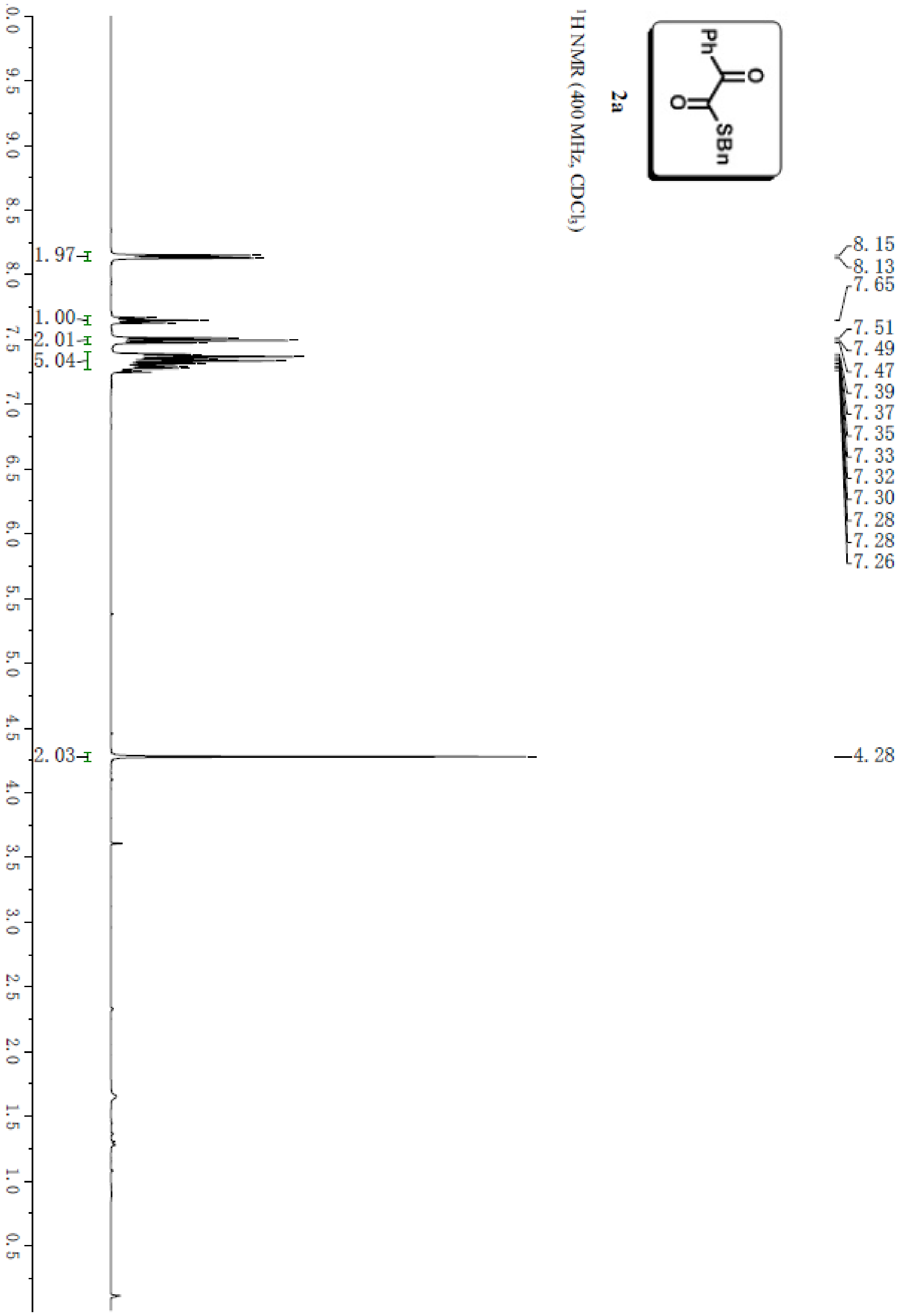

Supplementary Figure 2. ^{1}H NMR (400 MHz, $CDCl_3$) spectra of compound **2a**.

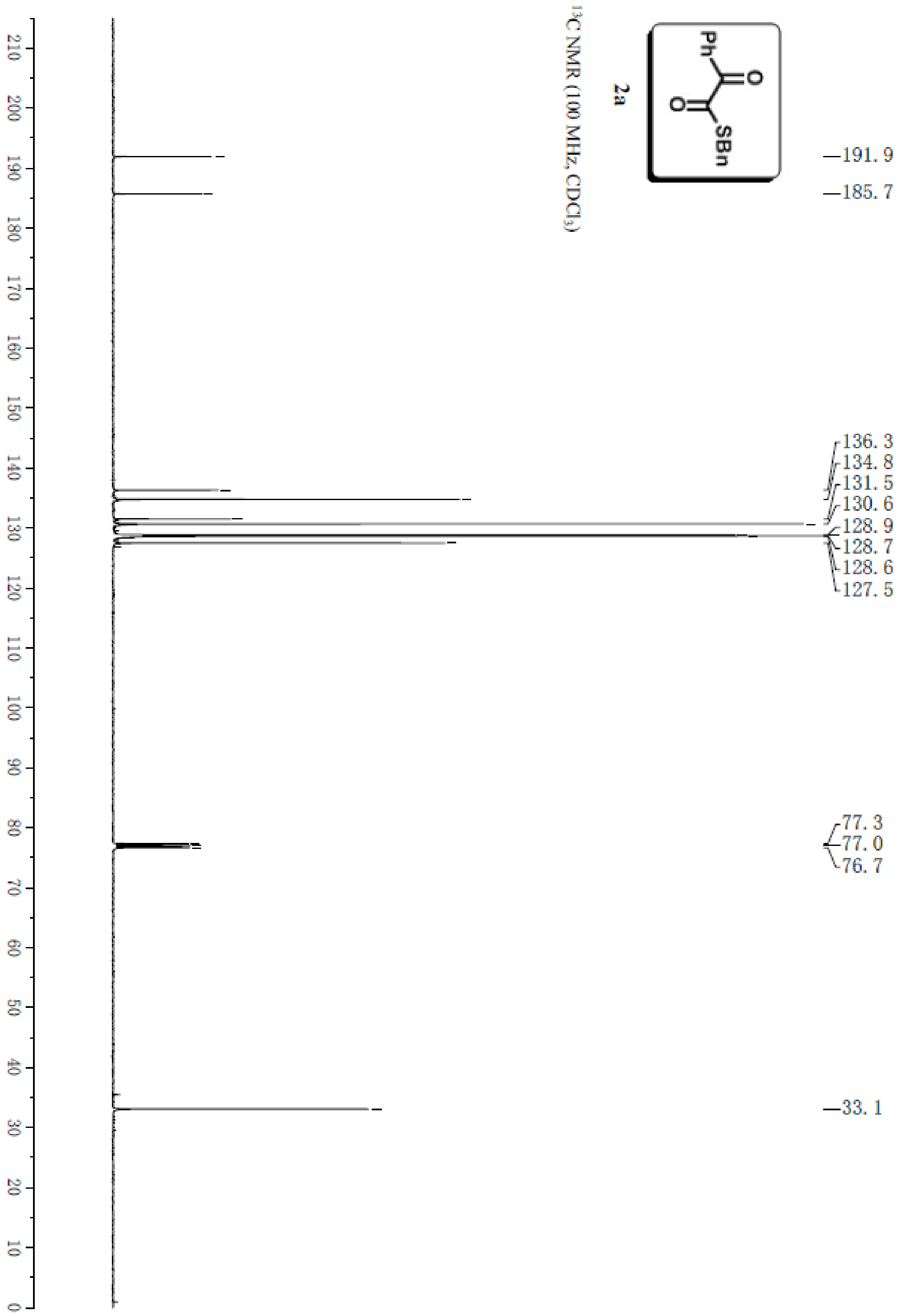

Supplementary Figure 3. ^{13}C NMR (100 MHz, $CDCl_3$) spectra of compound **2a**.

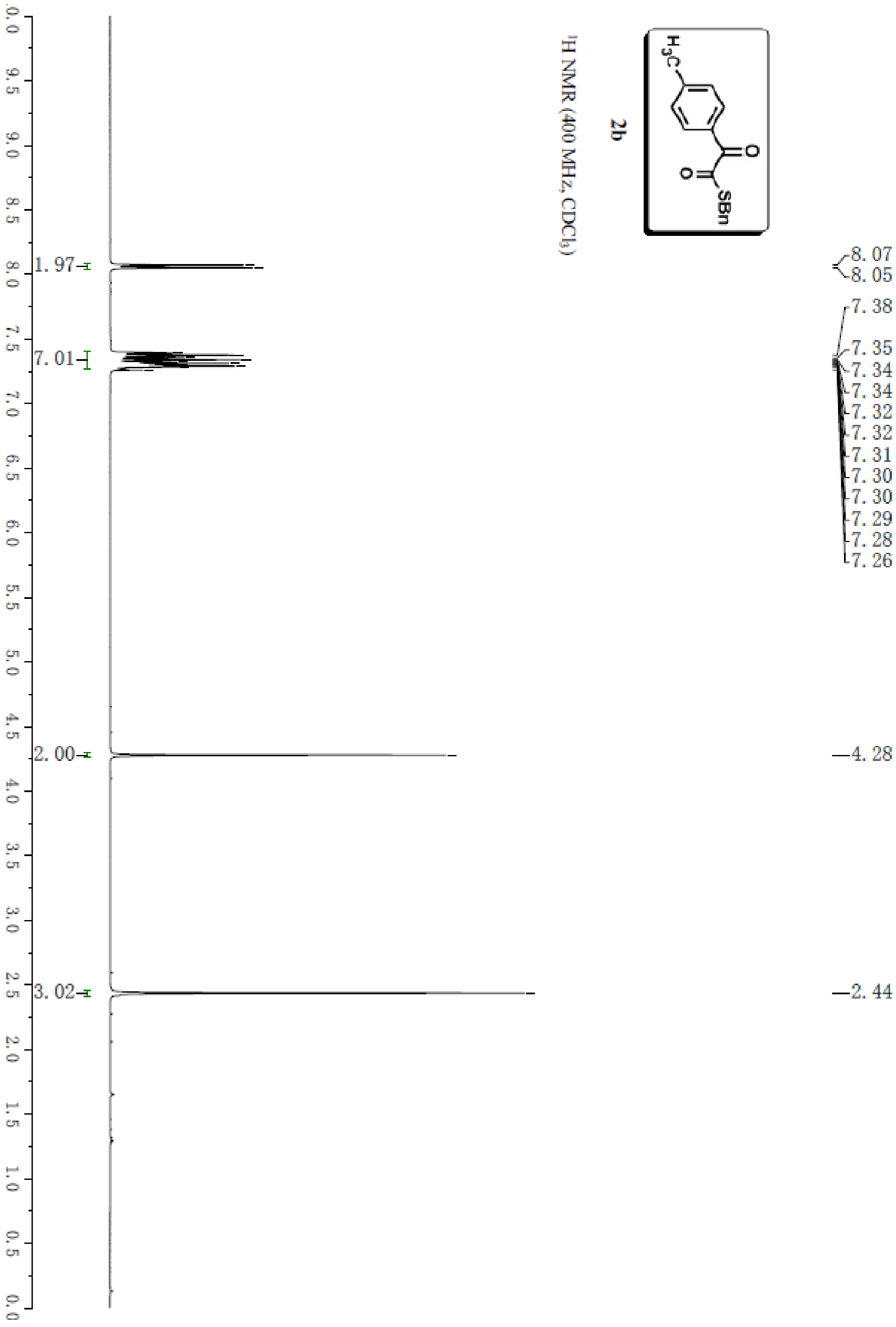

Supplementary Figure 4. ^{1}H NMR (400 MHz, $CDCl_3$) spectra of compound **2b**.

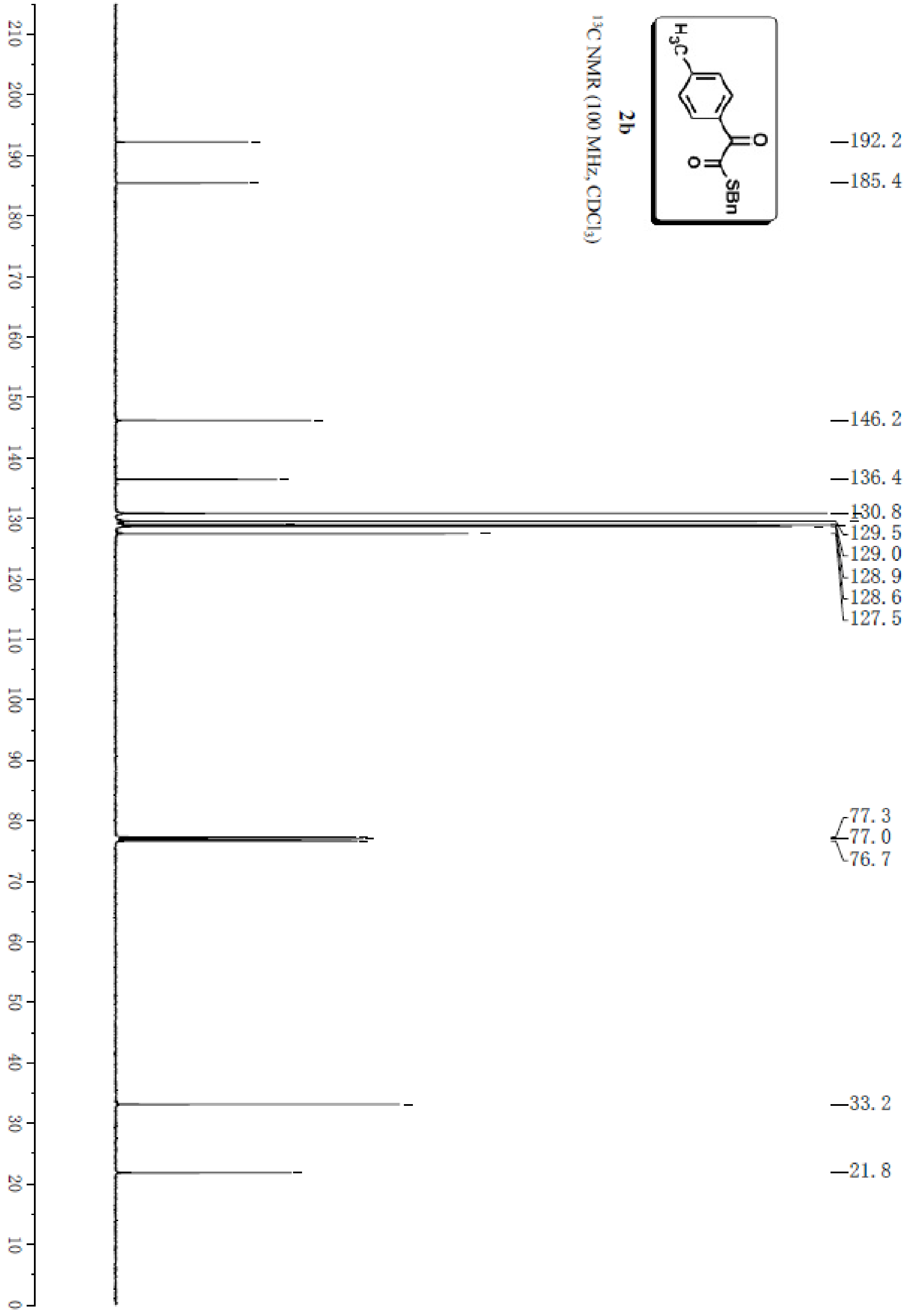

Supplementary Figure 5. ^{13}C NMR (100 MHz, $CDCl_3$) spectra of compound **2b**.

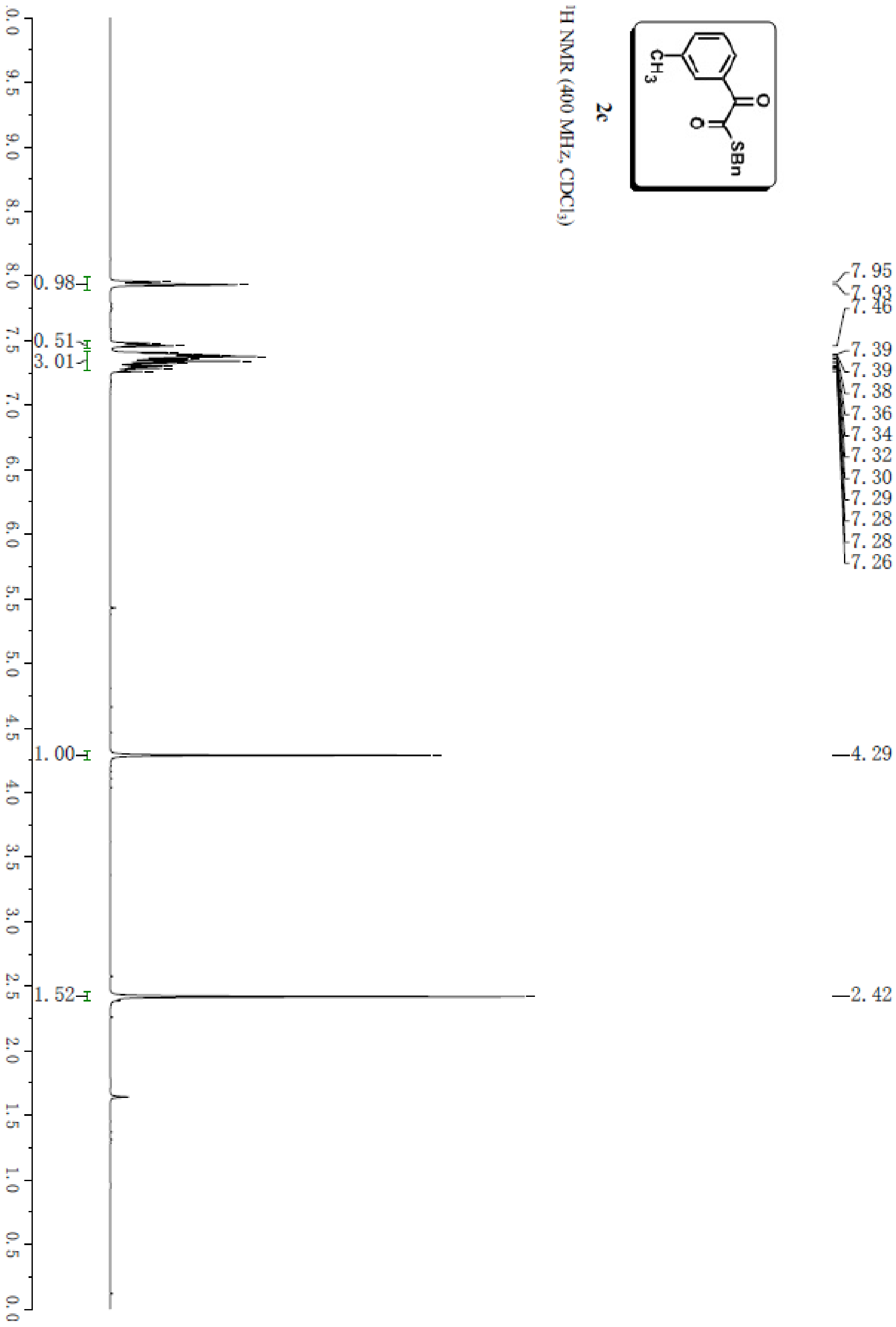

Supplementary Figure 6. ^{1}H NMR (400 MHz, $CDCl_3$) spectra of compound **2c**.

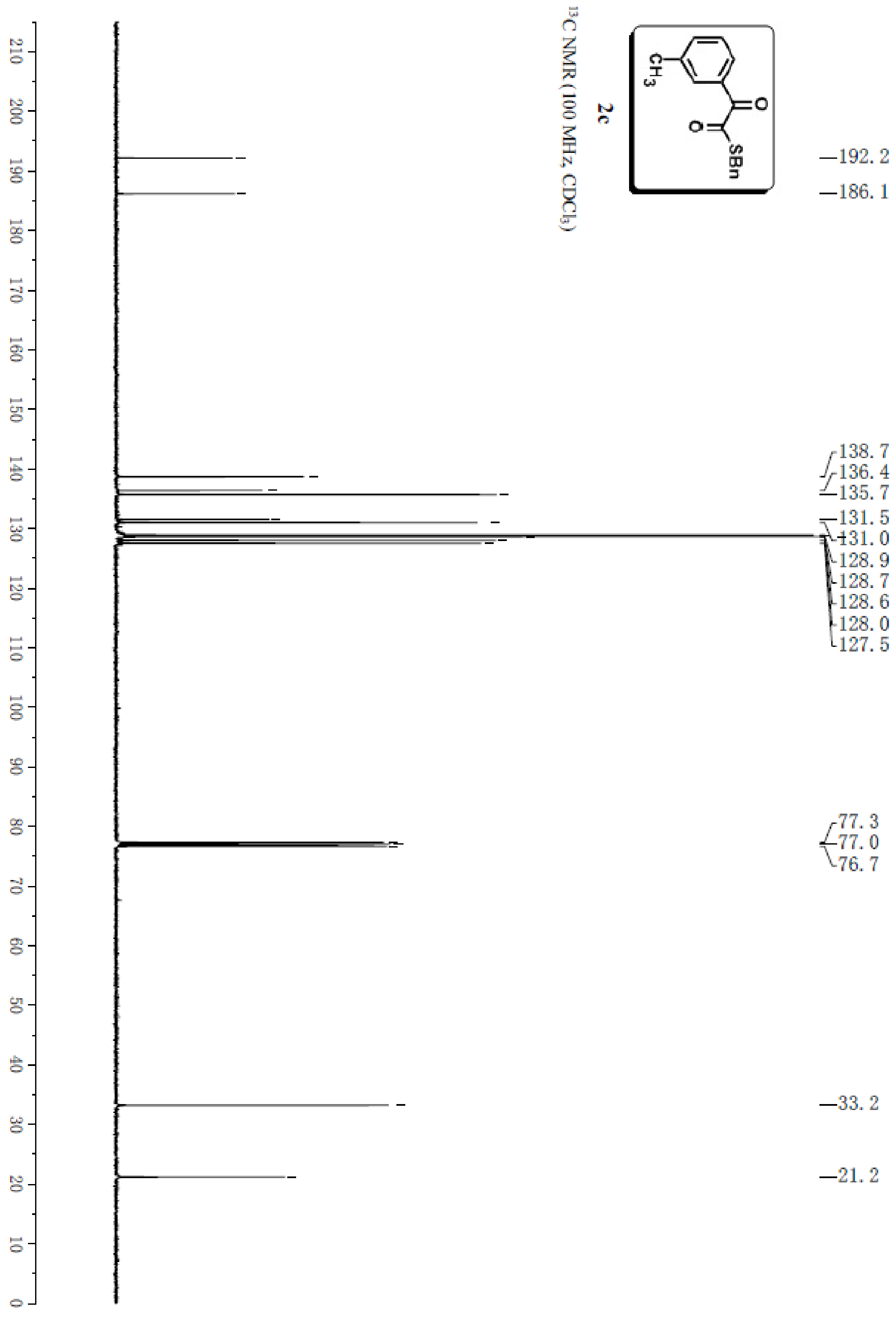

Supplementary Figure 7. ^{13}C NMR (100 MHz, $CDCl_3$) spectra of compound **2c**.

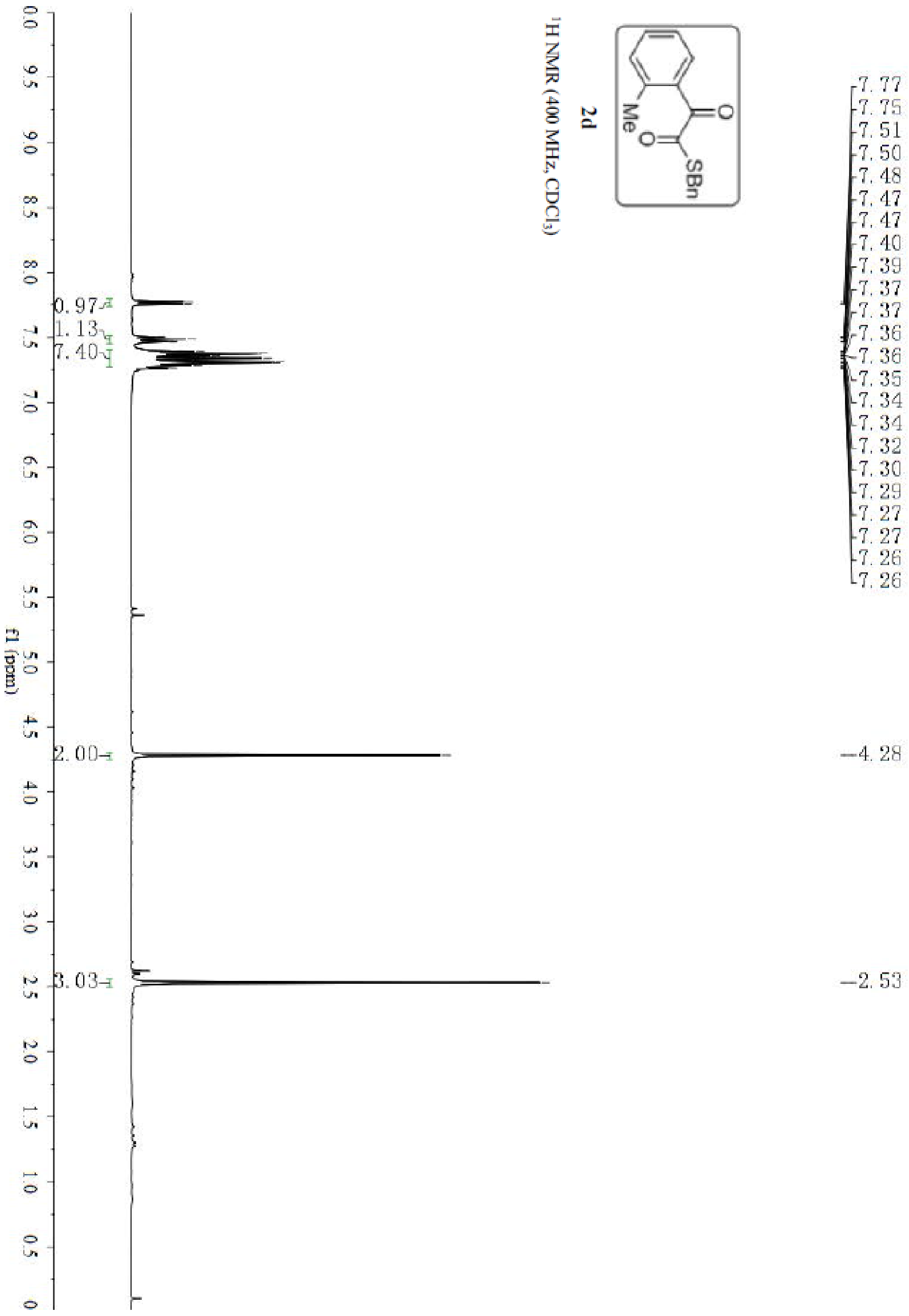

Supplementary Figure 8. ^{1}H NMR (400 MHz, $CDCl_3$) spectra of compound **2d**.

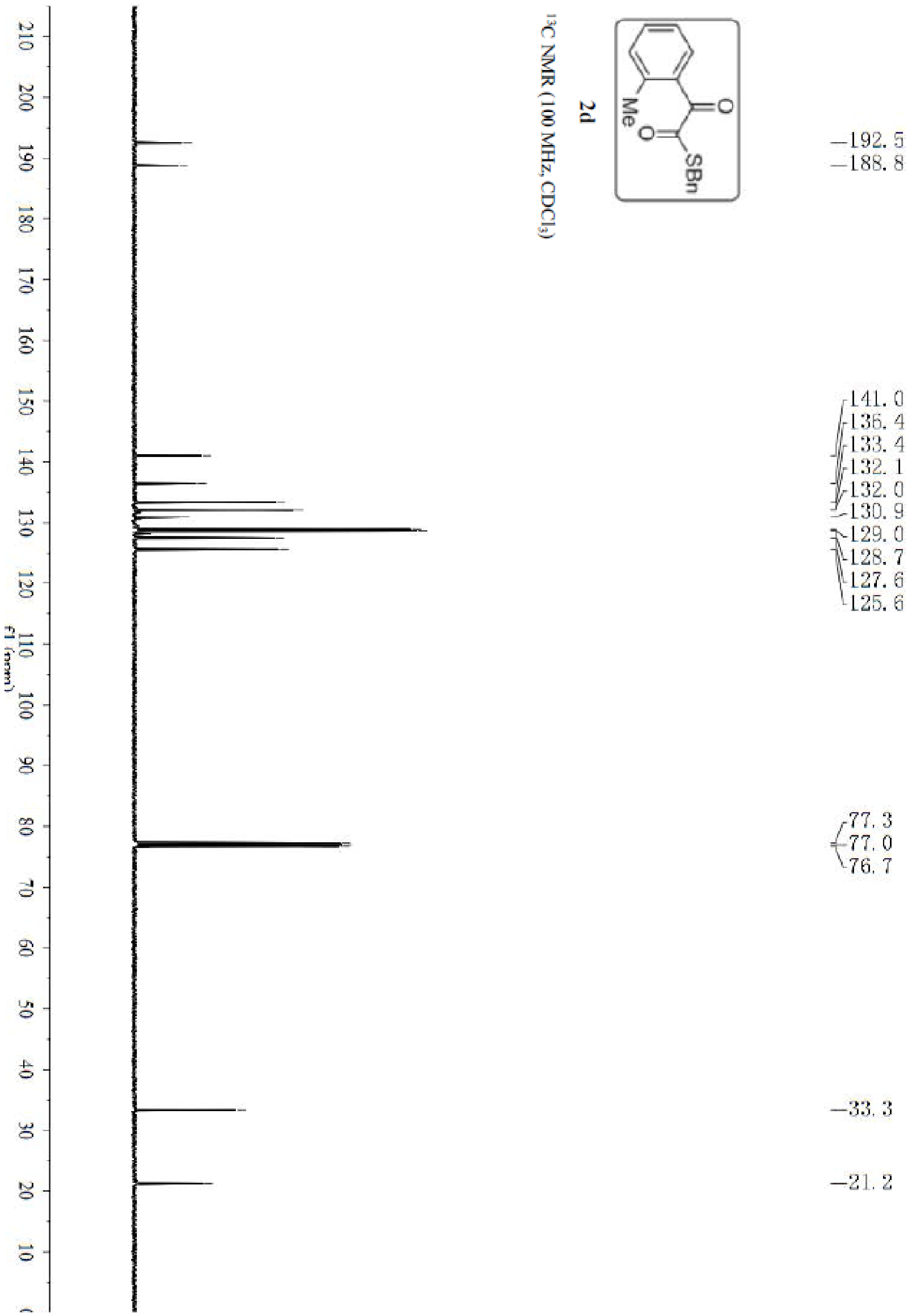

Supplementary Figure 9. ^{13}C NMR (100 MHz, $CDCl_3$) spectra of compound **2d**.

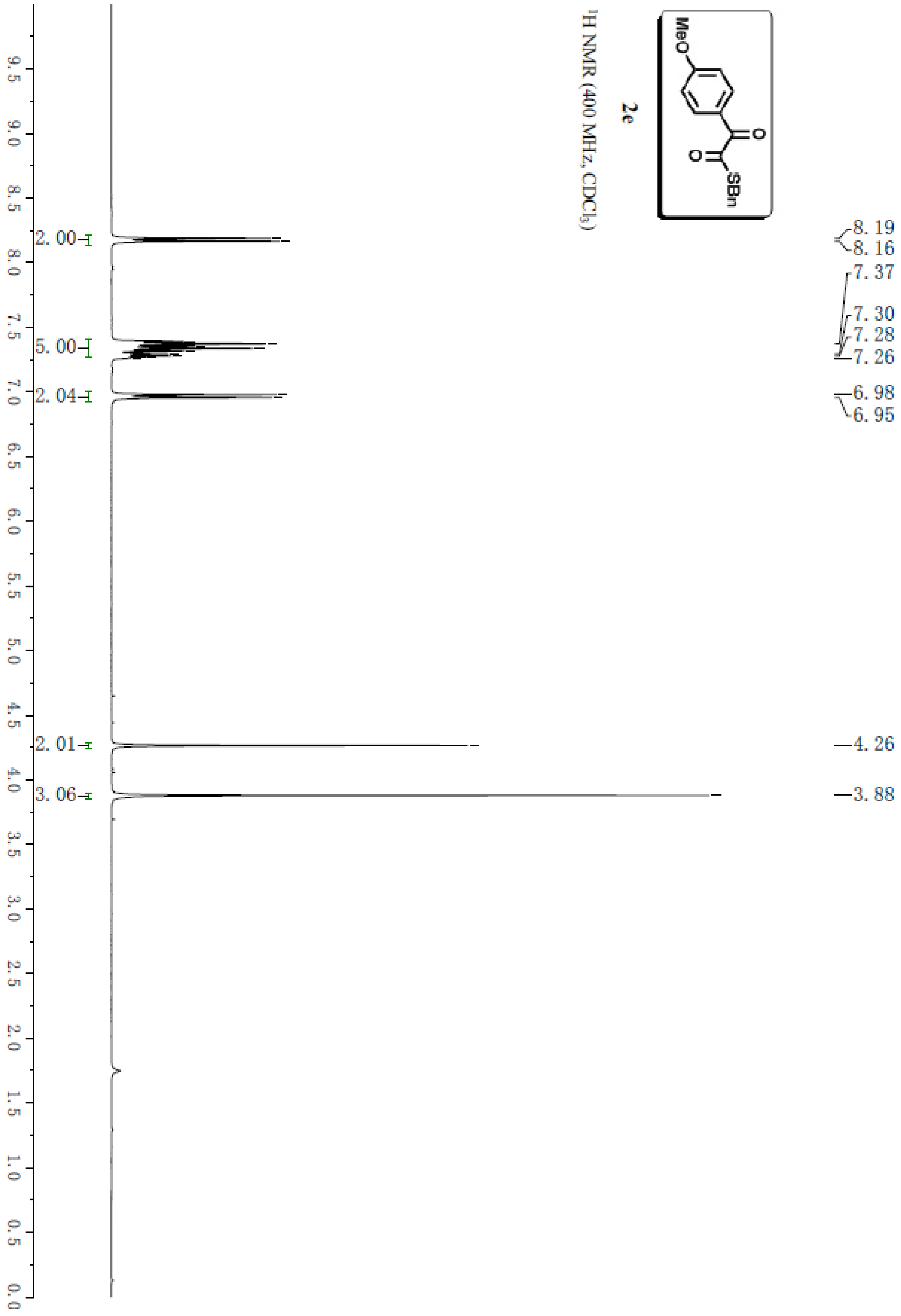

Supplementary Figure 10. ^{1}H NMR (400 MHz, $CDCl_3$) spectra of compound **2e**.

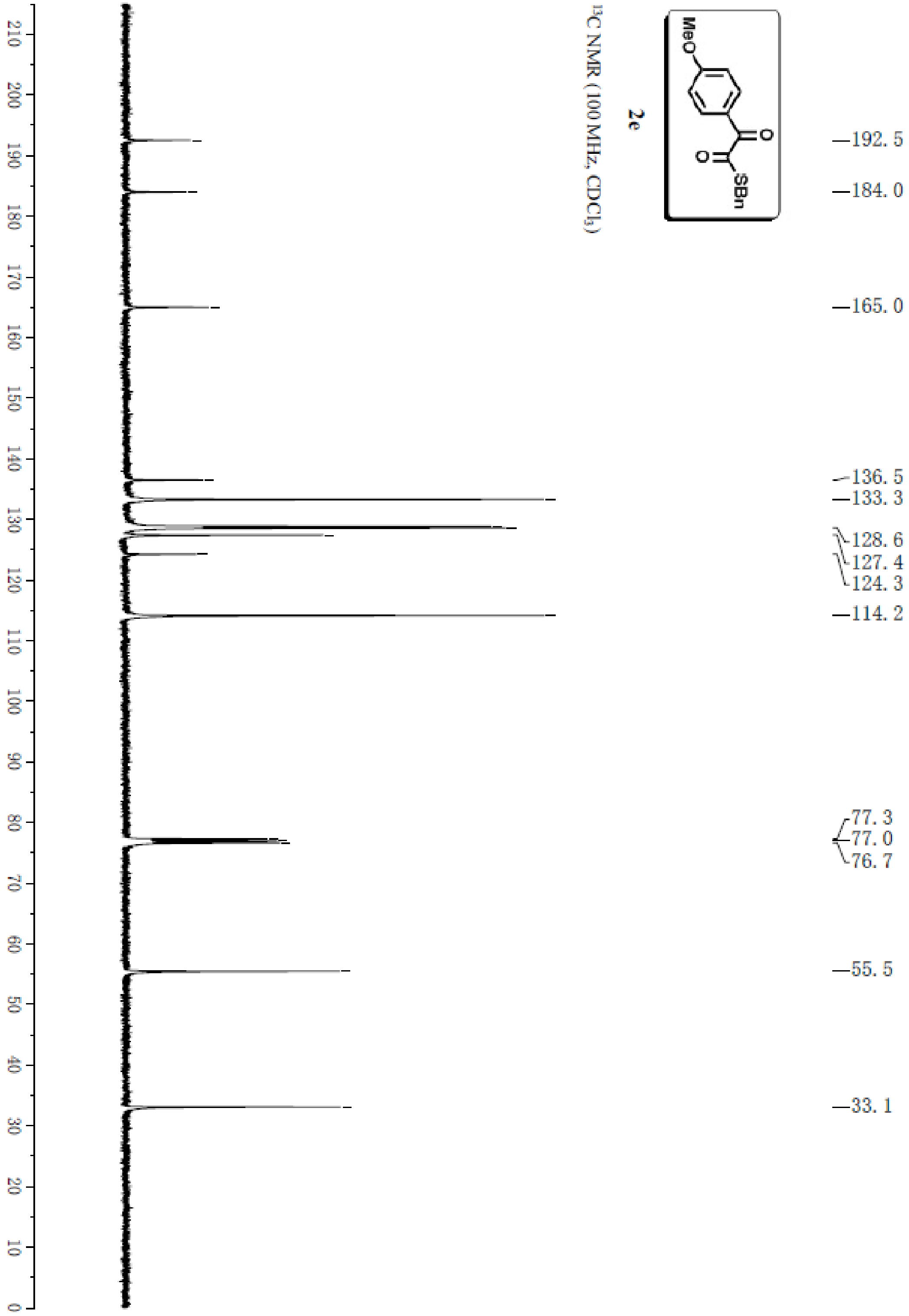

Supplementary Figure 11. ^{13}C NMR (100 MHz, $CDCl_3$) spectra of compound **2e**.

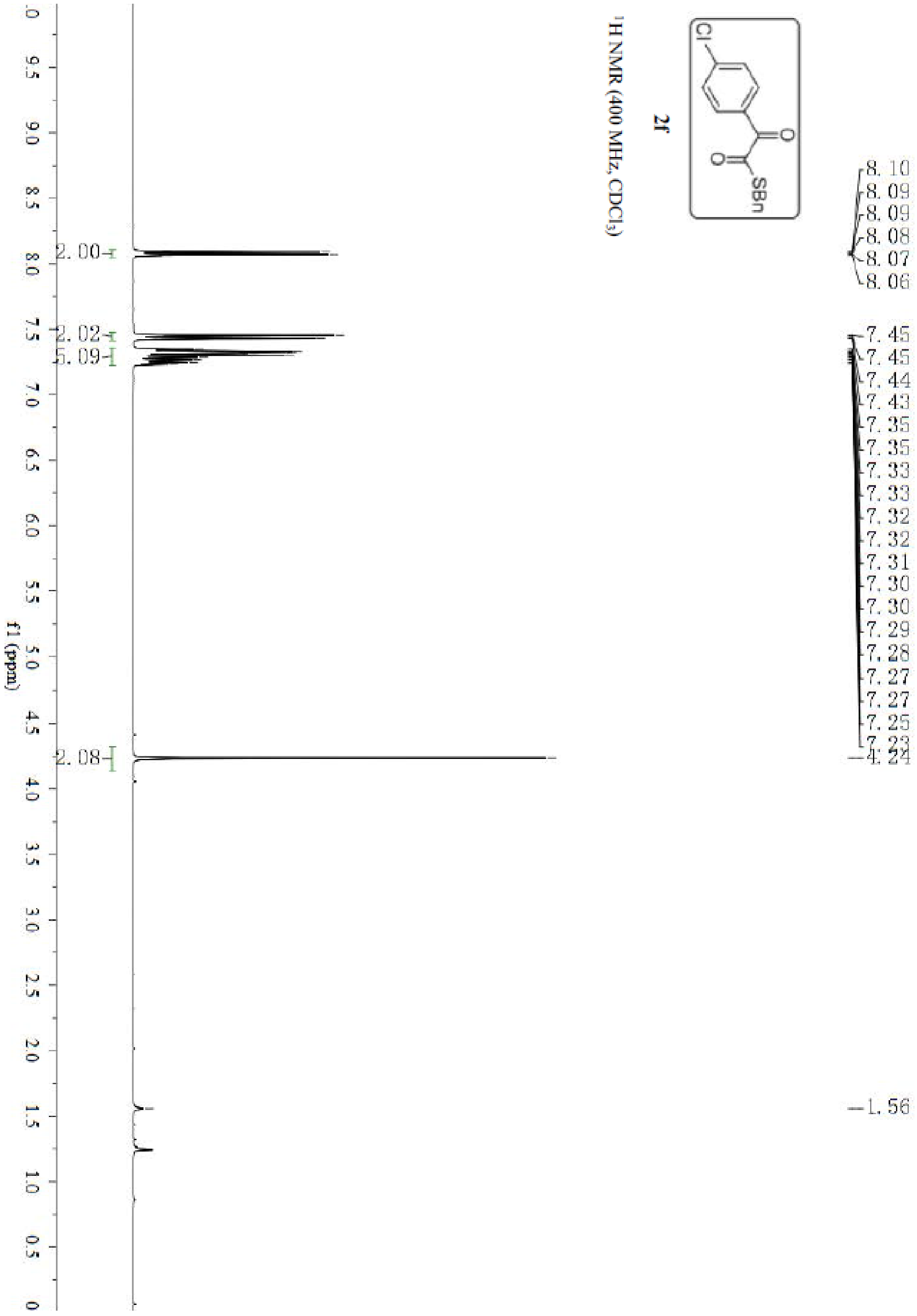

Supplementary Figure 12. ^{1}H NMR (400 MHz, $CDCl_3$) spectra of compound **2f**.

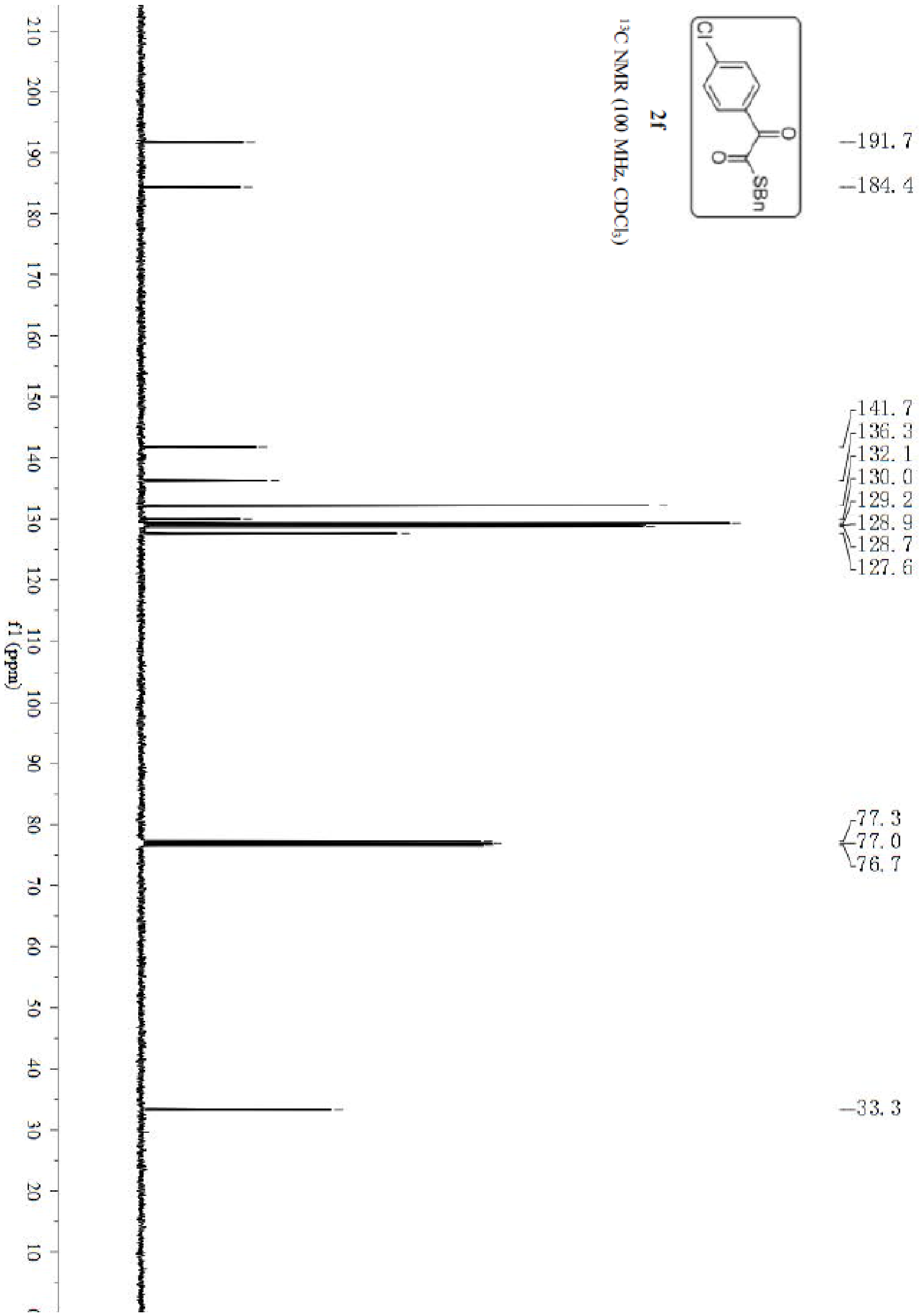

Supplementary Figure 13. ^{13}C NMR (100 MHz, $CDCl_3$) spectra of compound **2f**.

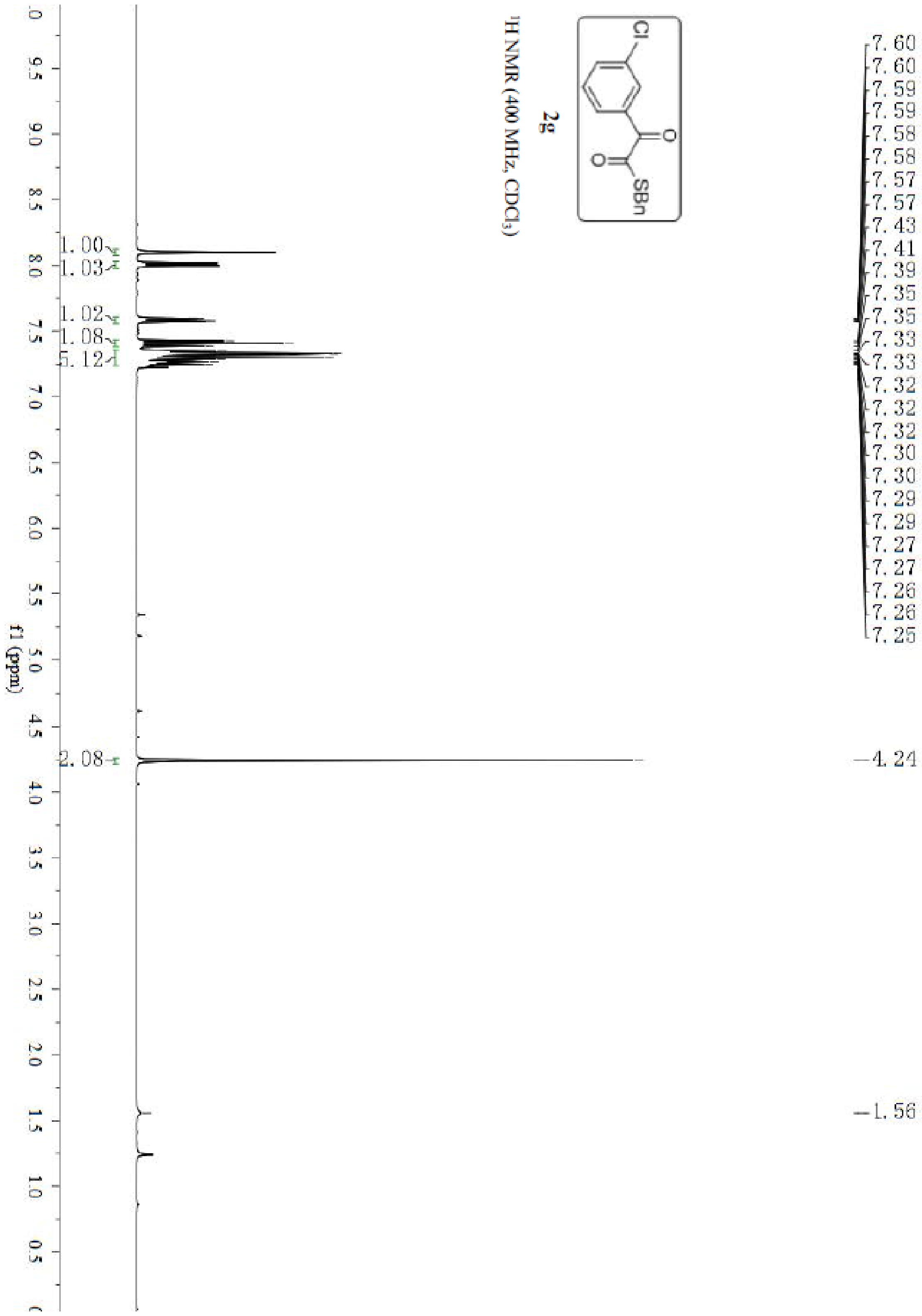

Supplementary Figure 14. ^{1}H NMR (400 MHz, $CDCl_3$) spectra of compound **2g**.

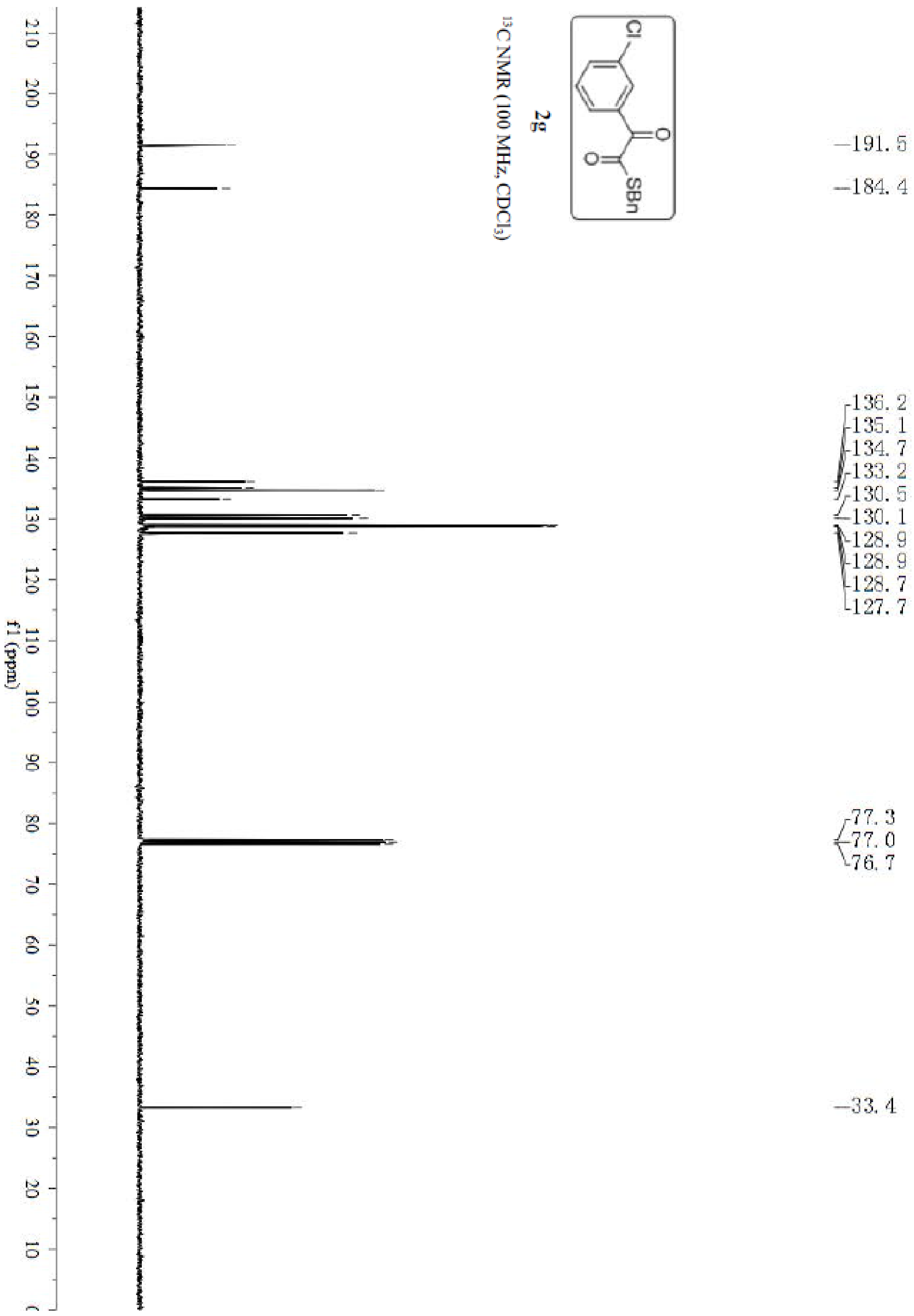

Supplementary Figure 15. ^{13}C NMR (100 MHz, $CDCl_3$) spectra of compound **2g**.

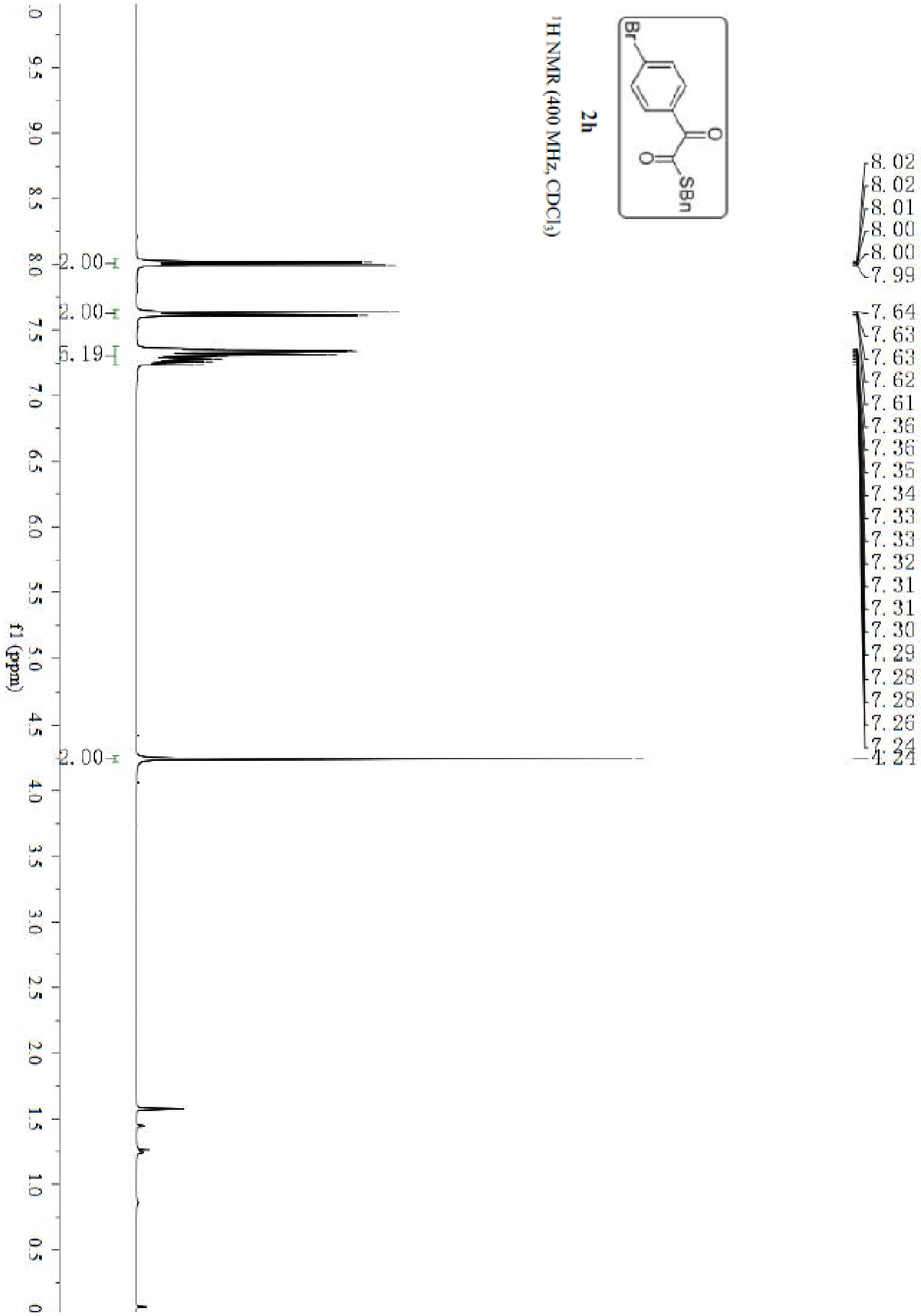

Supplementary Figure 16. ^{1}H NMR (400 MHz, $CDCl_3$) spectra of compound **2h**.

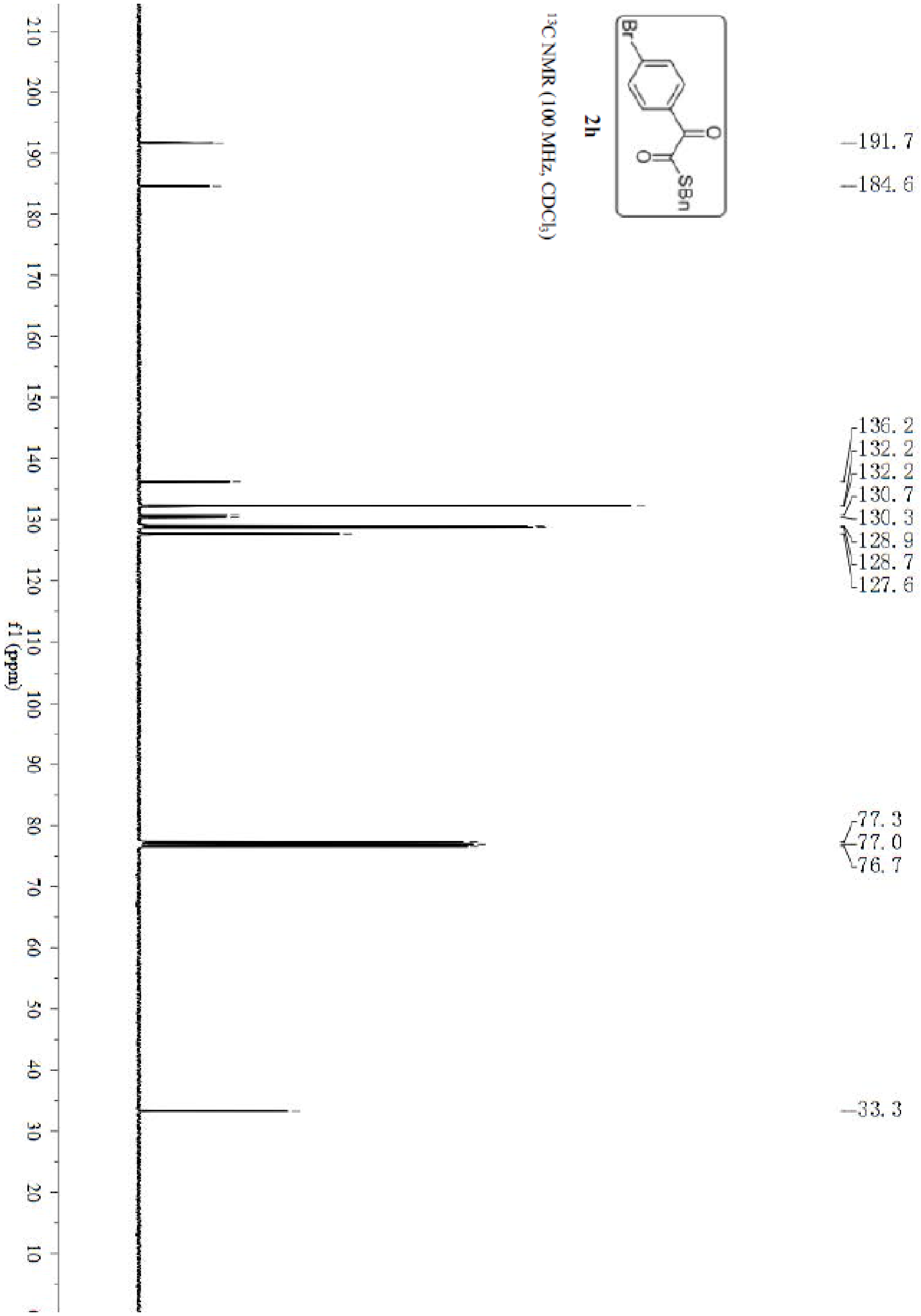

Supplementary Figure 17. ^{13}C NMR (100 MHz, $CDCl_3$) spectra of compound **2h**.

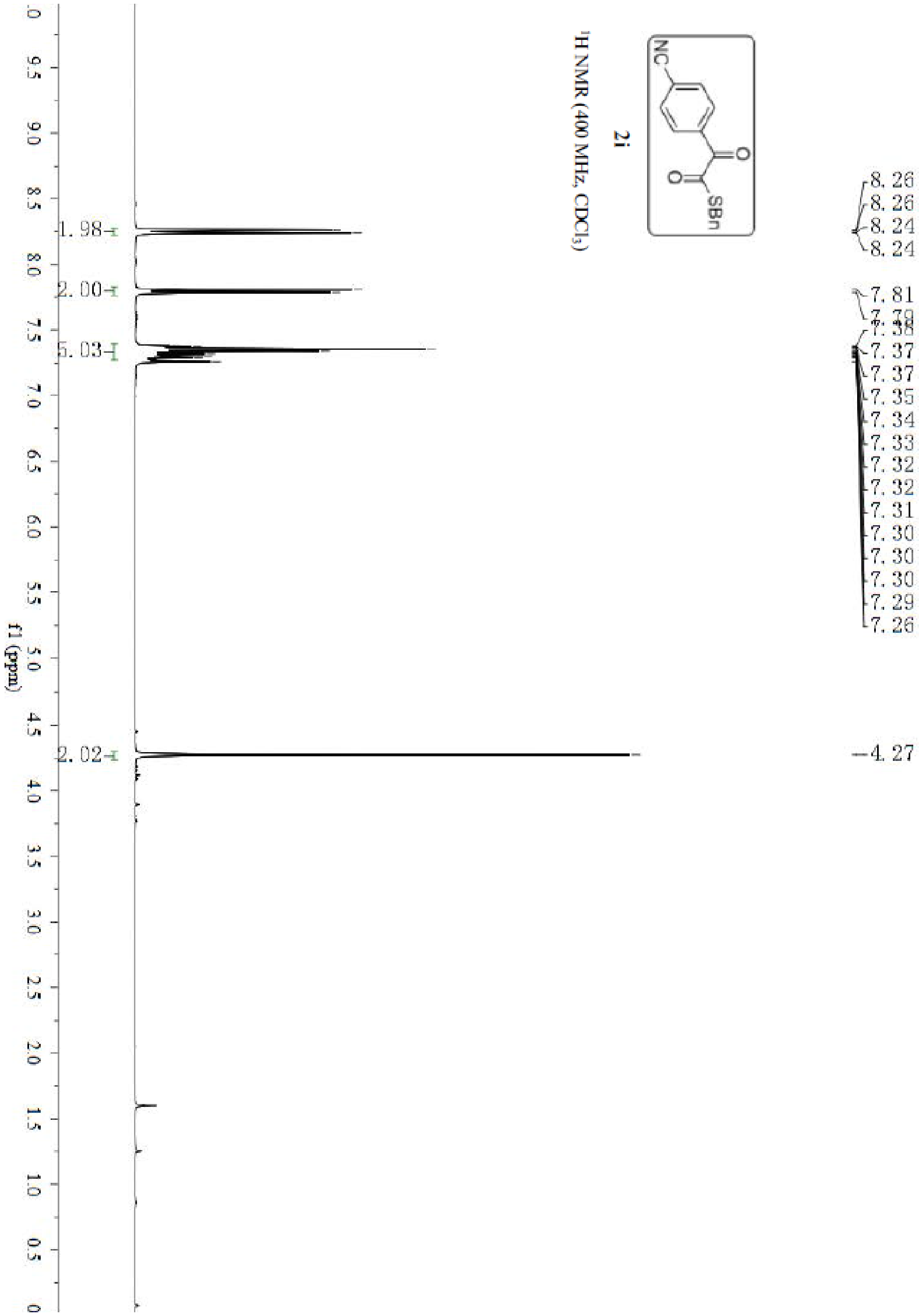

Supplementary Figure 18. ^{1}H NMR (400 MHz, $CDCl_3$) spectra of compound **2i**.

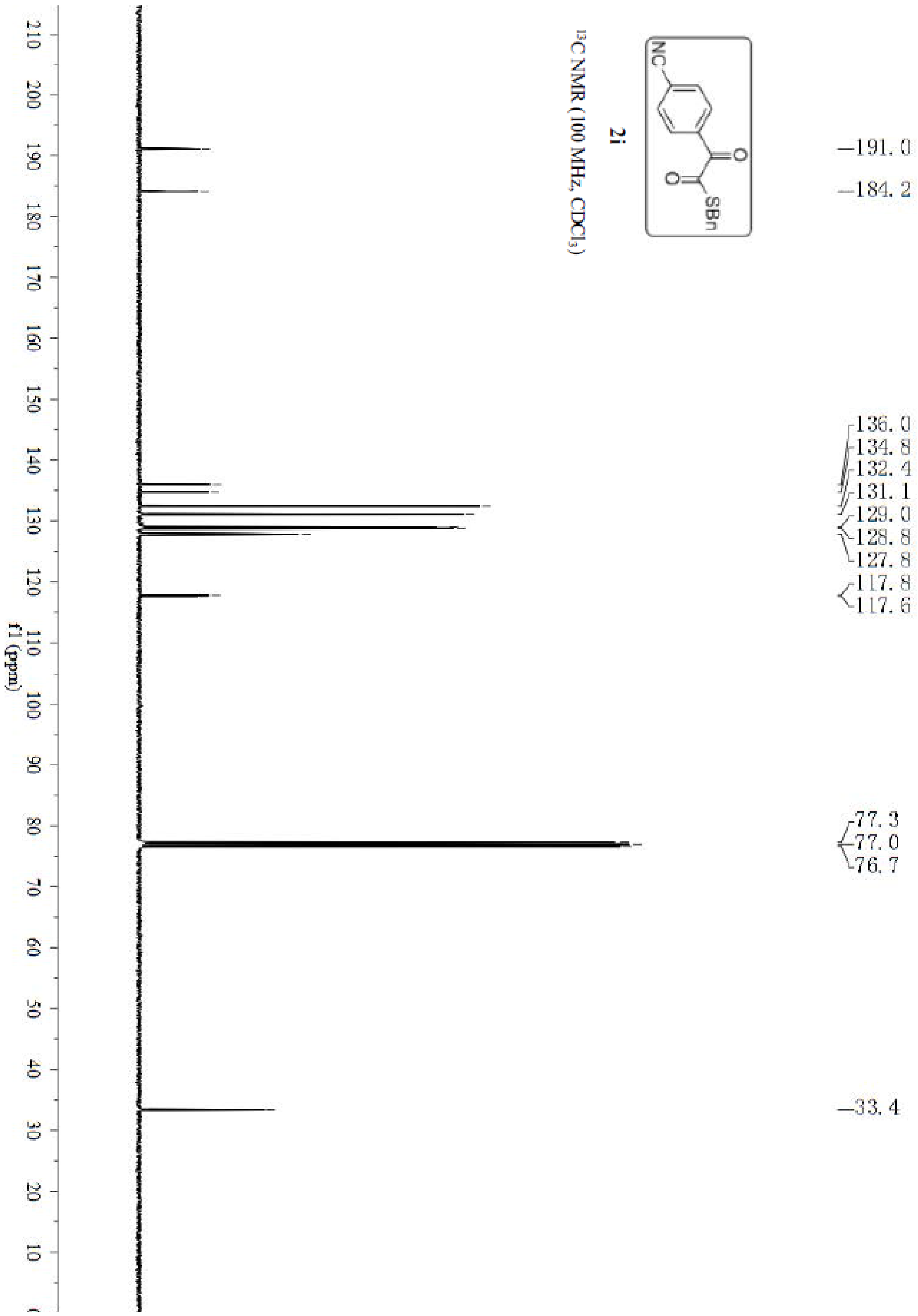

Supplementary Figure 19. ^{13}C NMR (100 MHz, $CDCl_3$) spectra of compound **2i**.

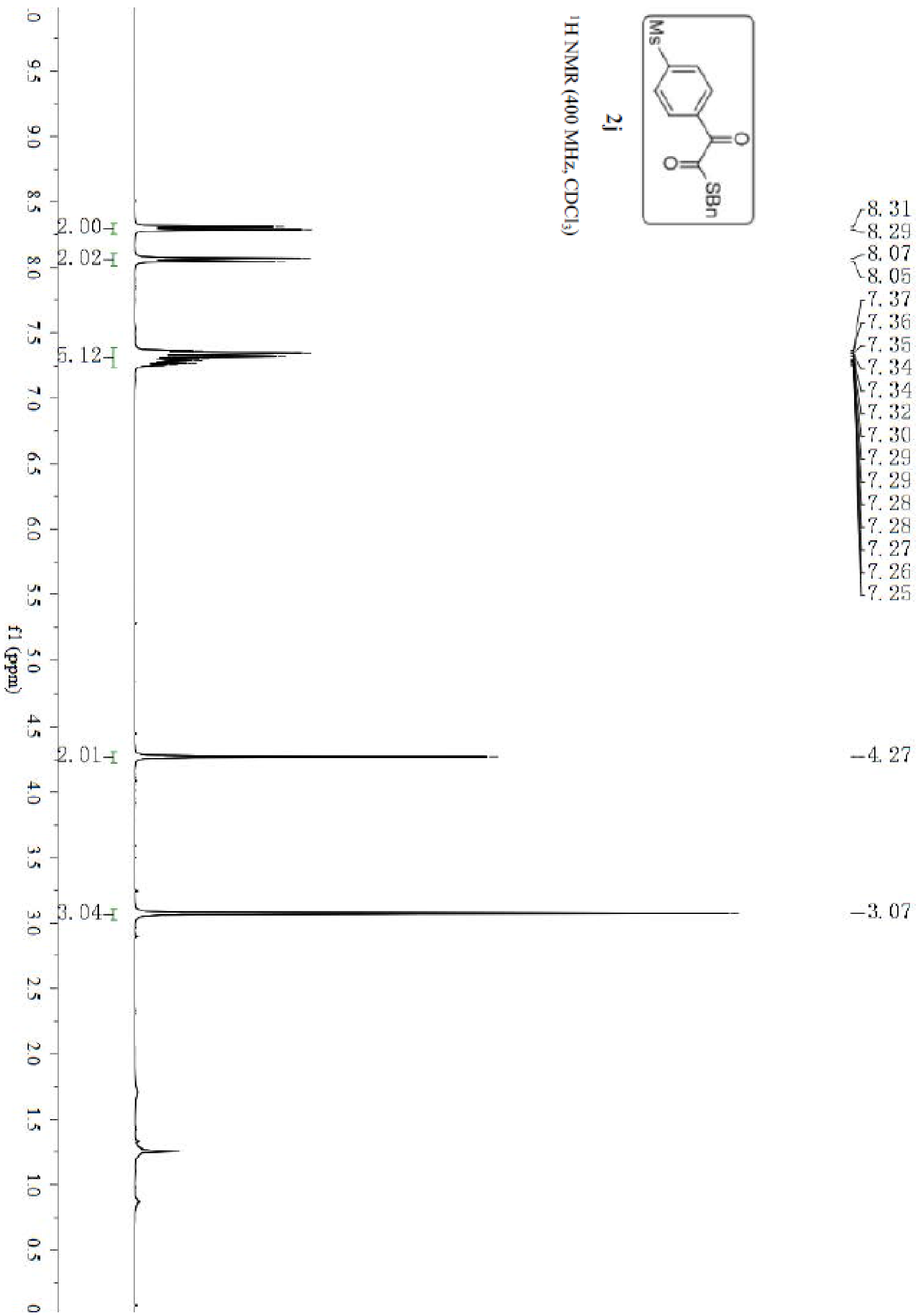

Supplementary Figure 20. ^{1}H NMR (400 MHz, $CDCl_3$) spectra of compound **2j**.

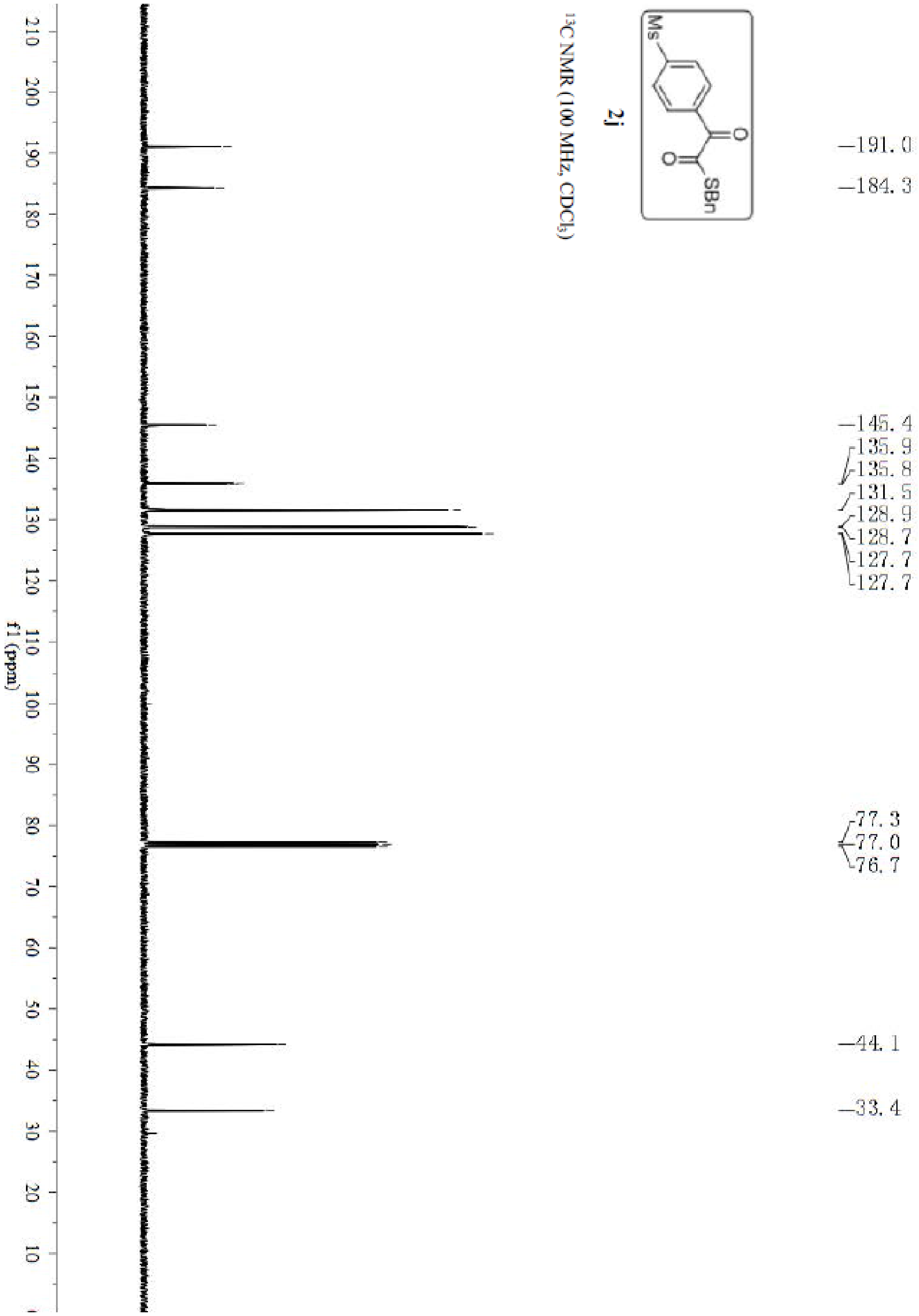

Supplementary Figure 21. ^{13}C NMR (100 MHz, $CDCl_3$) spectra of compound **2j**.

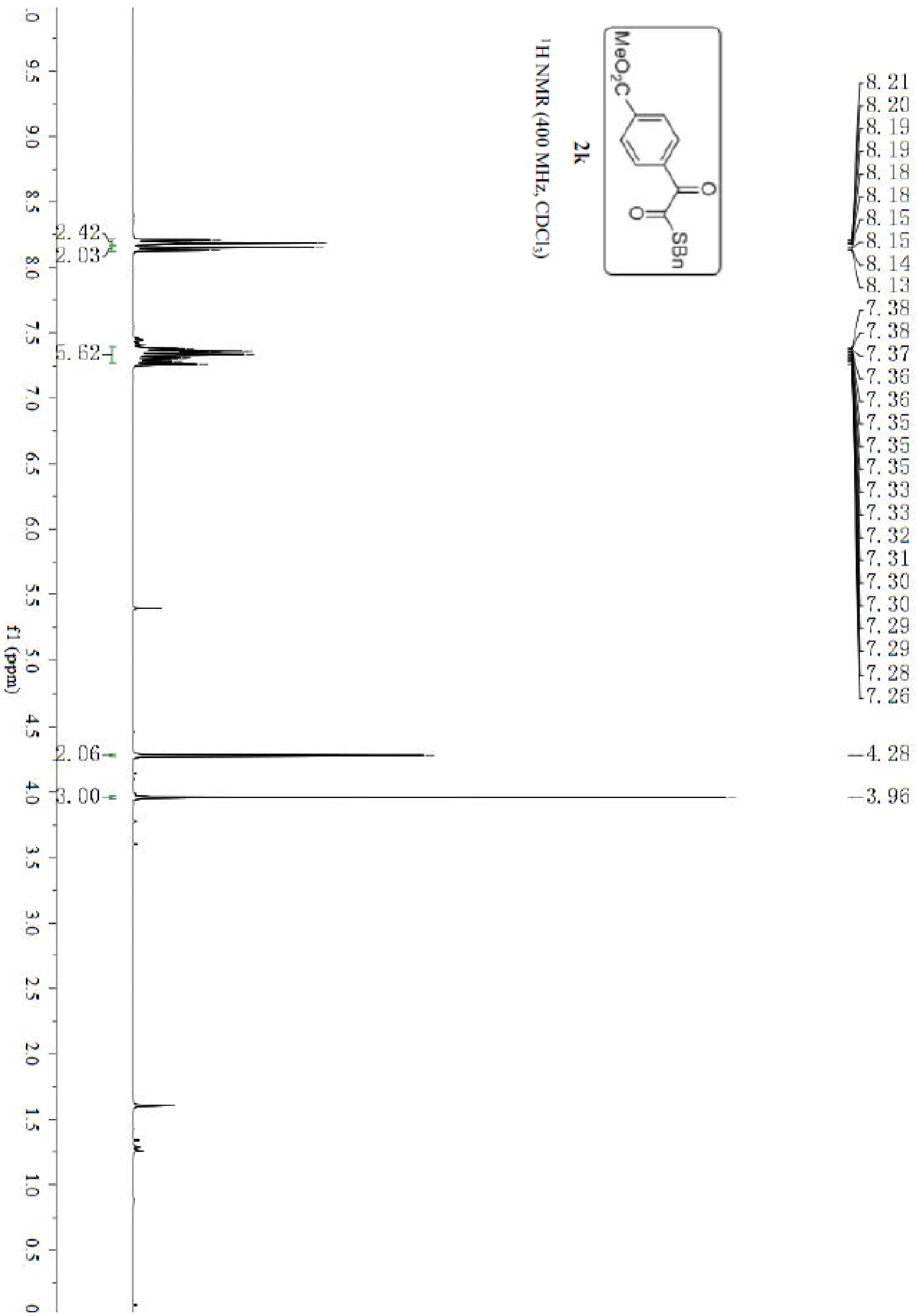

Supplementary Figure 22. ^{1}H NMR (400 MHz, $CDCl_3$) spectra of compound **2k**.

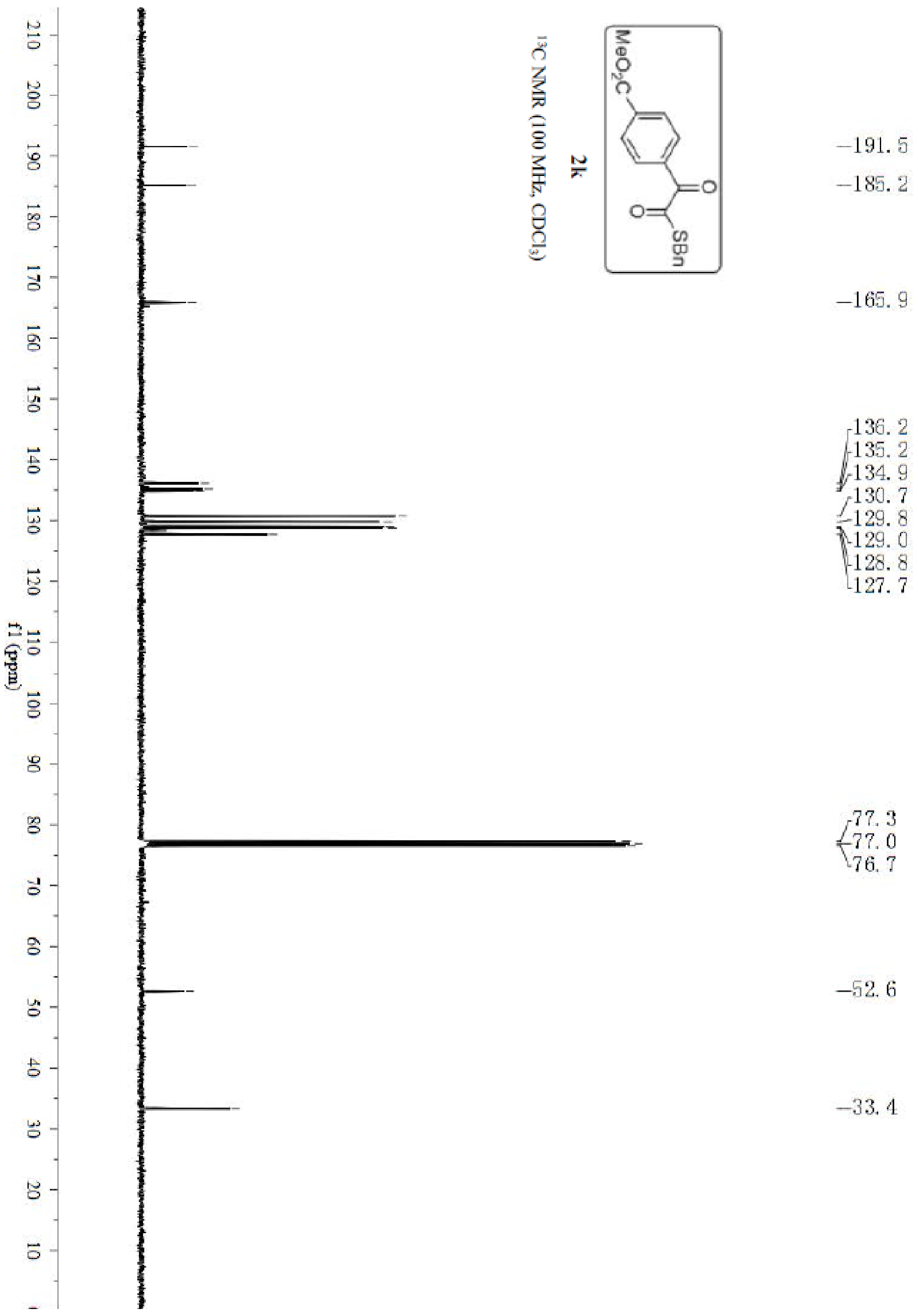

Supplementary Figure 23. ^{13}C NMR (100 MHz, $CDCl_3$) spectra of compound **2k**.

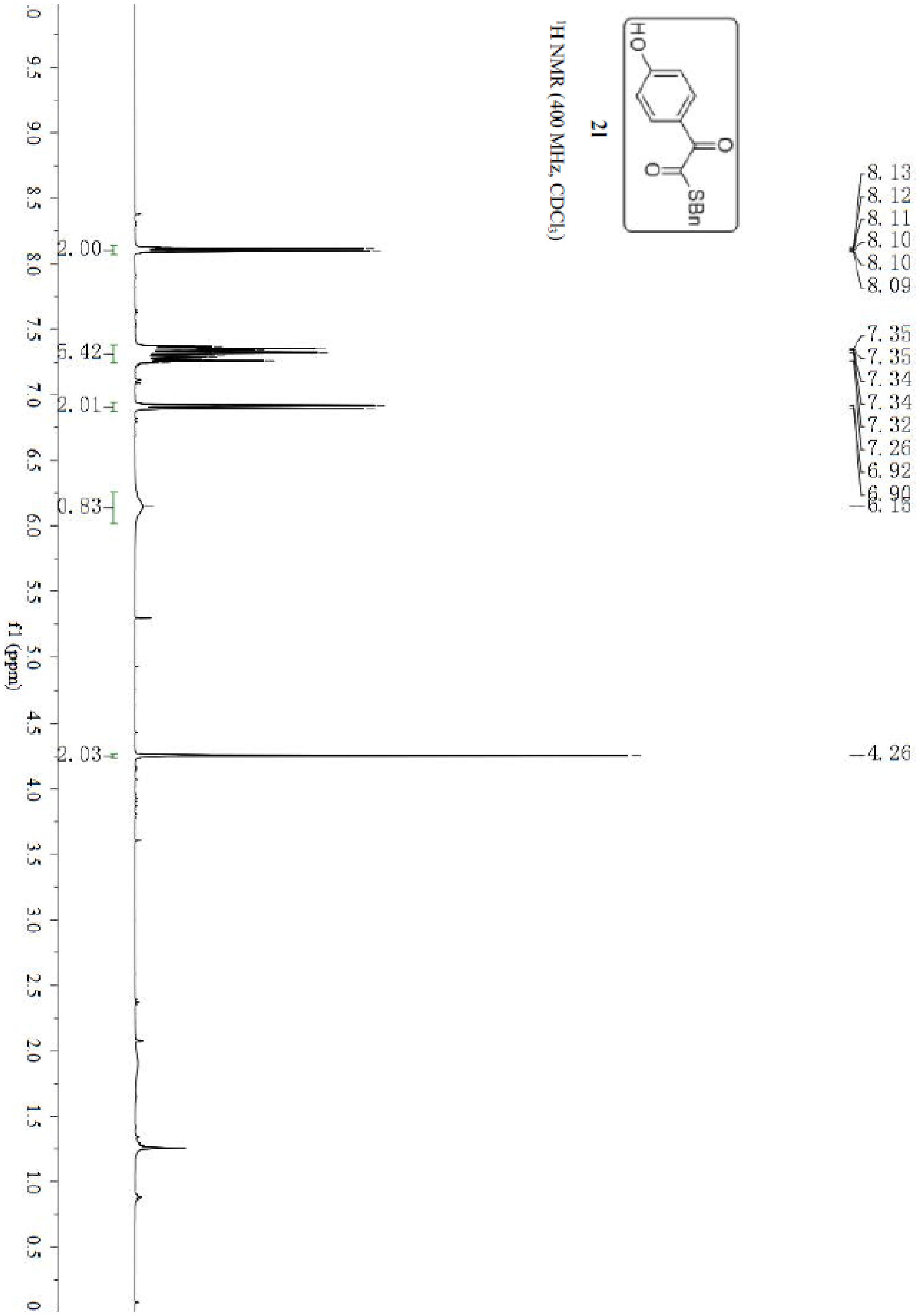

Supplementary Figure 24. ^{1}H NMR (400 MHz, $CDCl_3$) spectra of compound **2l**.

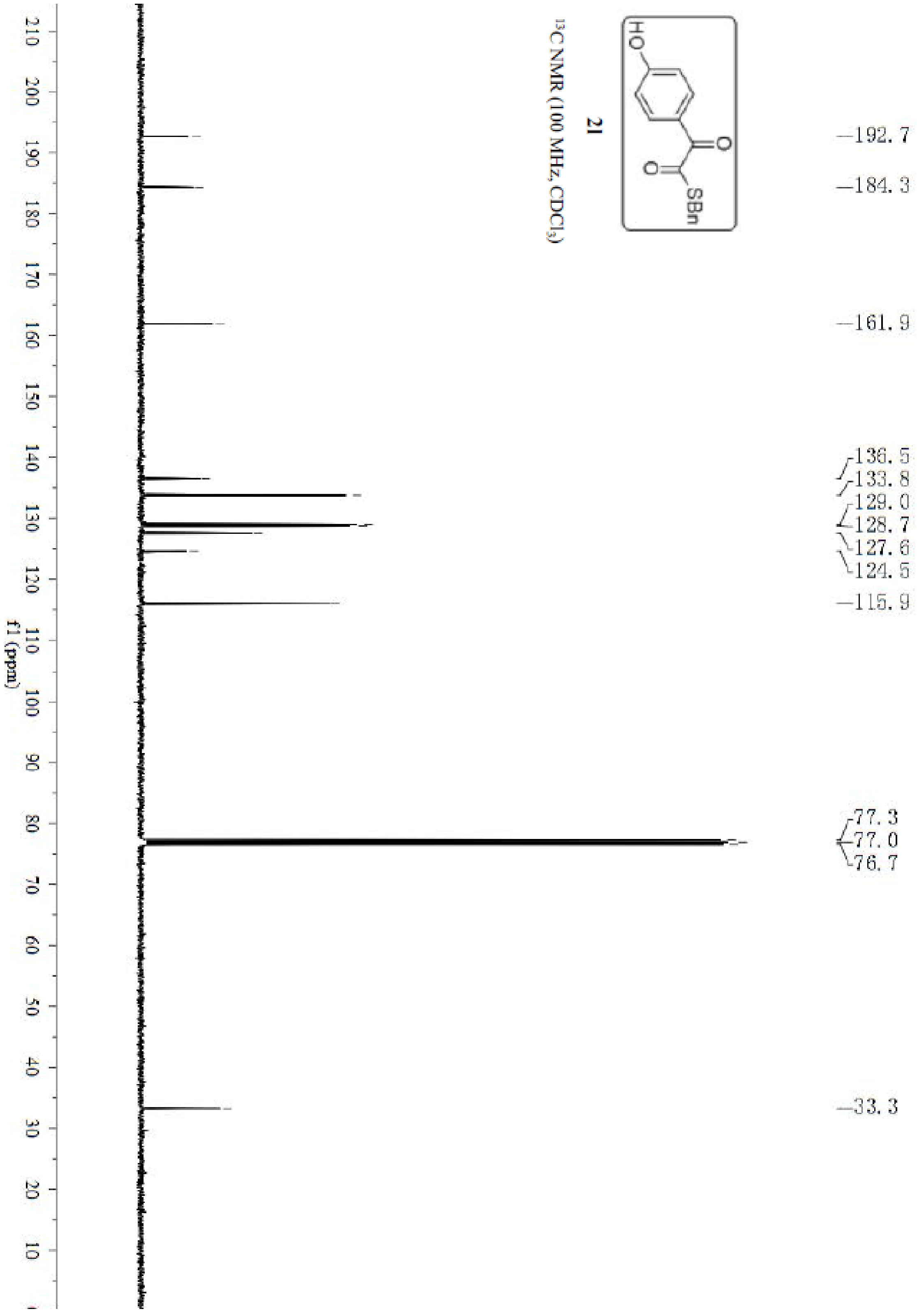

Supplementary Figure 25. ^{13}C NMR (100 MHz, $CDCl_3$) spectra of compound **2l**.

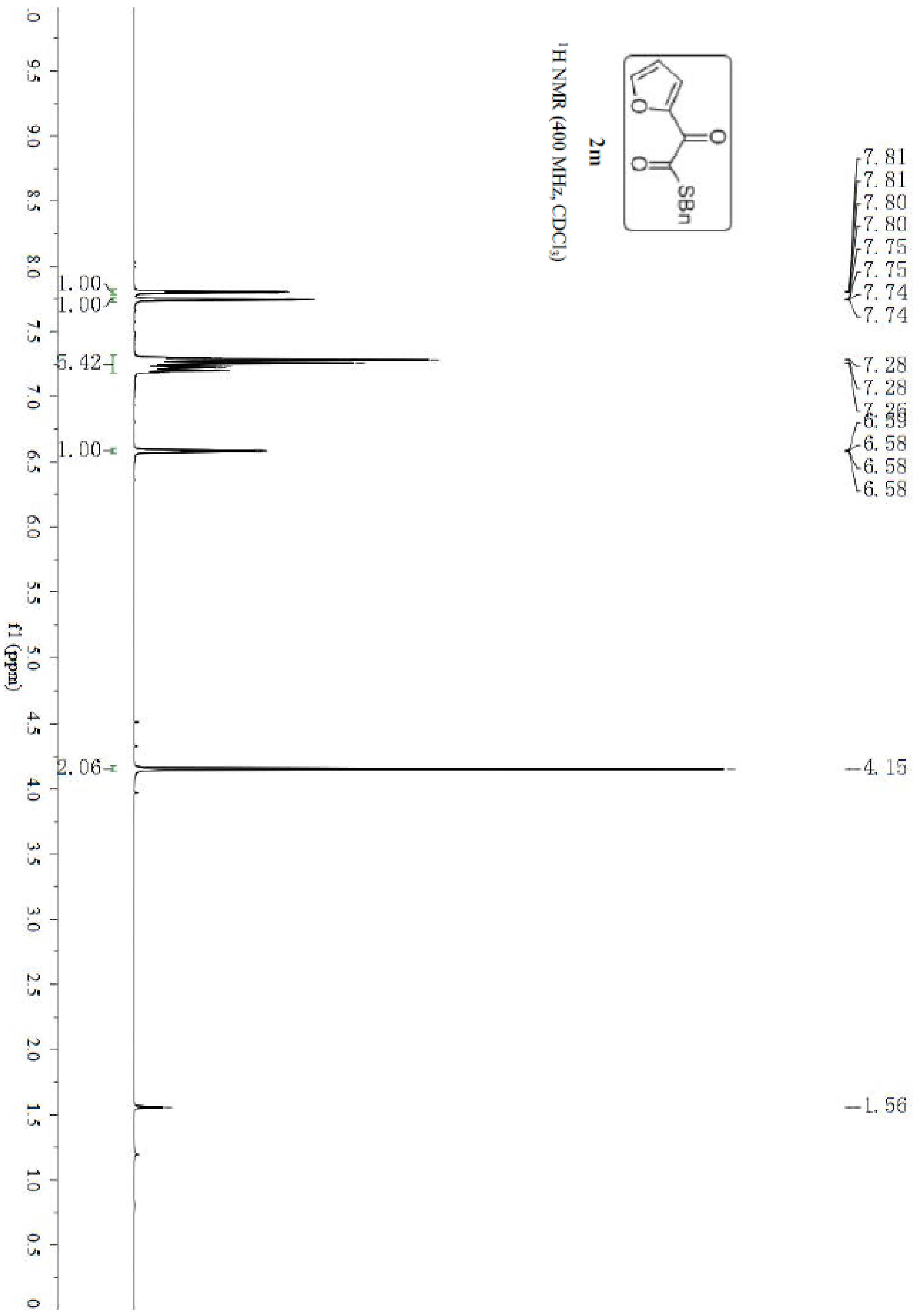

Supplementary Figure 26. ^{1}H NMR (400 MHz, $CDCl_3$) spectra of compound **2m**.

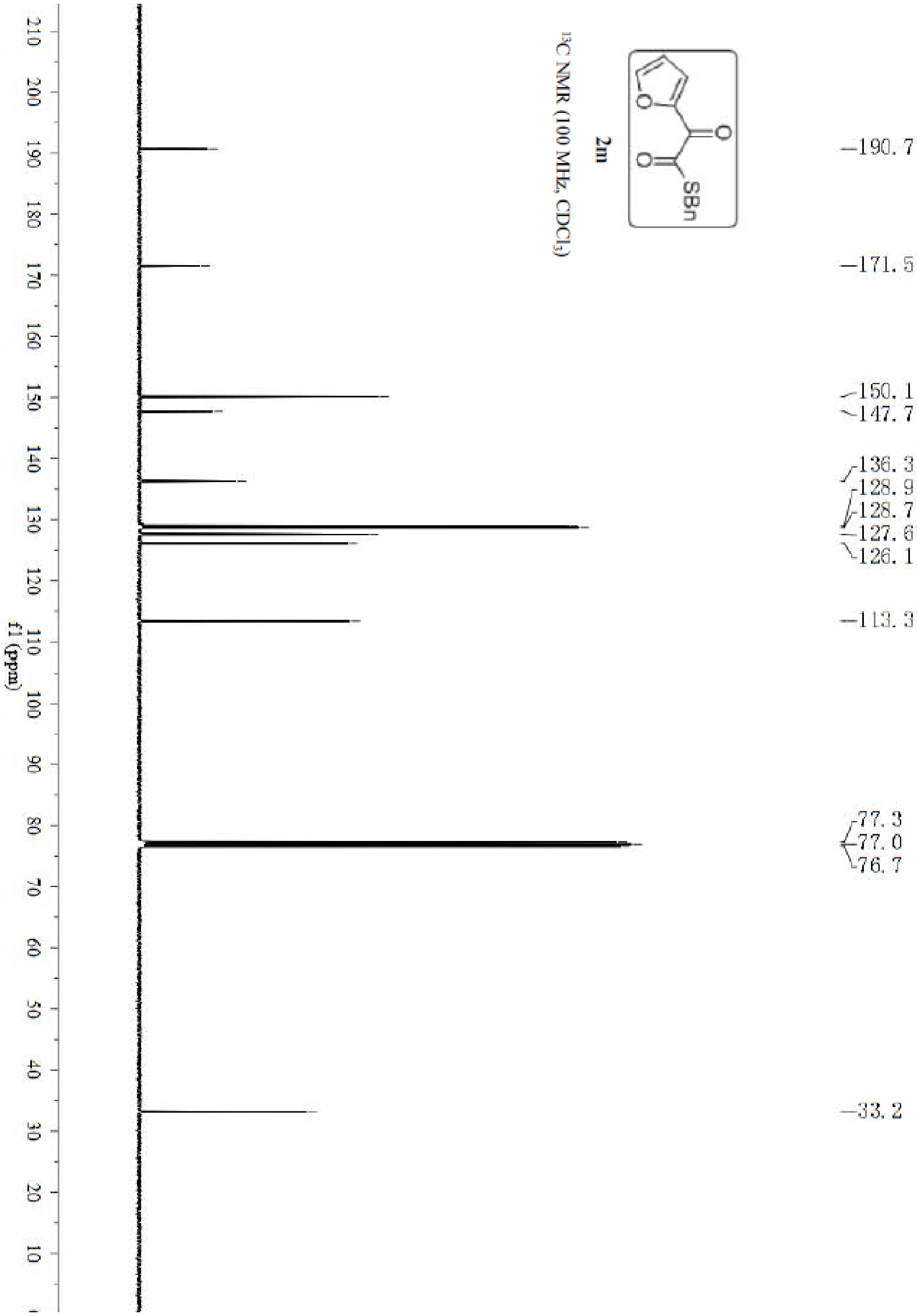

Supplementary Figure 27. ^{13}C NMR (100 MHz, $CDCl_3$) spectra of compound **2m**.

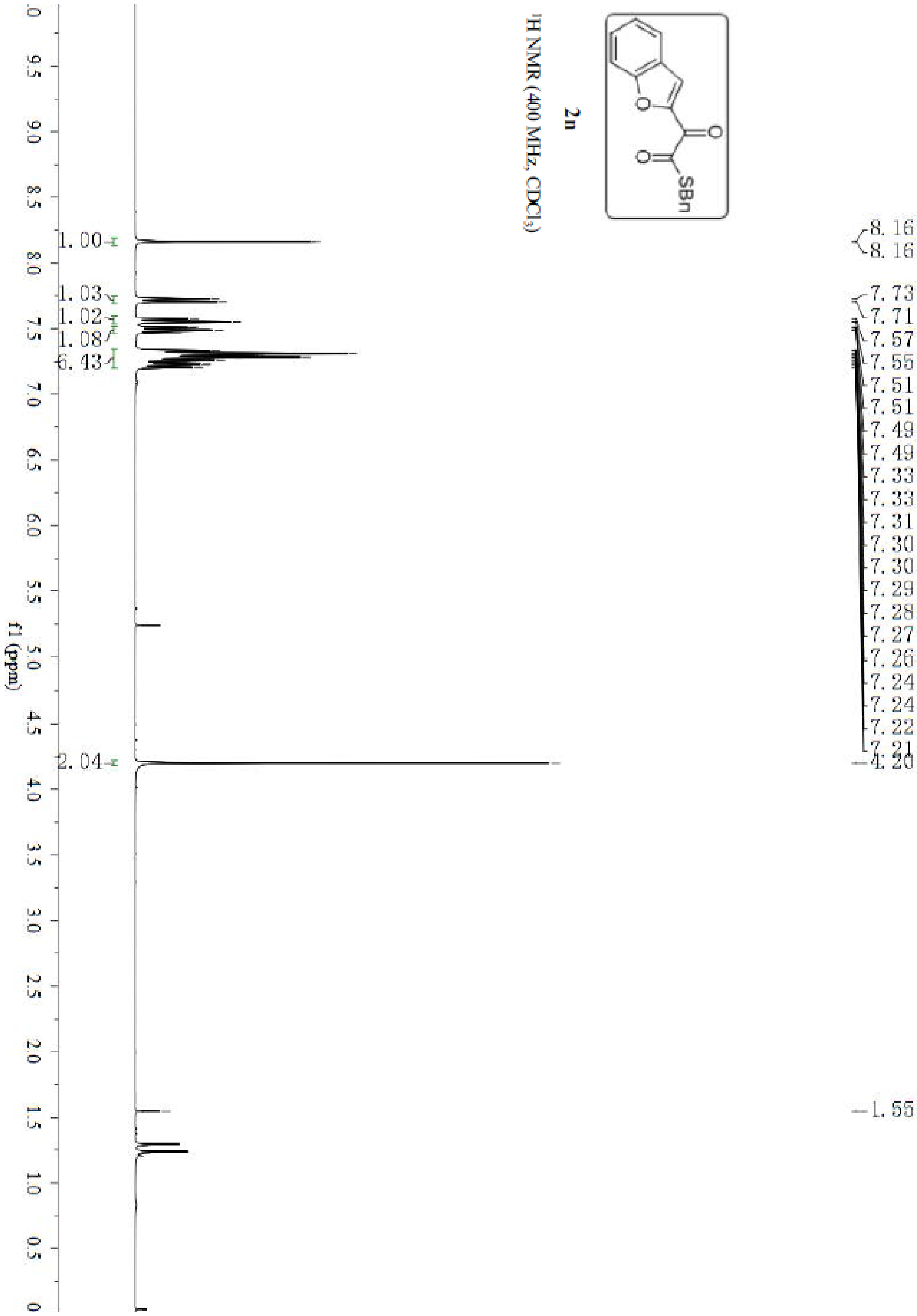

Supplementary Figure 28. ^{1}H NMR (400 MHz, $CDCl_3$) spectra of compound **2n**.

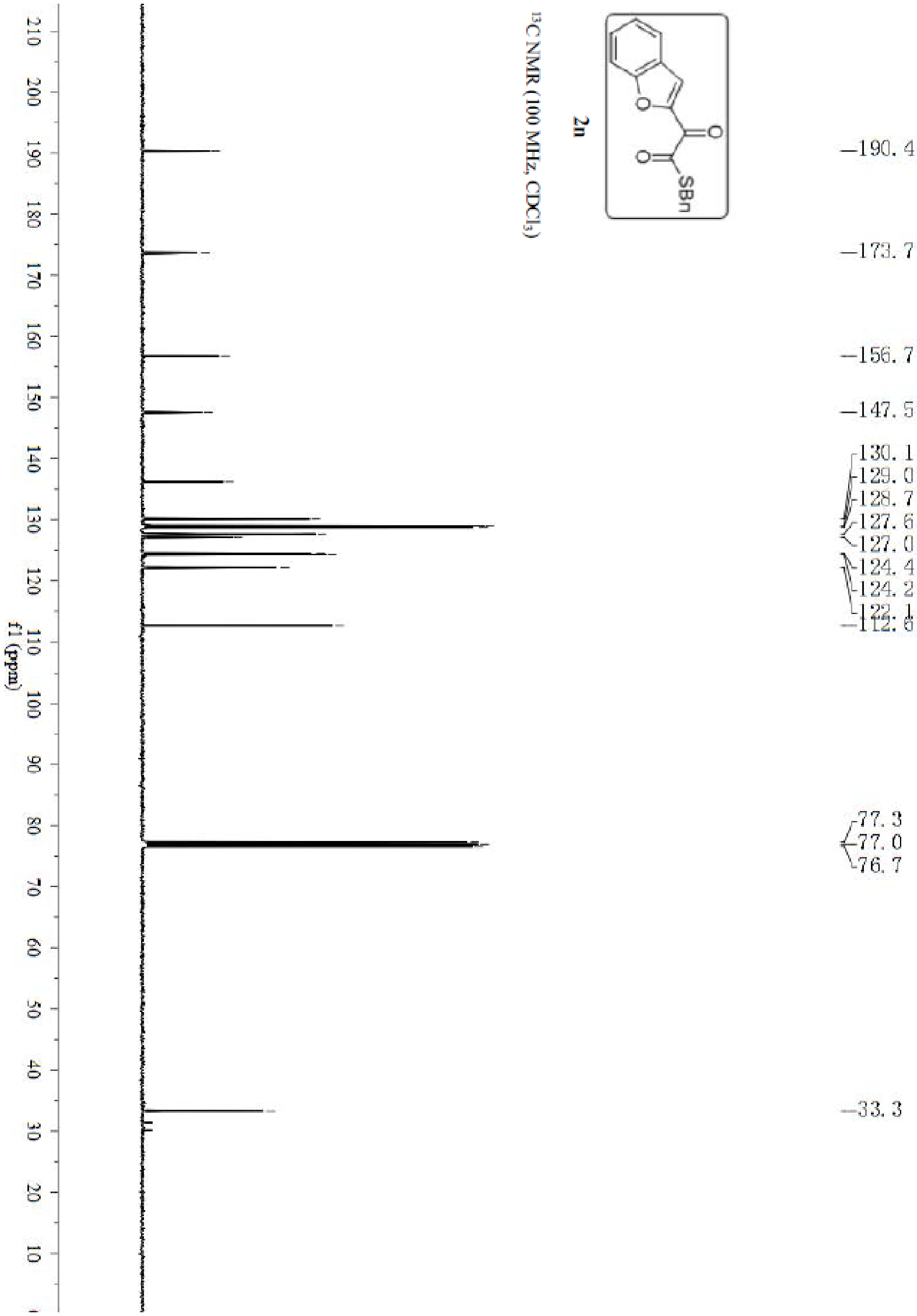

Supplementary Figure 29. ^{13}C NMR (100 MHz, $CDCl_3$) spectra of compound **2n**.

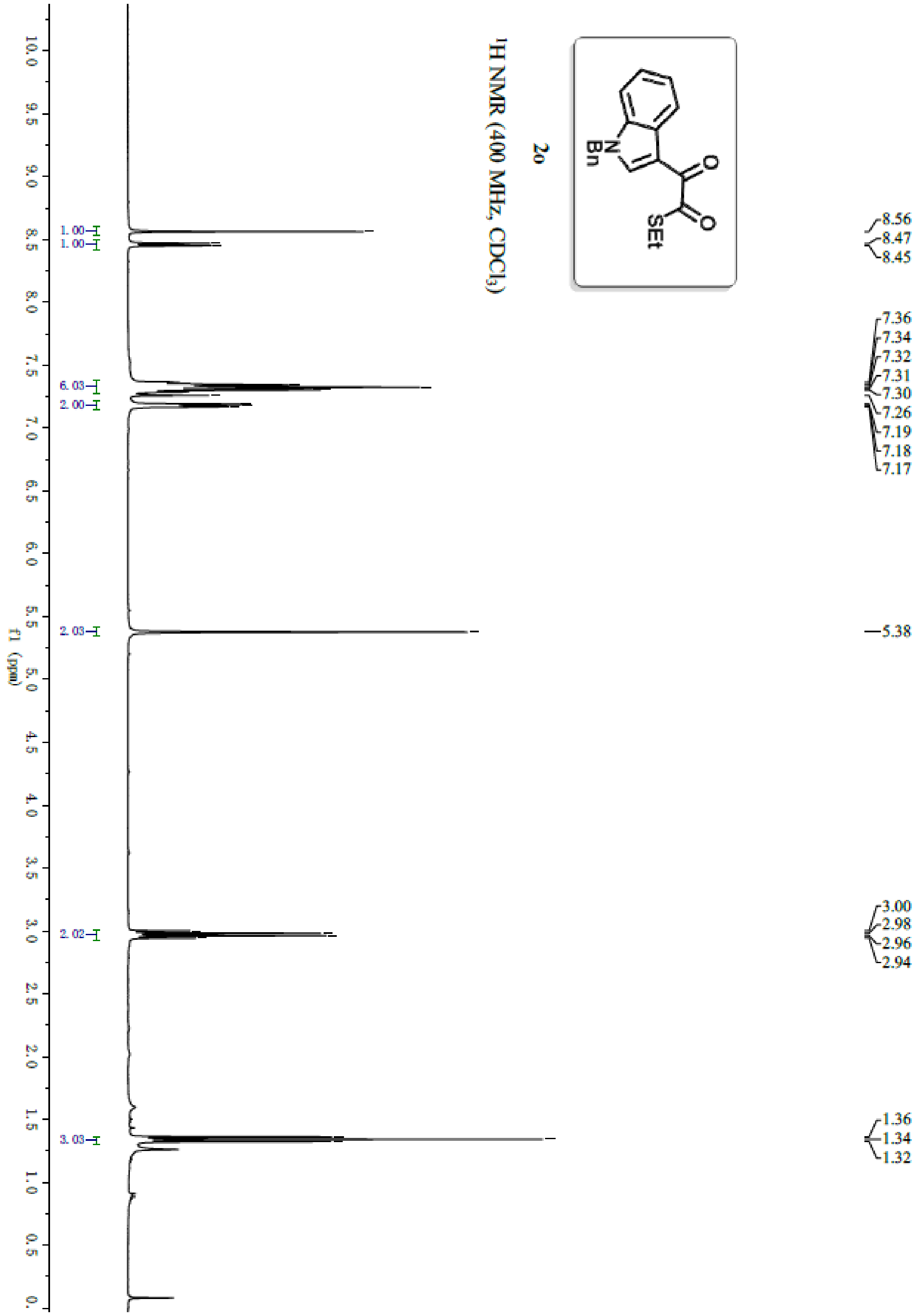

Supplementary Figure 30. ^{1}H NMR (400 MHz, $CDCl_3$) spectra of compound **2o**.

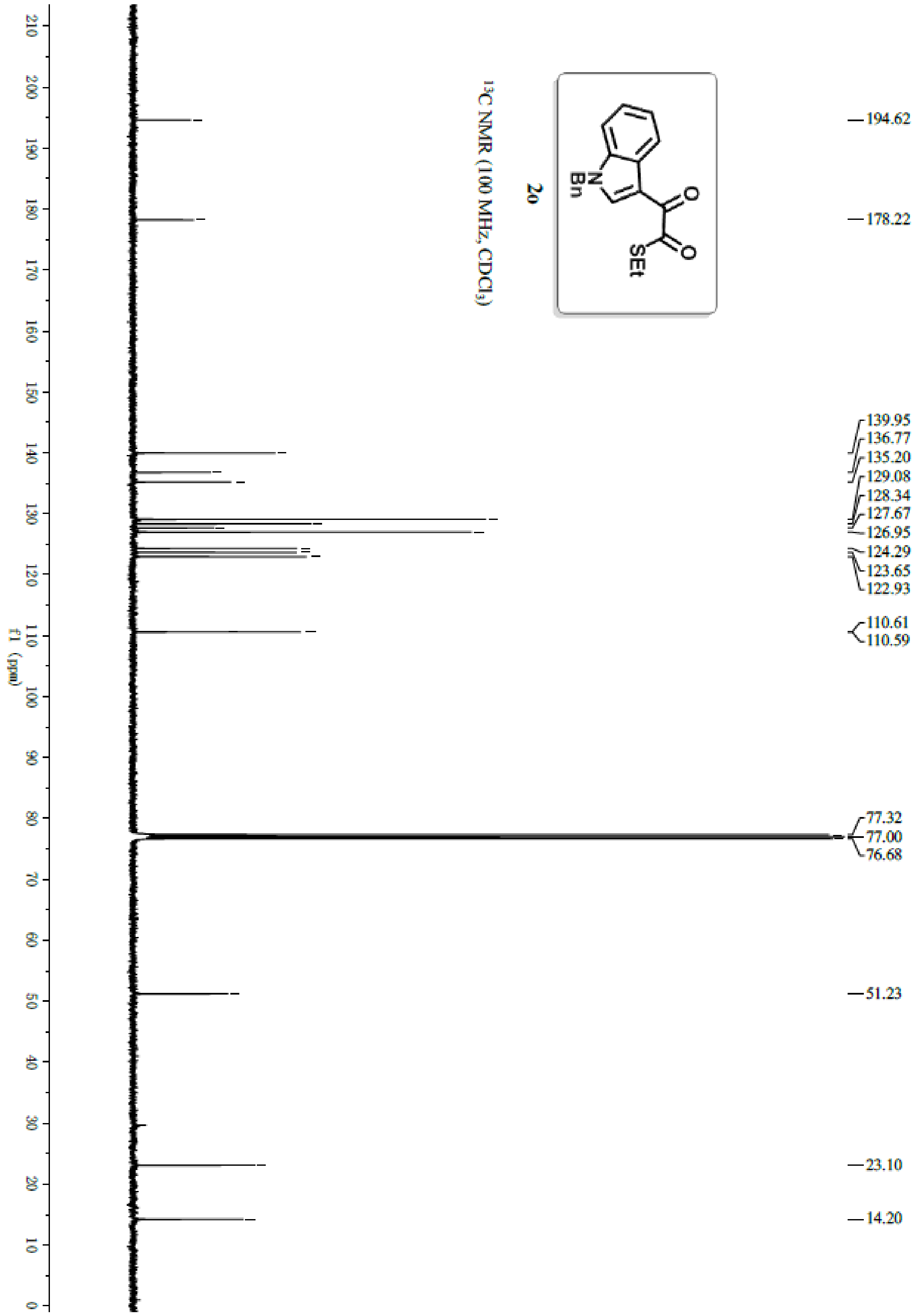

Supplementary Figure 31. ^{13}C NMR (100 MHz, $CDCl_3$) spectra of compound **2o**.

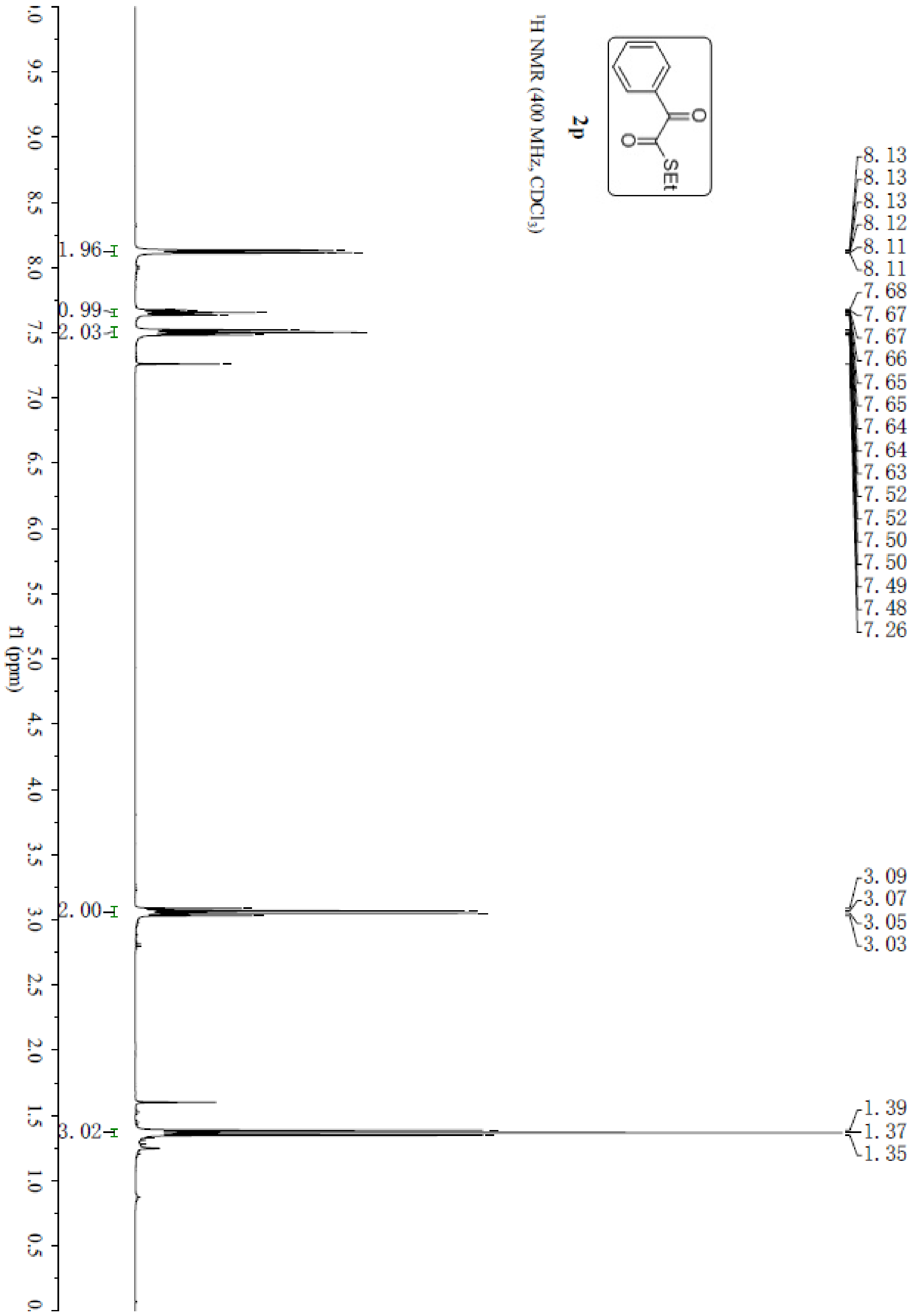

Supplementary Figure 32. ^{1}H NMR (400 MHz, $CDCl_3$) spectra of compound **2p**.

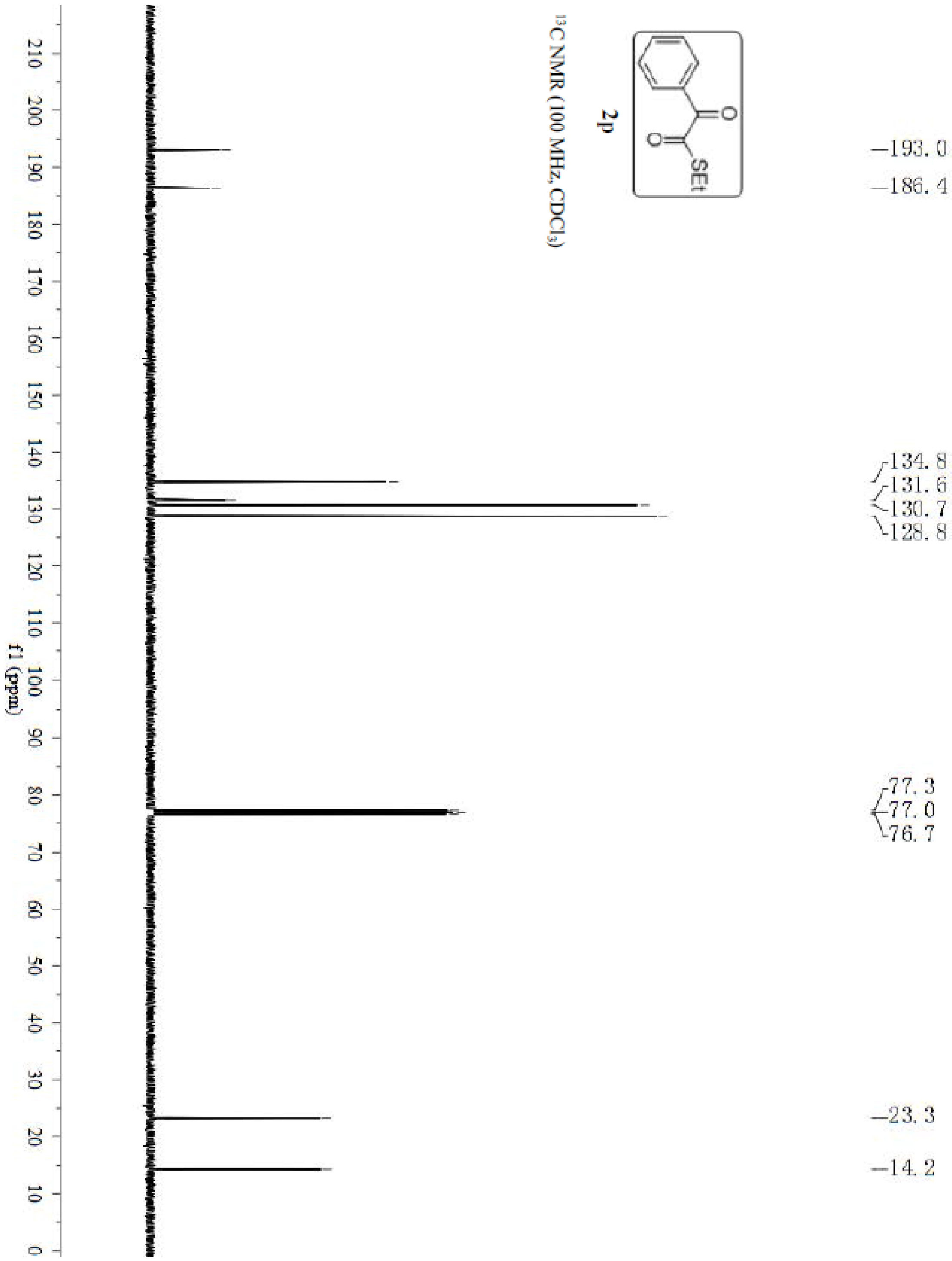

Supplementary Figure 33. ^{13}C NMR (100 MHz, $CDCl_3$) spectra of compound **2p**.

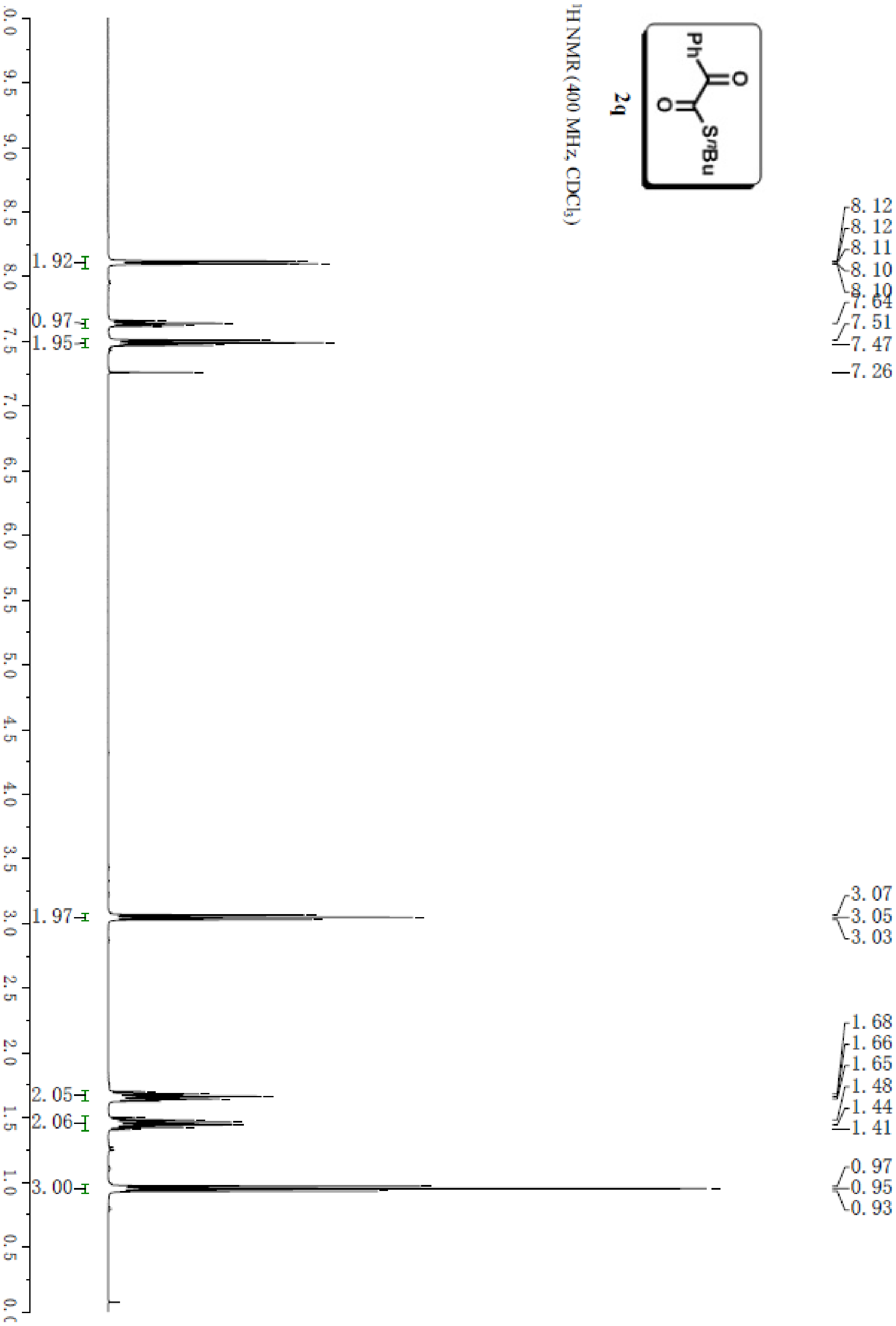

Supplementary Figure 34. ^{1}H NMR (400 MHz, $CDCl_3$) spectra of compound **2q**.

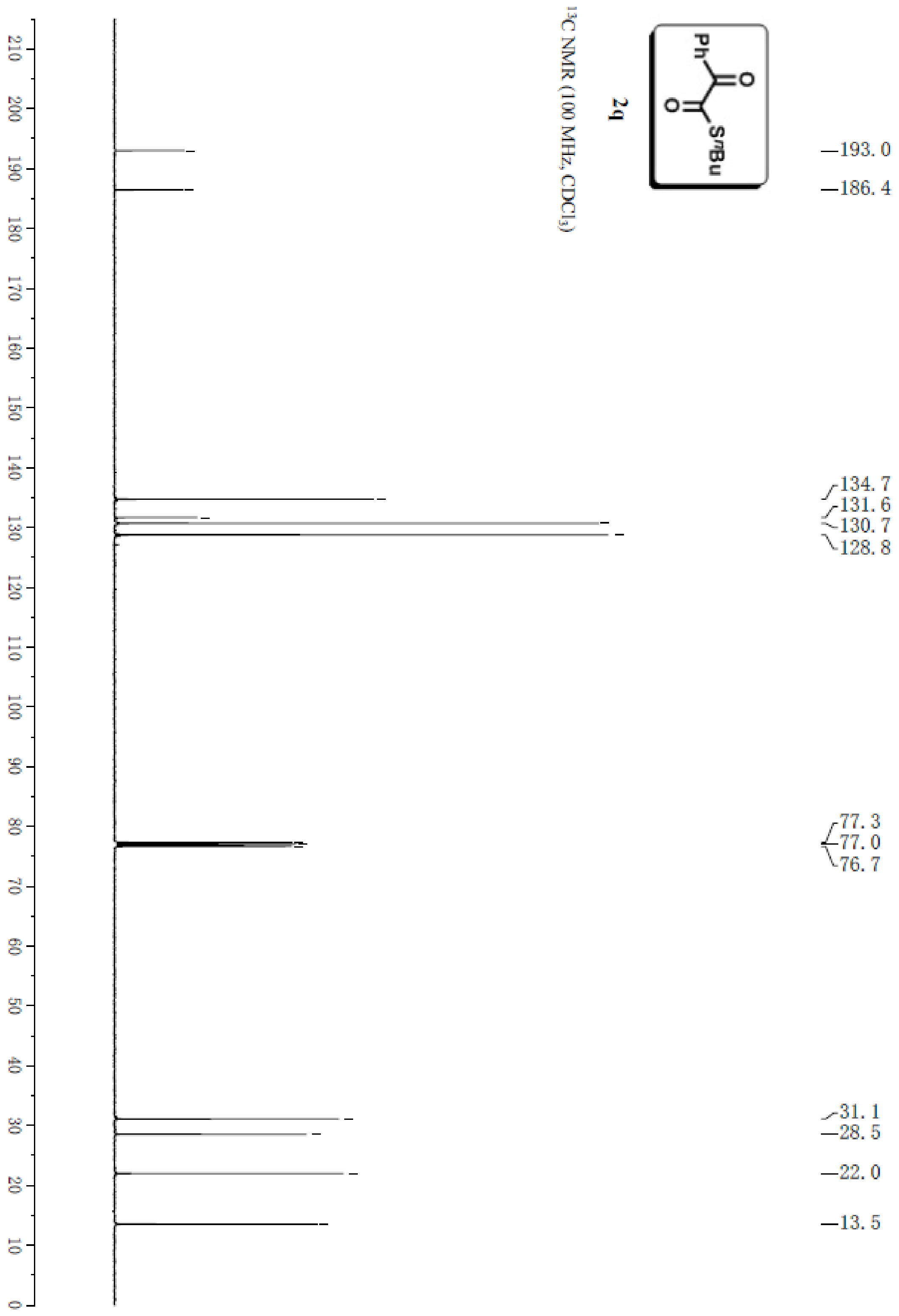

Supplementary Figure 35. ^{13}C NMR (100 MHz, $CDCl_3$) spectra of compound **2q**.

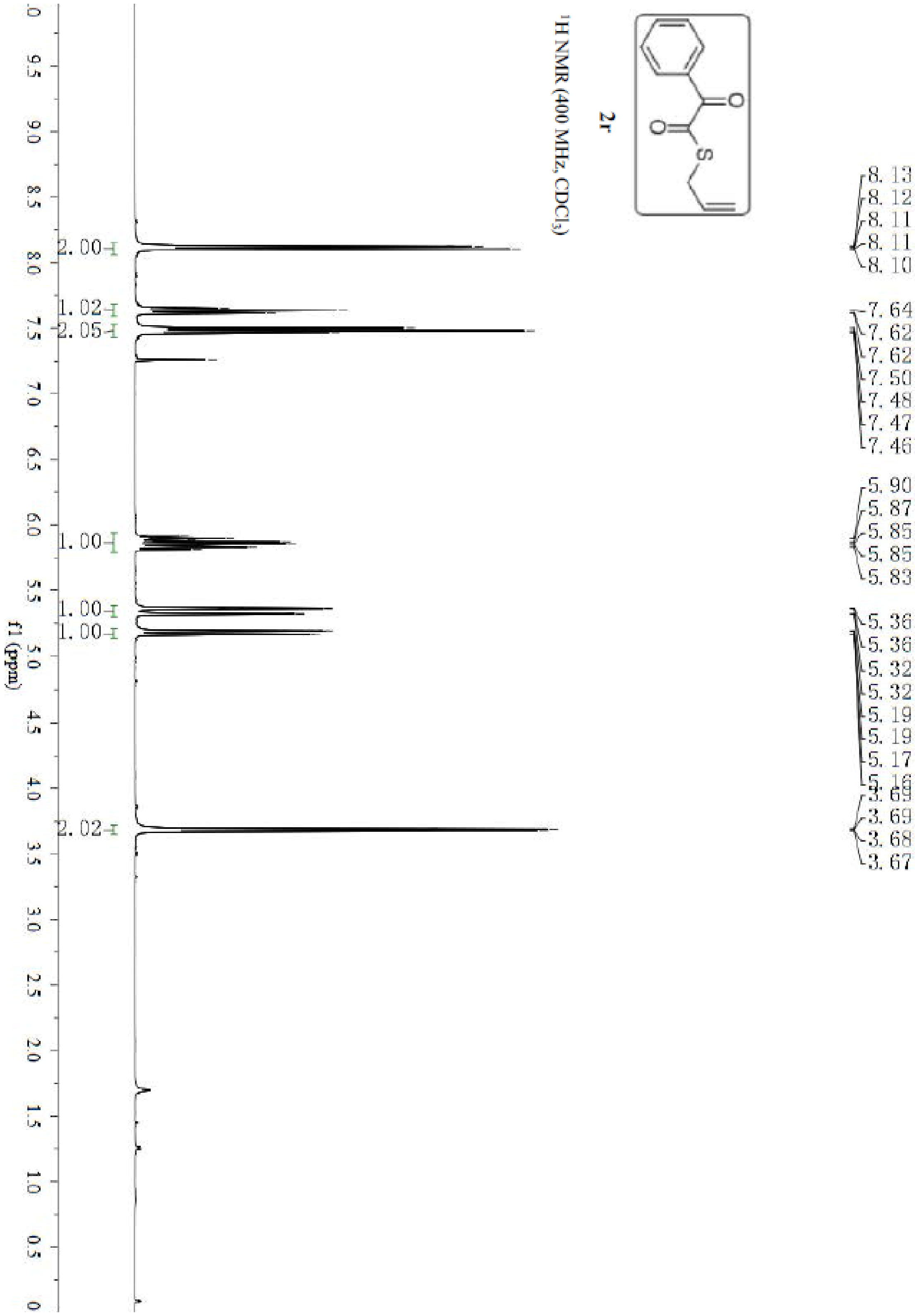

Supplementary Figure 36. ^{1}H NMR (400 MHz, $CDCl_3$) spectra of compound **2r**.

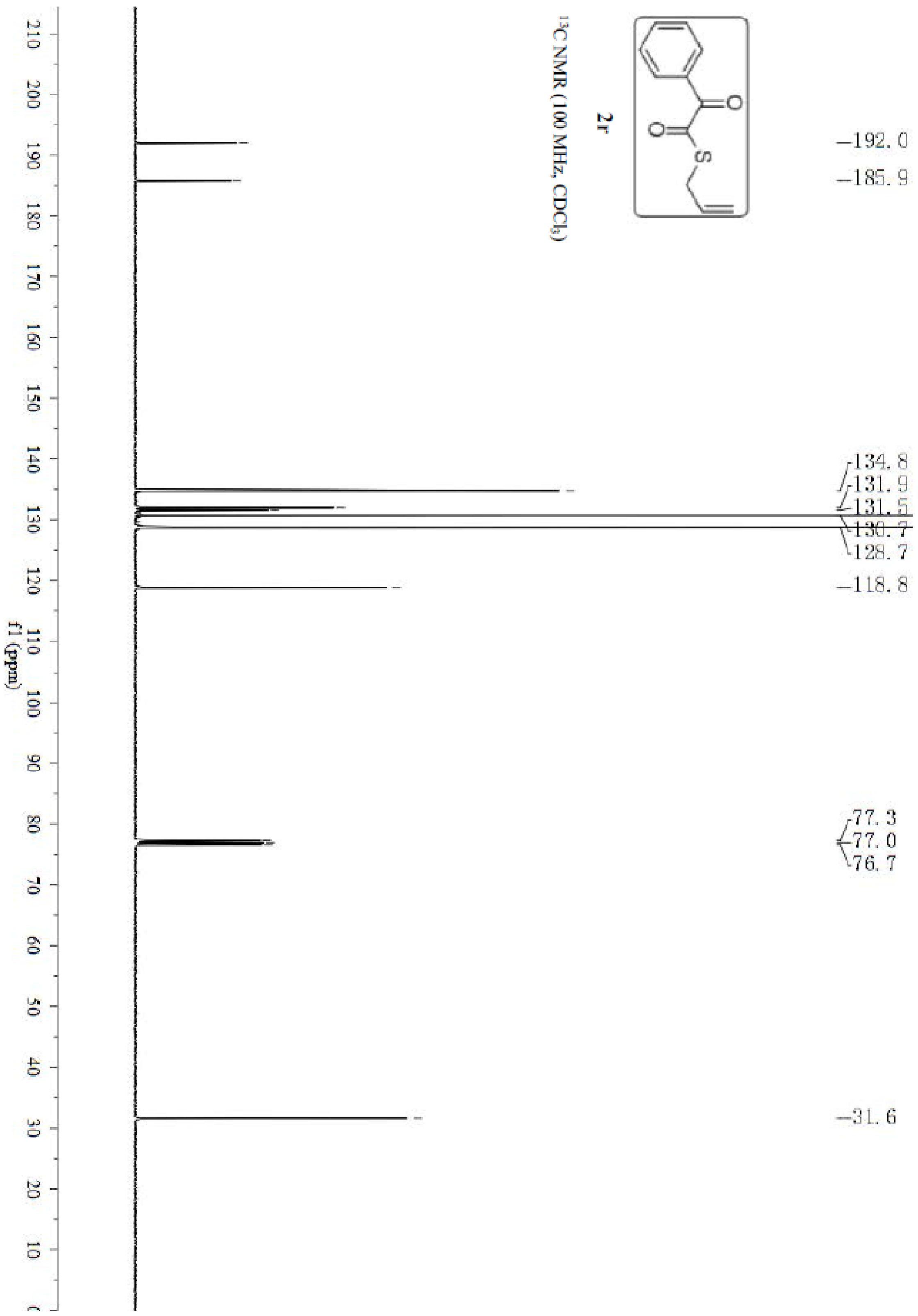

Supplementary Figure 37. ^{13}C NMR (100 MHz, $CDCl_3$) spectra of compound **2r**.

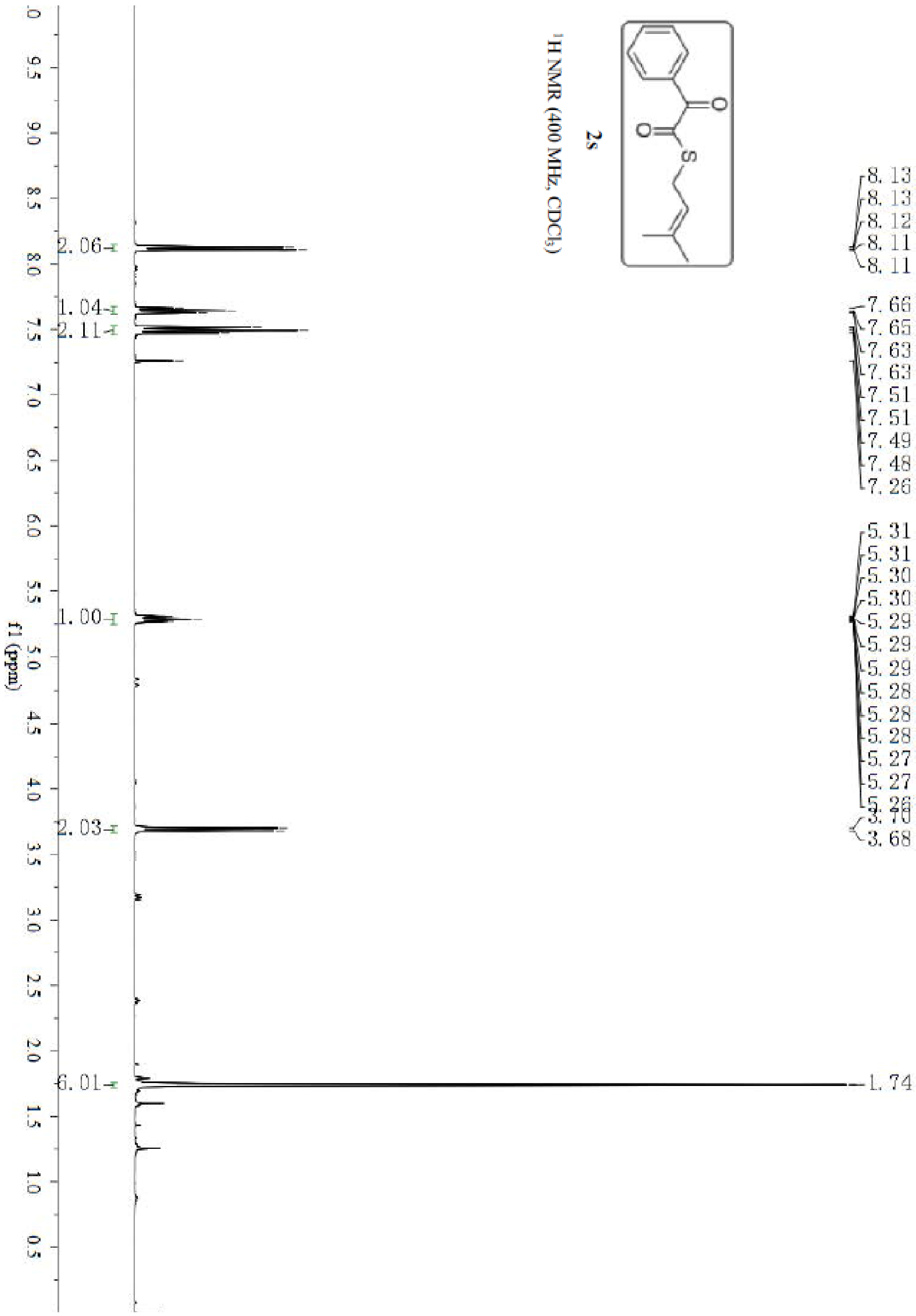

Supplementary Figure 38. ^{1}H NMR (400 MHz, $CDCl_3$) spectra of compound **2s**.

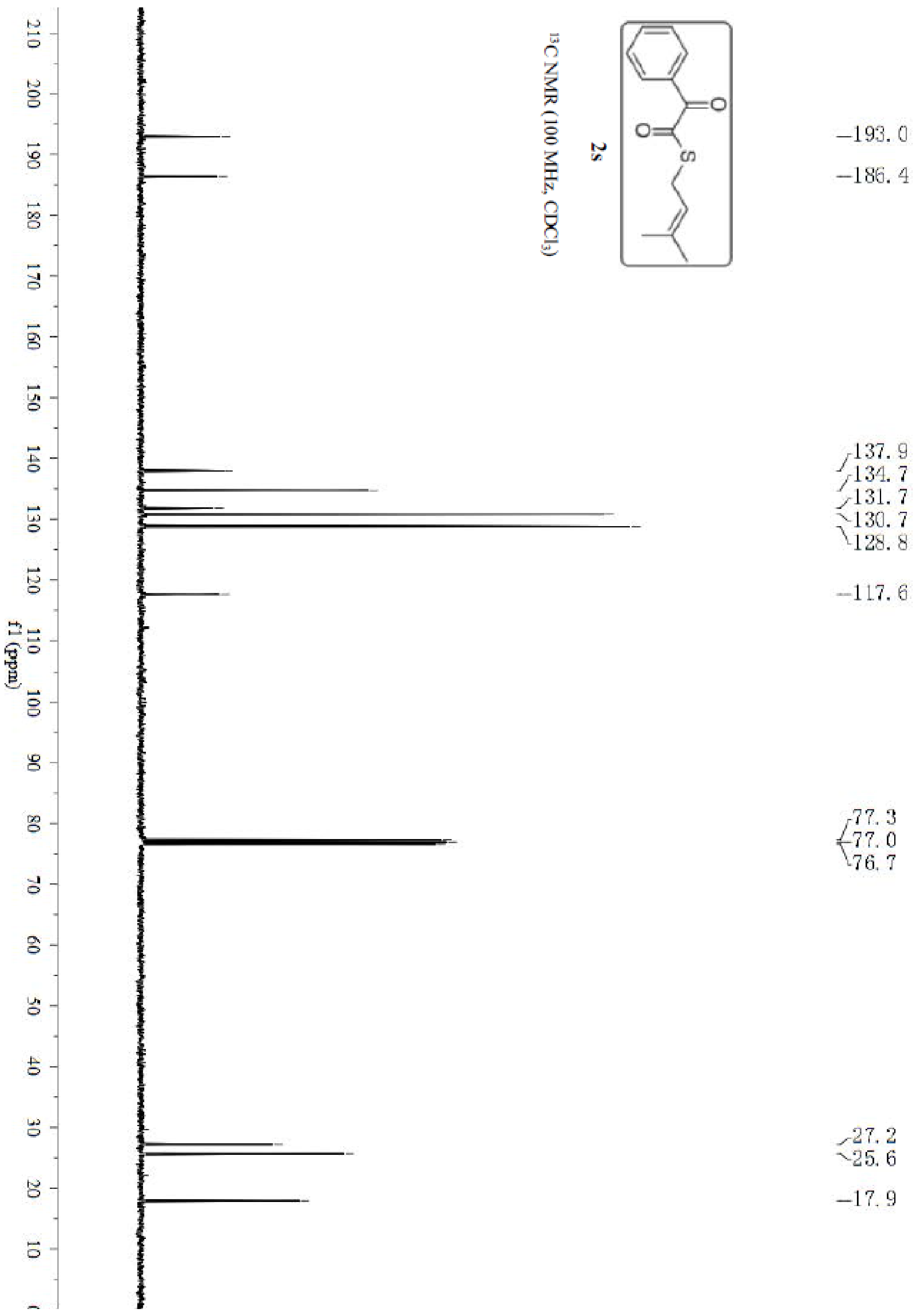

Supplementary Figure 39. ^{13}C NMR (100 MHz, $CDCl_3$) spectra of compound **2s**.

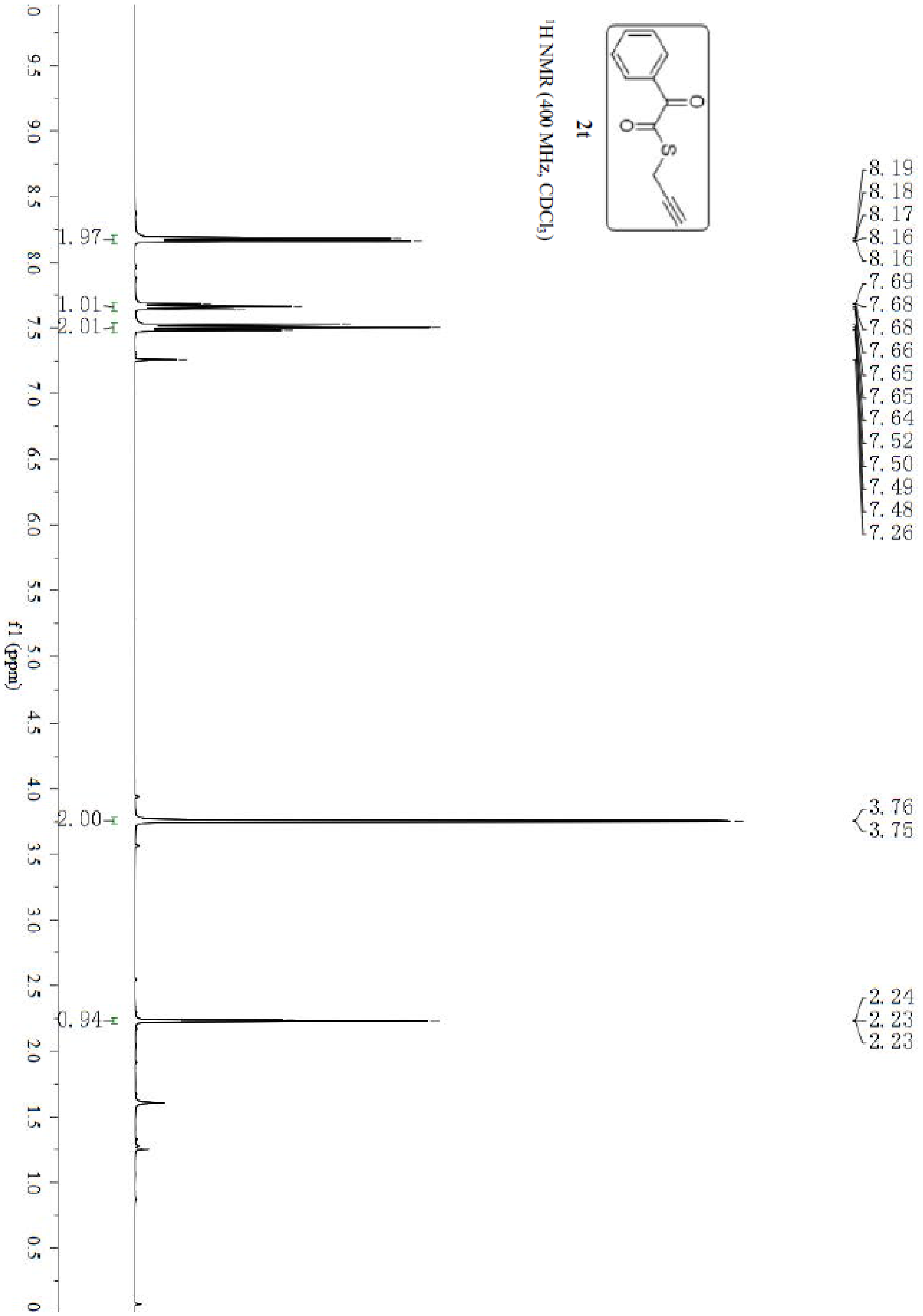

Supplementary Figure 40. ^{1}H NMR (400 MHz, $CDCl_3$) spectra of compound **2t**.

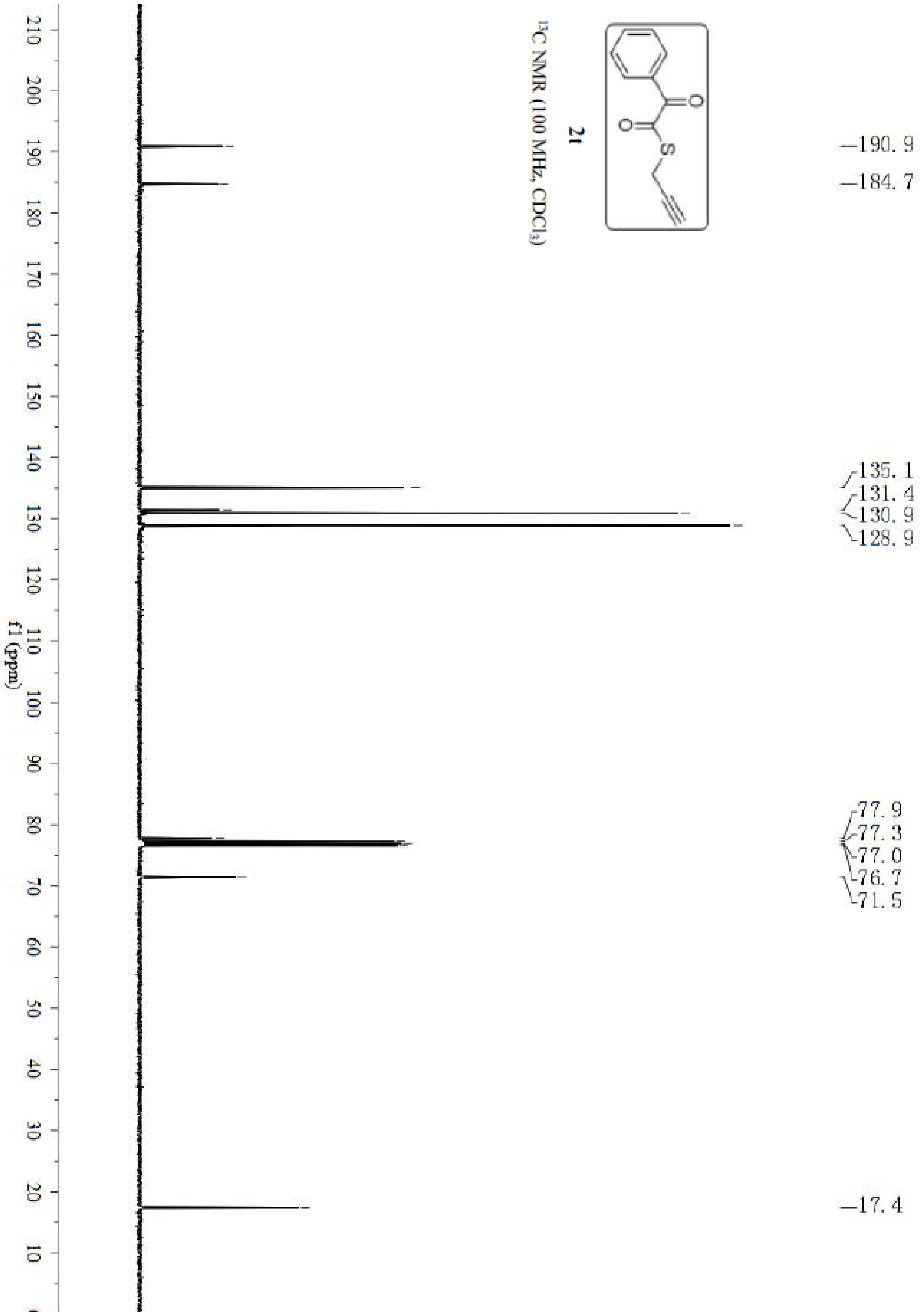

Supplementary Figure 41. ^{13}C NMR (100 MHz, $CDCl_3$) spectra of compound **2t**.

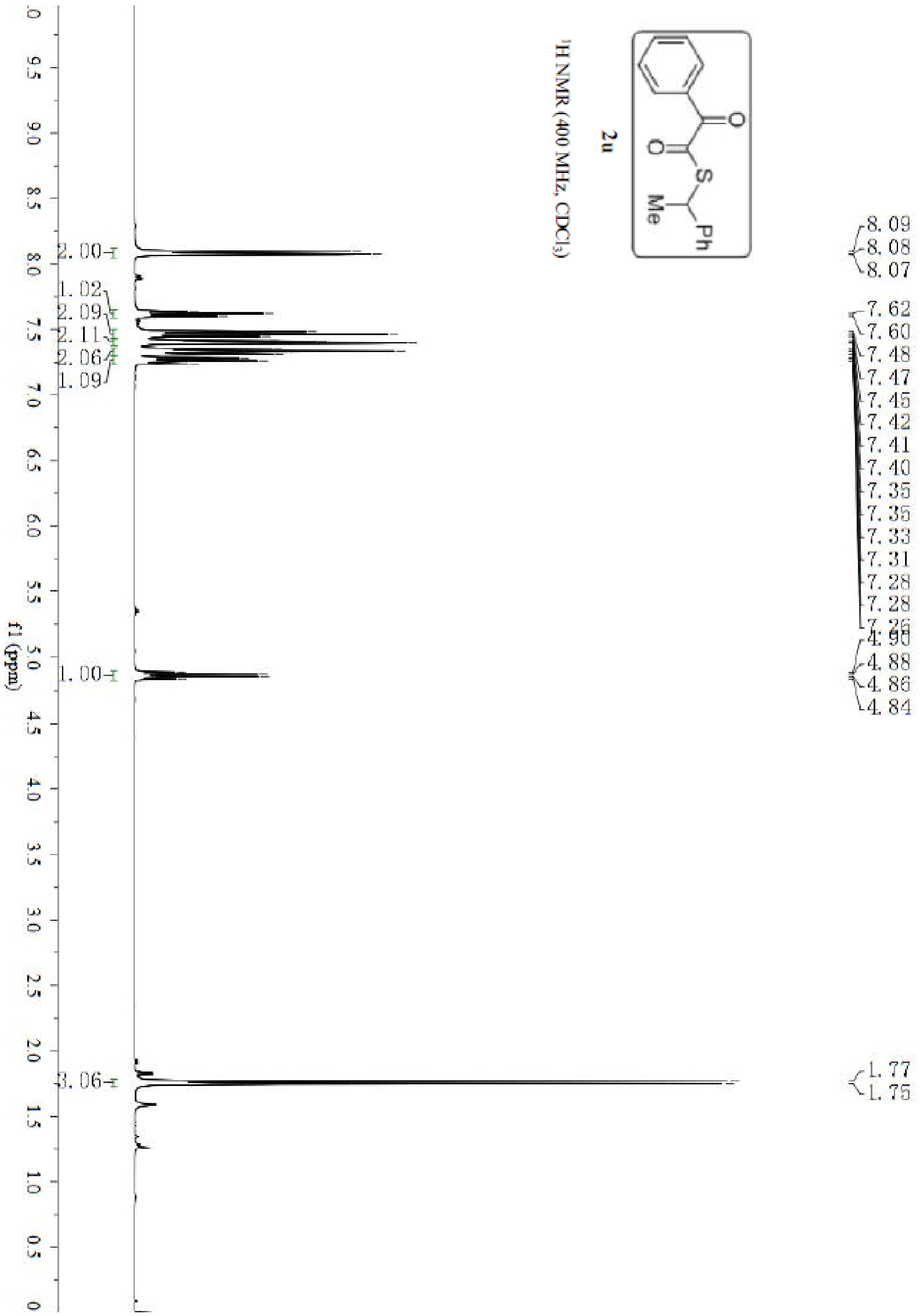

Supplementary Figure 42. ^{1}H NMR (400 MHz, $CDCl_3$) spectra of compound **2u**.

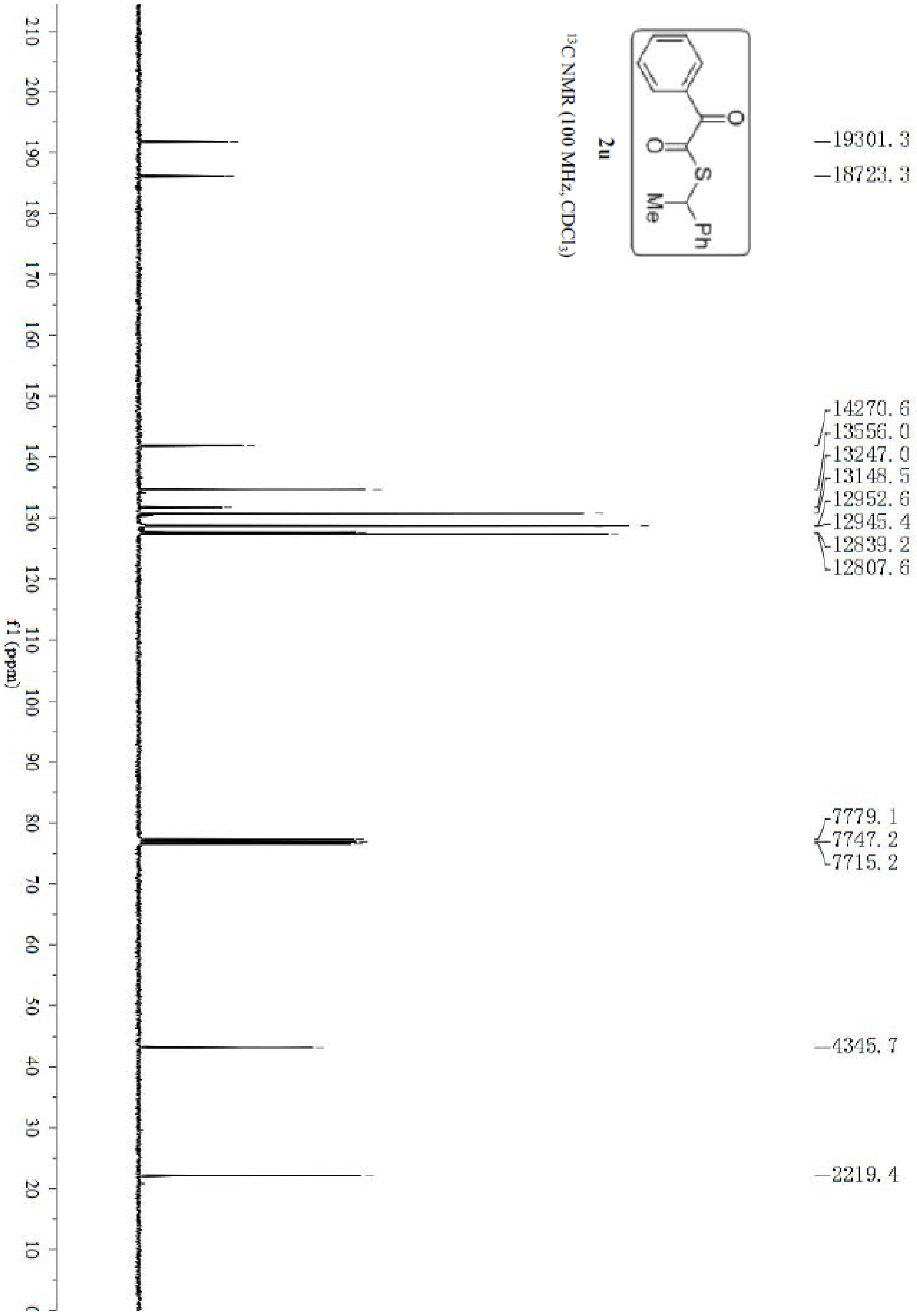

Supplementary Figure 43. ^{13}C NMR (100 MHz, $CDCl_3$) spectra of compound **2u**.

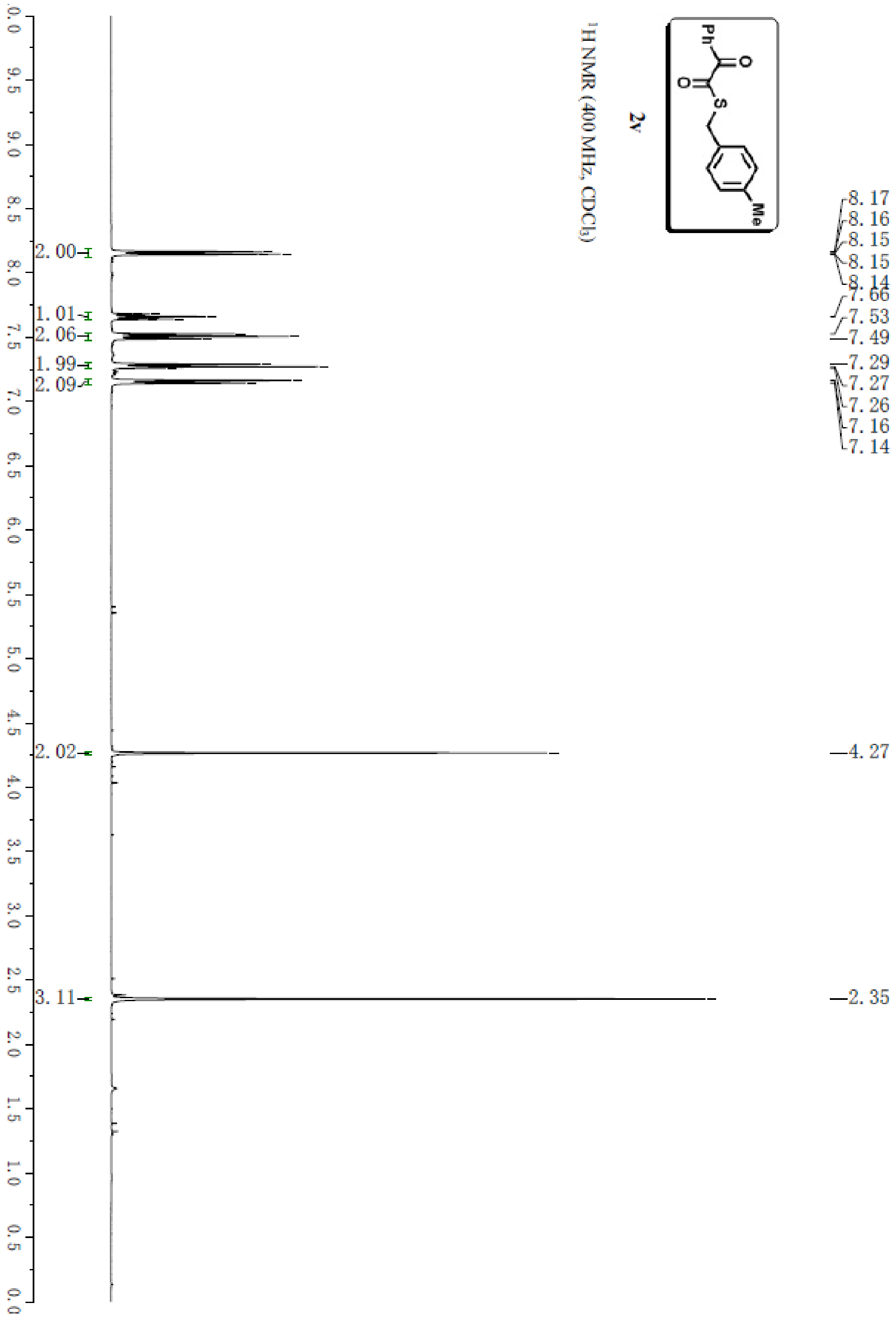

Supplementary Figure 44. ^{1}H NMR (400 MHz, $CDCl_3$) spectra of compound **2v**.

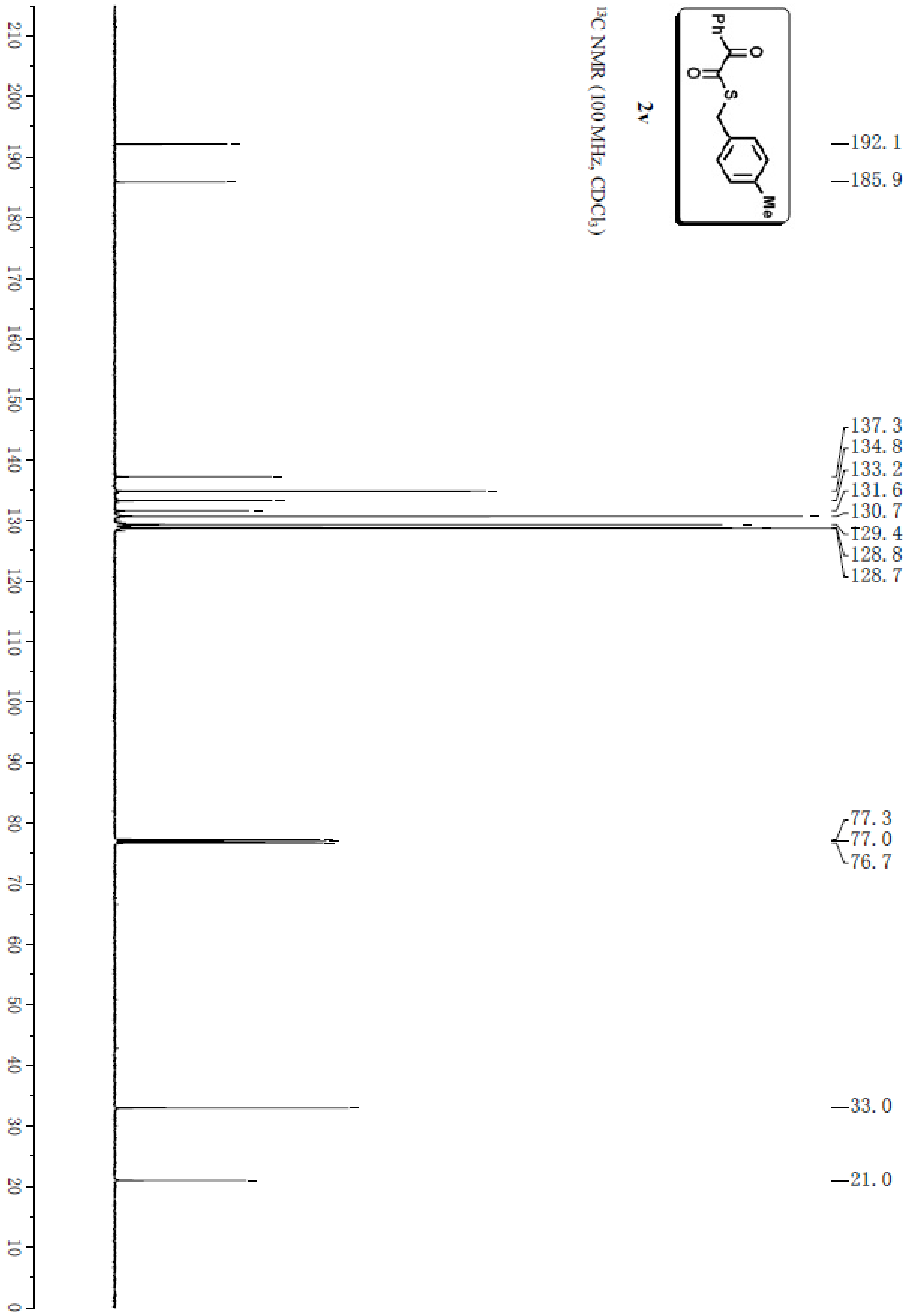

Supplementary Figure 45. ^{13}C NMR (100 MHz, $CDCl_3$) spectra of compound **2v**.

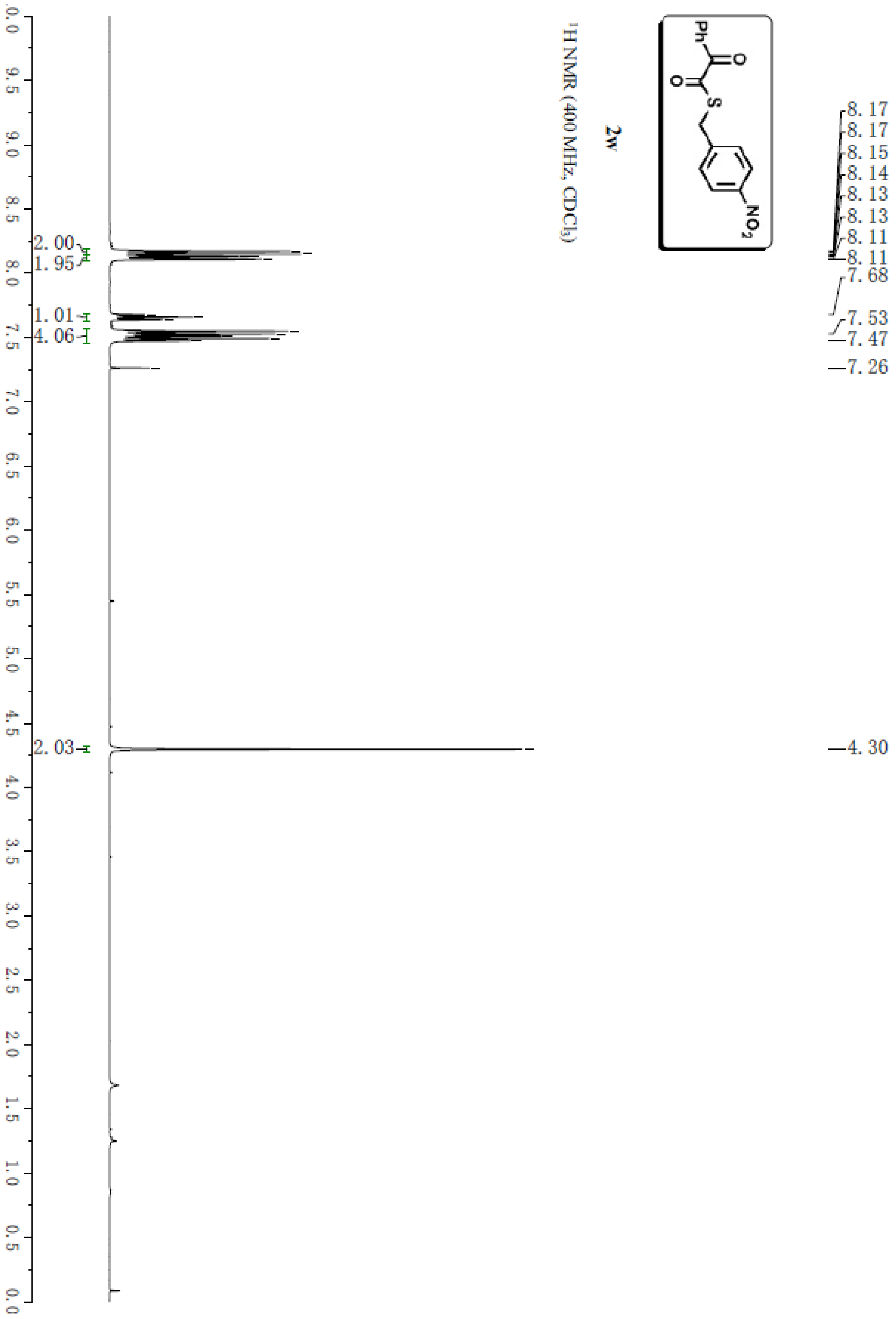

Supplementary Figure 46. ^{1}H NMR (400 MHz, $CDCl_3$) spectra of compound **2w**.

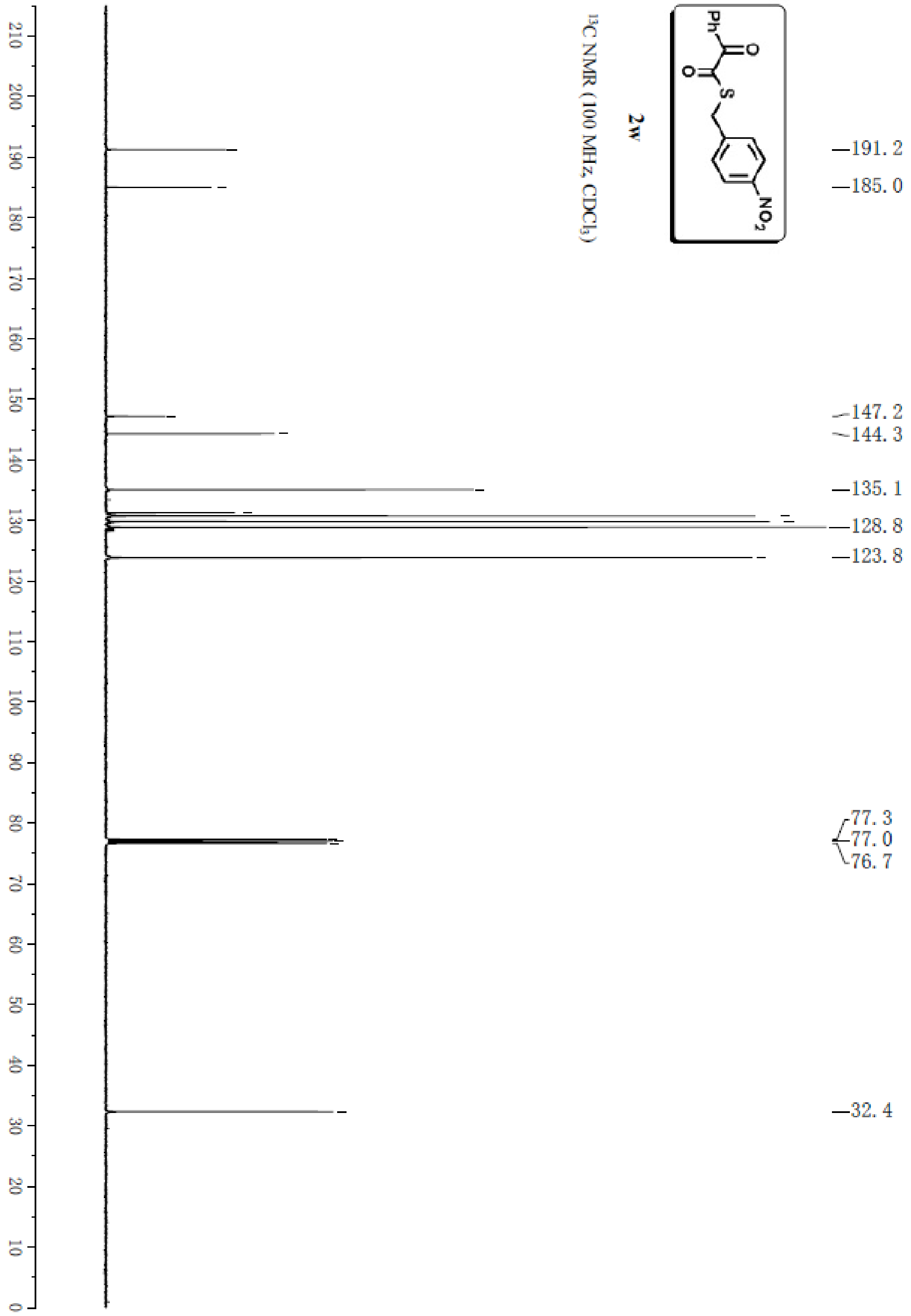

Supplementary Figure 47. ^{13}C NMR (100 MHz, $CDCl_3$) spectra of compound **2w**.

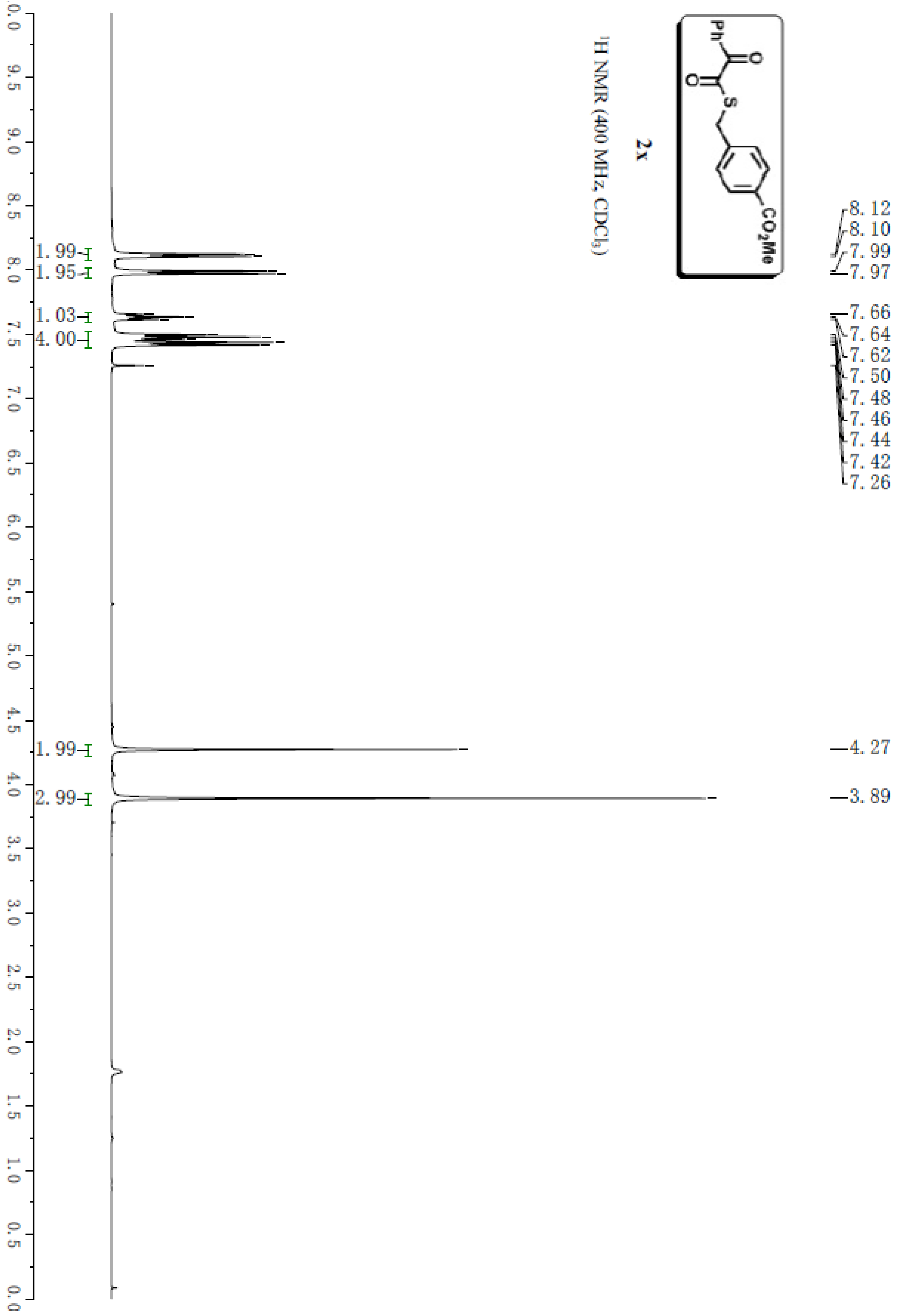

Supplementary Figure 48. ^{1}H NMR (400 MHz, $CDCl_3$) spectra of compound **2x**.

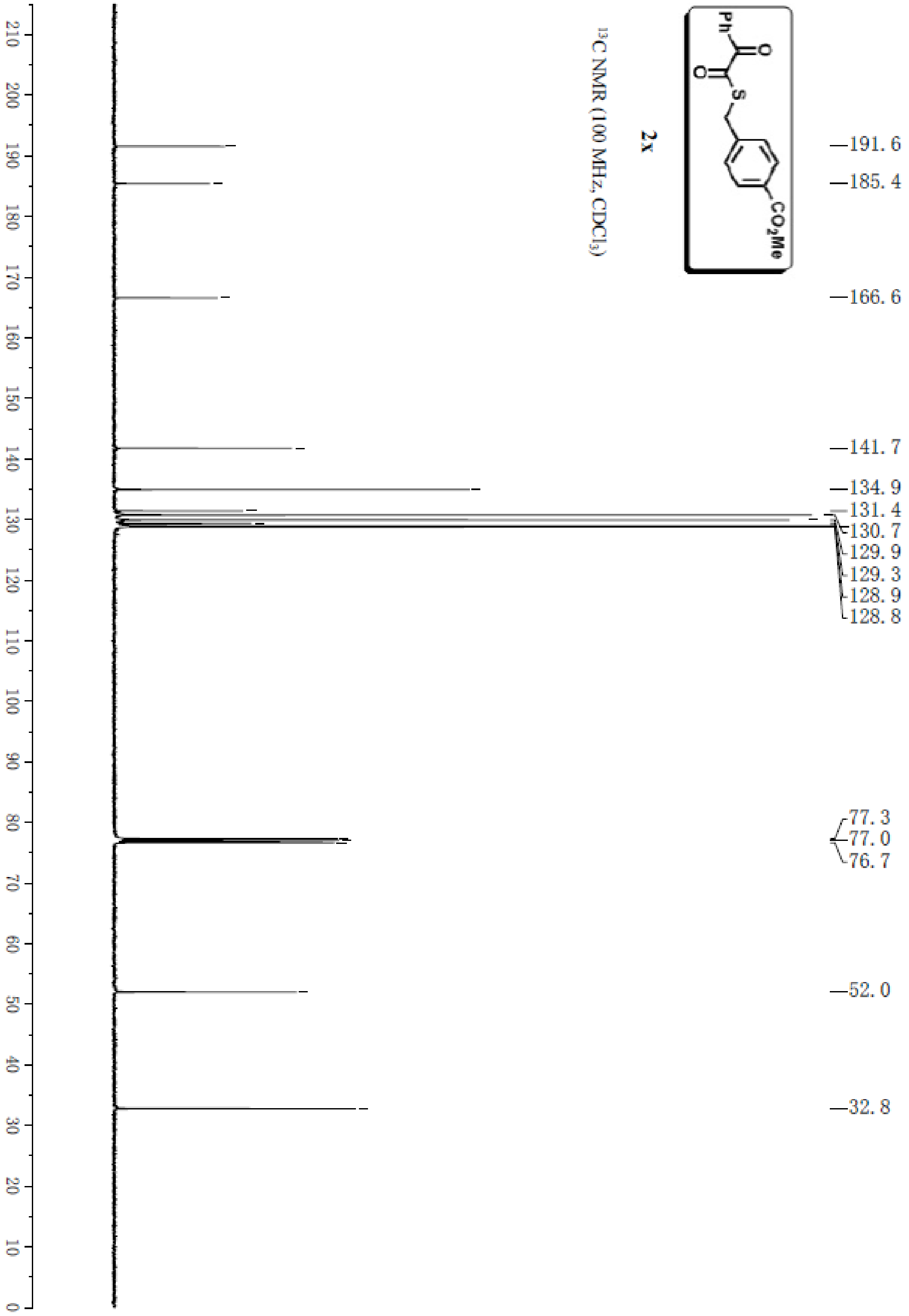

Supplementary Figure 49. ^{13}C NMR (100 MHz, $CDCl_3$) spectra of compound **2x**.

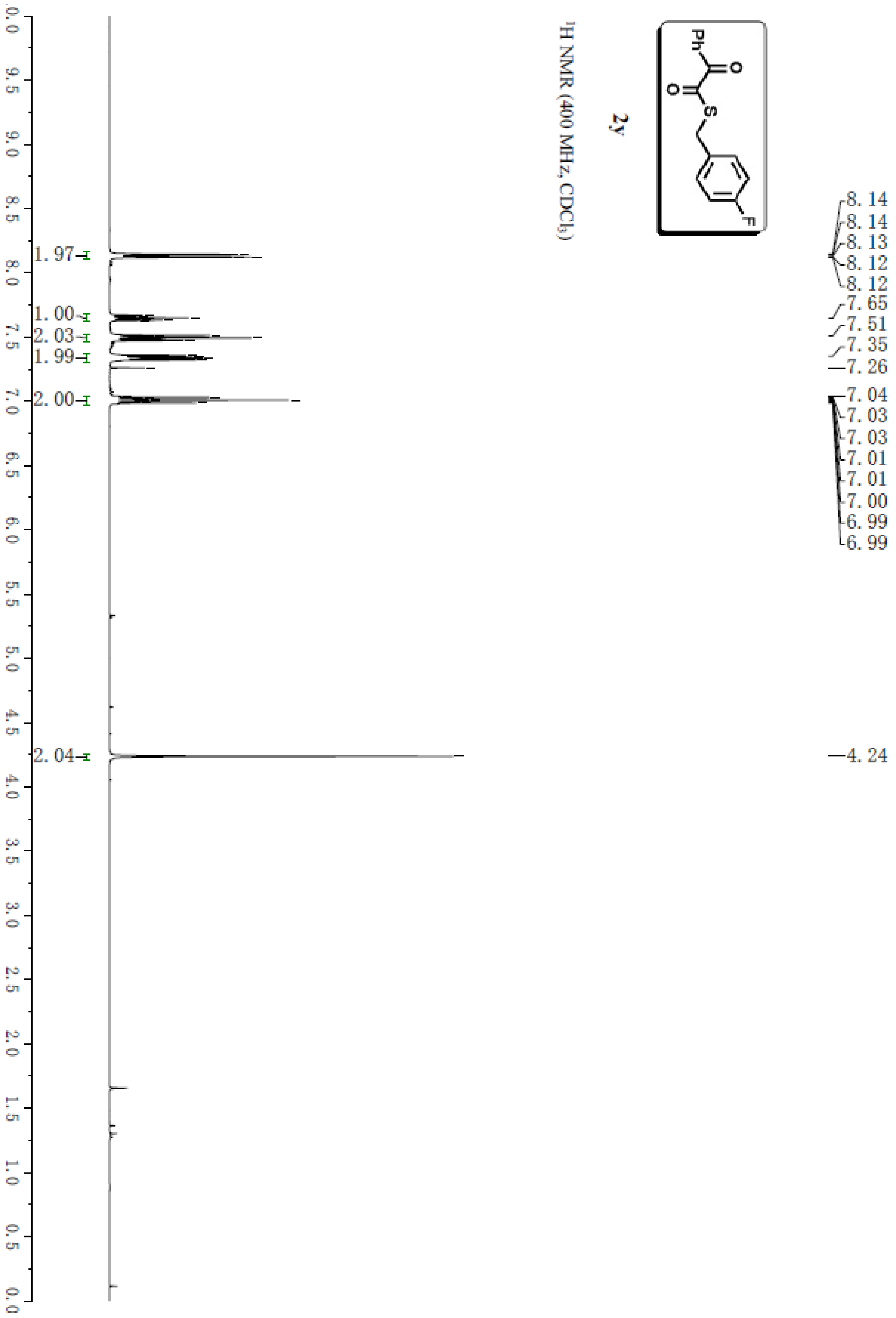

Supplementary Figure 50. ^{1}H NMR (400 MHz, $CDCl_3$) spectra of compound **2y**.

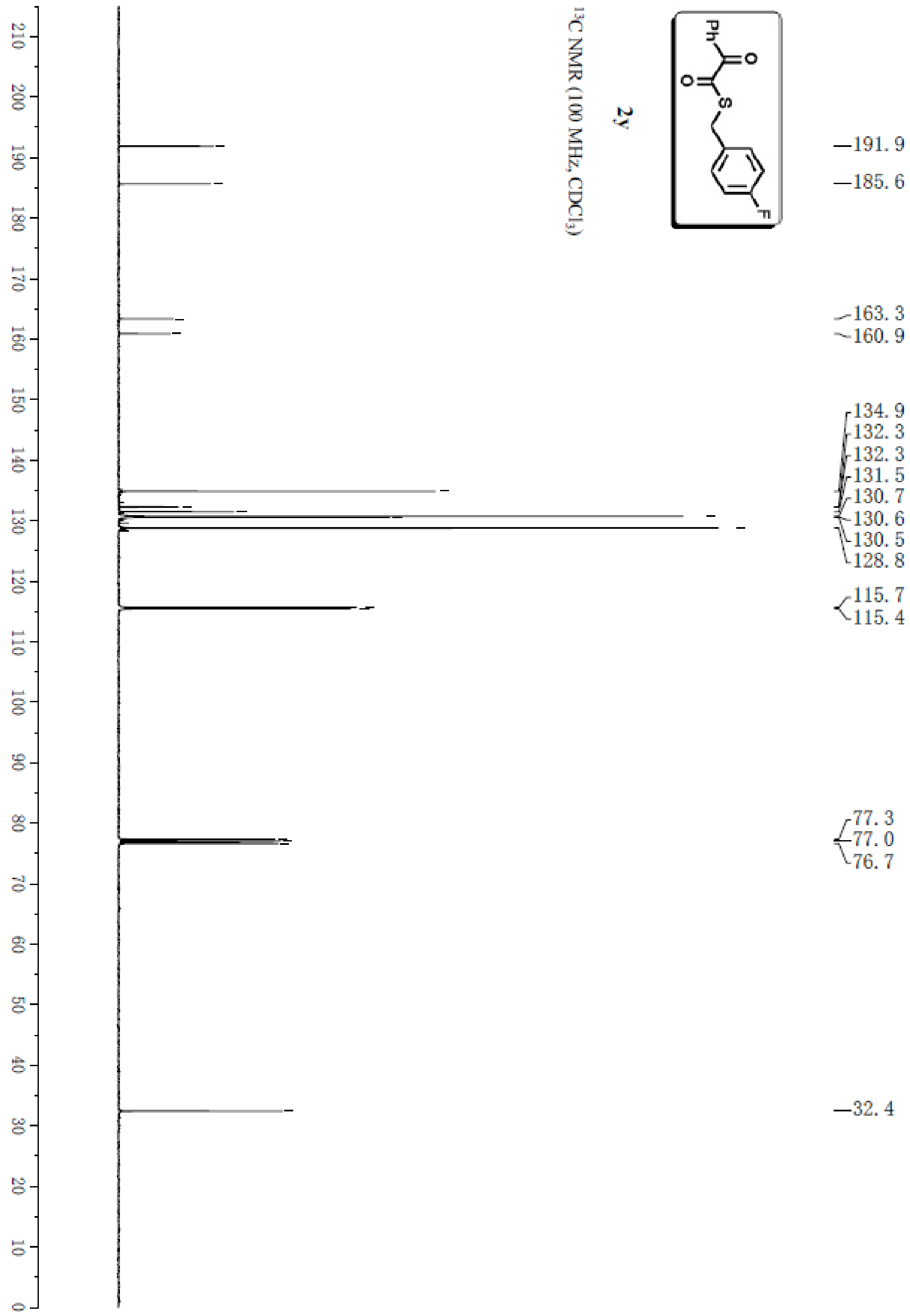

Supplementary Figure 51. ^{13}C NMR (100 MHz, $CDCl_3$) spectra of compound **2y**.

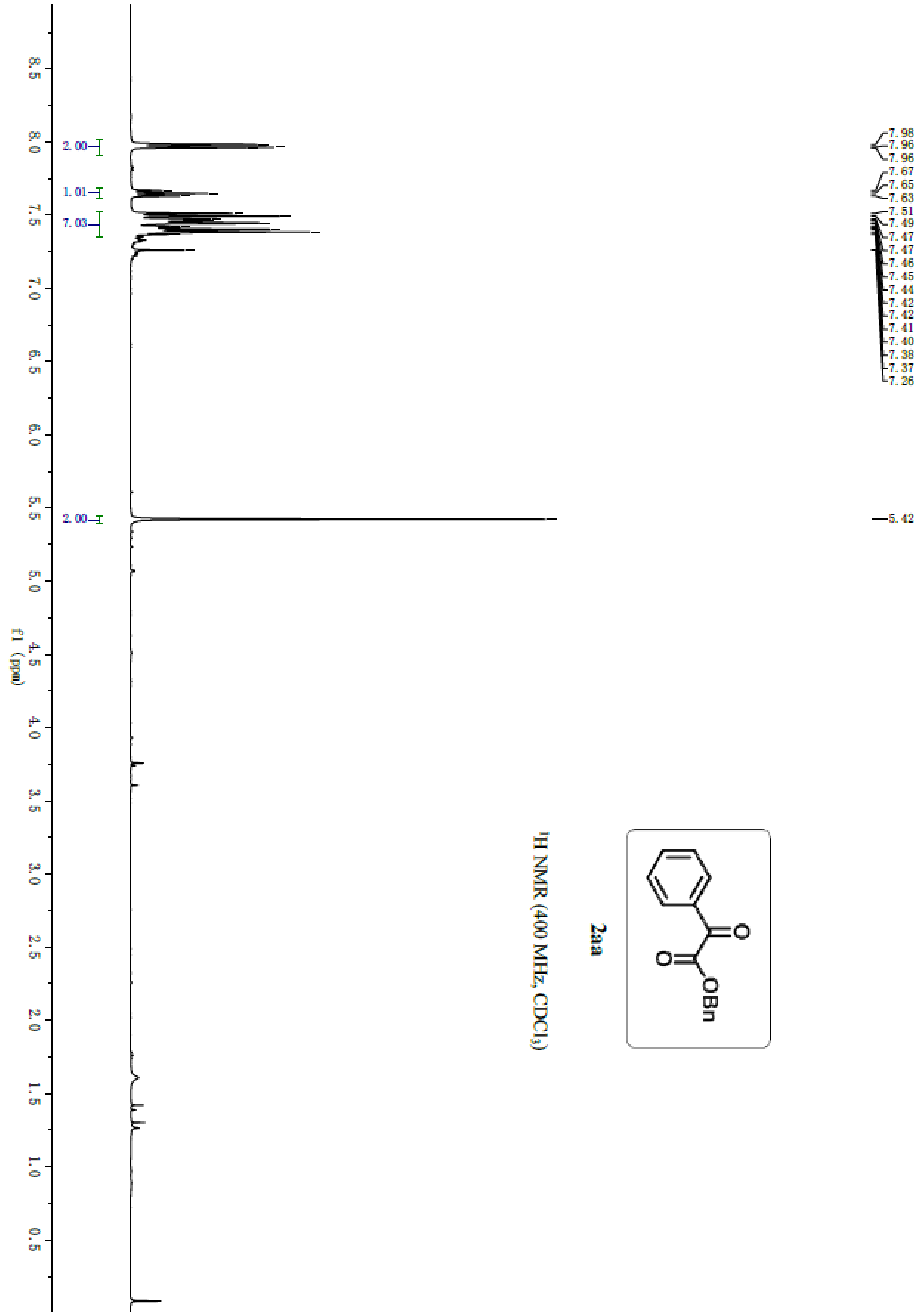

Supplementary Figure 52. ^{1}H NMR (400 MHz, $CDCl_3$) spectra of compound **2aa**.

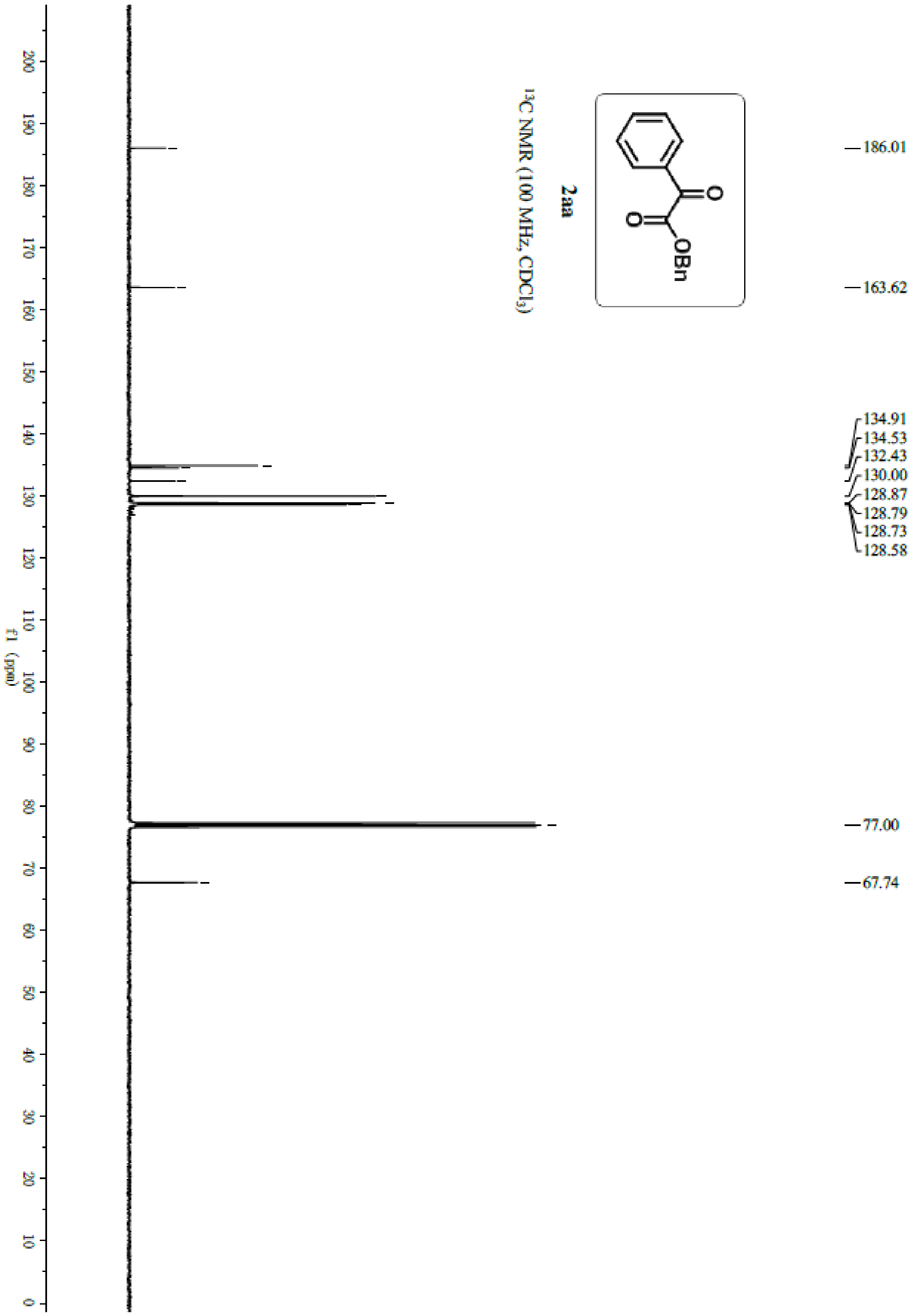

Supplementary Figure 53. ^{13}C NMR (100 MHz, $CDCl_3$) spectra of compound **2aa**.

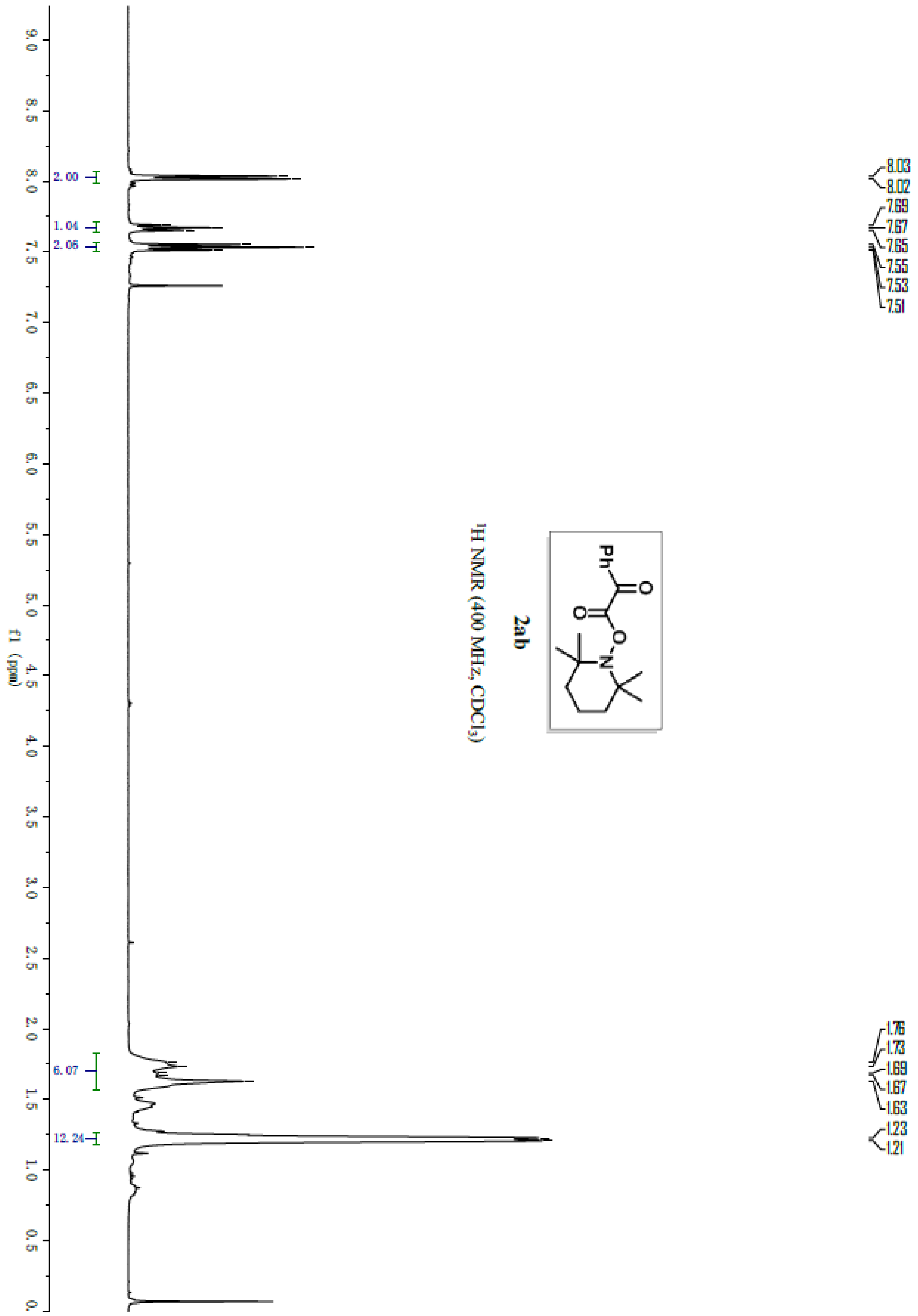

Supplementary Figure 54. ^{1}H NMR (400 MHz, $CDCl_3$) spectra of compound **2ab**.

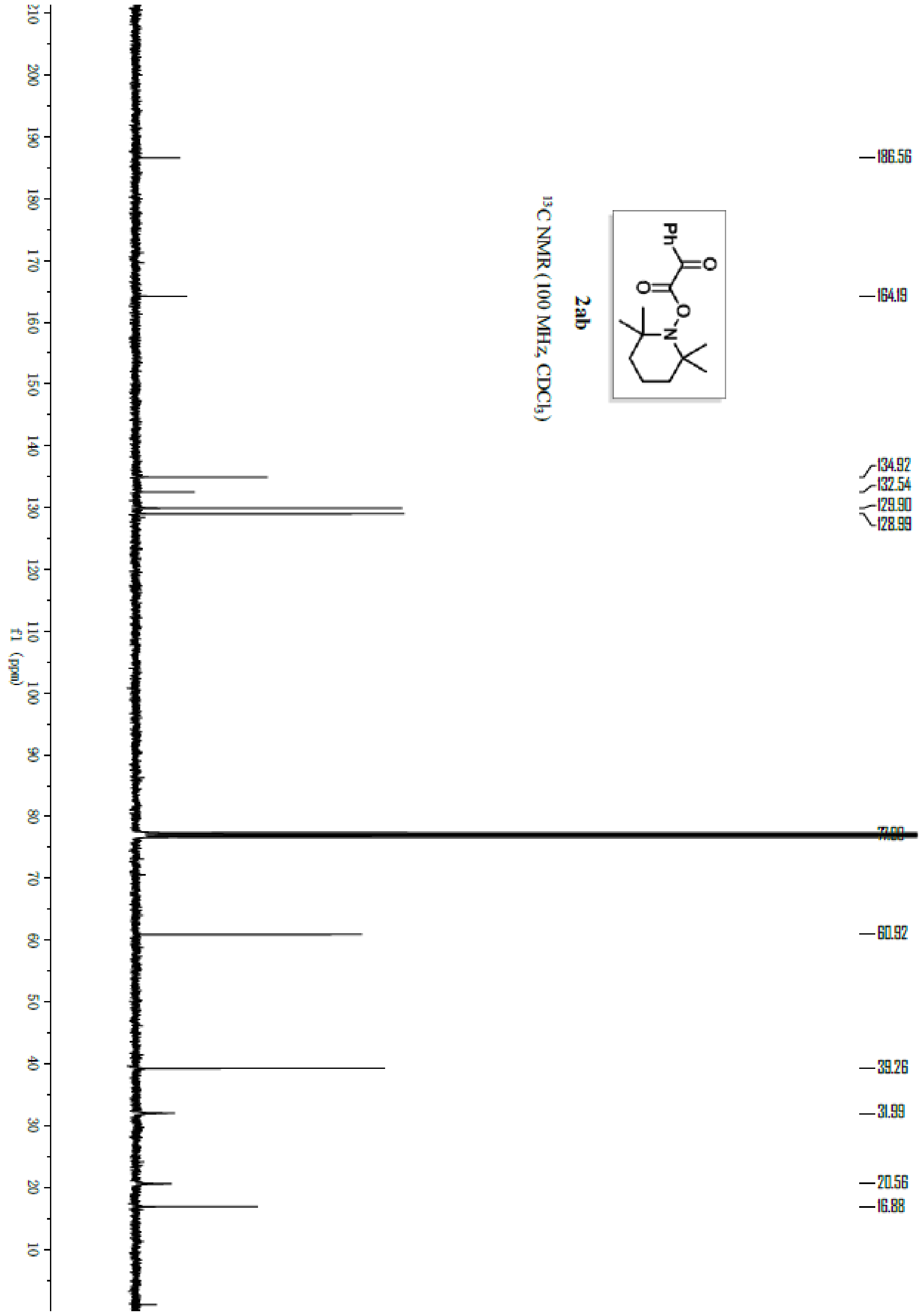

Supplementary Figure 55. ^{13}C NMR (100 MHz, $CDCl_3$) spectra of compound **2ab**.

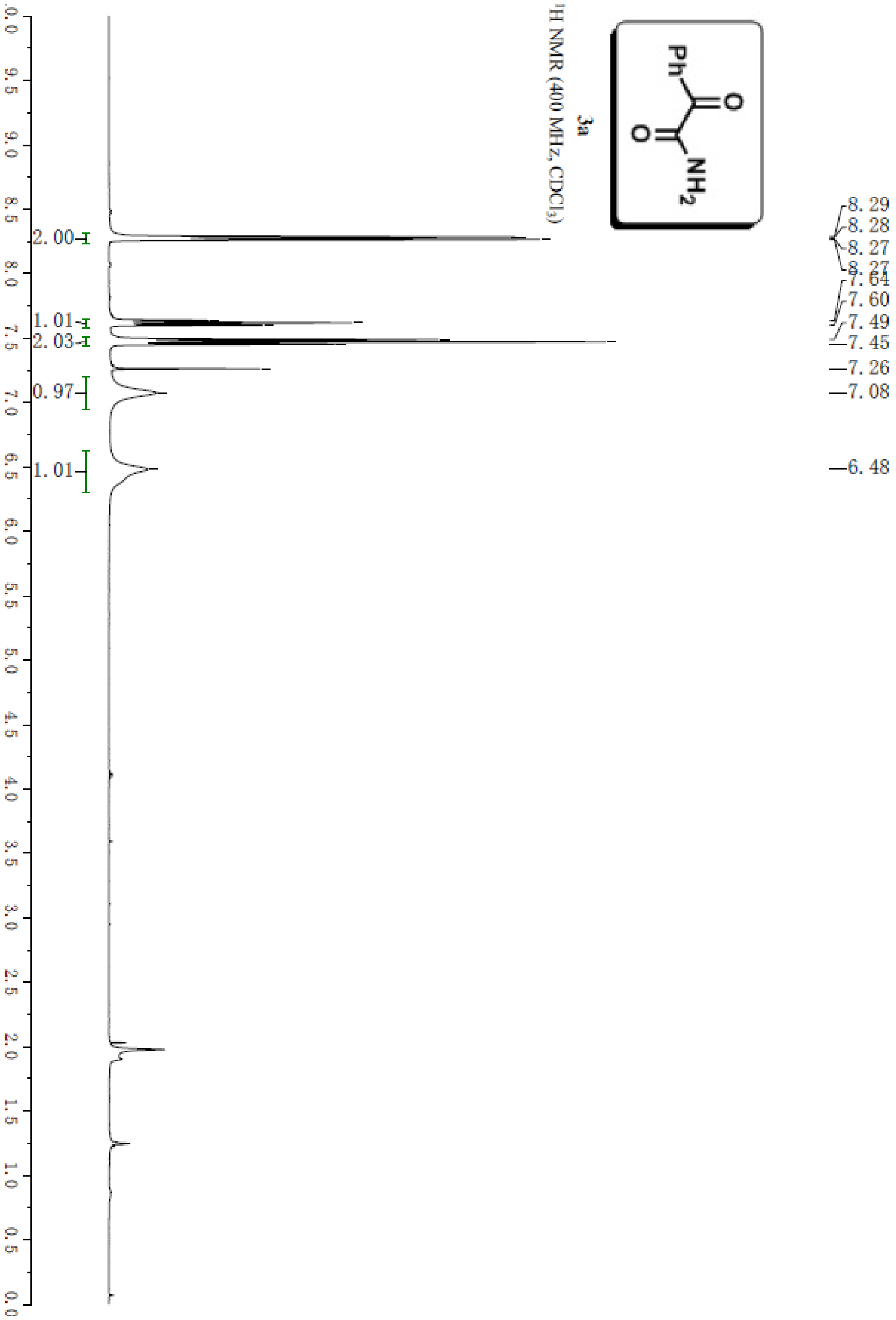

Supplementary Figure 56. ^{1}H NMR (400 MHz, $CDCl_3$) spectra of compound **3a**.

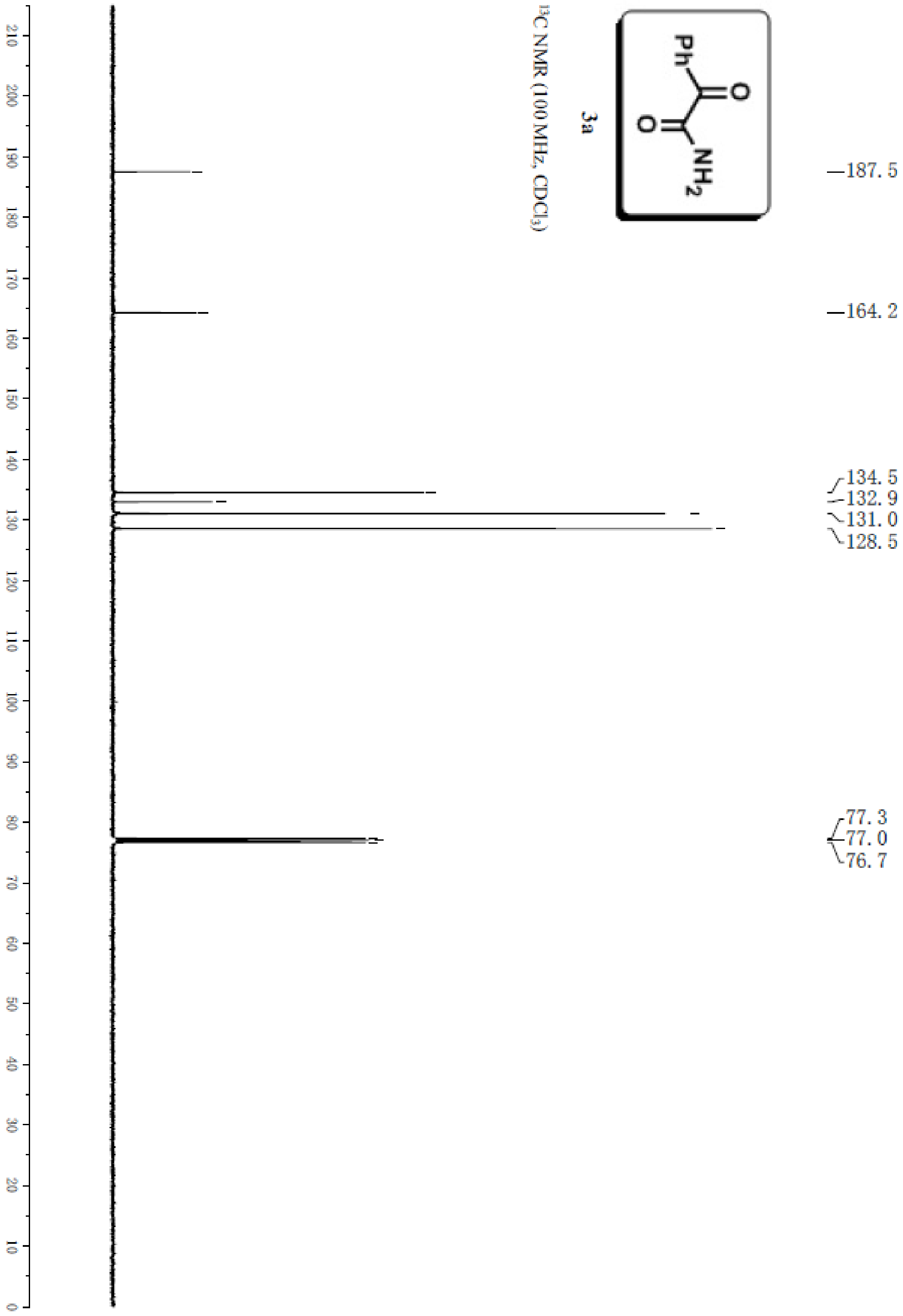

Supplementary Figure 57. ^{13}C NMR (100 MHz, $CDCl_3$) spectra of compound **3a**.

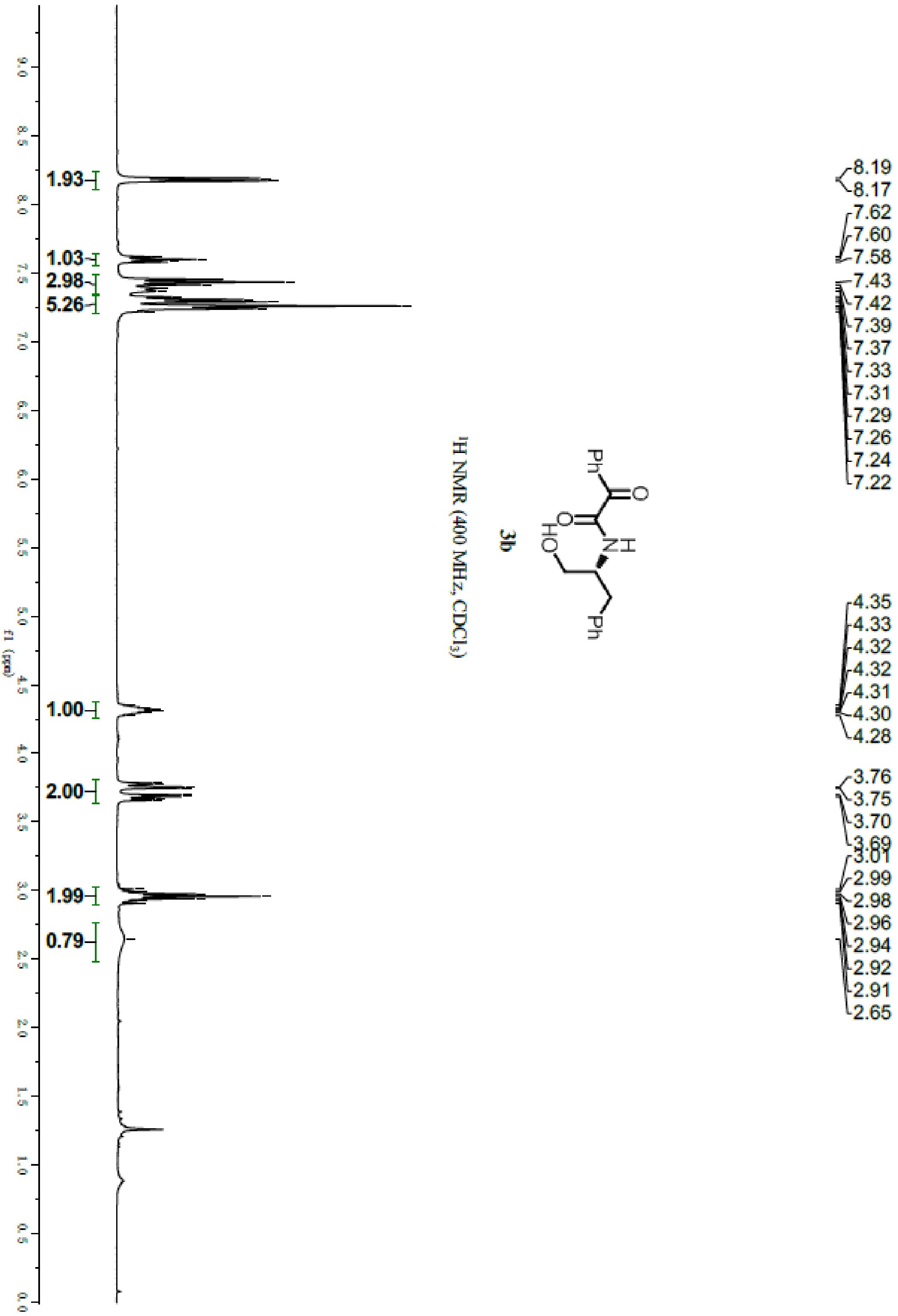

Supplementary Figure 58. ^{1}H NMR (400 MHz, $CDCl_3$) spectra of compound **3b**.

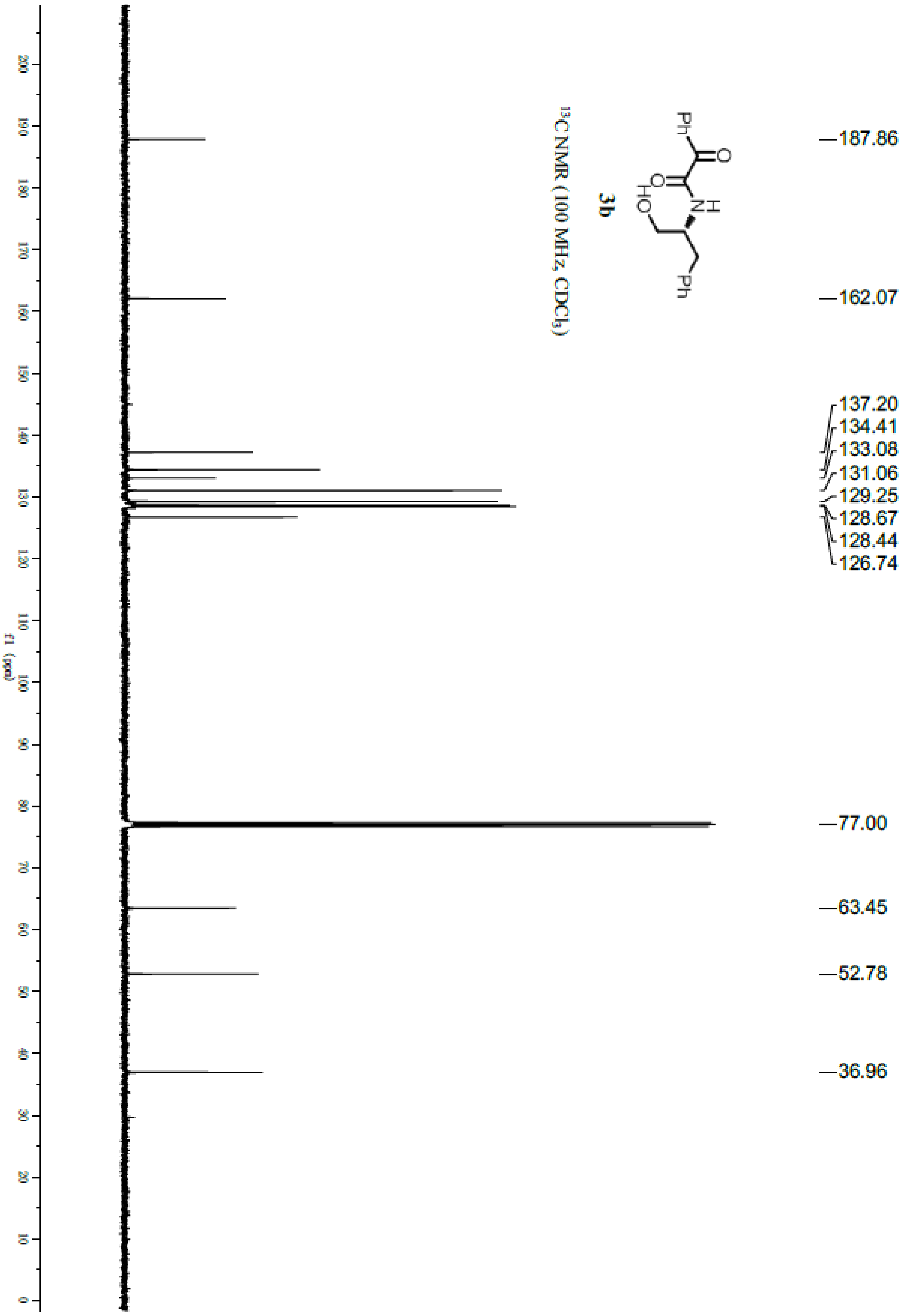

Supplementary Figure 59. ^{13}C NMR (100 MHz, $CDCl_3$) spectra of compound **3b**.

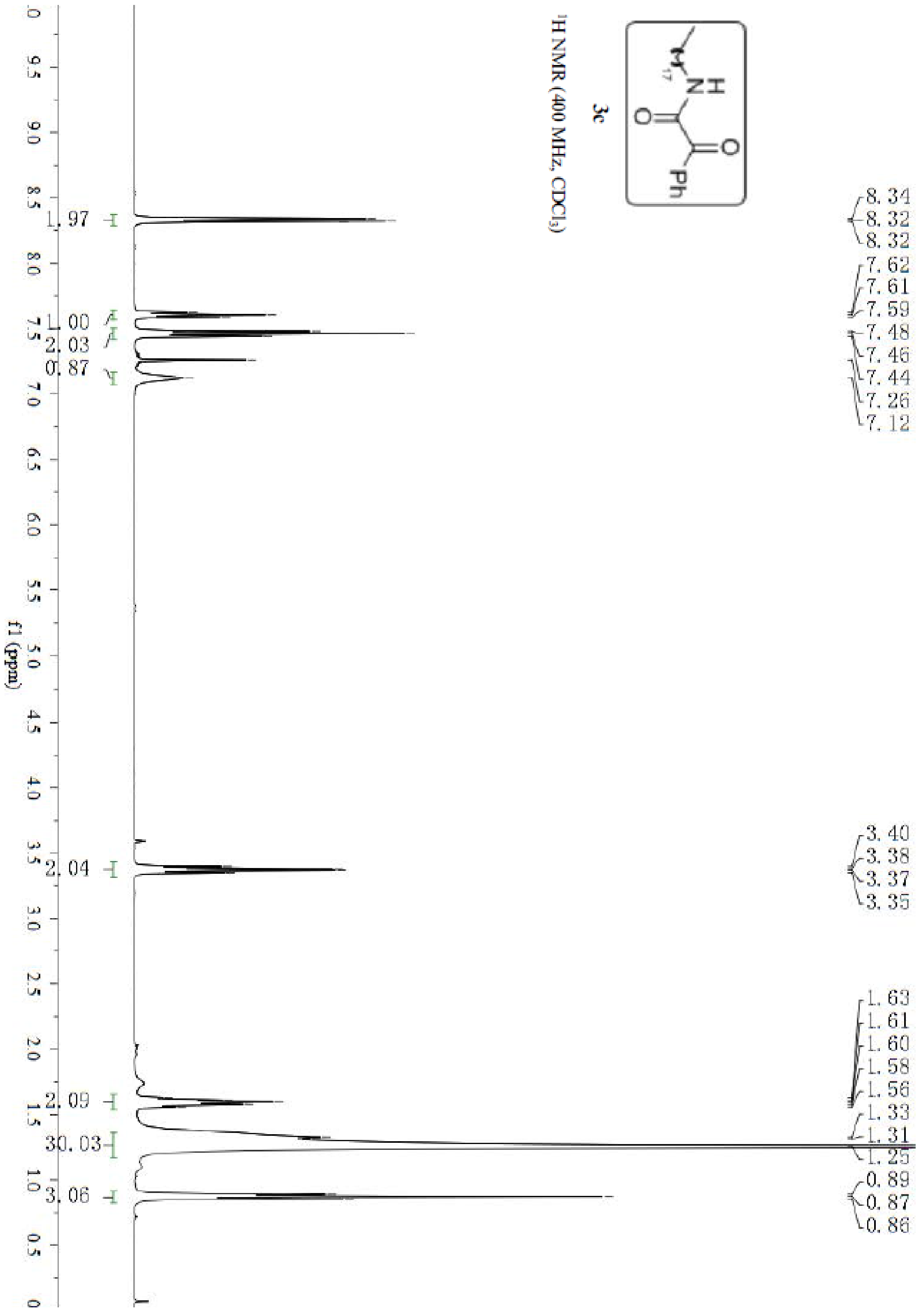

Supplementary Figure 60. ^{1}H NMR (400 MHz, $CDCl_3$) spectra of compound **3c**.

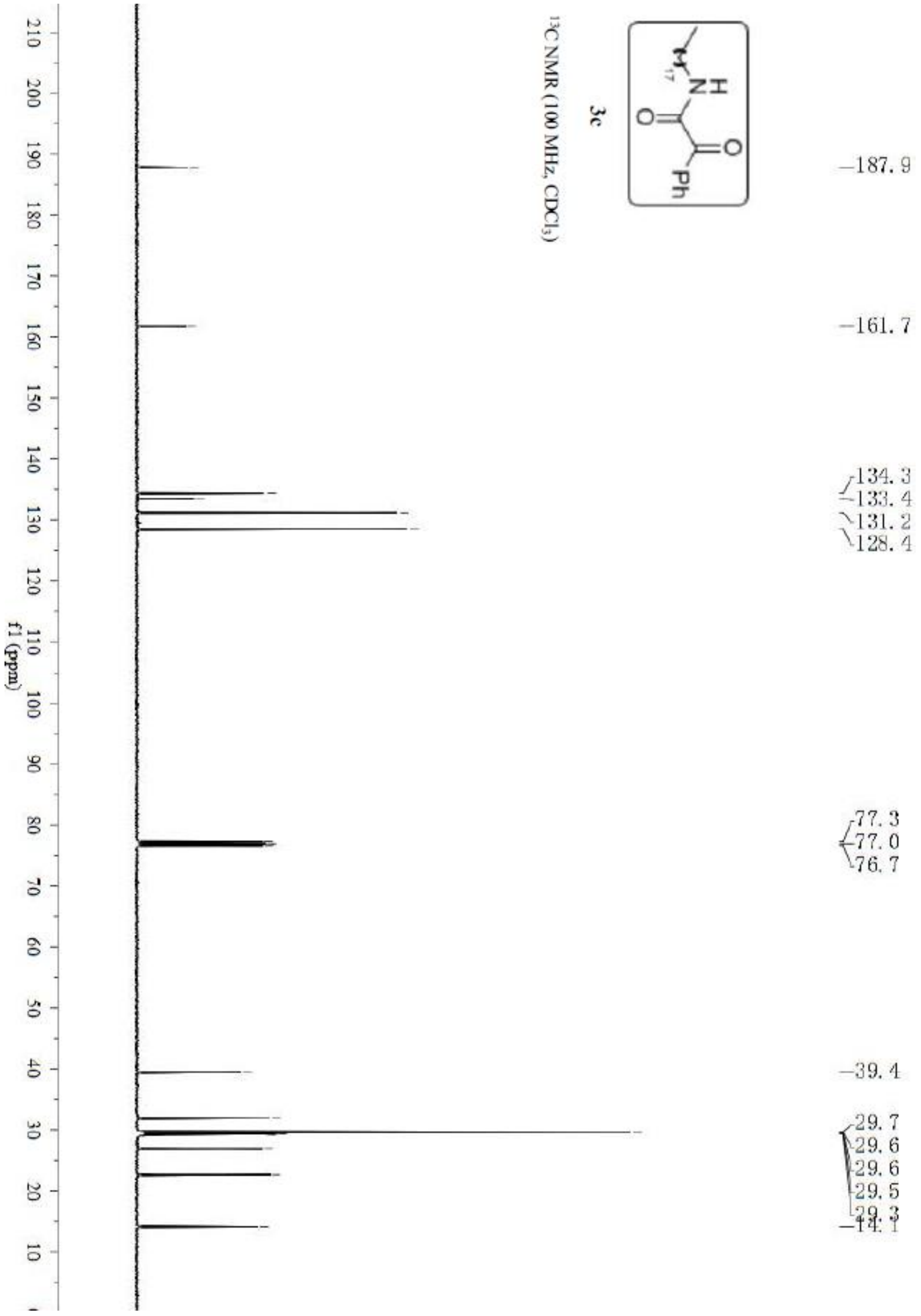

Supplementary Figure 61. ^{13}C NMR (100 MHz, $CDCl_3$) spectra of compound **3c**.

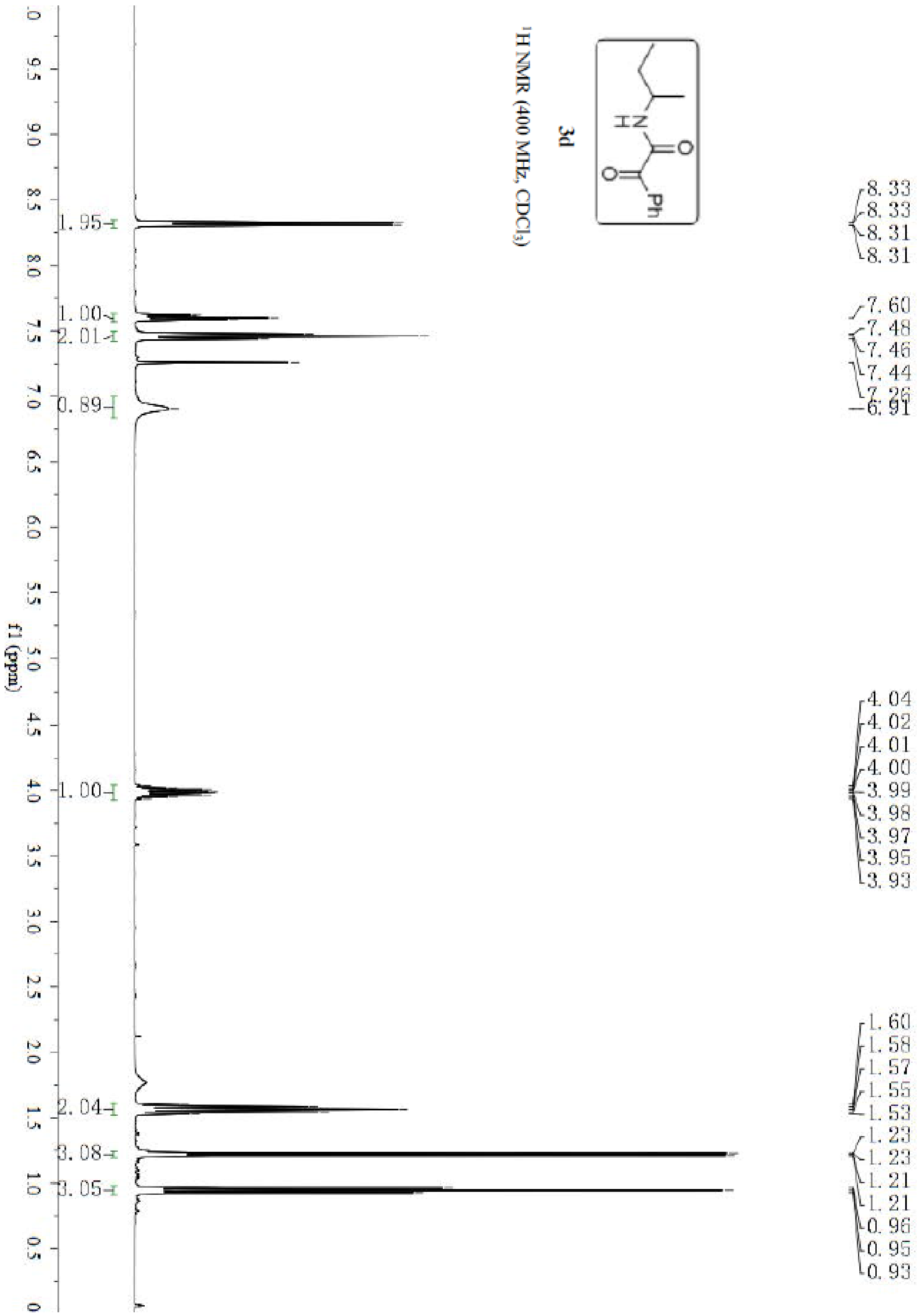

Supplementary Figure 62. ^{1}H NMR (400 MHz, $CDCl_3$) spectra of compound **3d**.

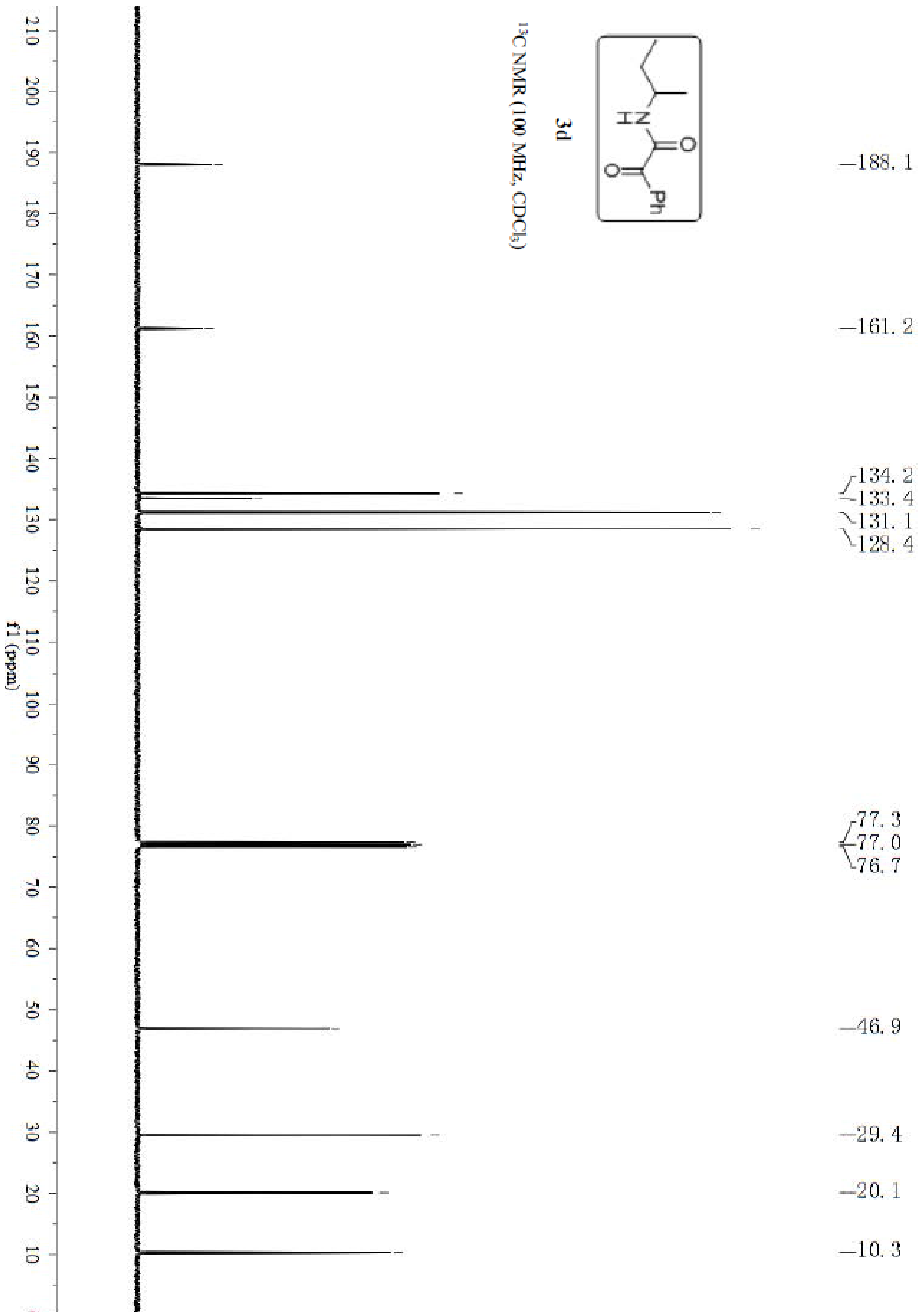

Supplementary Figure 63. ^{13}C NMR (100 MHz, $CDCl_3$) spectra of compound **3d**.

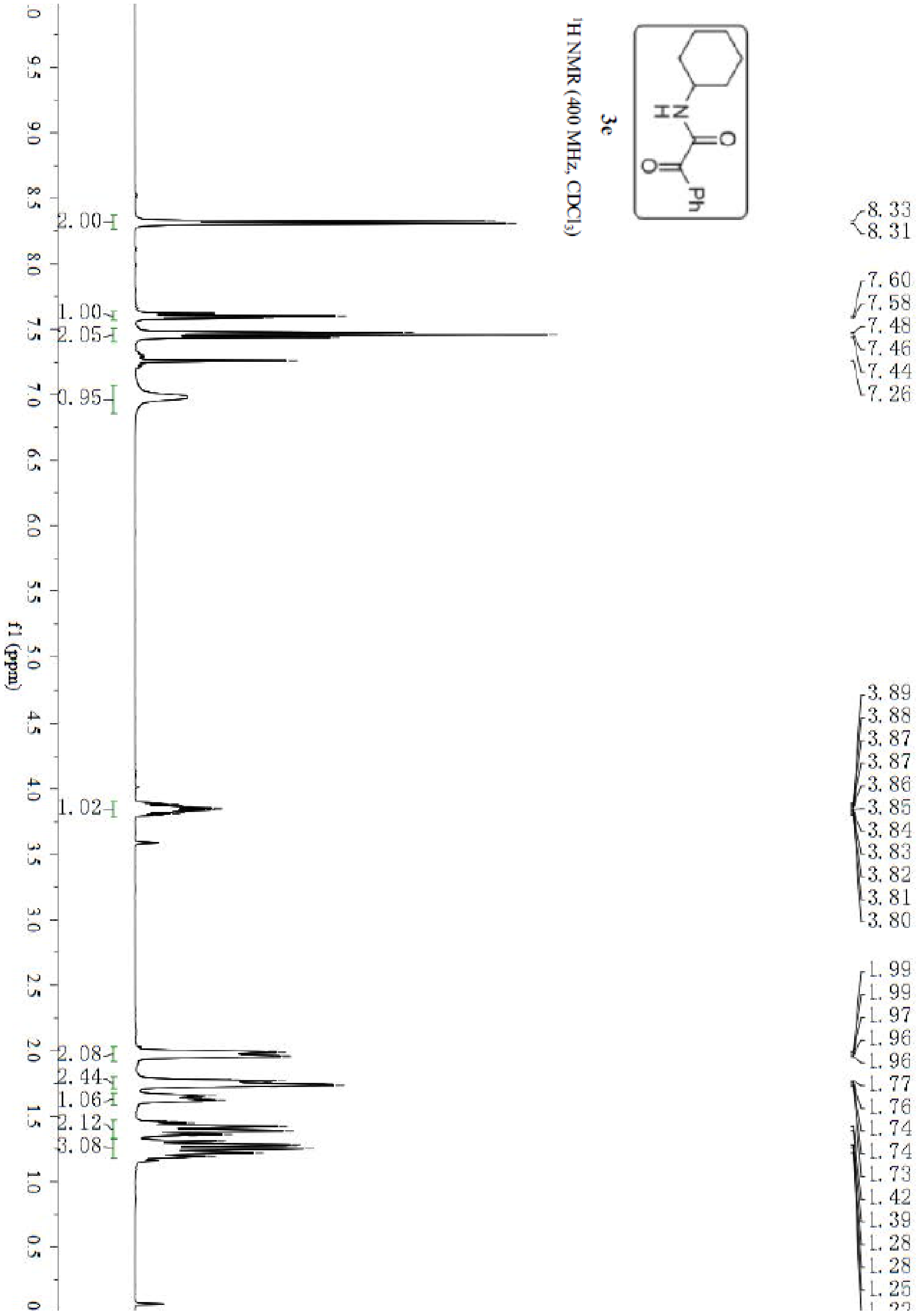

Supplementary Figure 64. ^{1}H NMR (400 MHz, $CDCl_3$) spectra of compound **3e**.

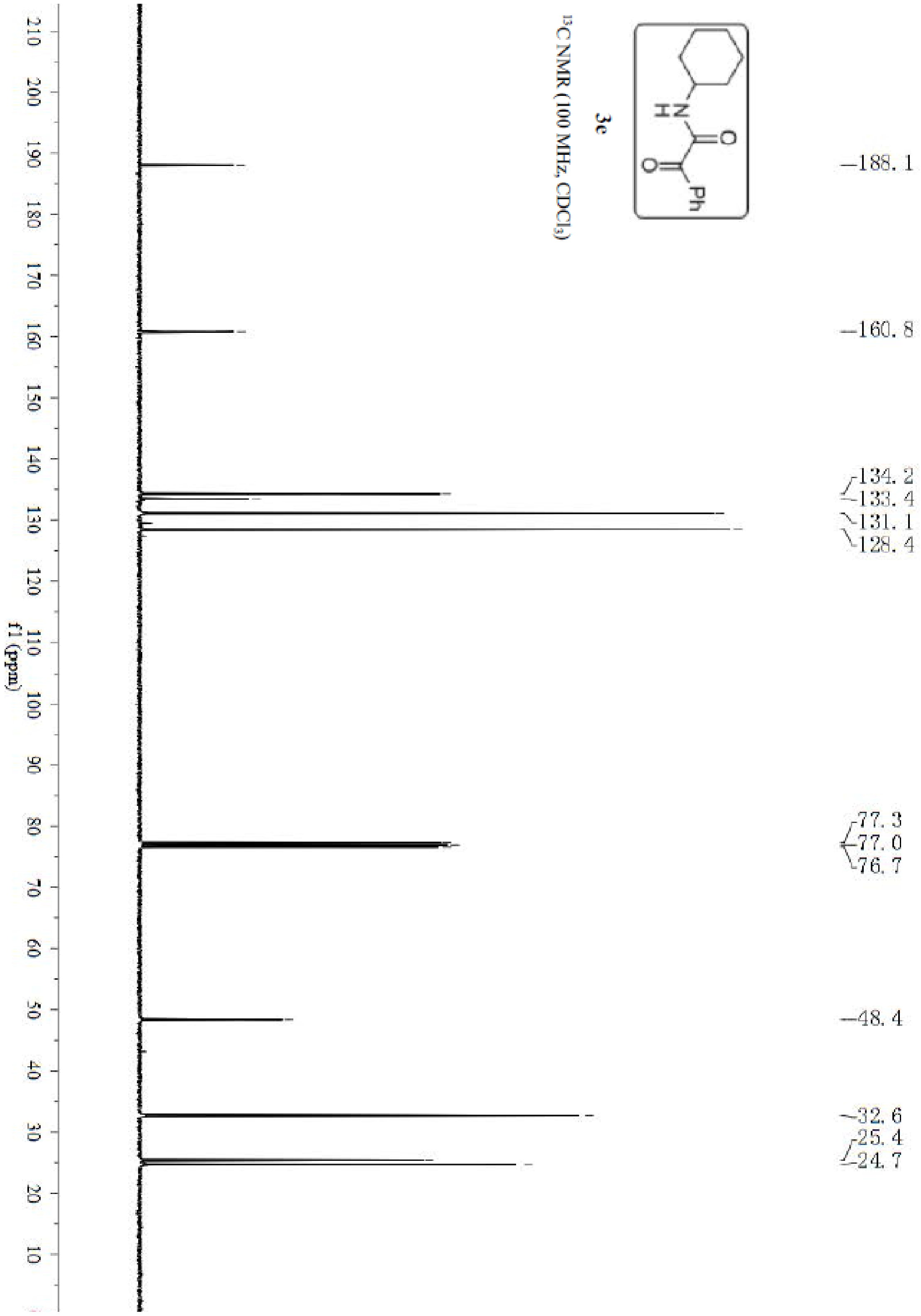

Supplementary Figure 65. ^{13}C NMR (100 MHz, $CDCl_3$) spectra of compound **3e**.

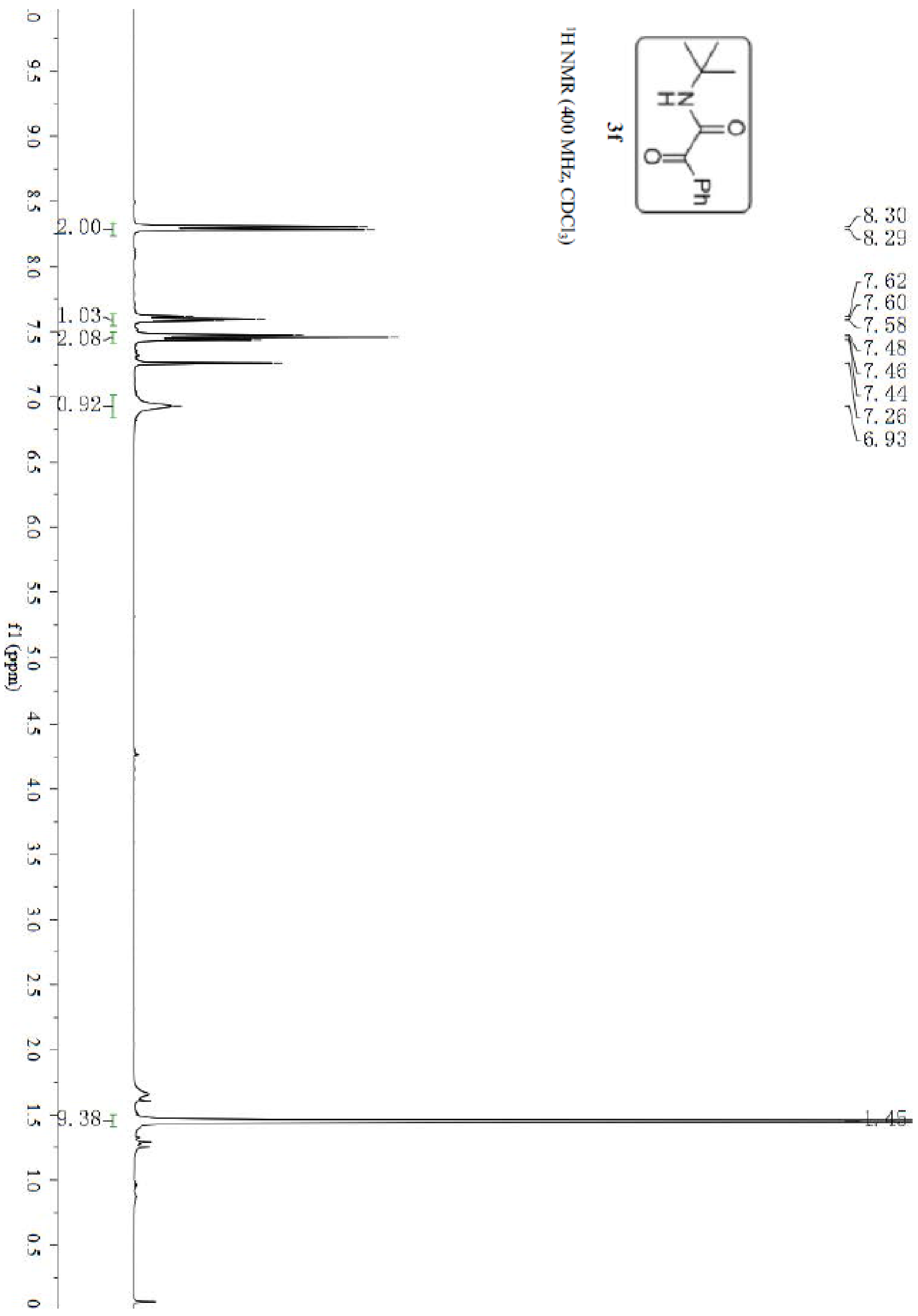

Supplementary Figure 66. ^{1}H NMR (400 MHz, $CDCl_3$) spectra of compound **3f**.

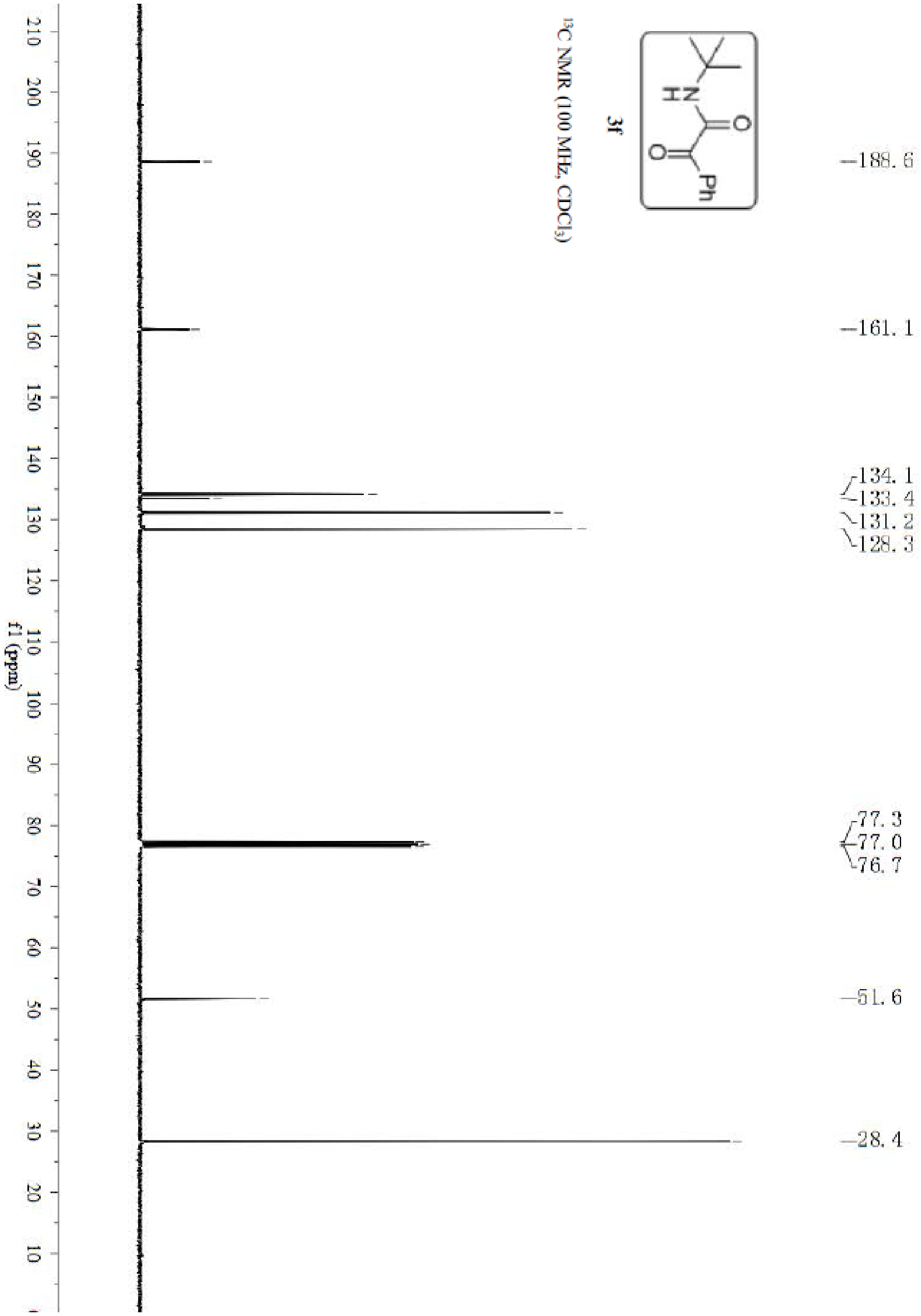

Supplementary Figure 67. ^{13}C NMR (100 MHz, $CDCl_3$) spectra of compound **3f**.

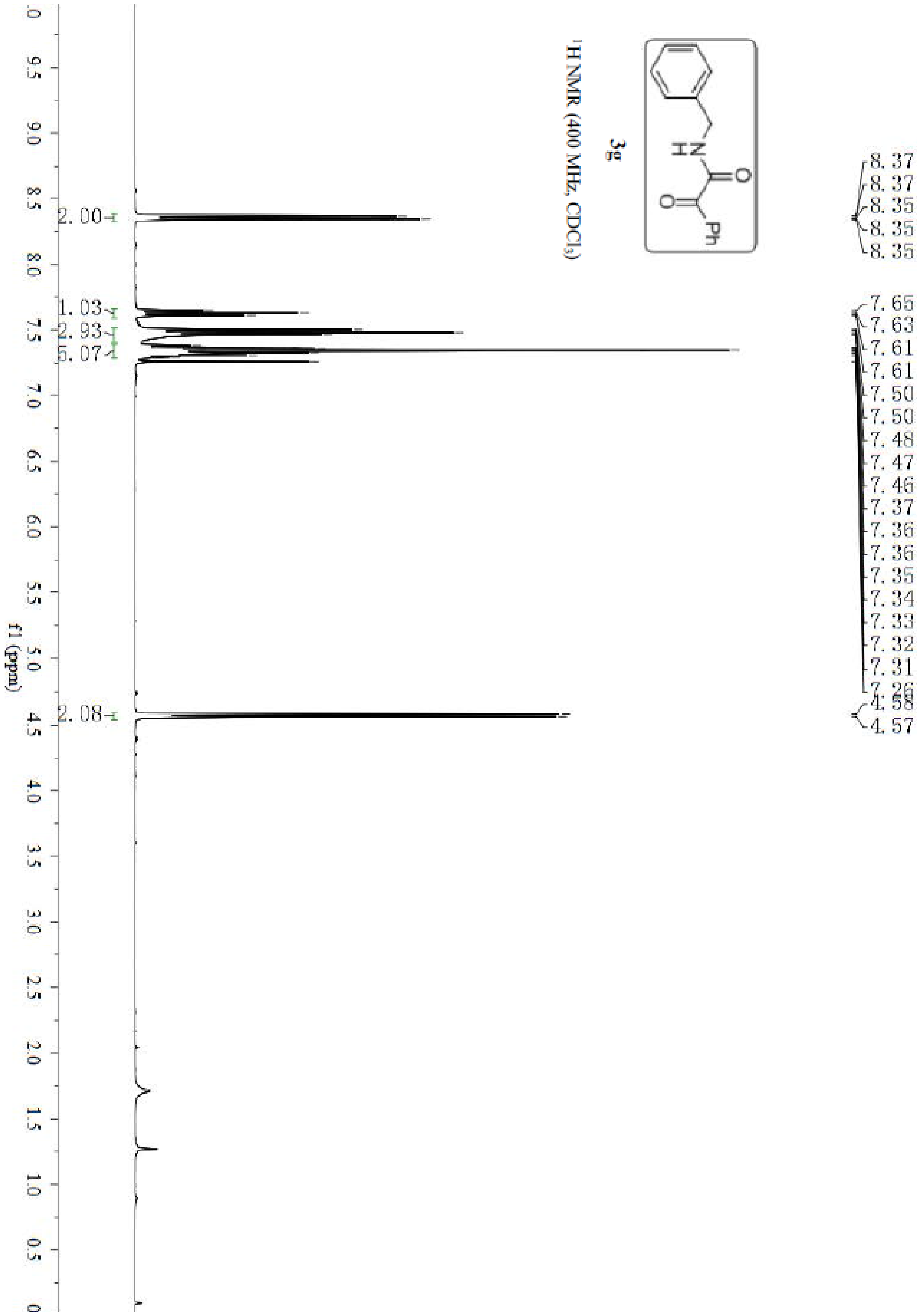

Supplementary Figure 68. ^{1}H NMR (400 MHz, $CDCl_3$) spectra of compound **3g**.

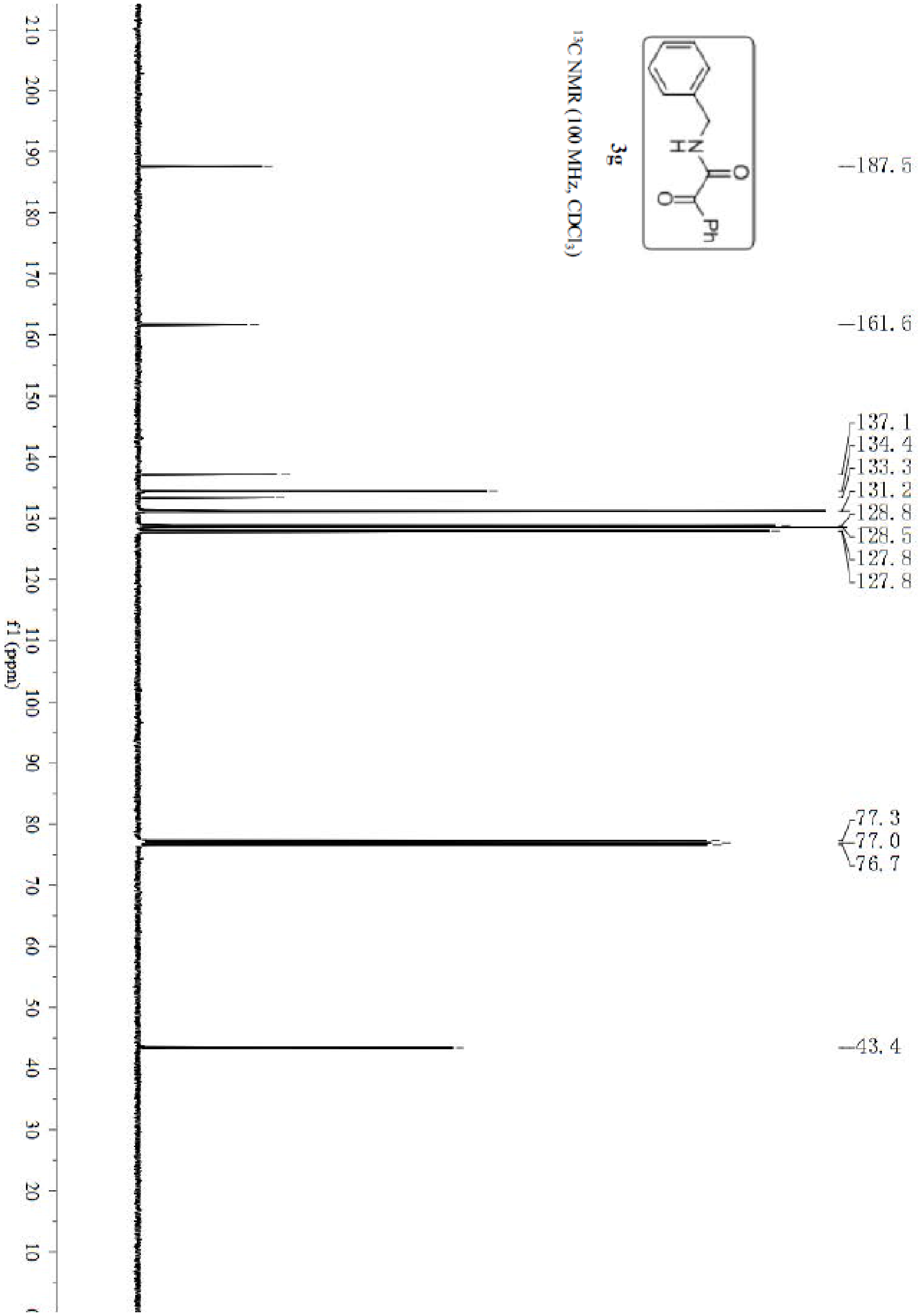

Supplementary Figure 69. ^{13}C NMR (100 MHz, $CDCl_3$) spectra of compound **3g**.

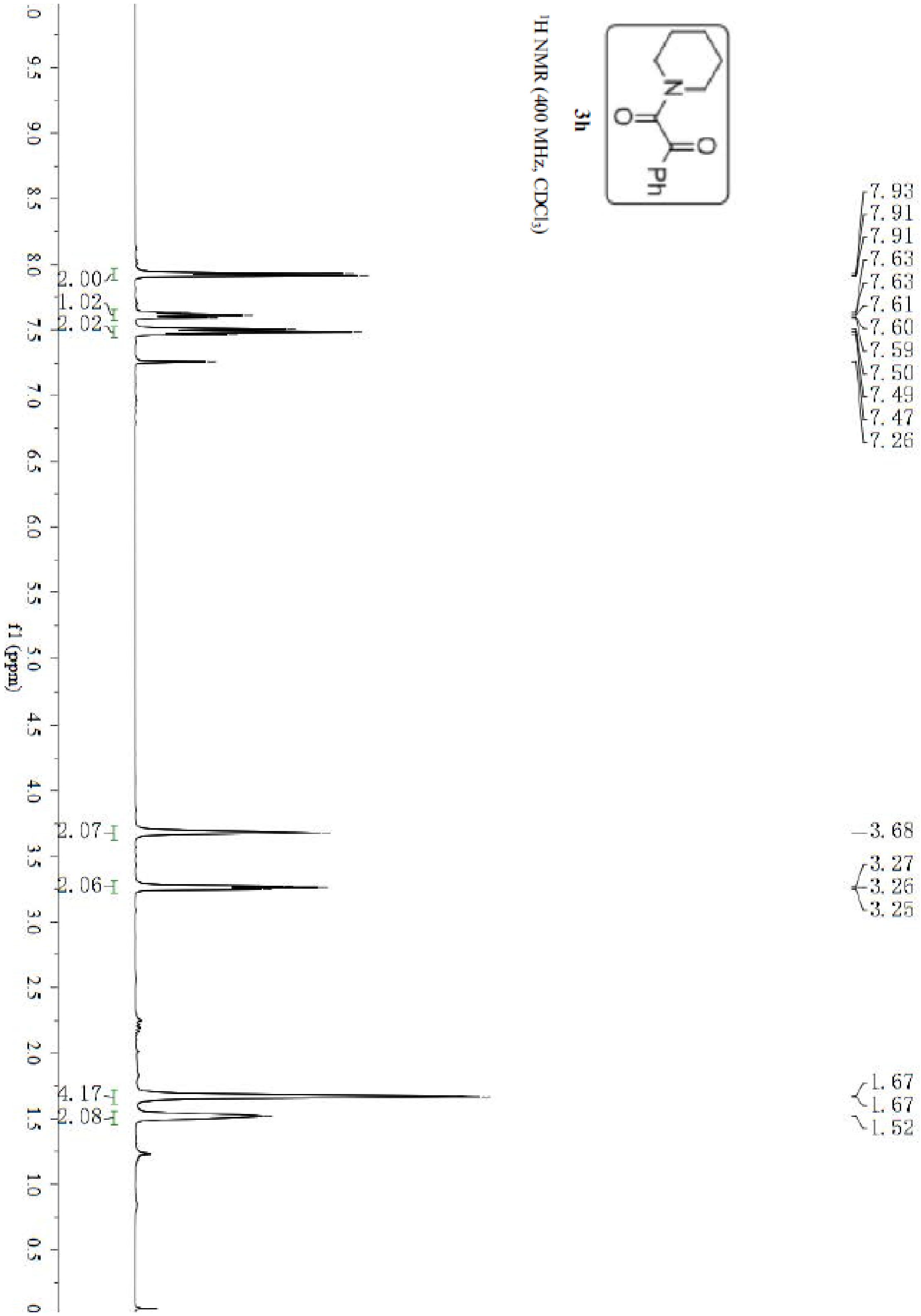

Supplementary Figure 70. ^{1}H NMR (400 MHz, $CDCl_3$) spectra of compound **3h**.

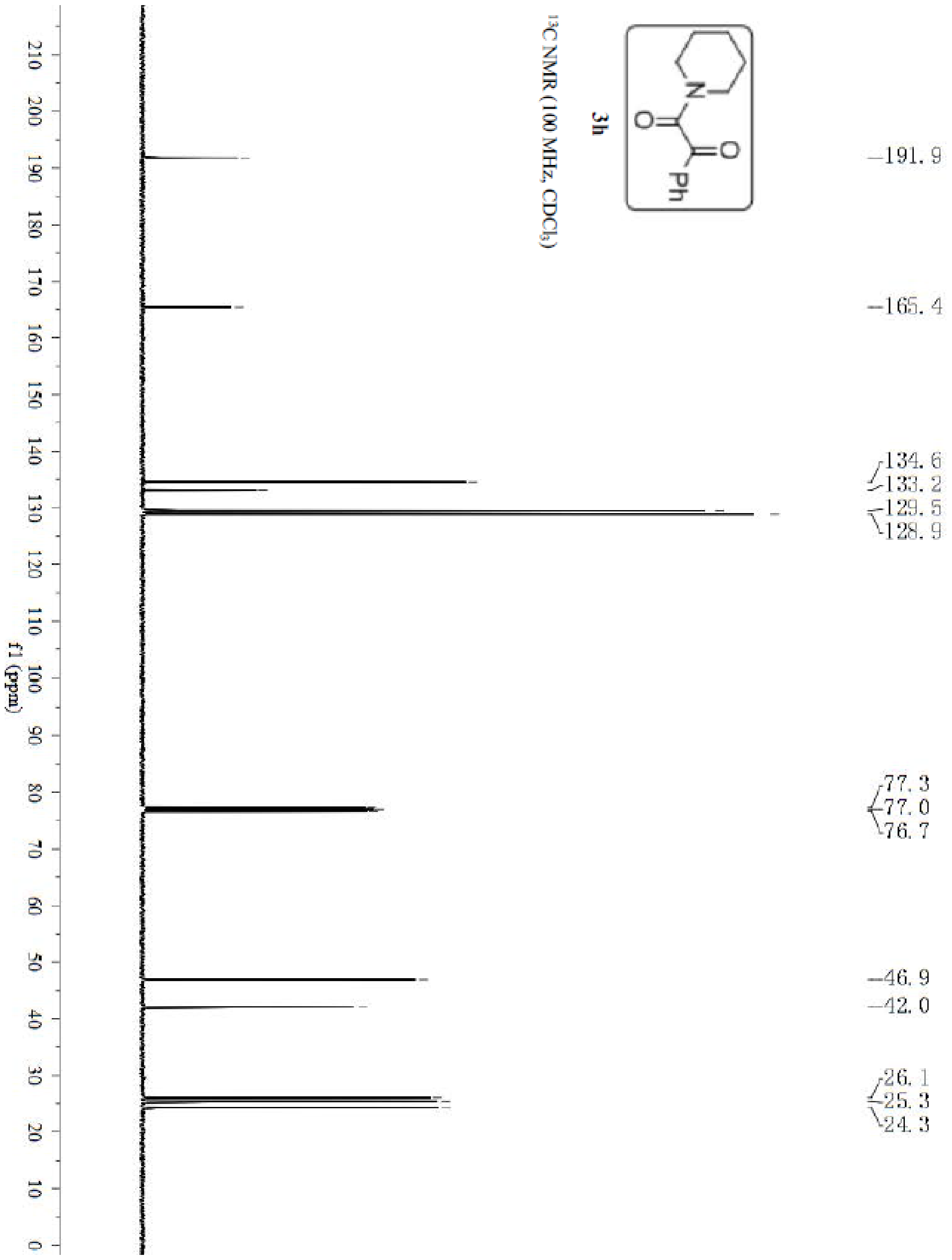

Supplementary Figure 71. ^{13}C NMR (100 MHz, $CDCl_3$) spectra of compound **3h**.

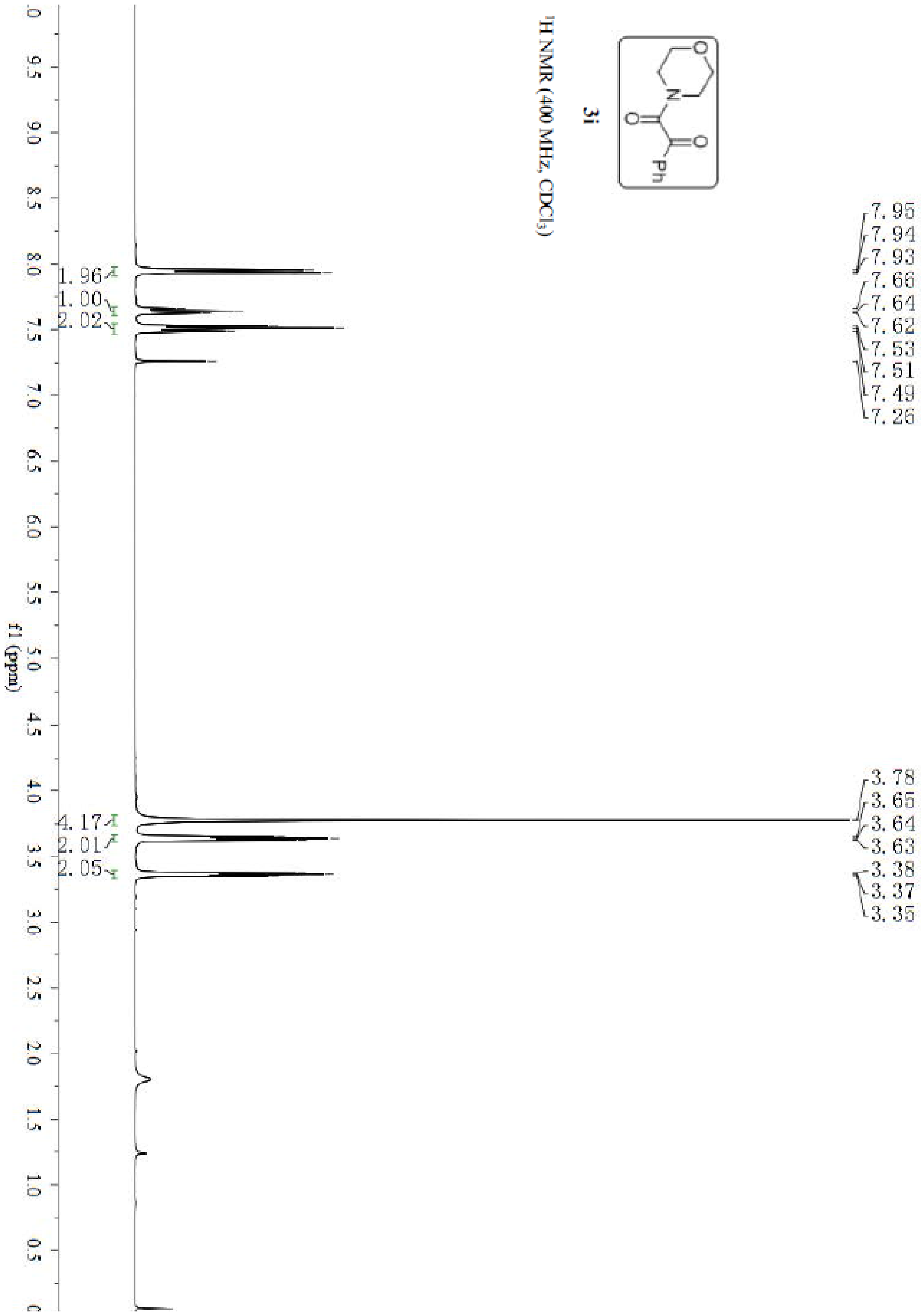

Supplementary Figure 72. ^{1}H NMR (400 MHz, $CDCl_3$) spectra of compound **3i**.

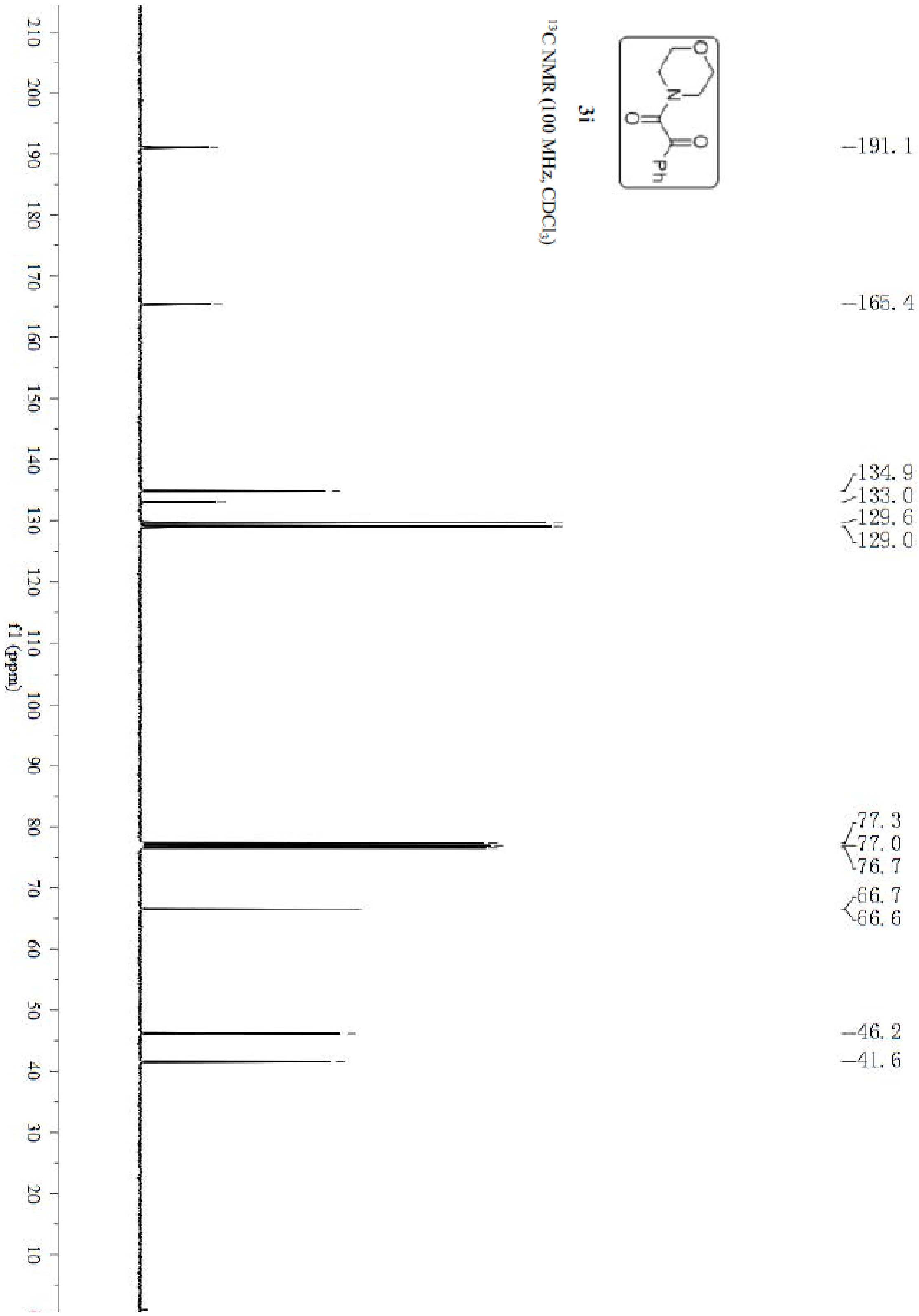

Supplementary Figure 73. ^{13}C NMR (100 MHz, $CDCl_3$) spectra of compound **3i**.

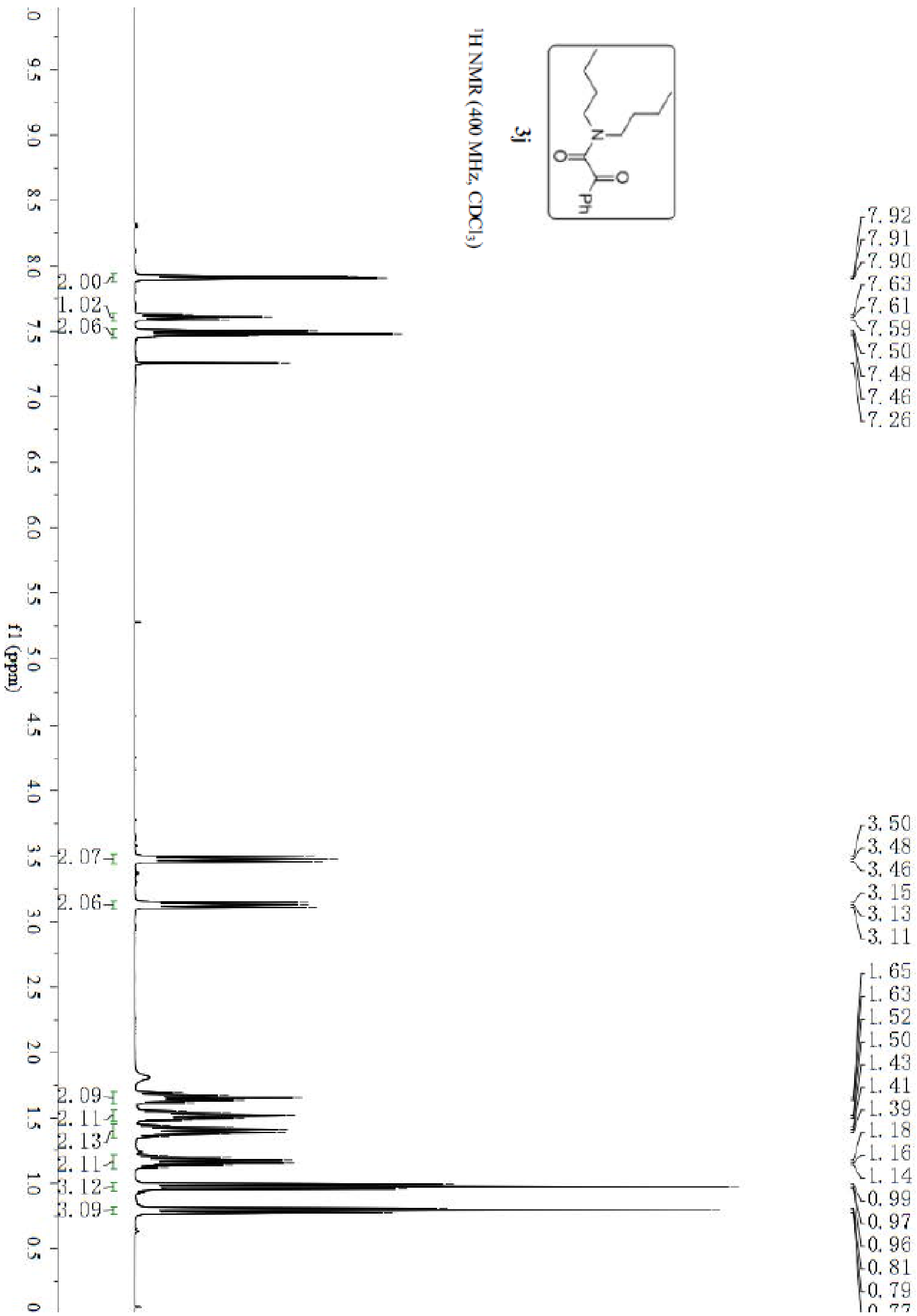

Supplementary Figure 74. ^{1}H NMR (400 MHz, $CDCl_3$) spectra of compound **3j**.

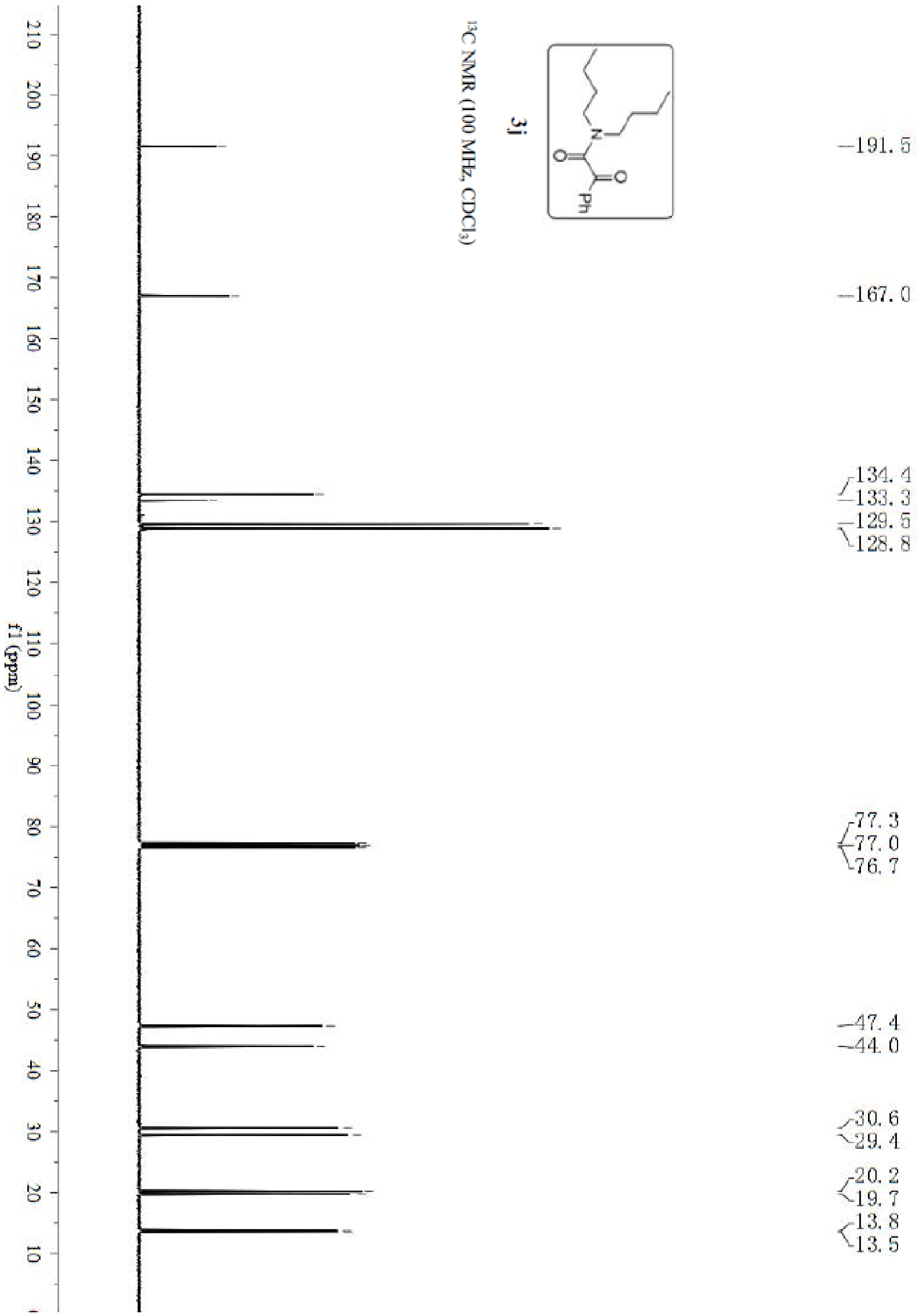

Supplementary Figure 75. ^{13}C NMR (100 MHz, $CDCl_3$) spectra of compound **3j**.

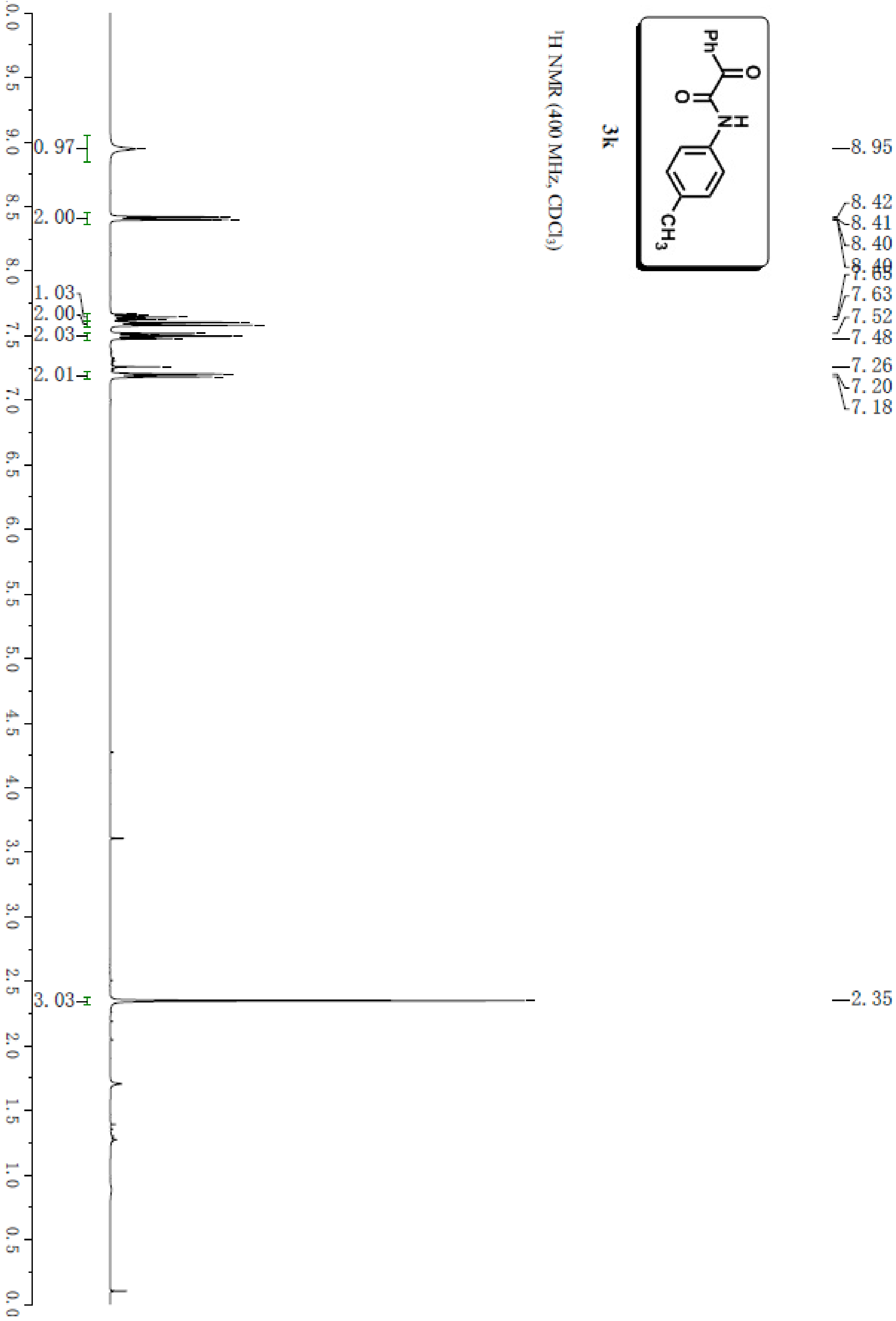

Supplementary Figure 76. ^{1}H NMR (400 MHz, $CDCl_3$) spectra of compound **3k**.

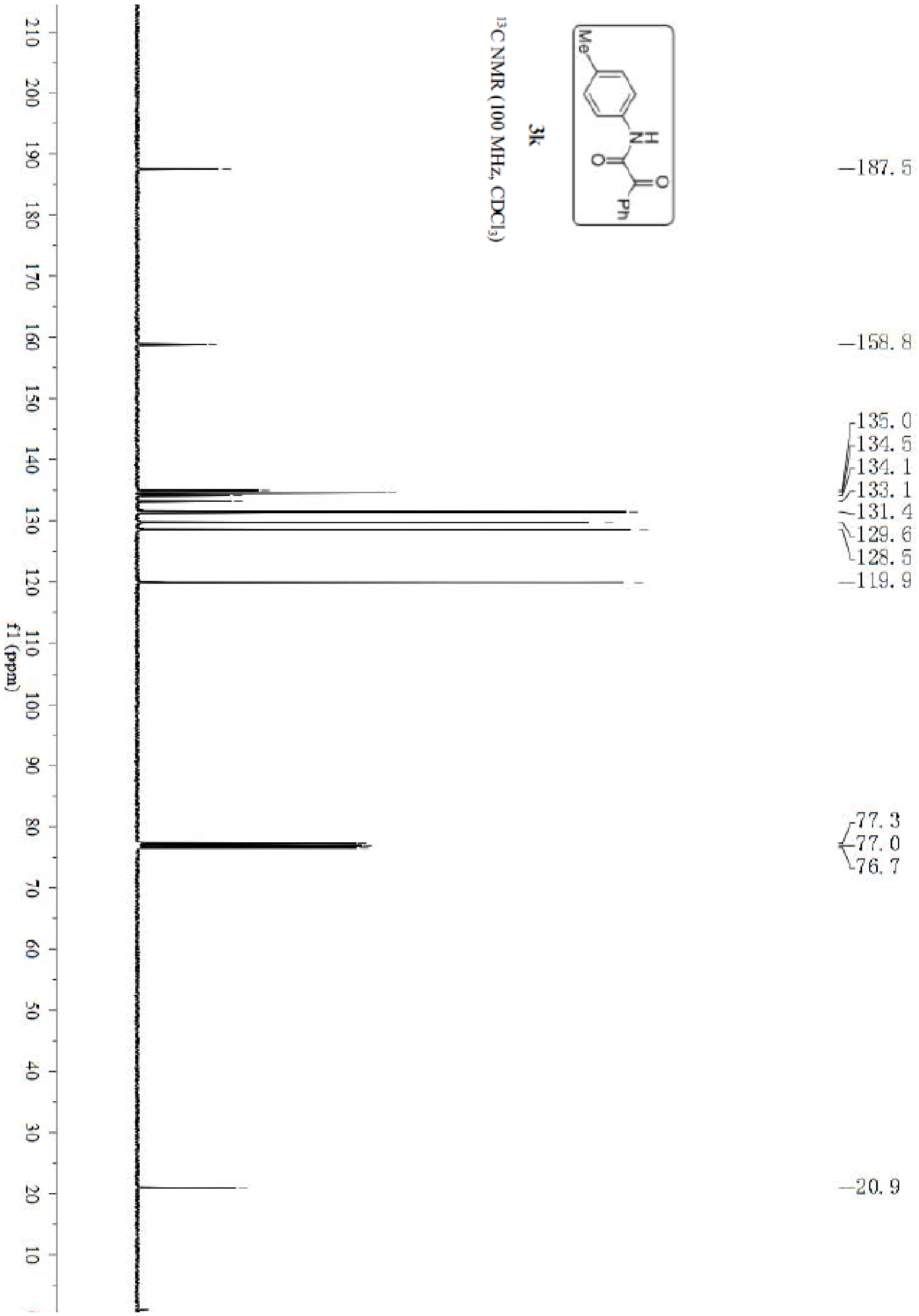

Supplementary Figure 77. ^{13}C NMR (100 MHz, $CDCl_3$) spectra of compound **3k**.

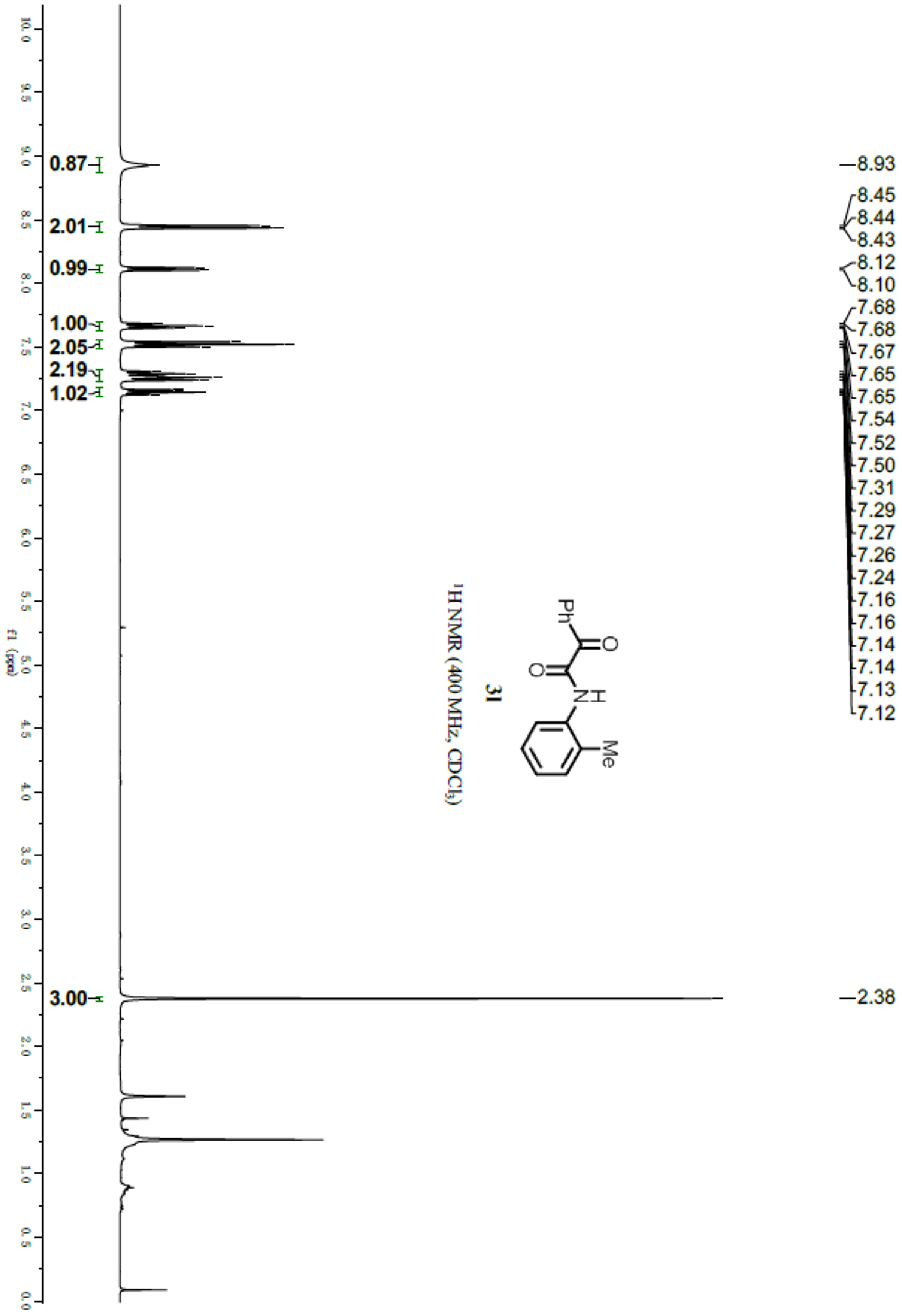

Supplementary Figure 78. ^{1}H NMR (400 MHz, $CDCl_3$) spectra of compound **3l**.

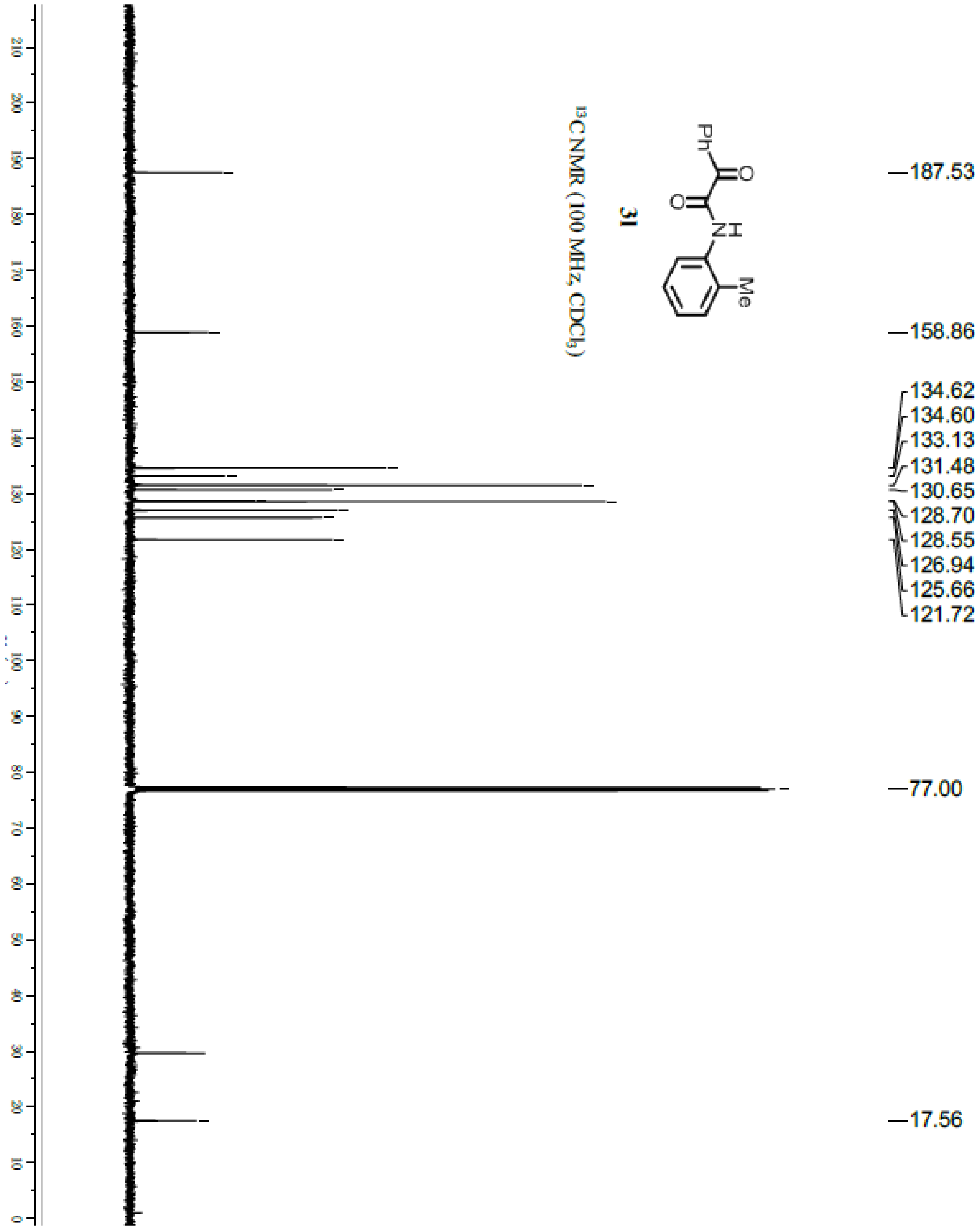

Supplementary Figure 79. ^{13}C NMR (100 MHz, $CDCl_3$) spectra of compound **3l**.

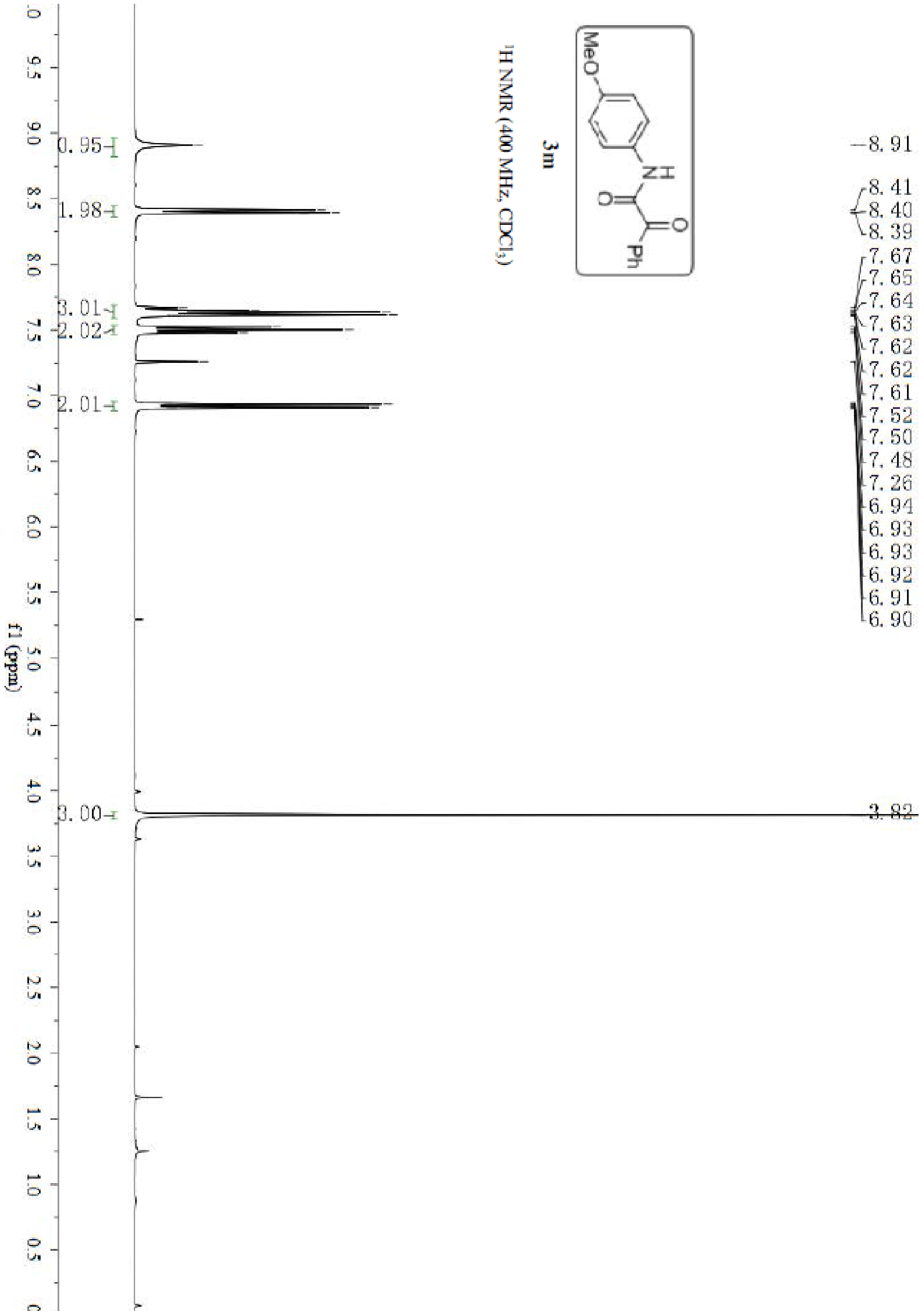

Supplementary Figure 80. ^{1}H NMR (400 MHz, $CDCl_3$) spectra of compound **3m**.

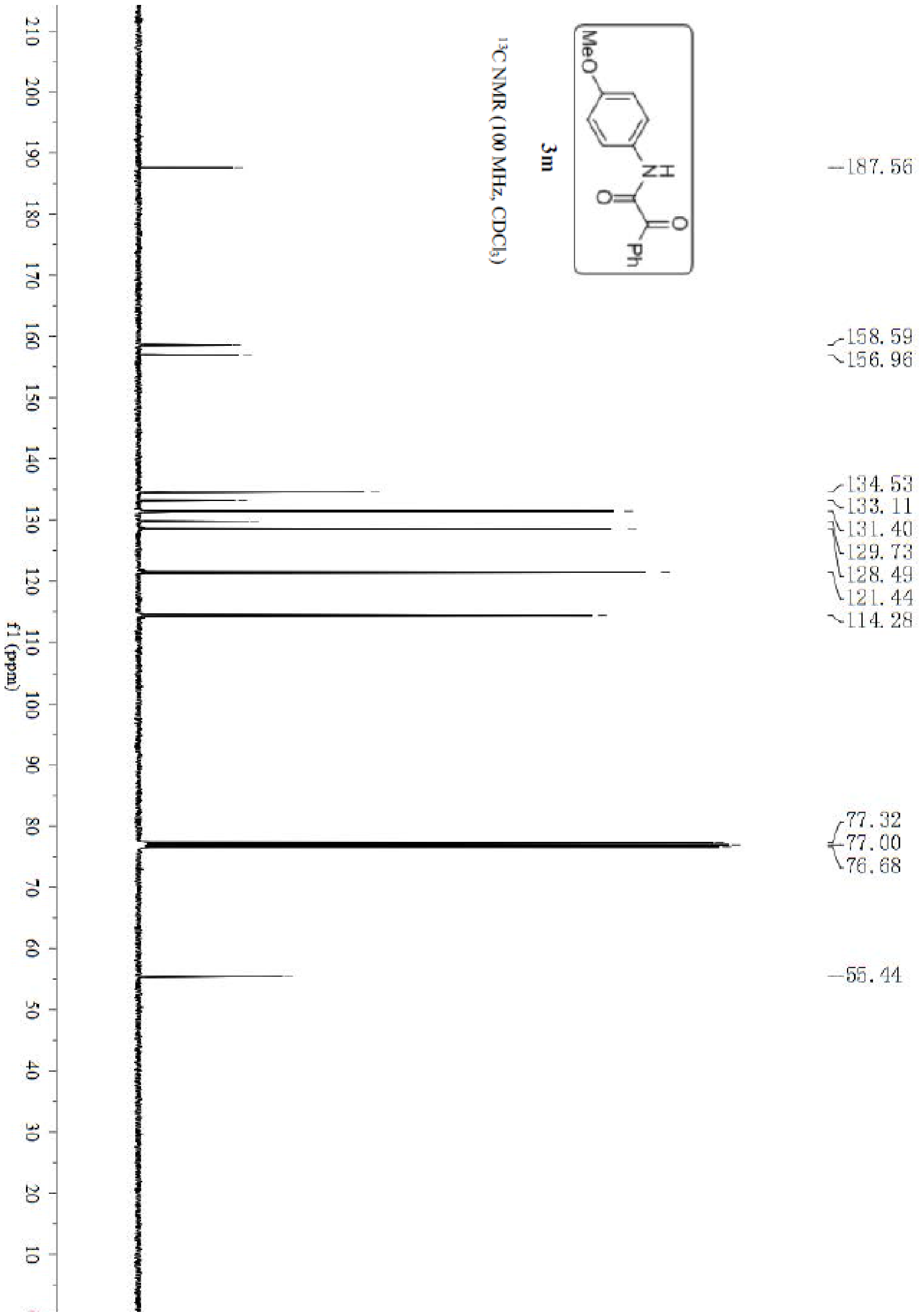

Supplementary Figure 81. ^{13}C NMR (100 MHz, $CDCl_3$) spectra of compound **3m**.

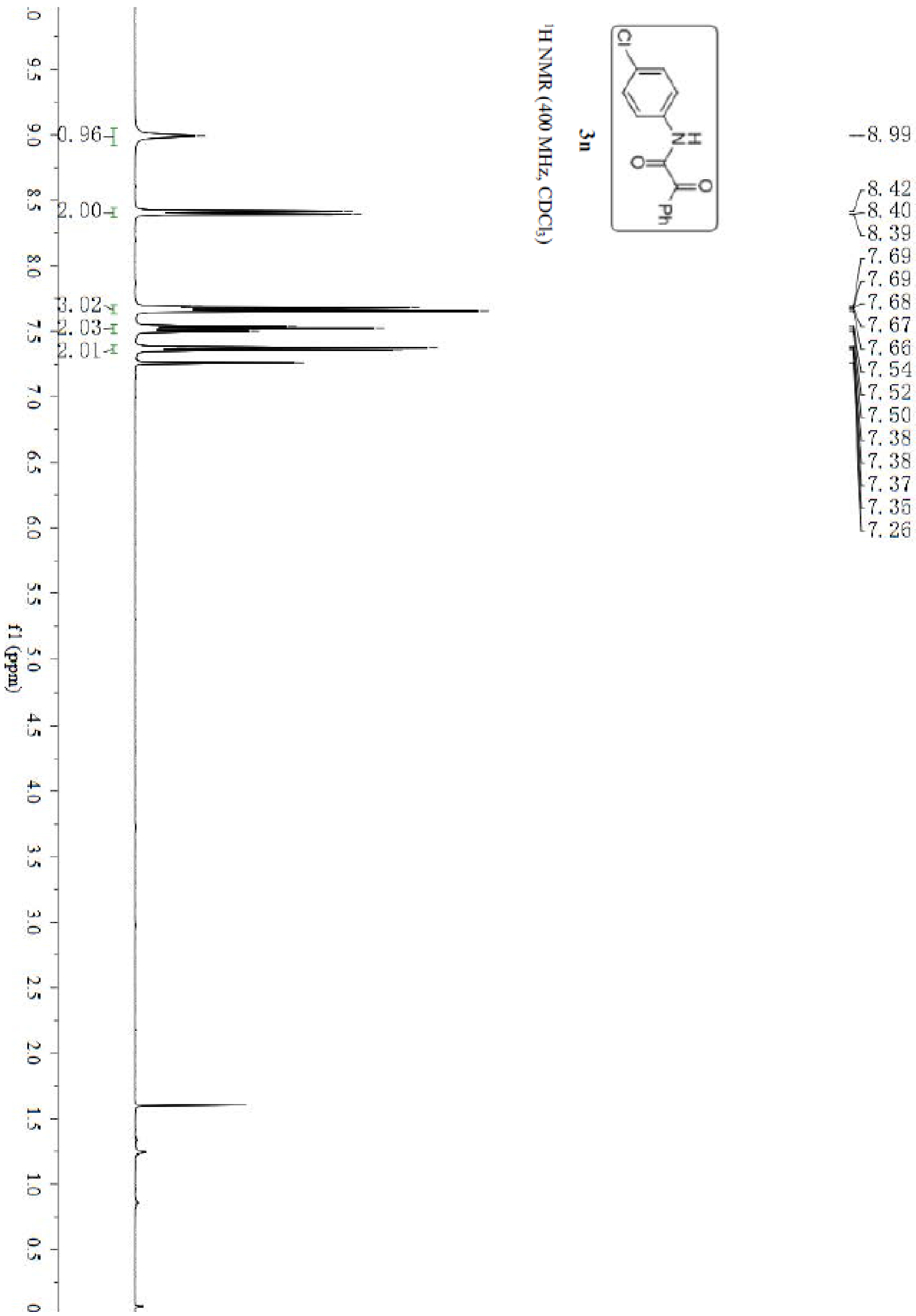

Supplementary Figure 82. ^{1}H NMR (400 MHz, $CDCl_3$) spectra of compound **3n**.

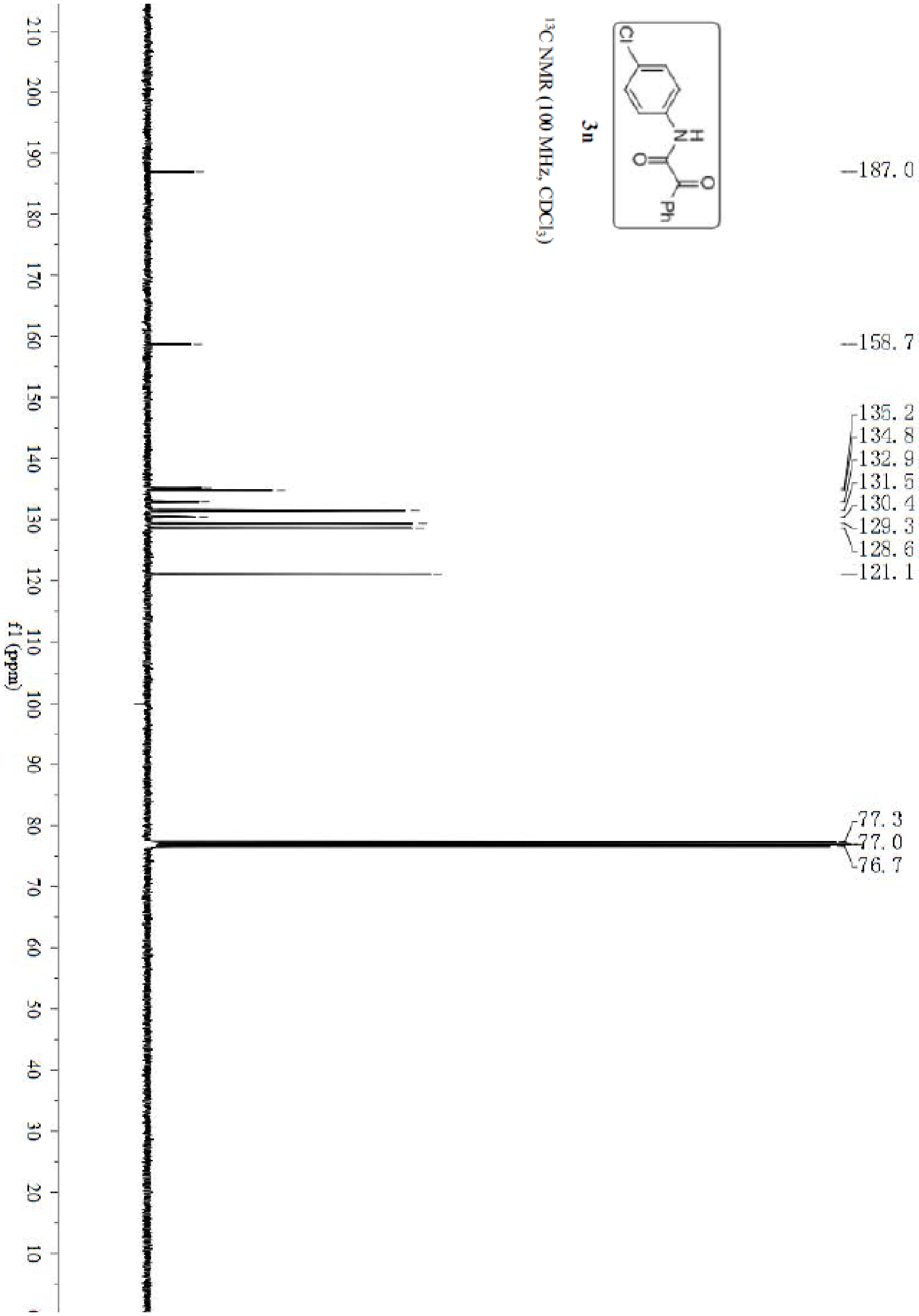

Supplementary Figure 83. ^{13}C NMR (100 MHz, $CDCl_3$) spectra of compound **3n**.

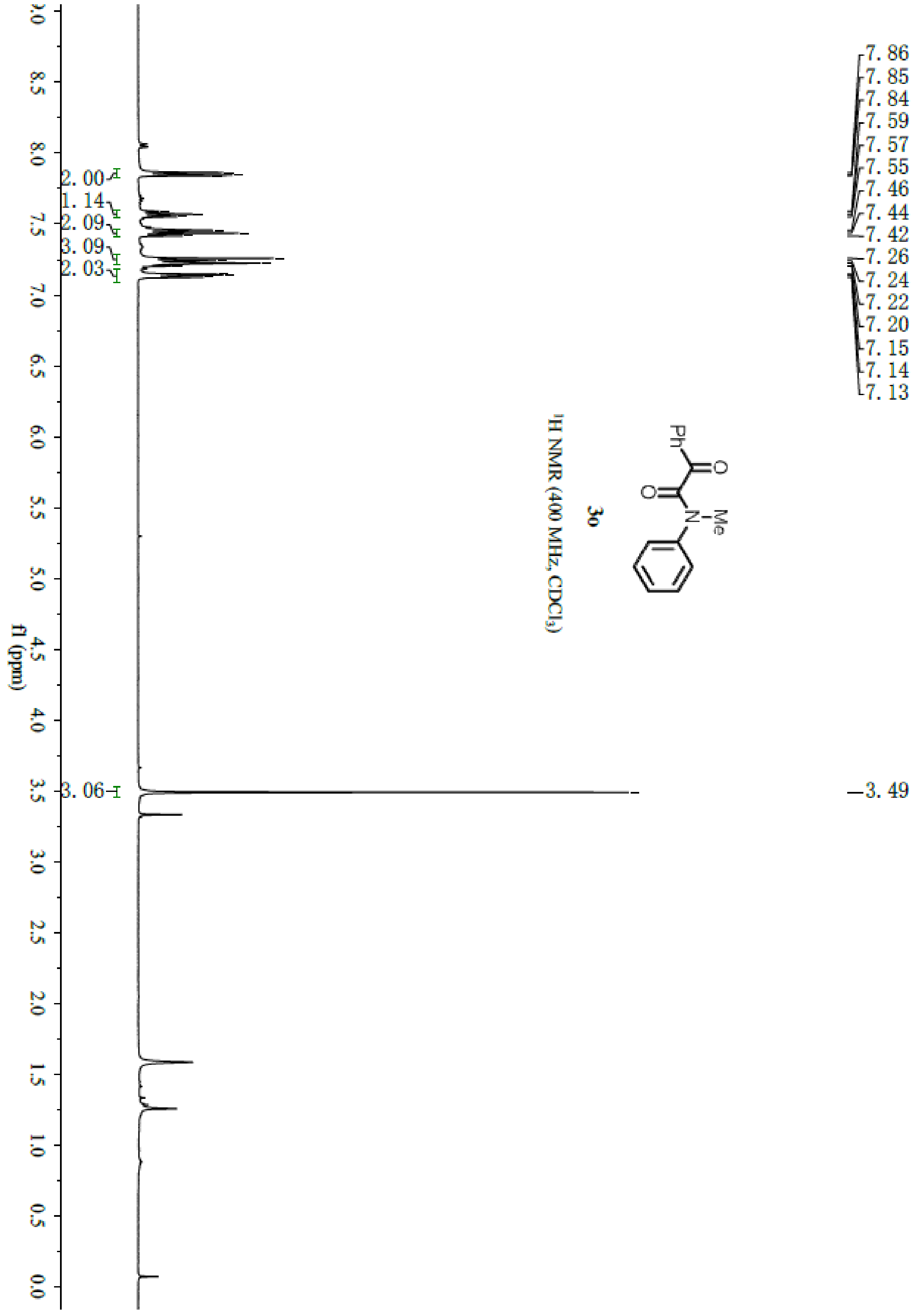

Supplementary Figure 84. ^{1}H NMR (400 MHz, $CDCl_3$) spectra of compound **3o**.

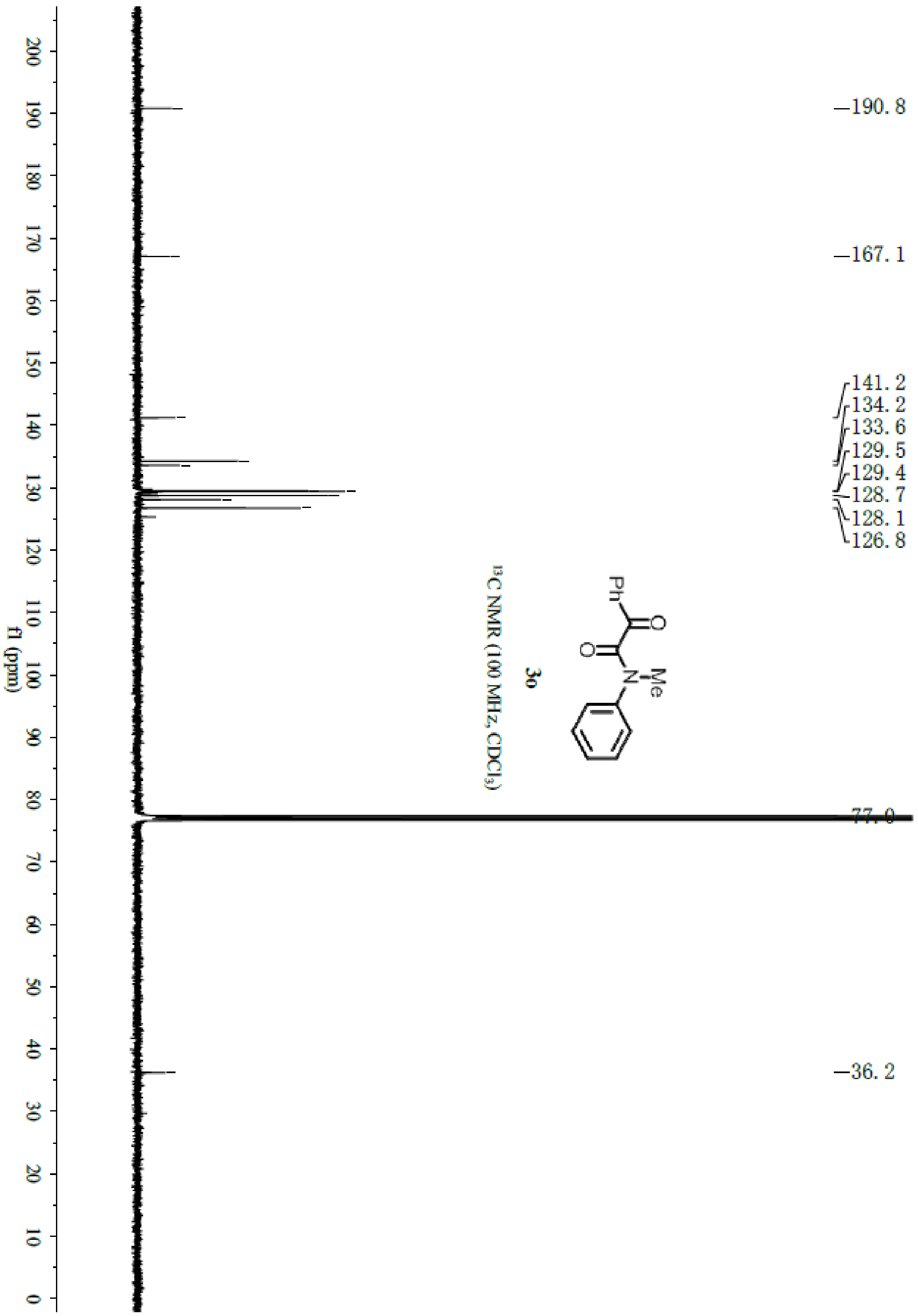

Supplementary Figure 85. ^{13}C NMR (100 MHz, $CDCl_3$) spectra of compound **3o**.

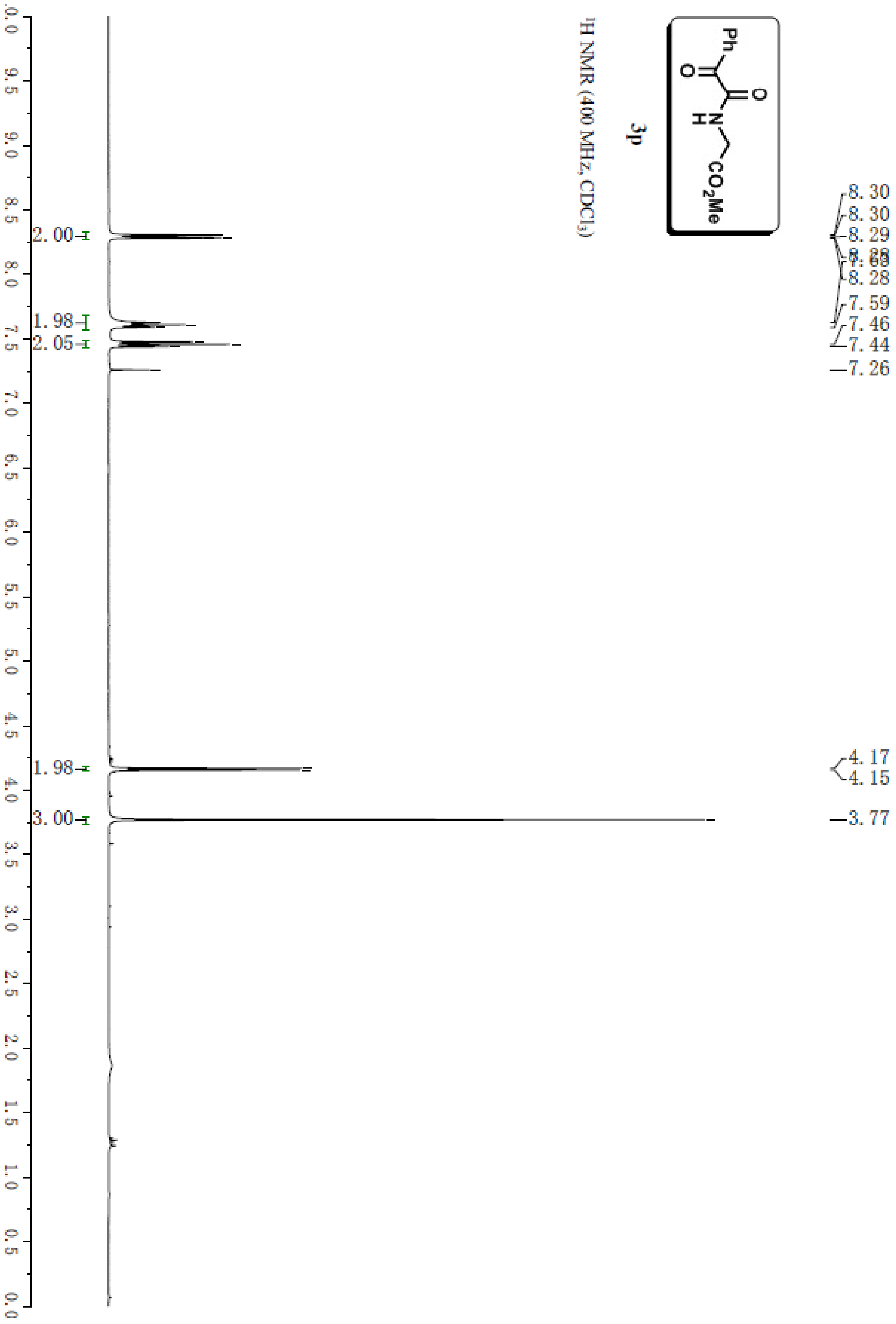

Supplementary Figure 86. ^{1}H NMR (400 MHz, $CDCl_3$) spectra of compound **3p**.

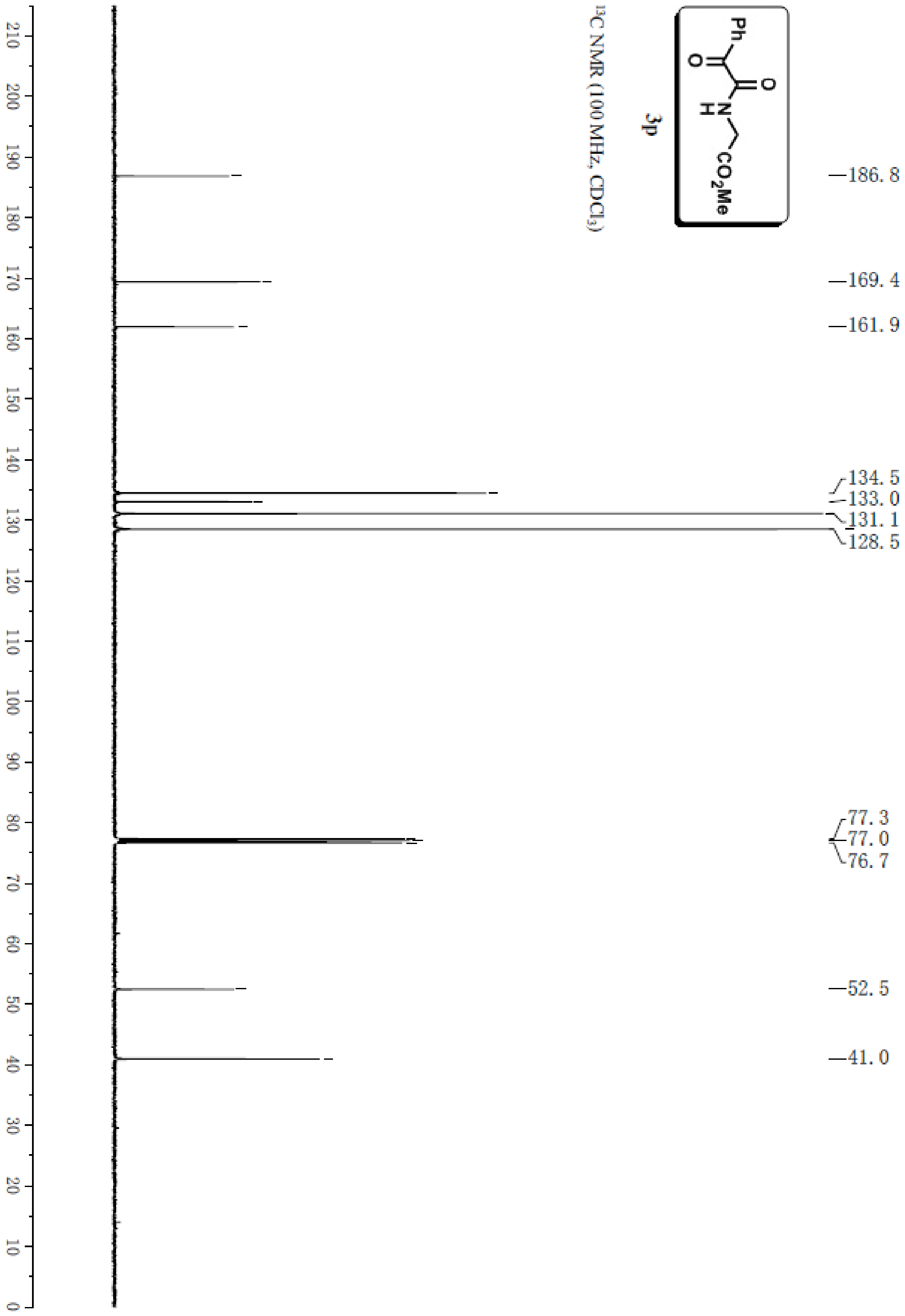

Supplementary Figure 87. ^{13}C NMR (100 MHz, $CDCl_3$) spectra of compound **3p**.

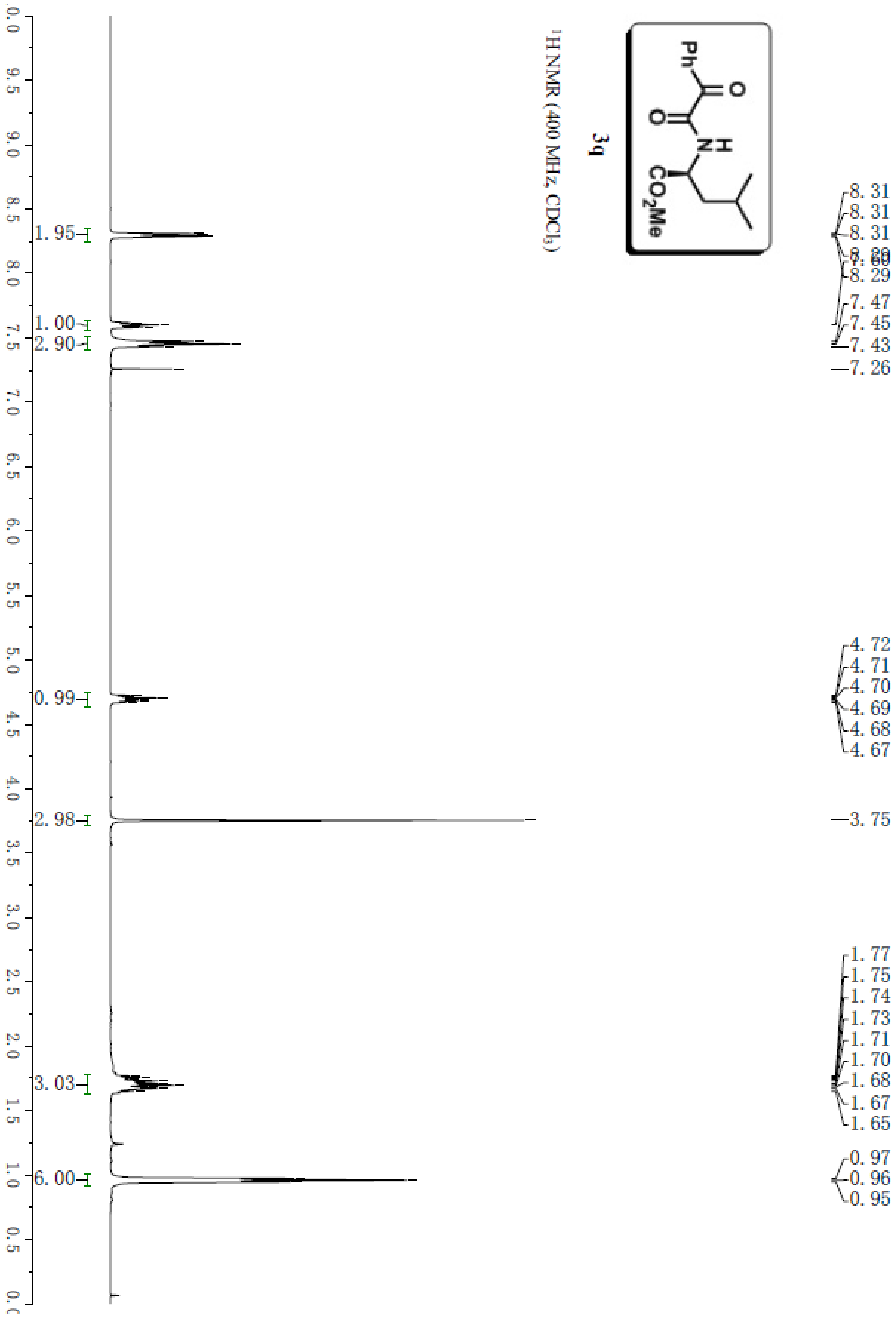

Supplementary Figure 88. ^{1}H NMR (400 MHz, $CDCl_3$) spectra of compound **3q**.

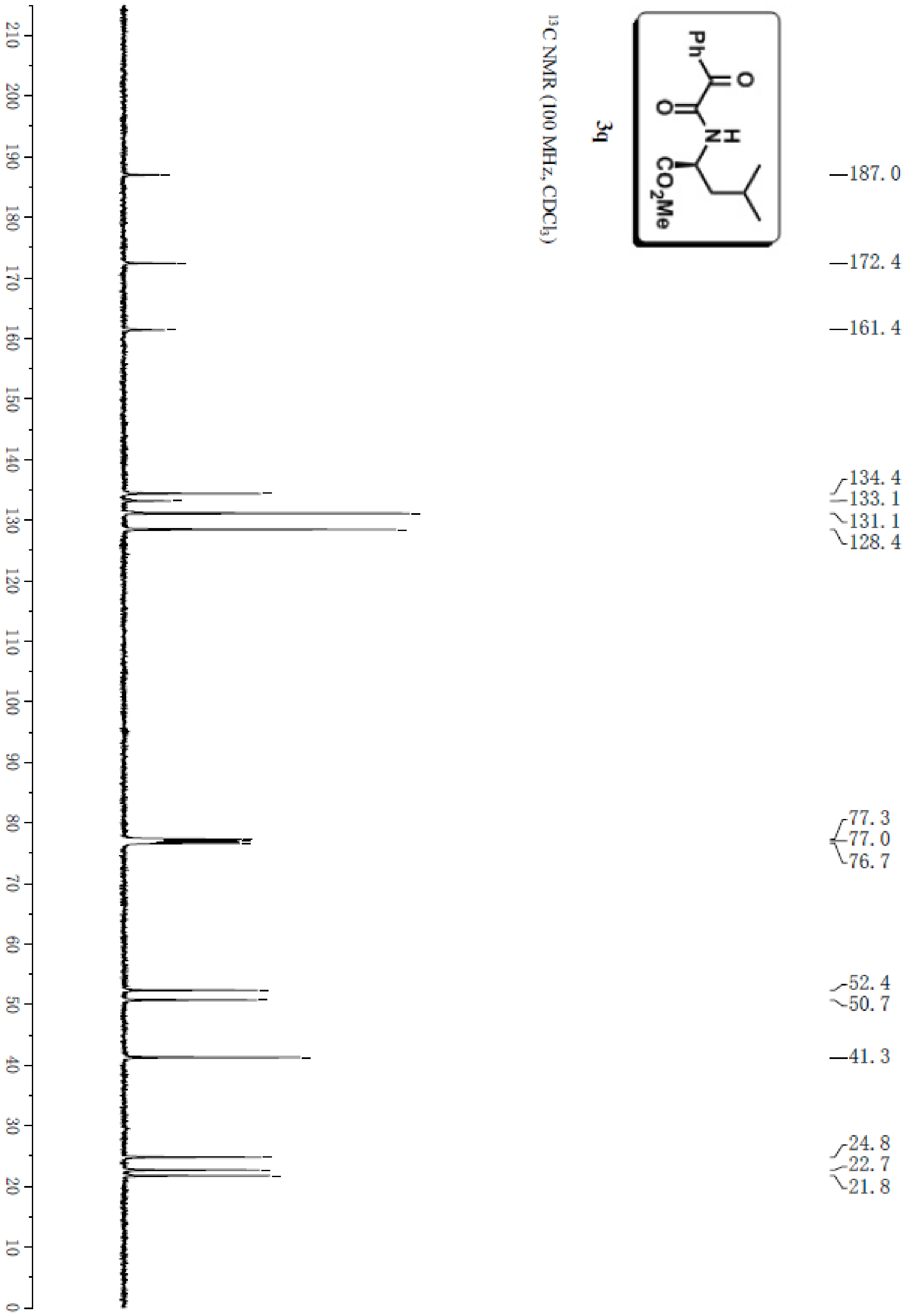

Supplementary Figure 89. ^{13}C NMR (100 MHz, $CDCl_3$) spectra of compound **3q**.

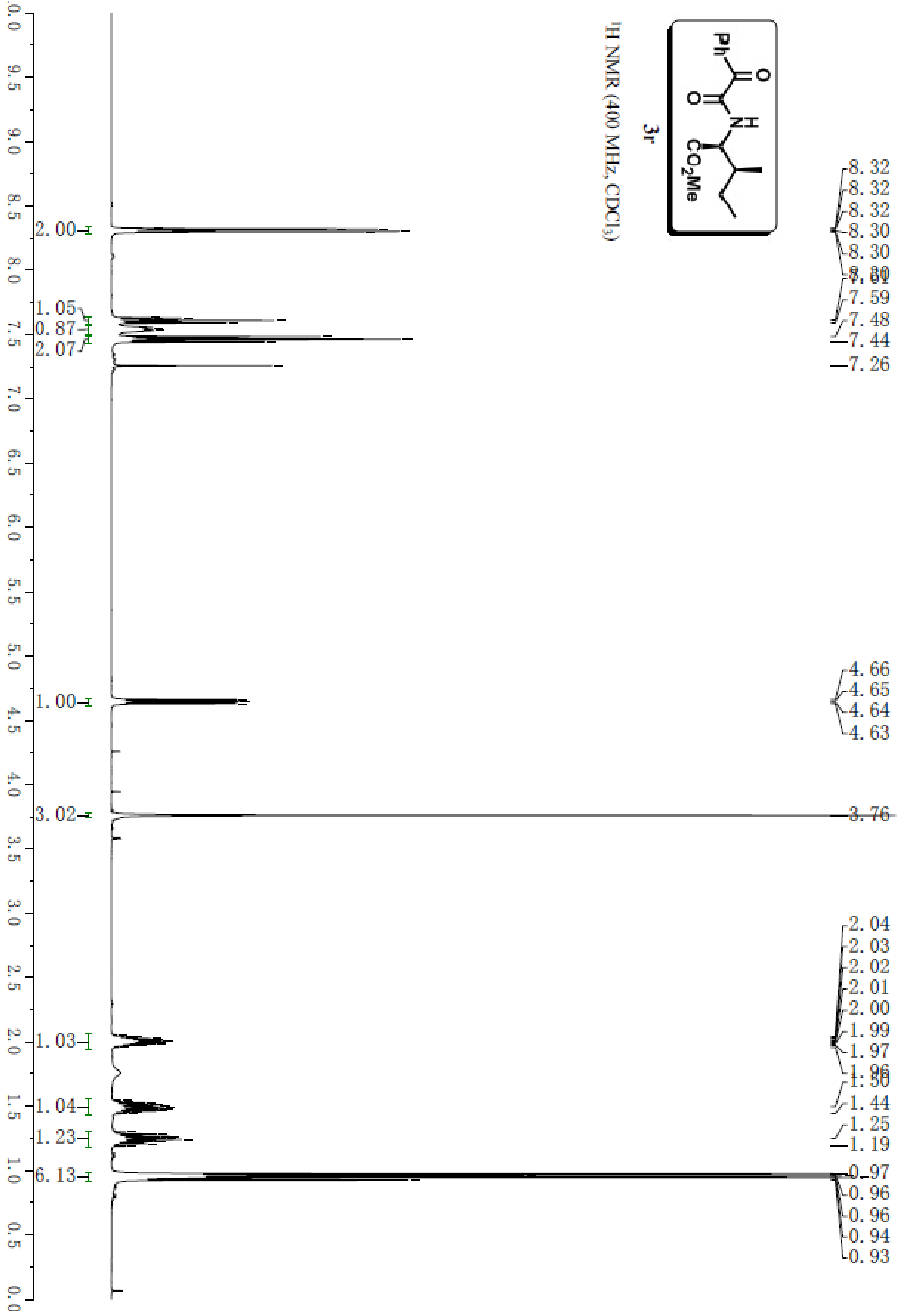

Supplementary Figure 90. ^{1}H NMR (400 MHz, $CDCl_3$) spectra of compound **3r**.

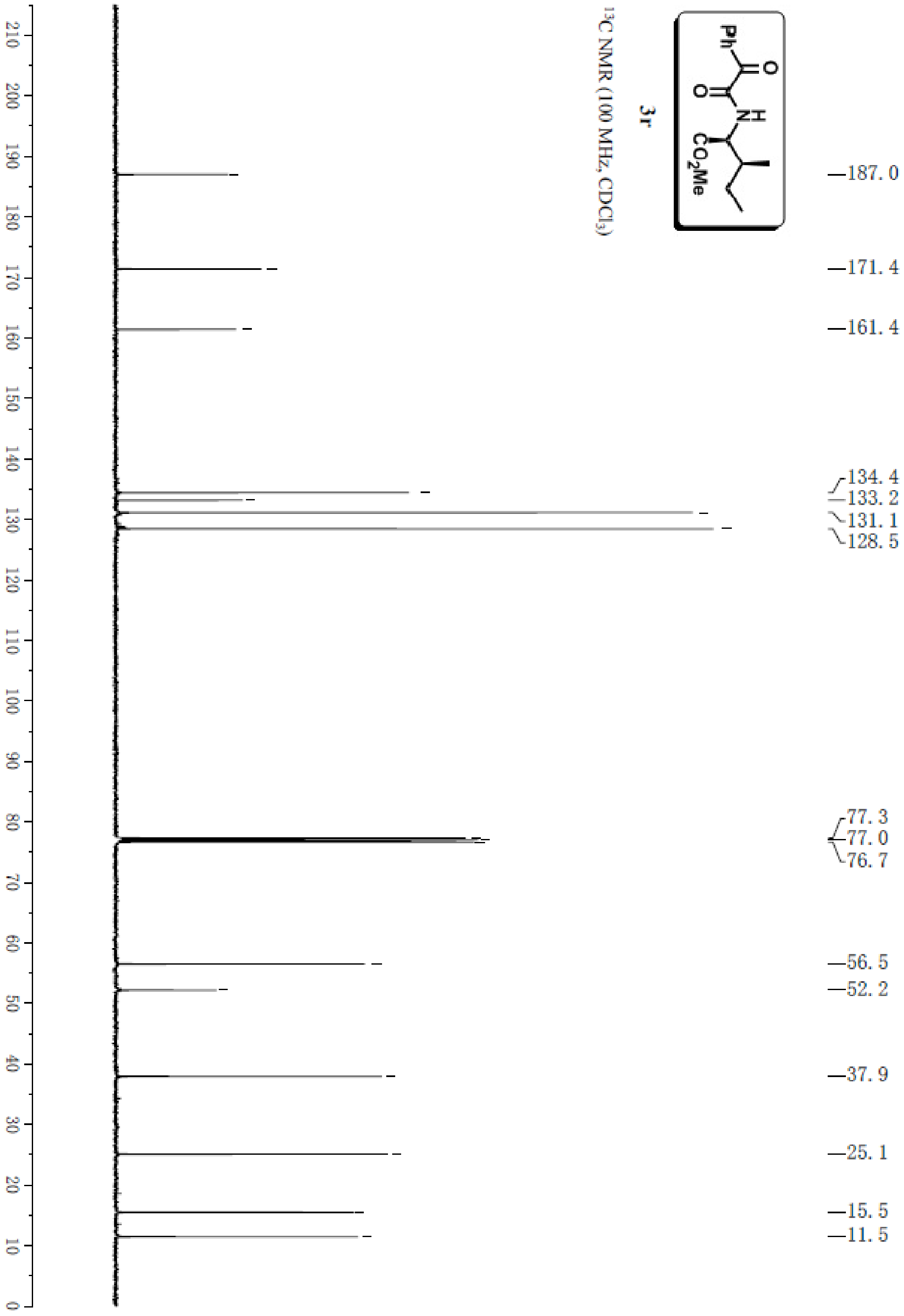

Supplementary Figure 91. ^{13}C NMR (100 MHz, $CDCl_3$) spectra of compound **3r**.

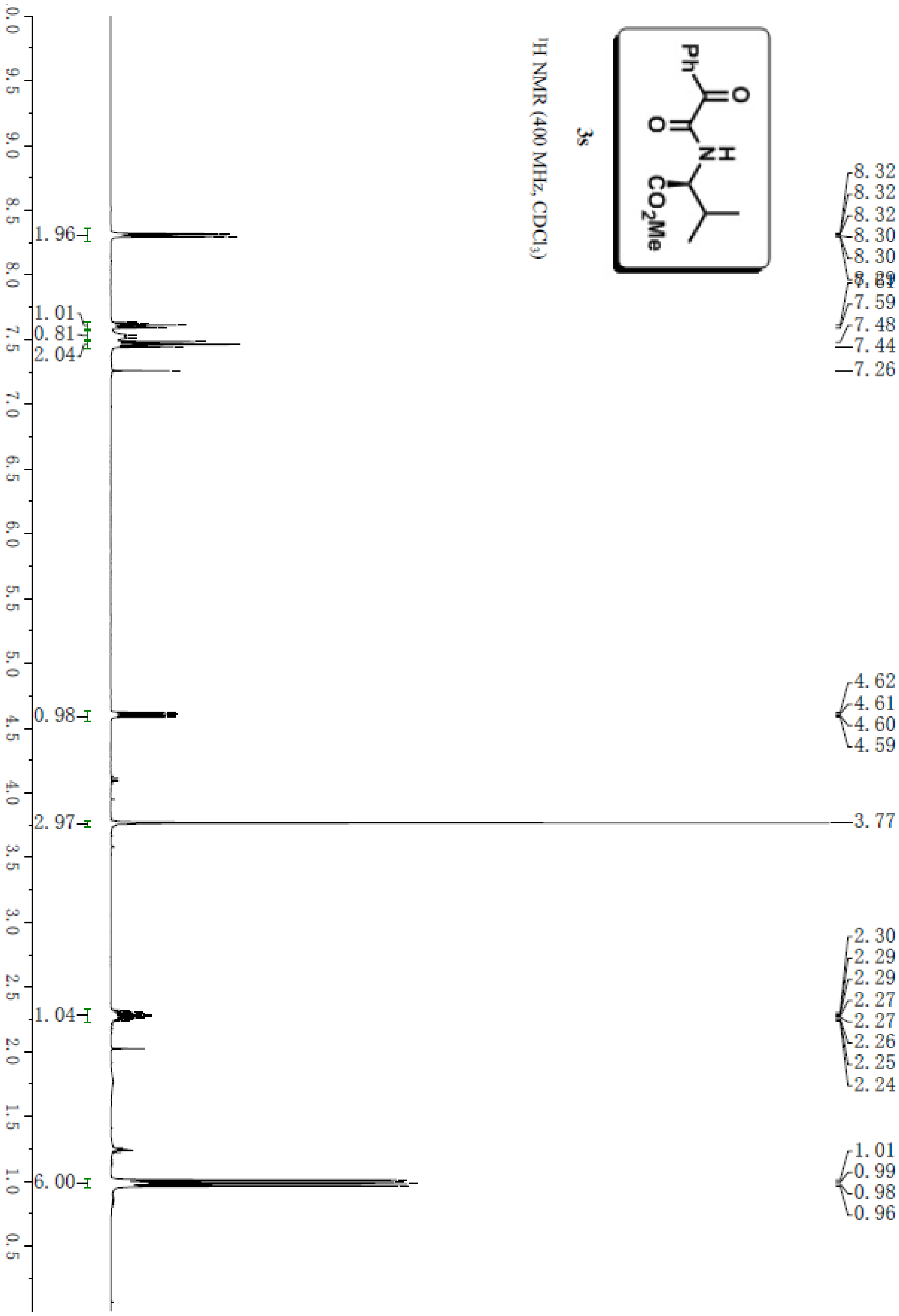

Supplementary Figure 92. ^{1}H NMR (400 MHz, $CDCl_3$) spectra of compound **3s**.

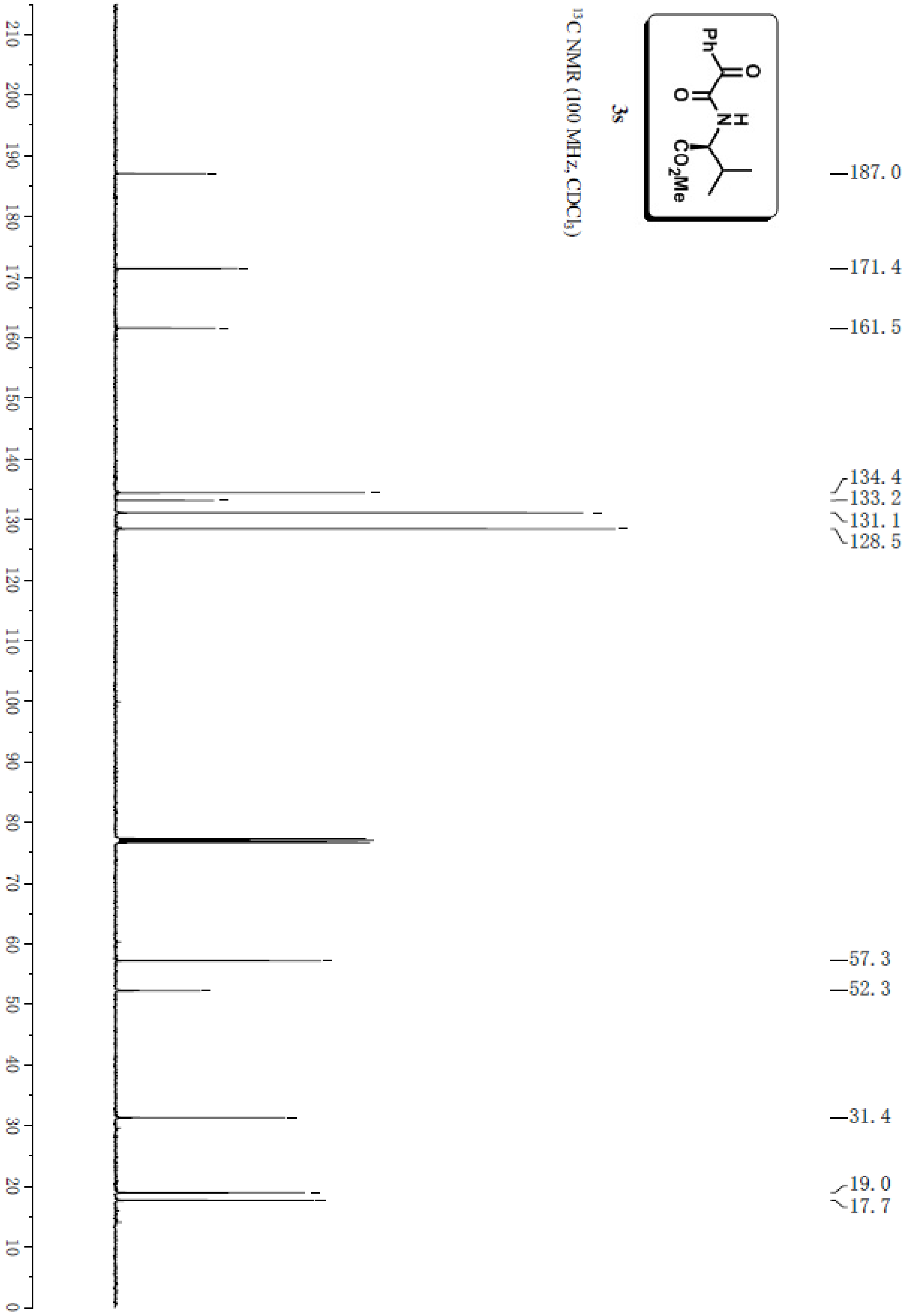

Supplementary Figure 93. ^{13}C NMR (100 MHz, $CDCl_3$) spectra of compound **3s**.

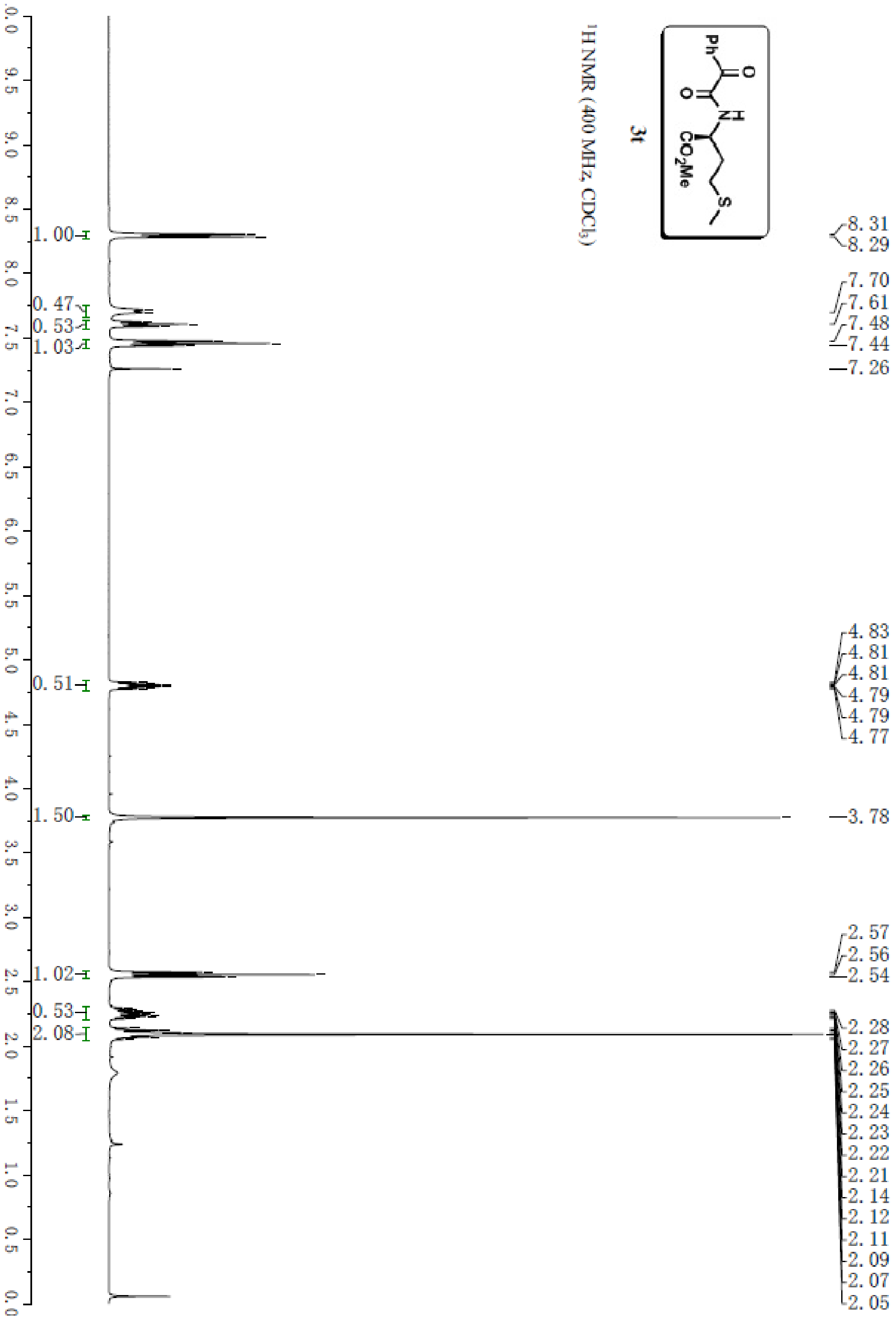

Supplementary Figure 94. ^{1}H NMR (400 MHz, $CDCl_3$) spectra of compound **3t**.

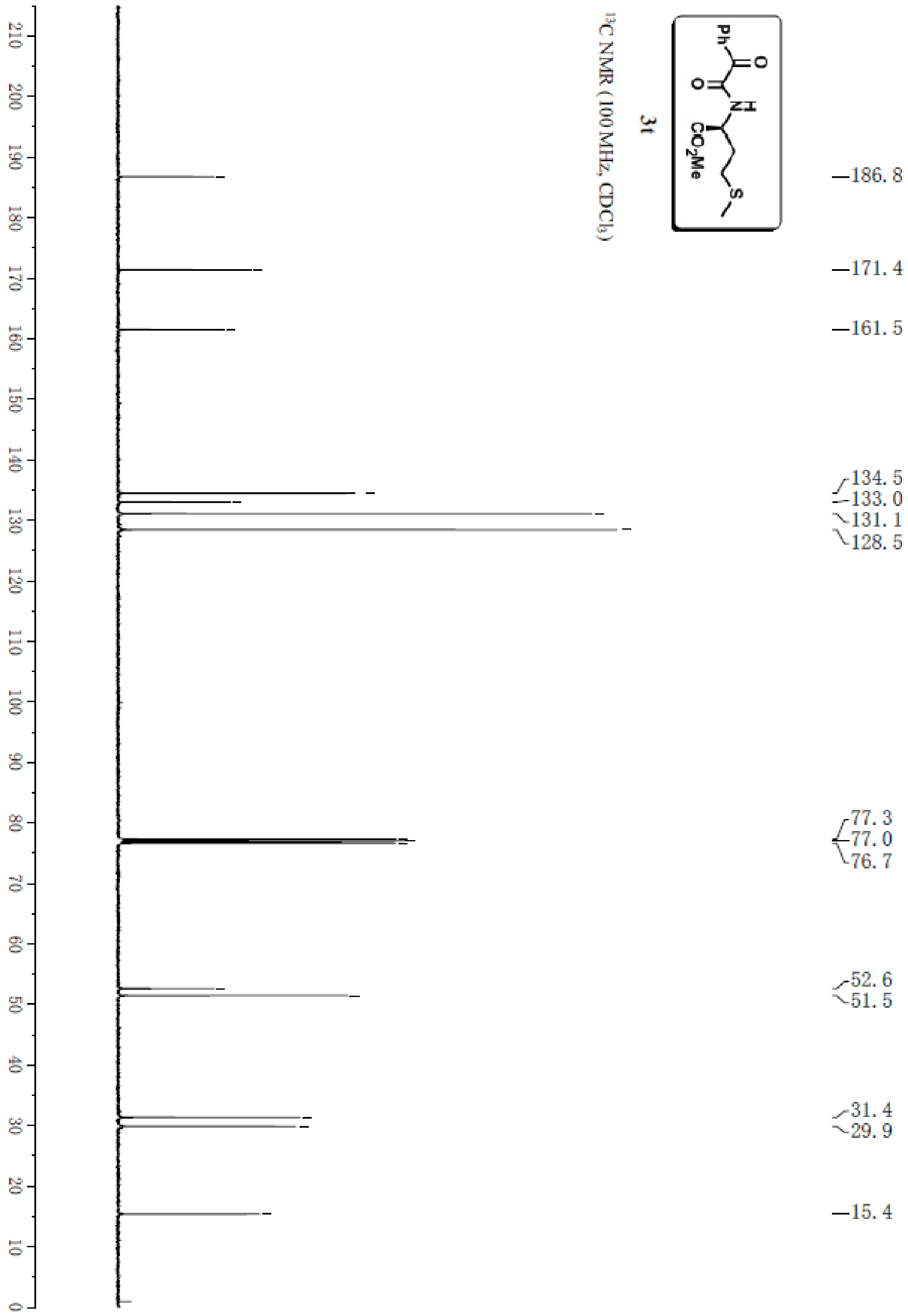

Supplementary Figure 95. ^{13}C NMR (100 MHz, $CDCl_3$) spectra of compound **3t**.

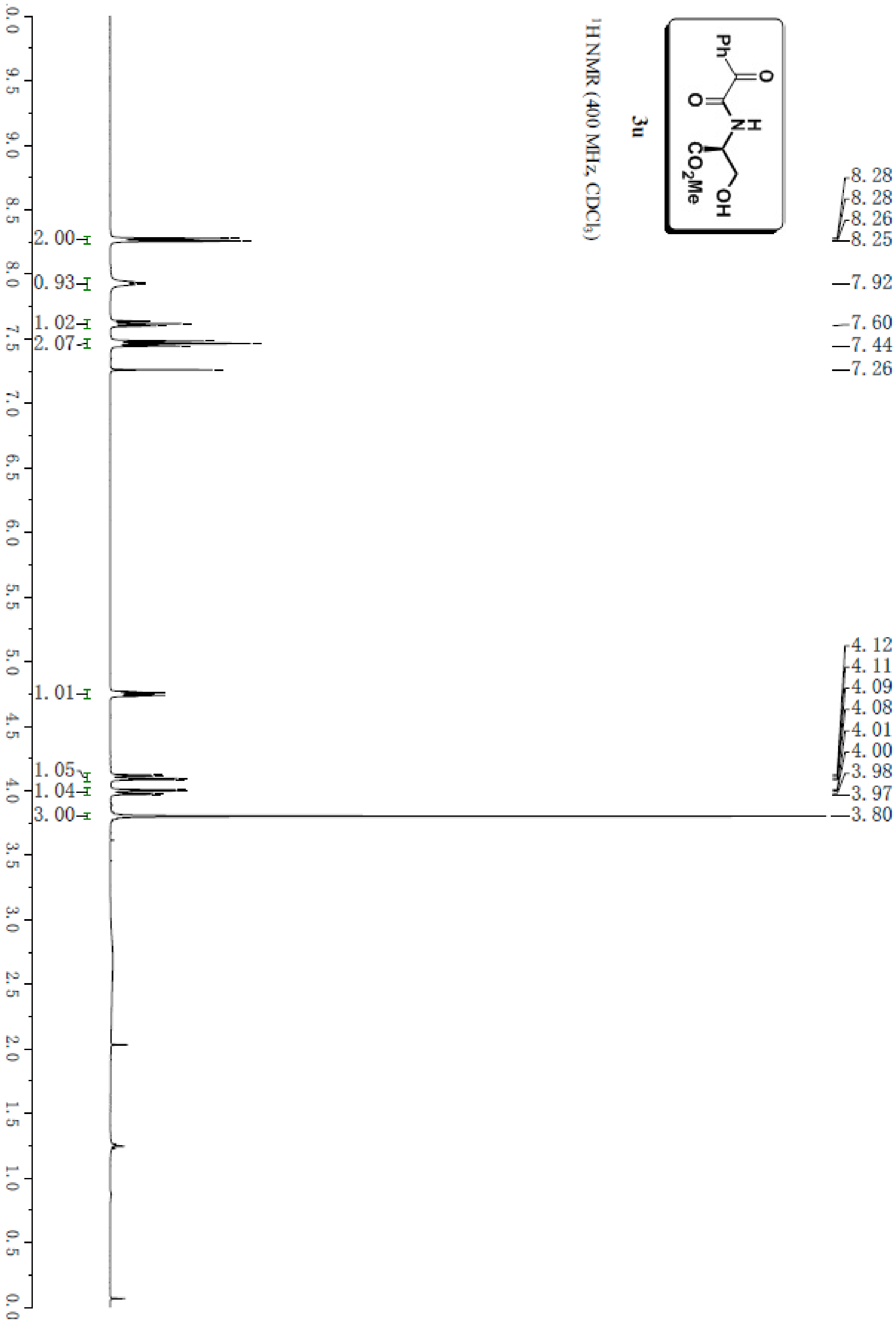

Supplementary Figure 96. ^{1}H NMR (400 MHz, $CDCl_3$) spectra of compound **3u**.

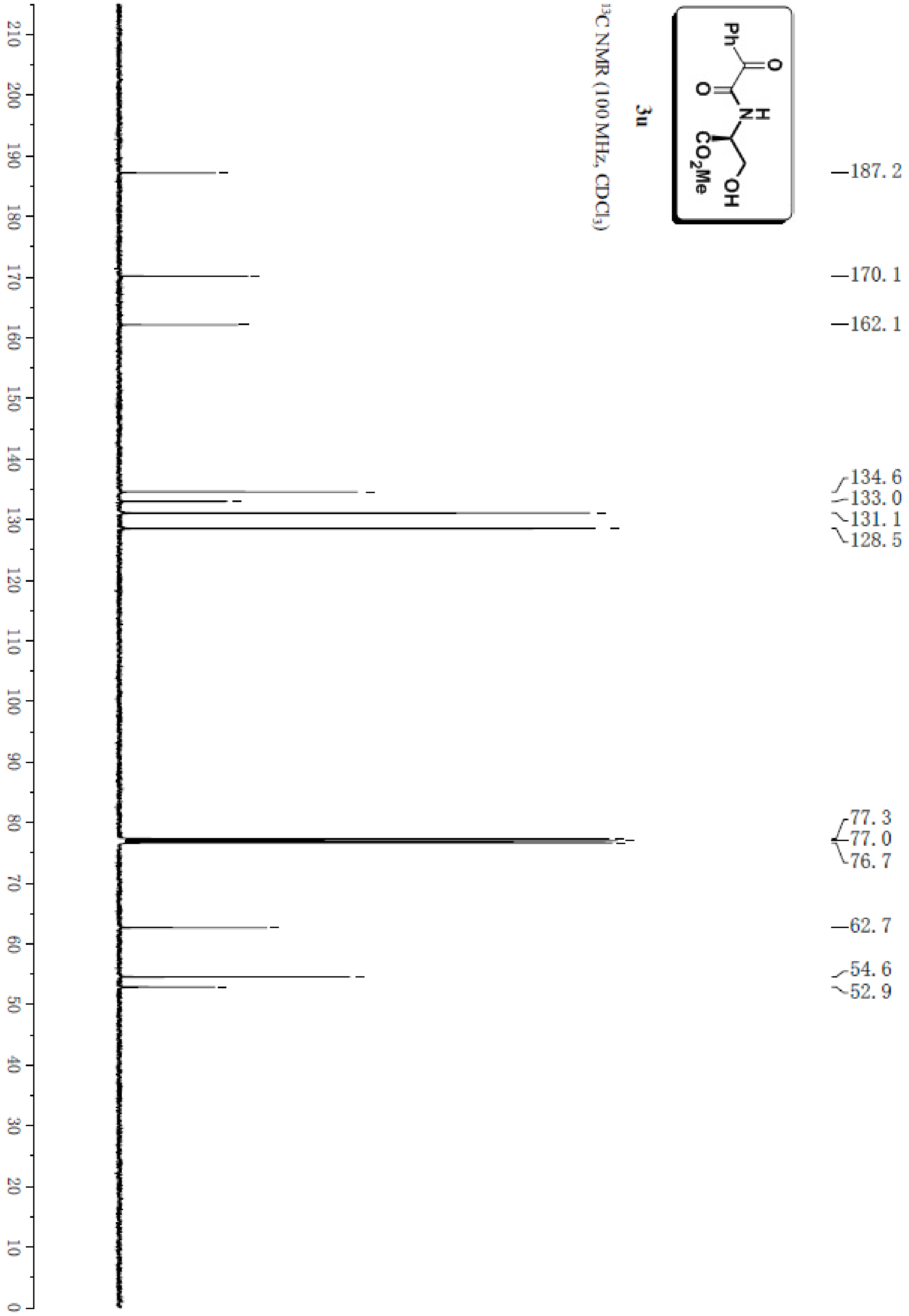

Supplementary Figure 97. ^{13}C NMR (100 MHz, $CDCl_3$) spectra of compound **3u**.

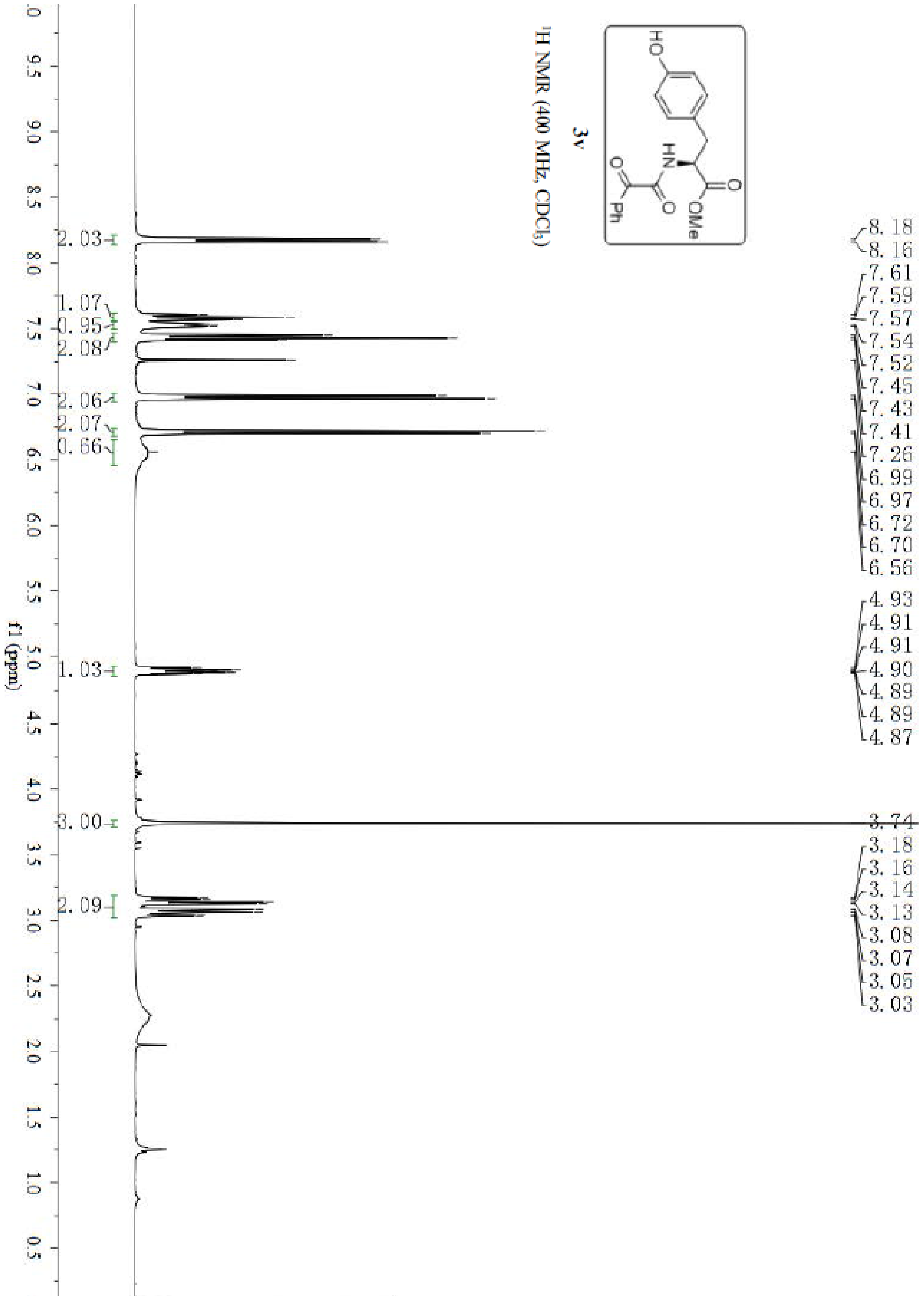

Supplementary Figure 98. ^{1}H NMR (400 MHz, $CDCl_3$) spectra of compound **3v**.

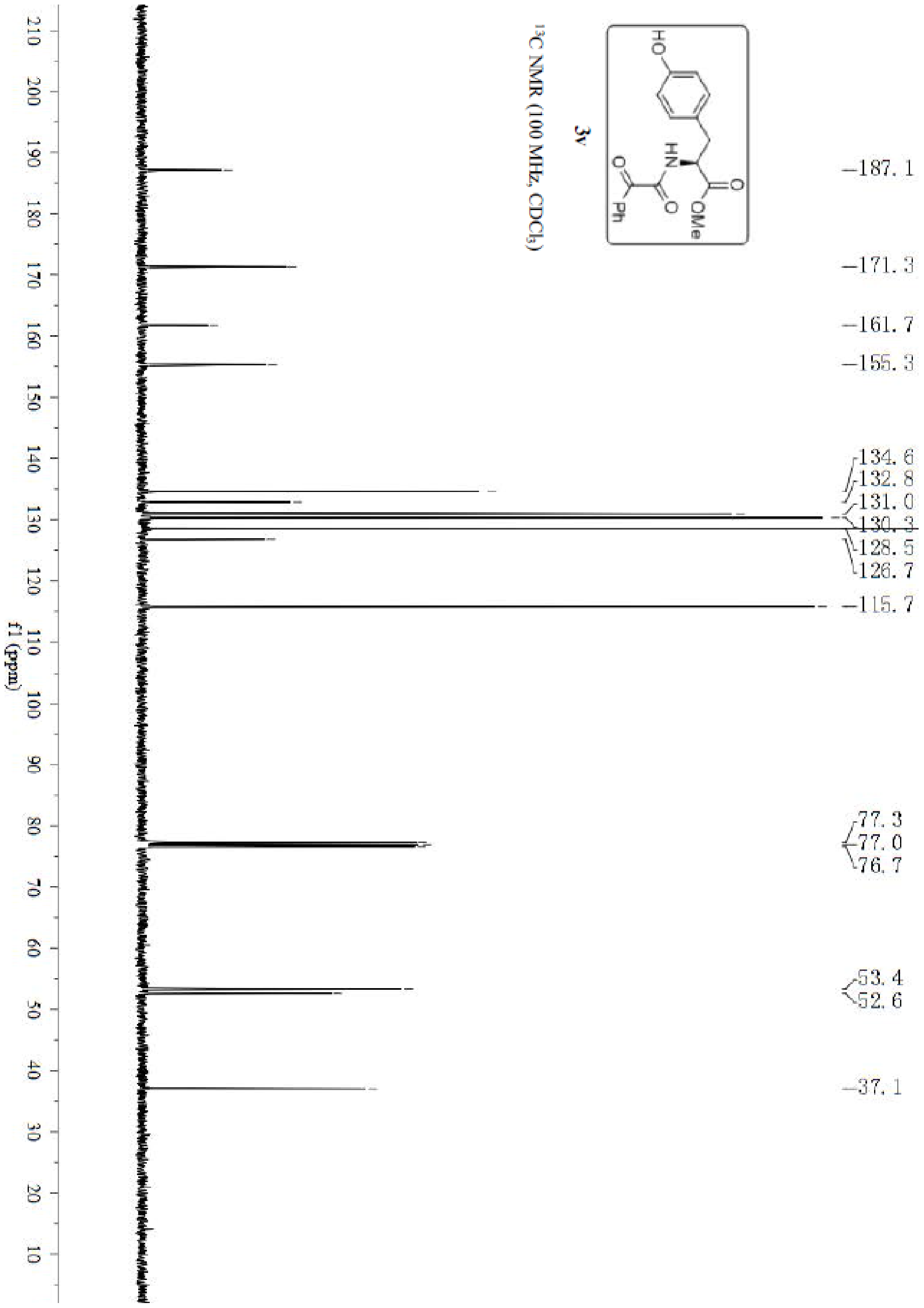

Supplementary Figure 99. ^{13}C NMR (100 MHz, $CDCl_3$) spectra of compound **3v**.

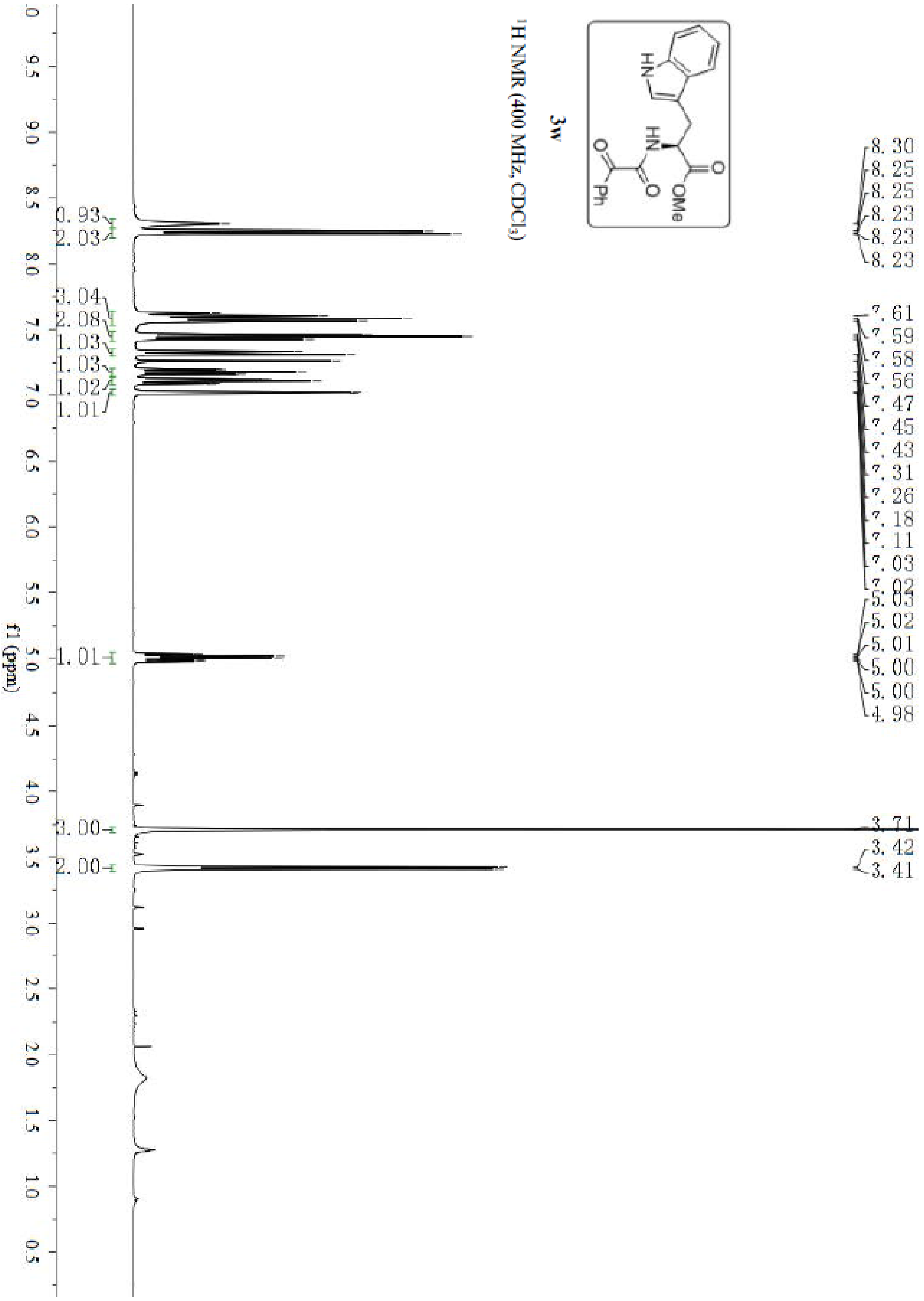

Supplementary Figure 100. ^{1}H NMR (400 MHz, $CDCl_3$) spectra of compound **3w**.

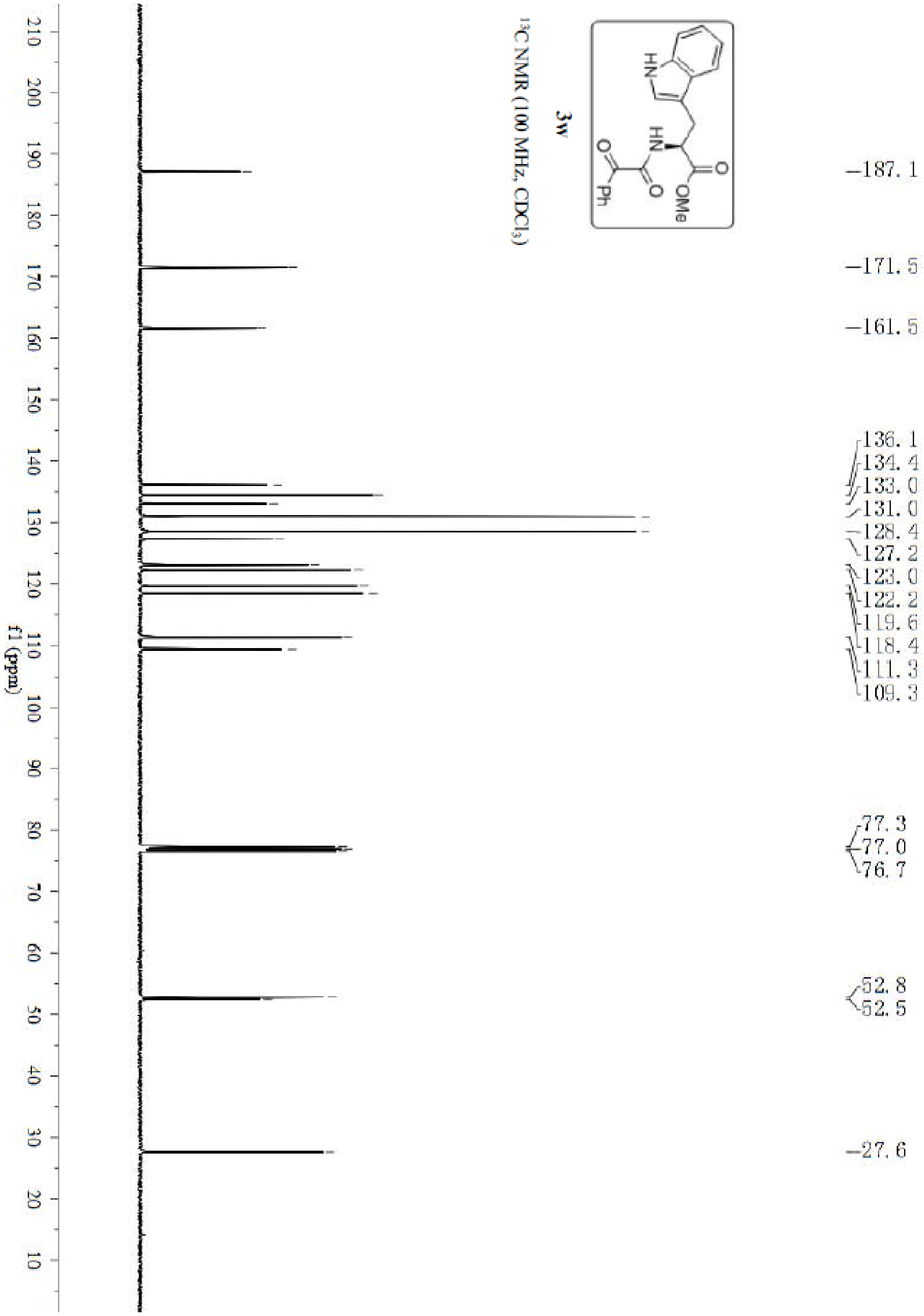

Supplementary Figure 101. ^{13}C NMR (100 MHz, $CDCl_3$) spectra of compound **3w**.

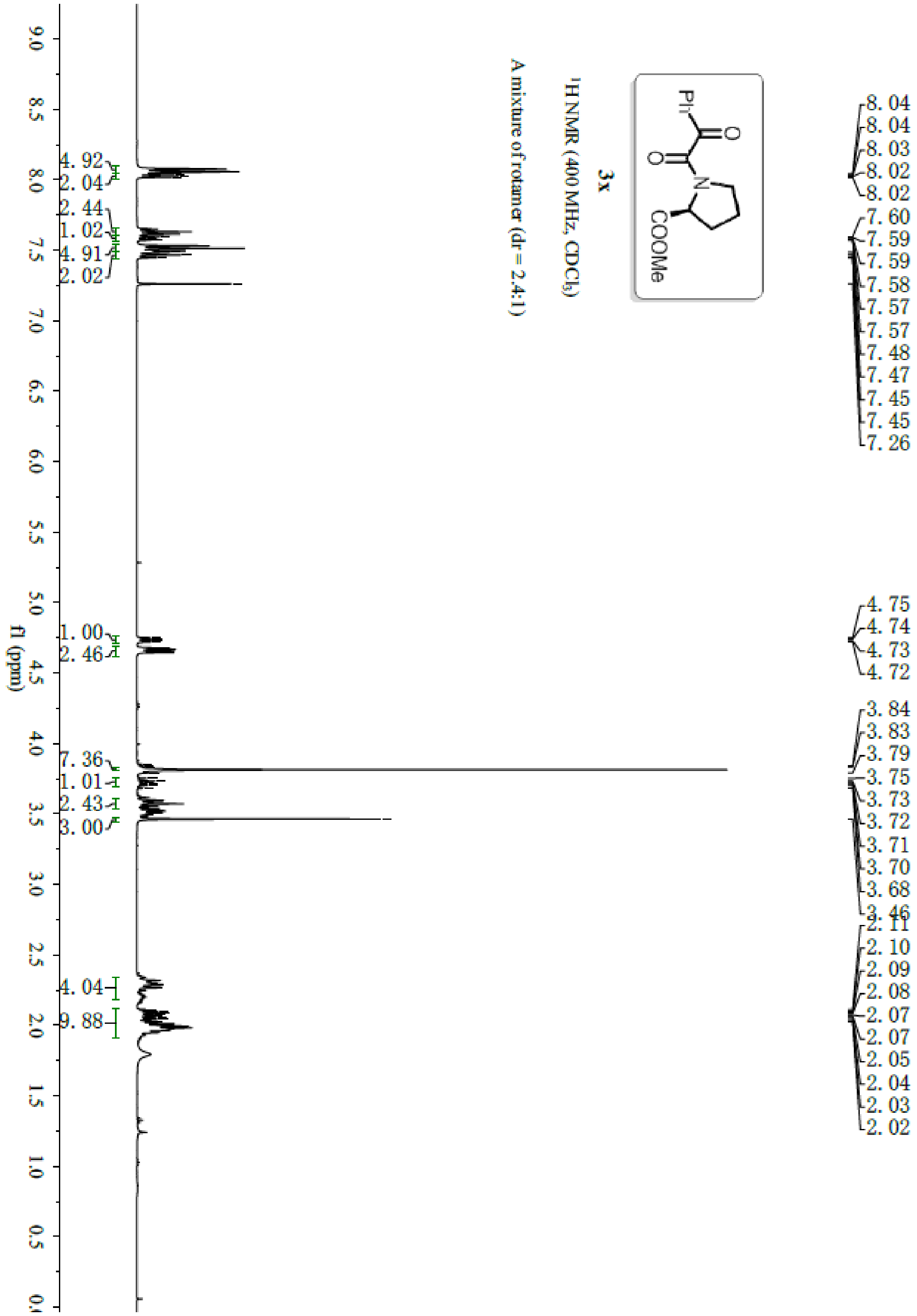

Supplementary Figure 102. ^{1}H NMR (400 MHz, $CDCl_3$) spectra of compound **3x**.

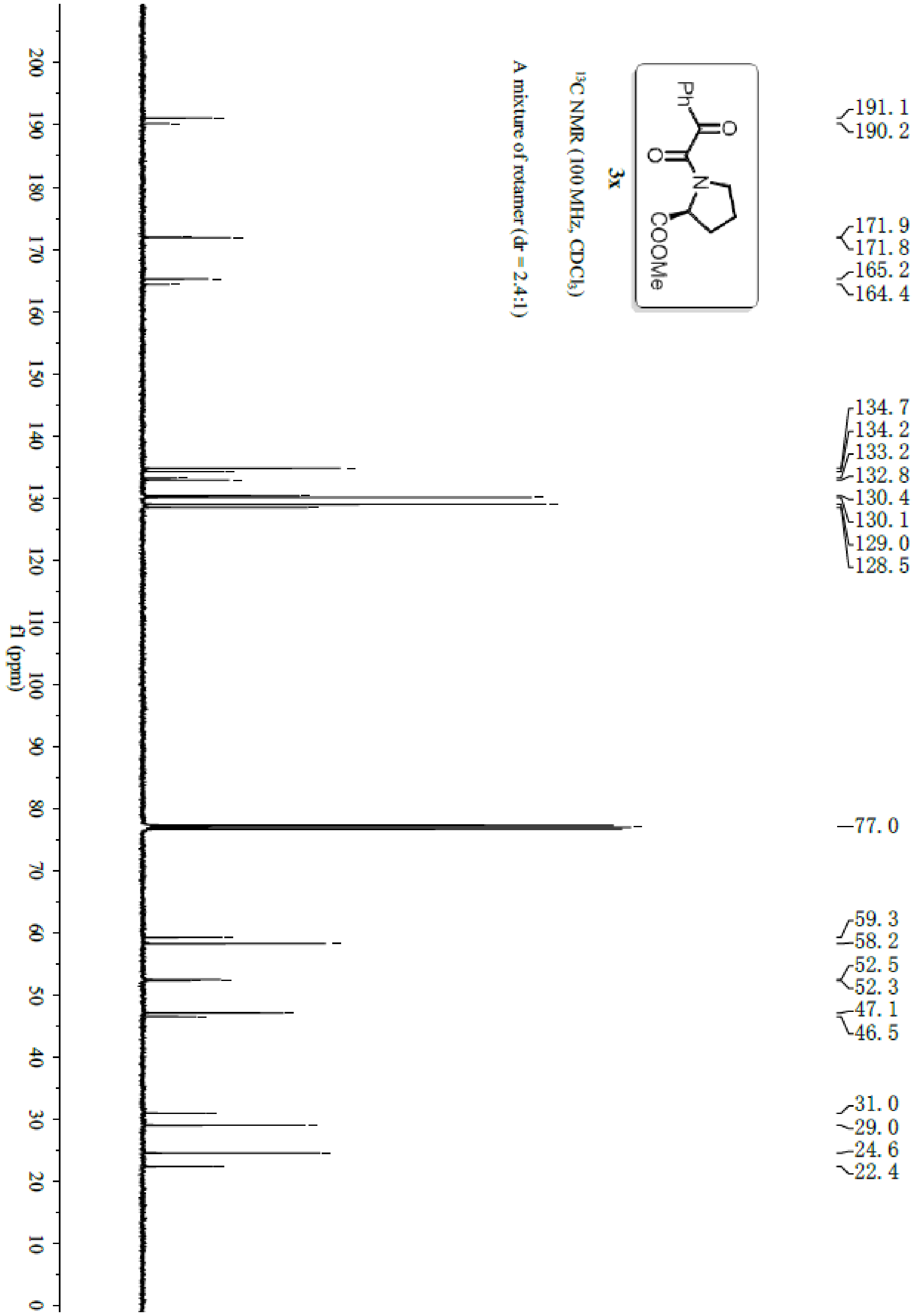

Supplementary Figure 103. ^{13}C NMR (100 MHz, $CDCl_3$) spectra of compound **3x**.

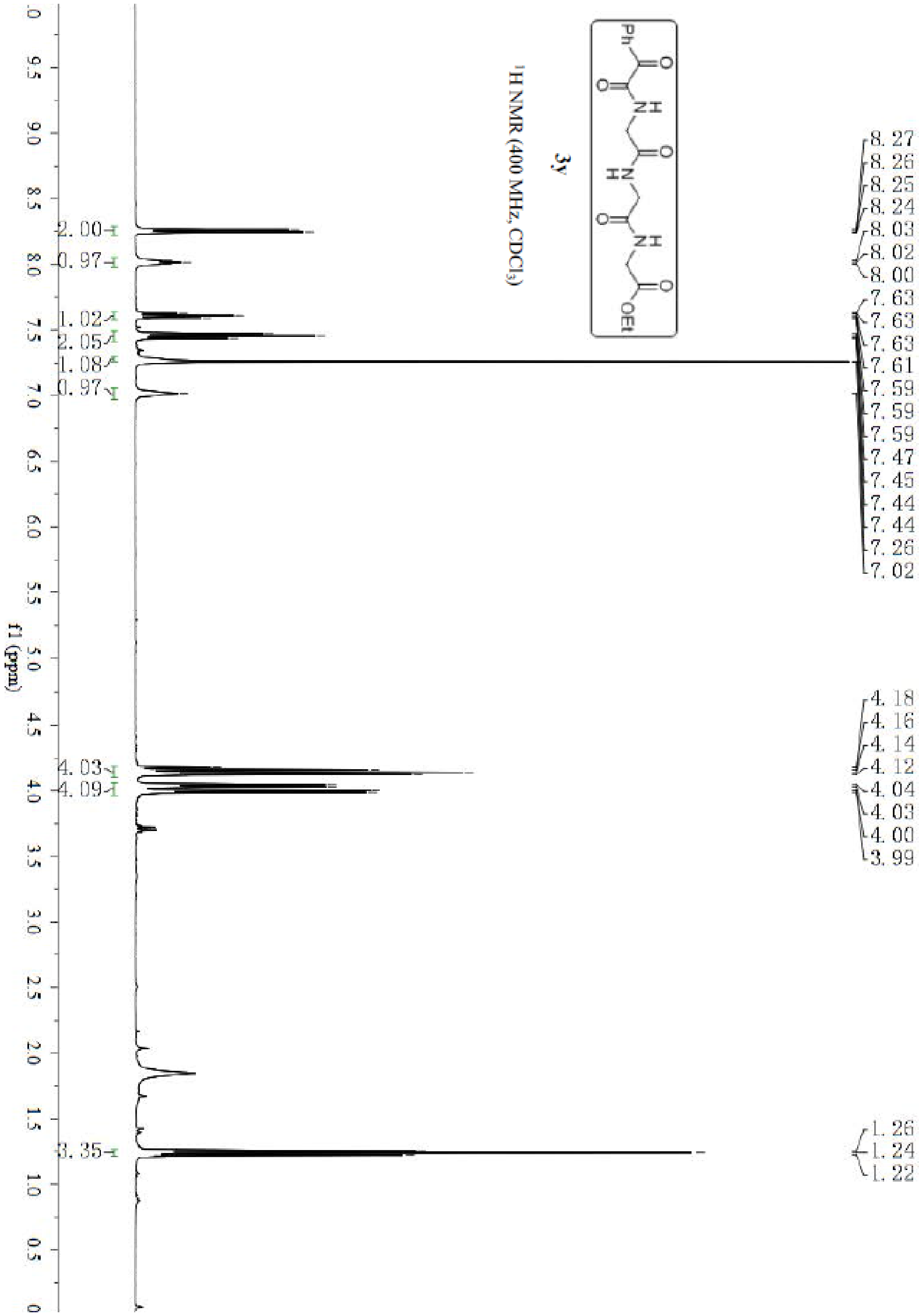

Supplementary Figure 104. ^{1}H NMR (400 MHz, $CDCl_3$) spectra of compound **3y**.

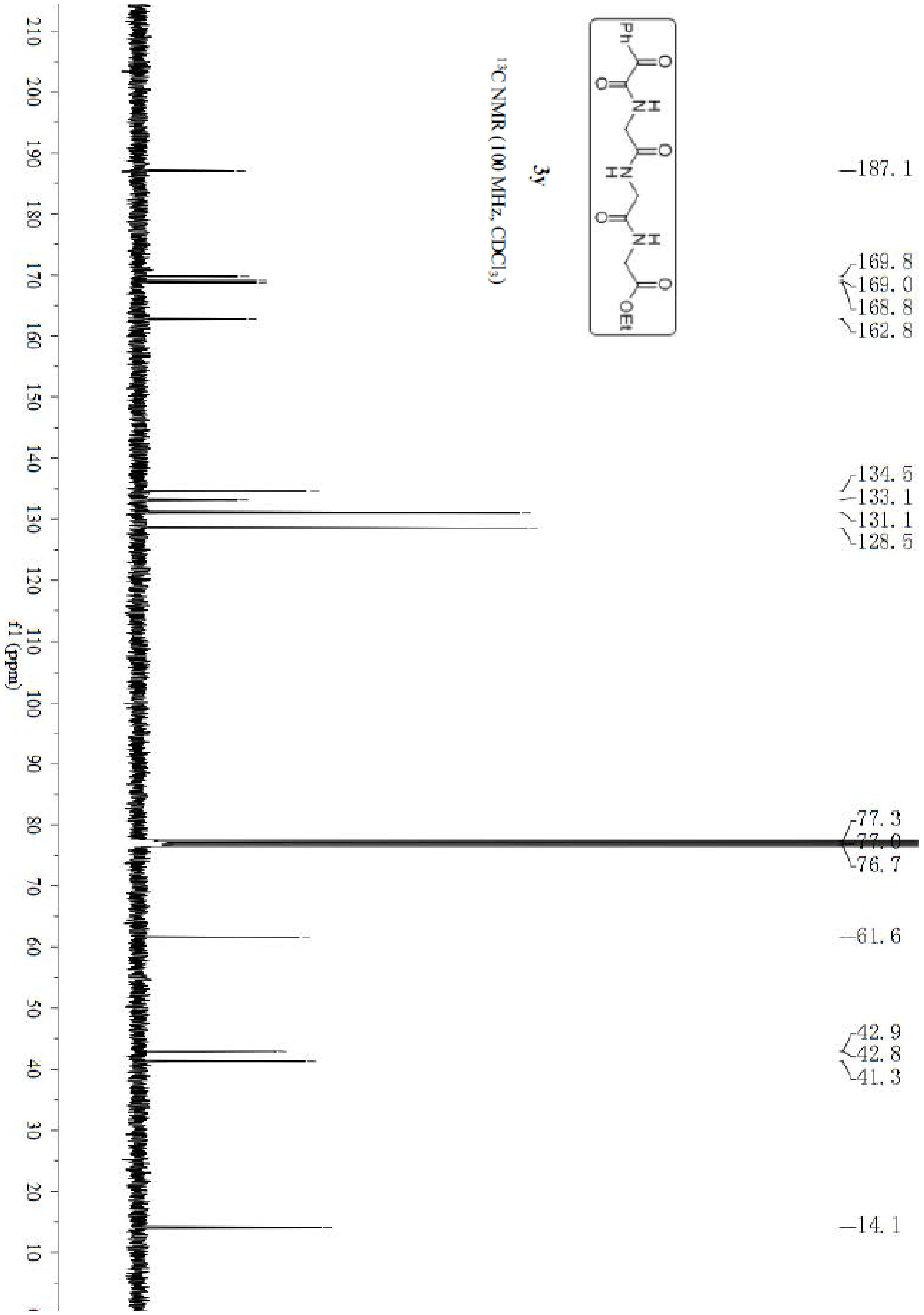

Supplementary Figure 105. ^{13}C NMR (100 MHz, $CDCl_3$) spectra of compound **3y**.

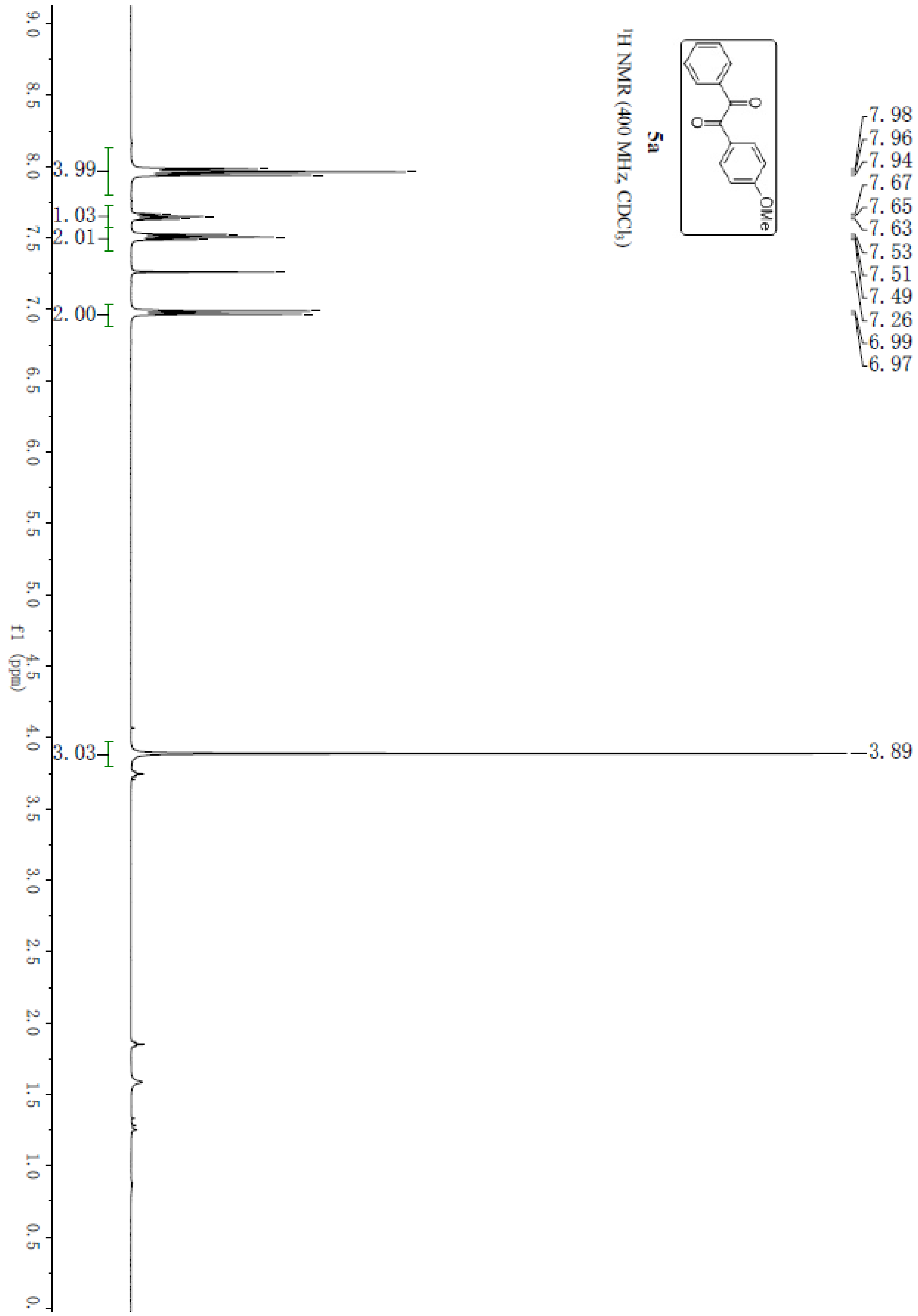

Supplementary Figure 106. ^{1}H NMR (400 MHz, $CDCl_3$) spectra of compound **5a**.

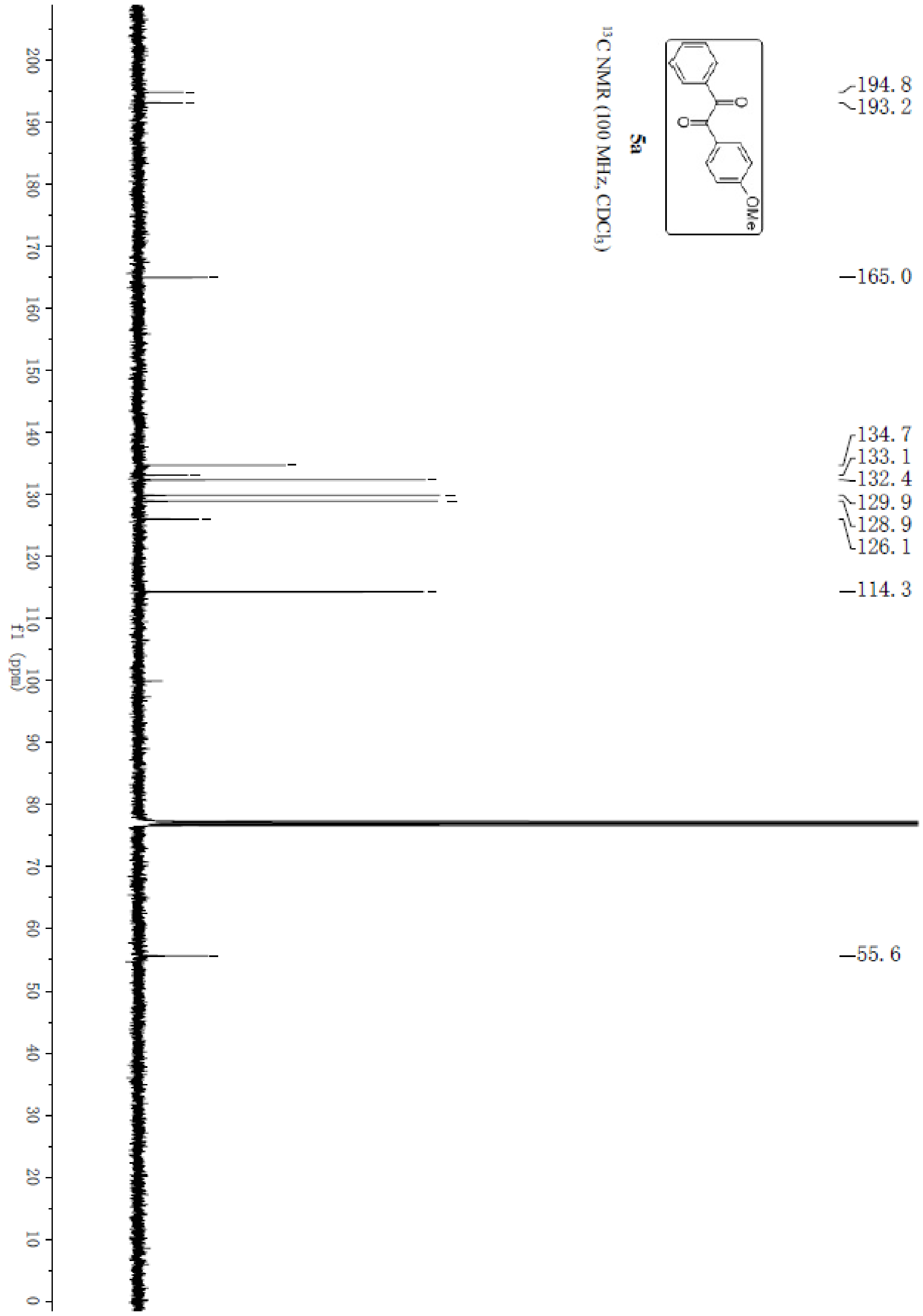

Supplementary Figure 107. ^{13}C NMR (100 MHz, $CDCl_3$) spectra of compound **5a**.

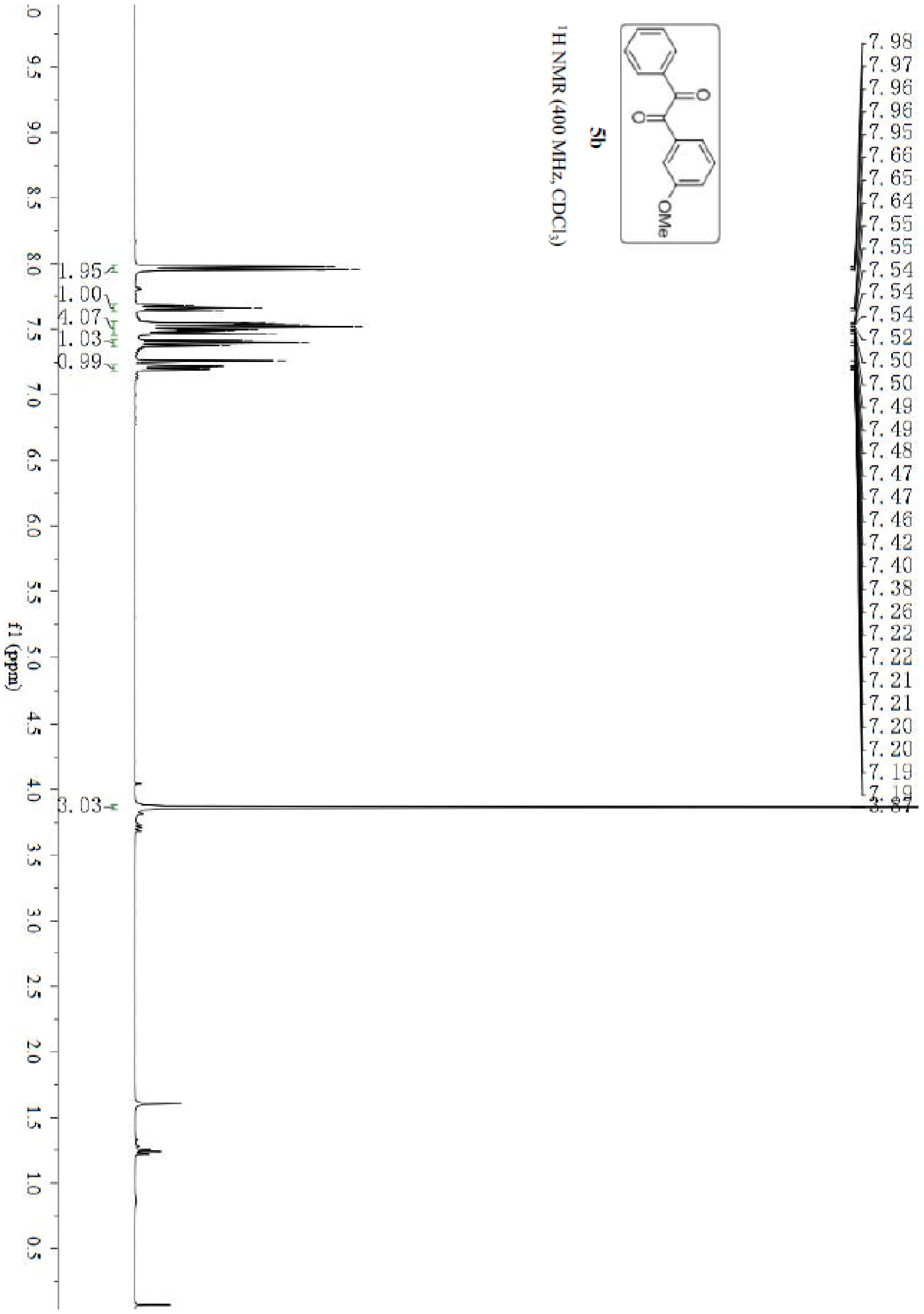

Supplementary Figure 108. ^{1}H NMR (400 MHz, $CDCl_3$) spectra of compound **5b**.

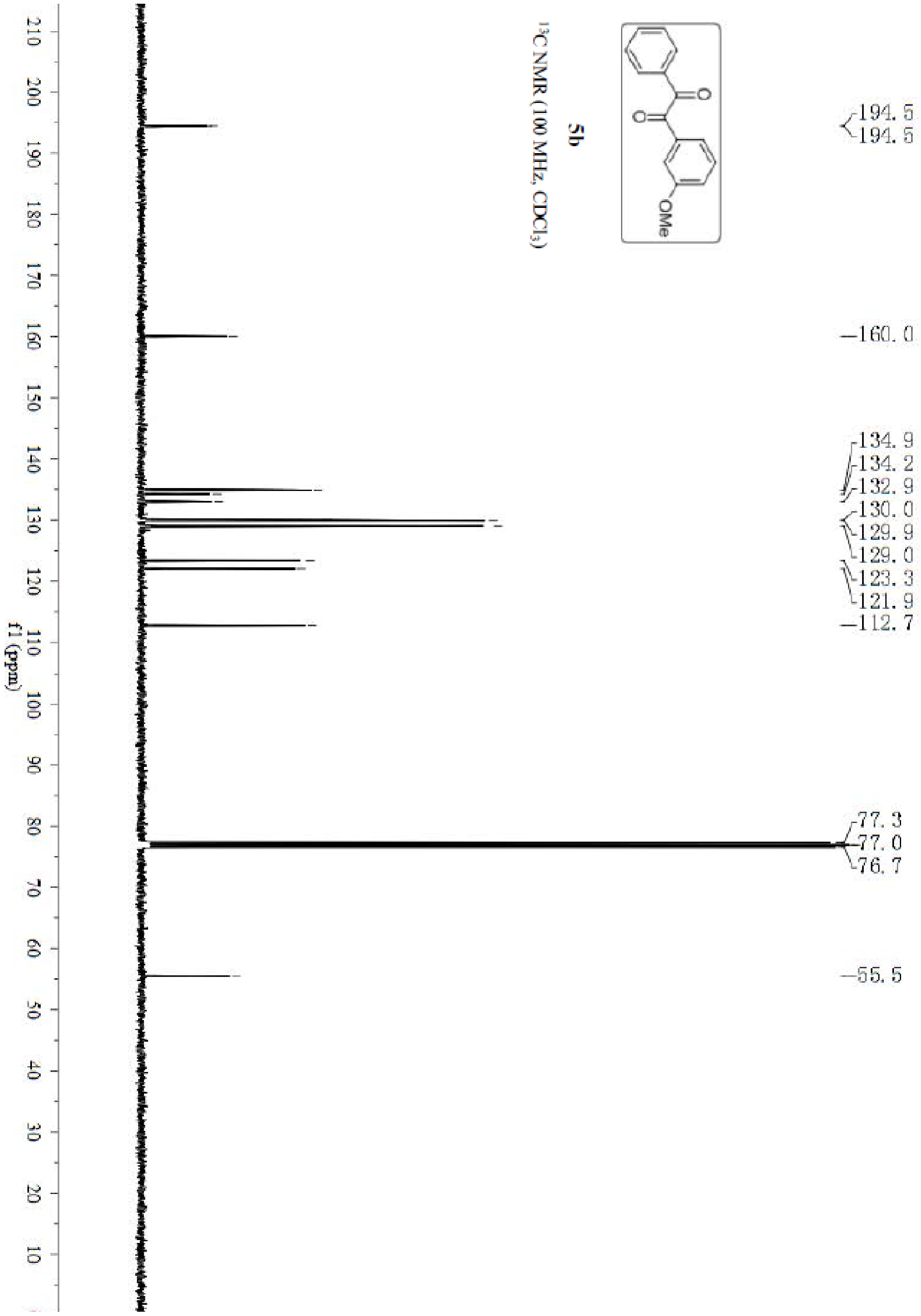

Supplementary Figure 109. ^{13}C NMR (100 MHz, $CDCl_3$) spectra of compound **5b**.

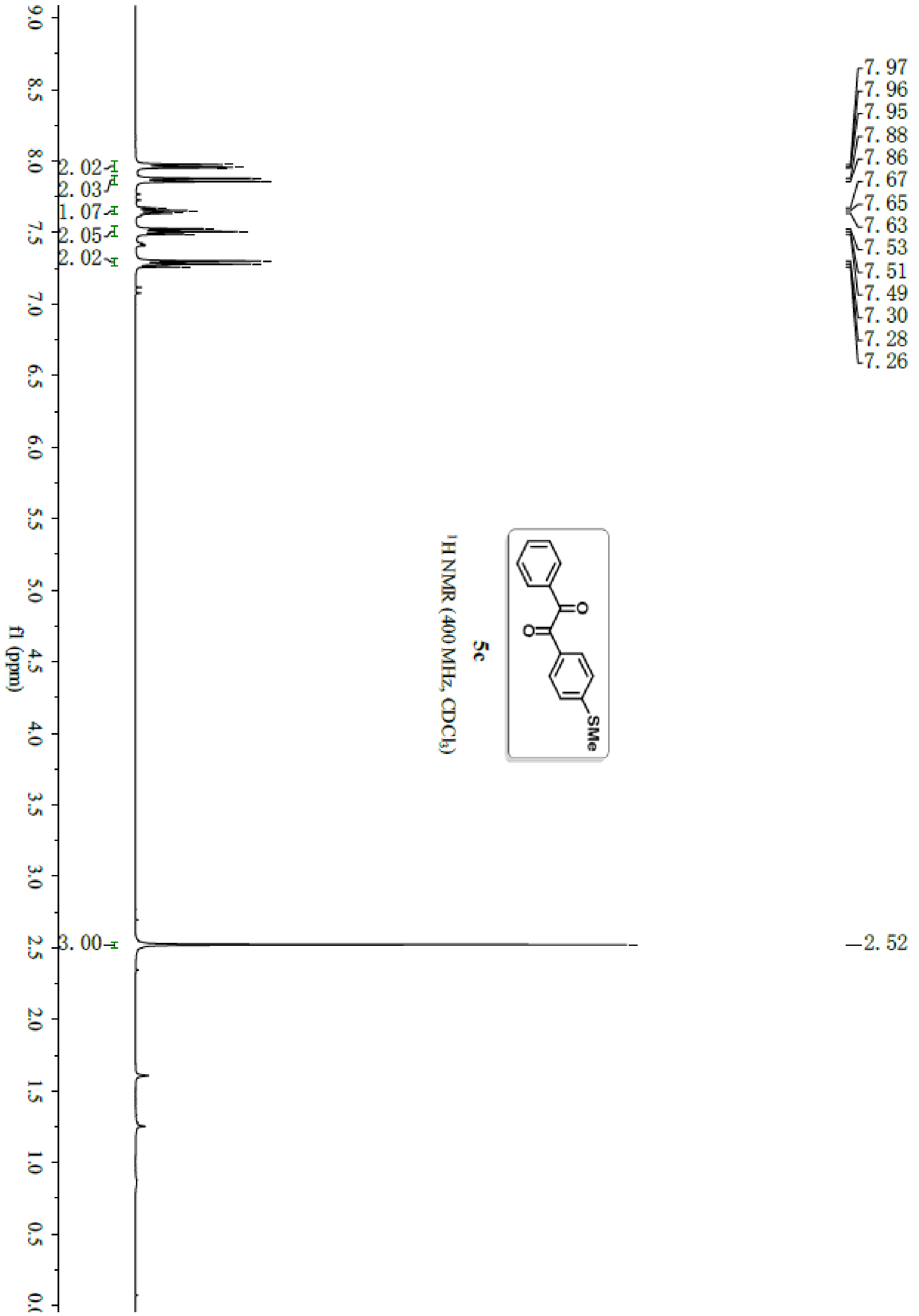

Supplementary Figure 110. ^{1}H NMR (400 MHz, $CDCl_3$) spectra of compound **5c**.

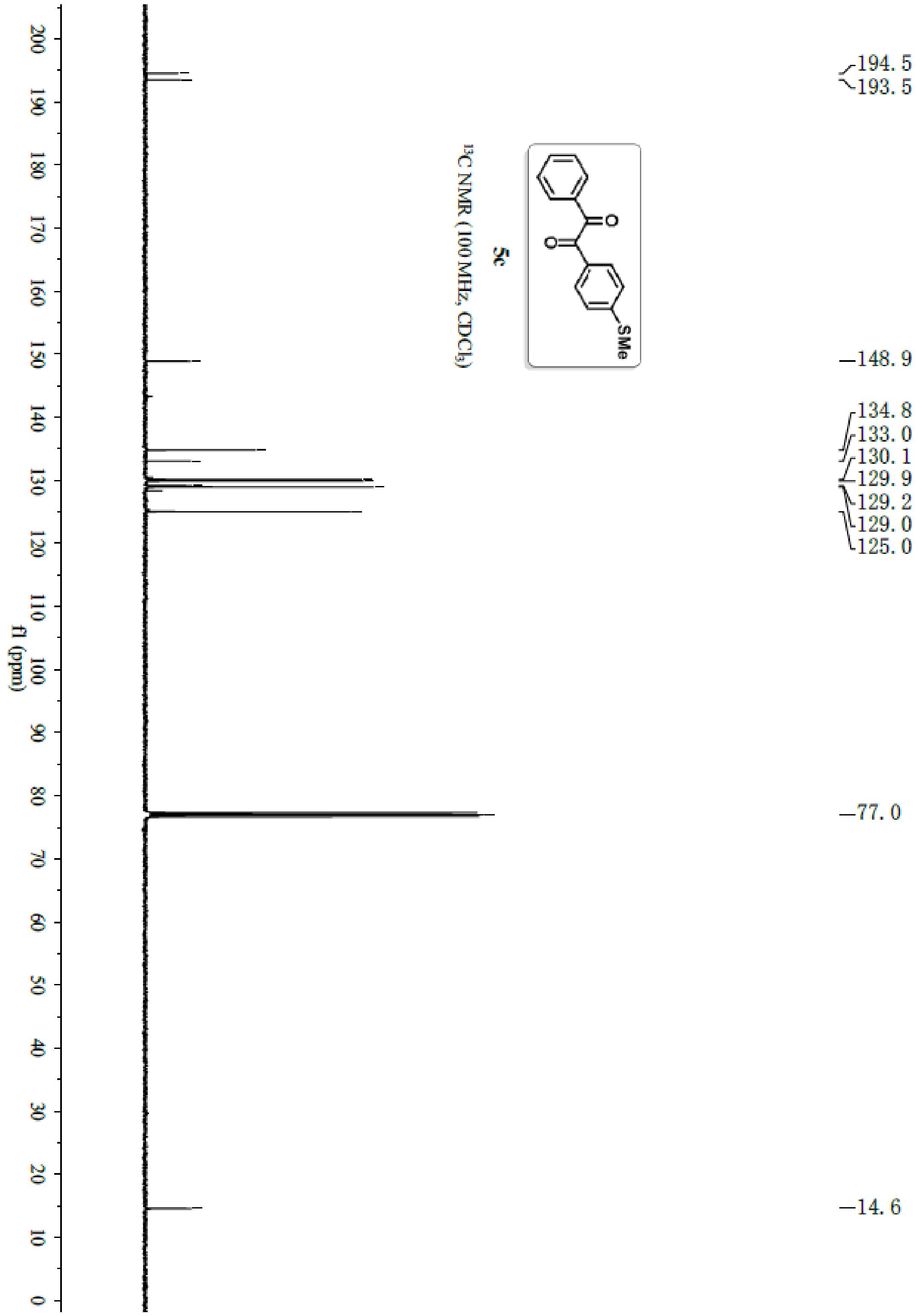

Supplementary Figure 111. ^{13}C NMR (100 MHz, $CDCl_3$) spectra of compound **5c**.

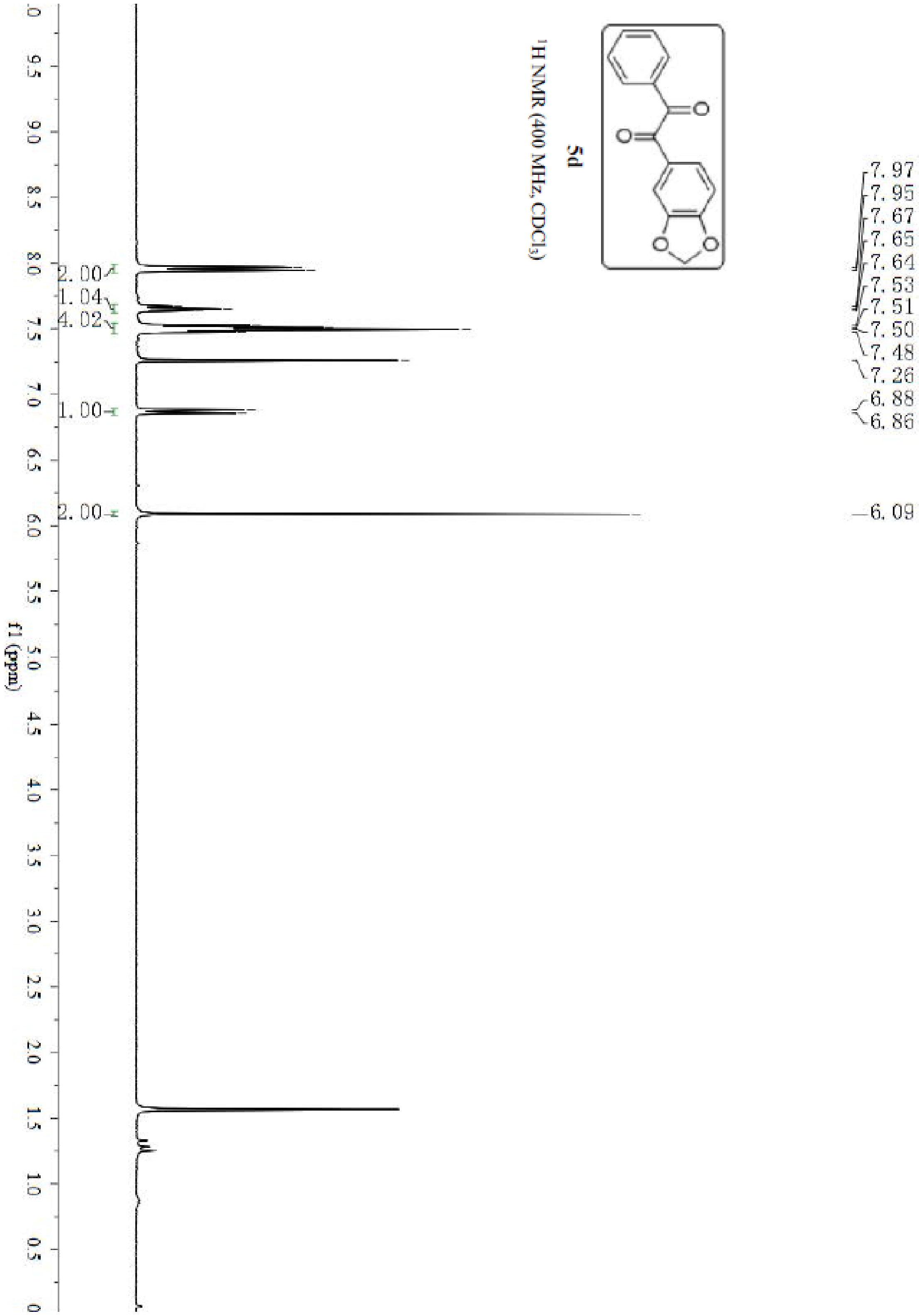

Supplementary Figure 112. ^{1}H NMR (400 MHz, $CDCl_3$) spectra of compound **5d**.

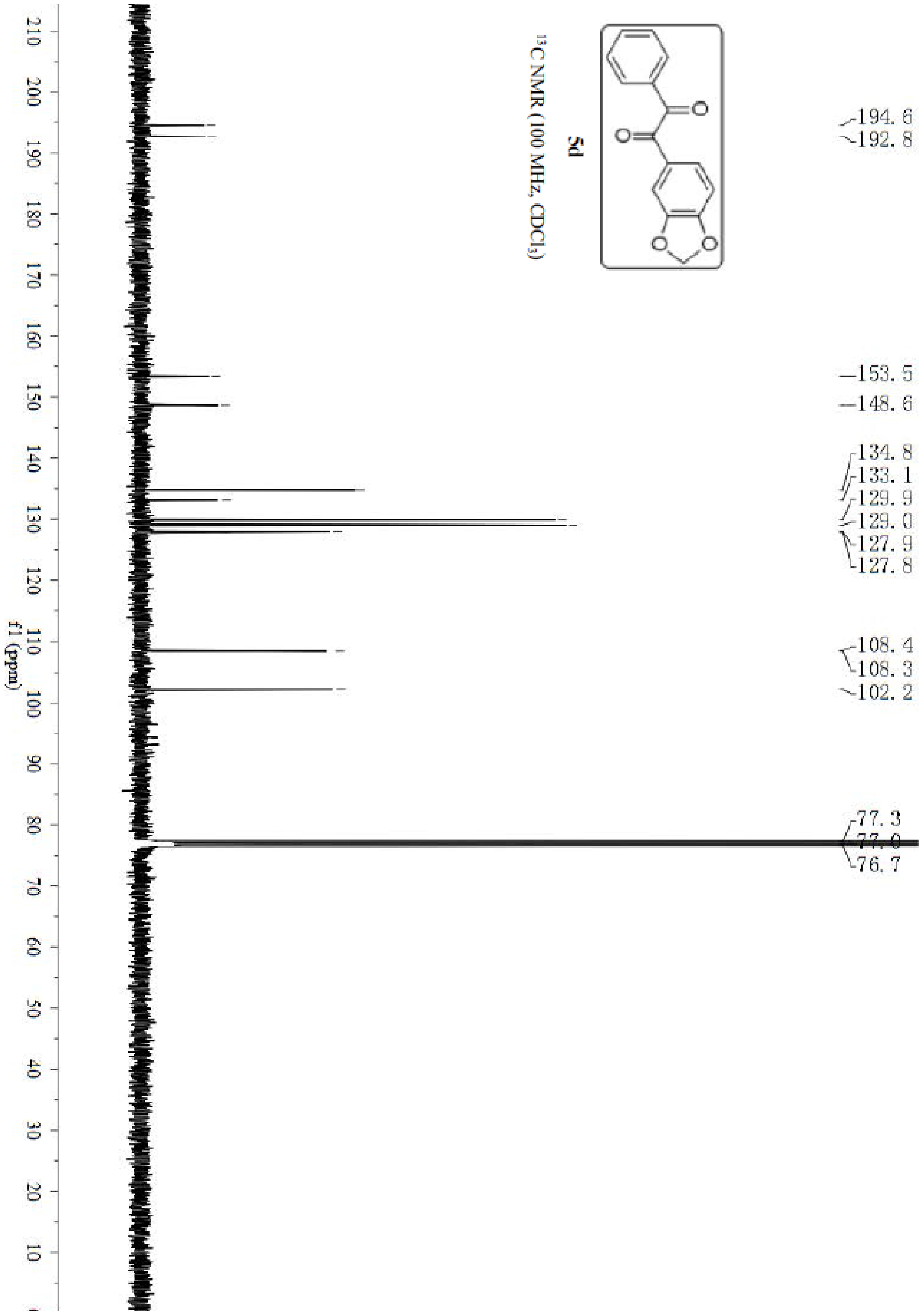

Supplementary Figure 113. ^{13}C NMR (100 MHz, $CDCl_3$) spectra of compound **5d**.

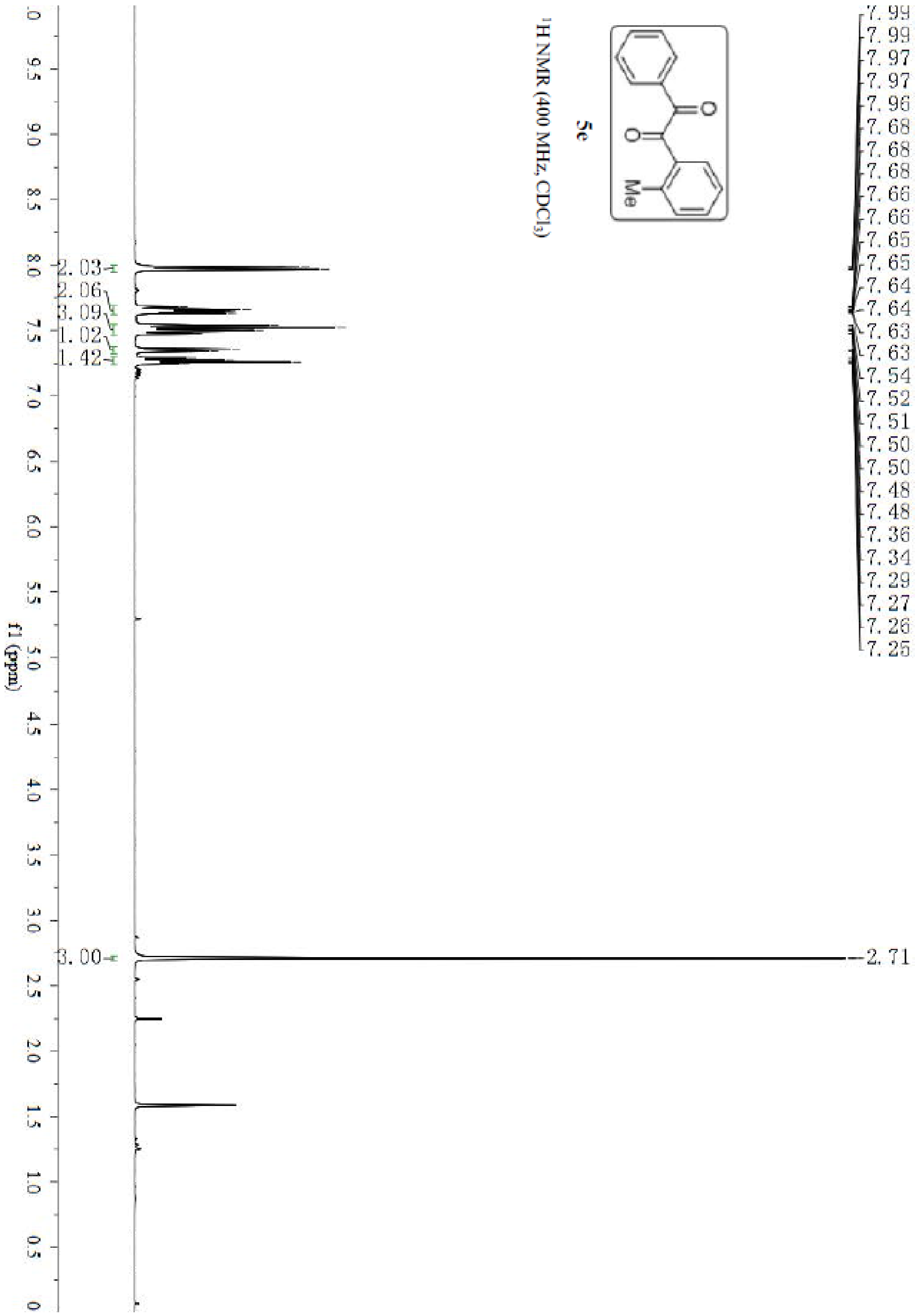

Supplementary Figure 114. ^{1}H NMR (400 MHz, $CDCl_3$) spectra of compound **5e**.

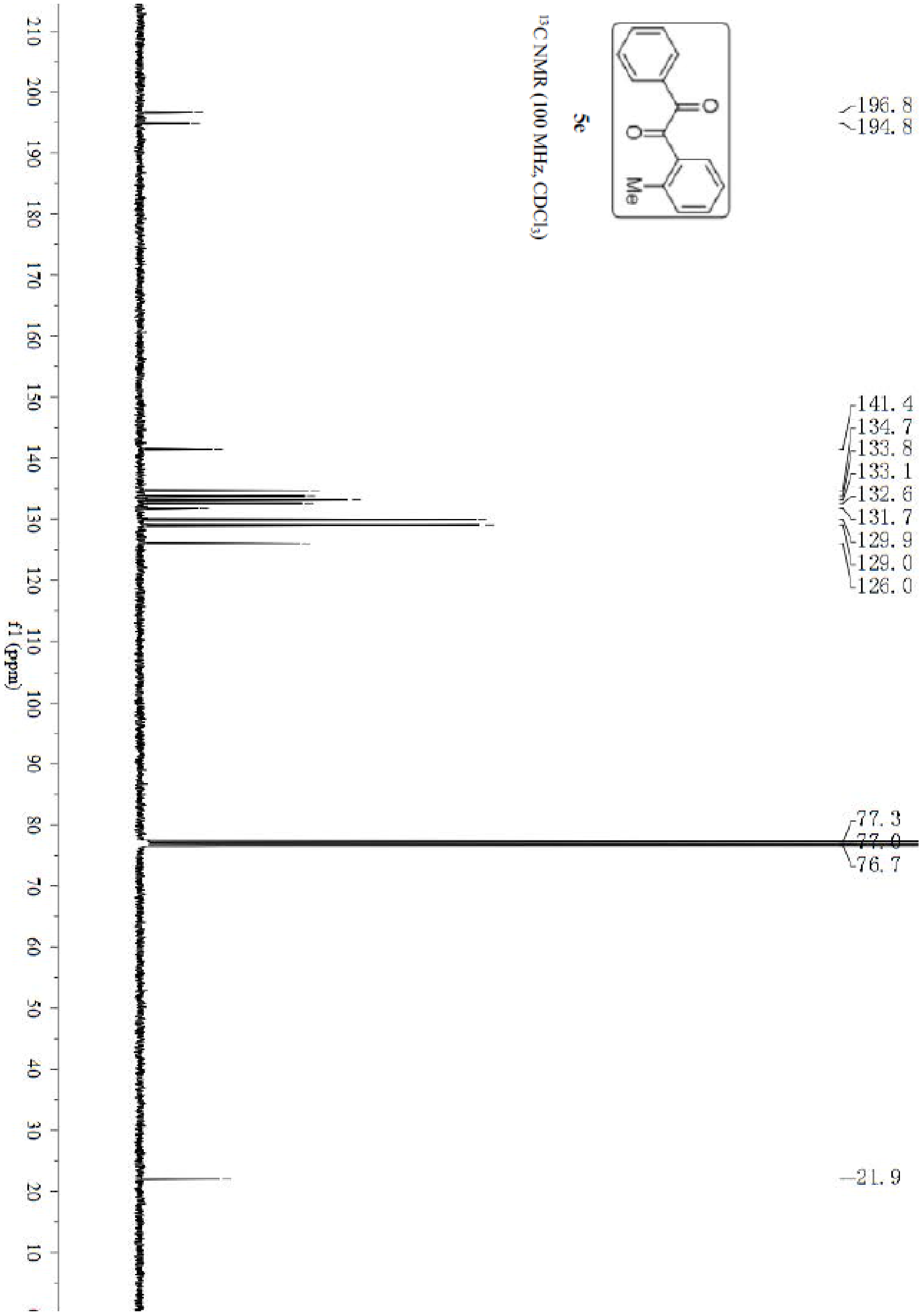

Supplementary Figure 115. ^{13}C NMR (100 MHz, $CDCl_3$) spectra of compound **5e**.

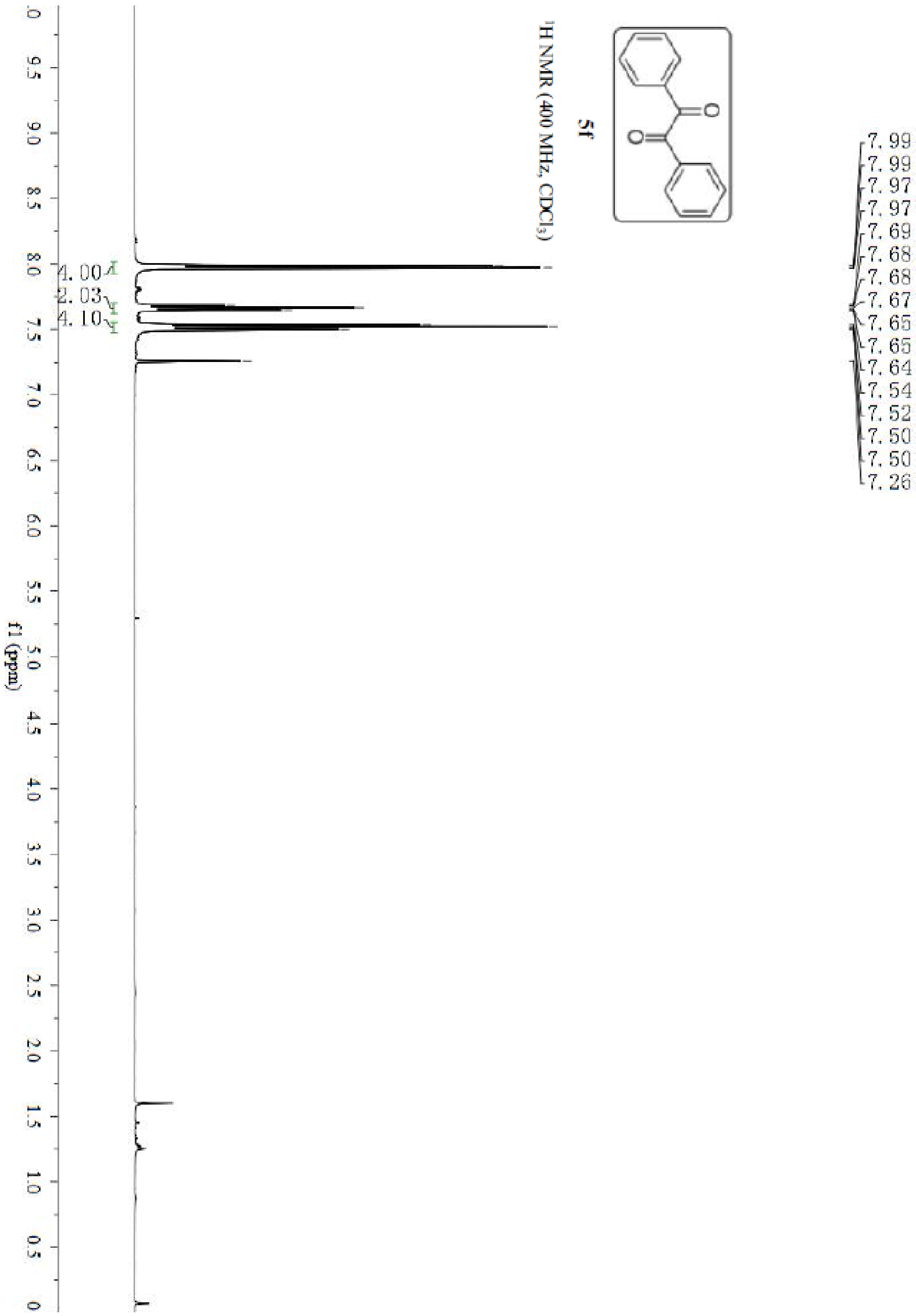

Supplementary Figure 116. ^{1}H NMR (400 MHz, $CDCl_3$) spectra of compound **5f**.

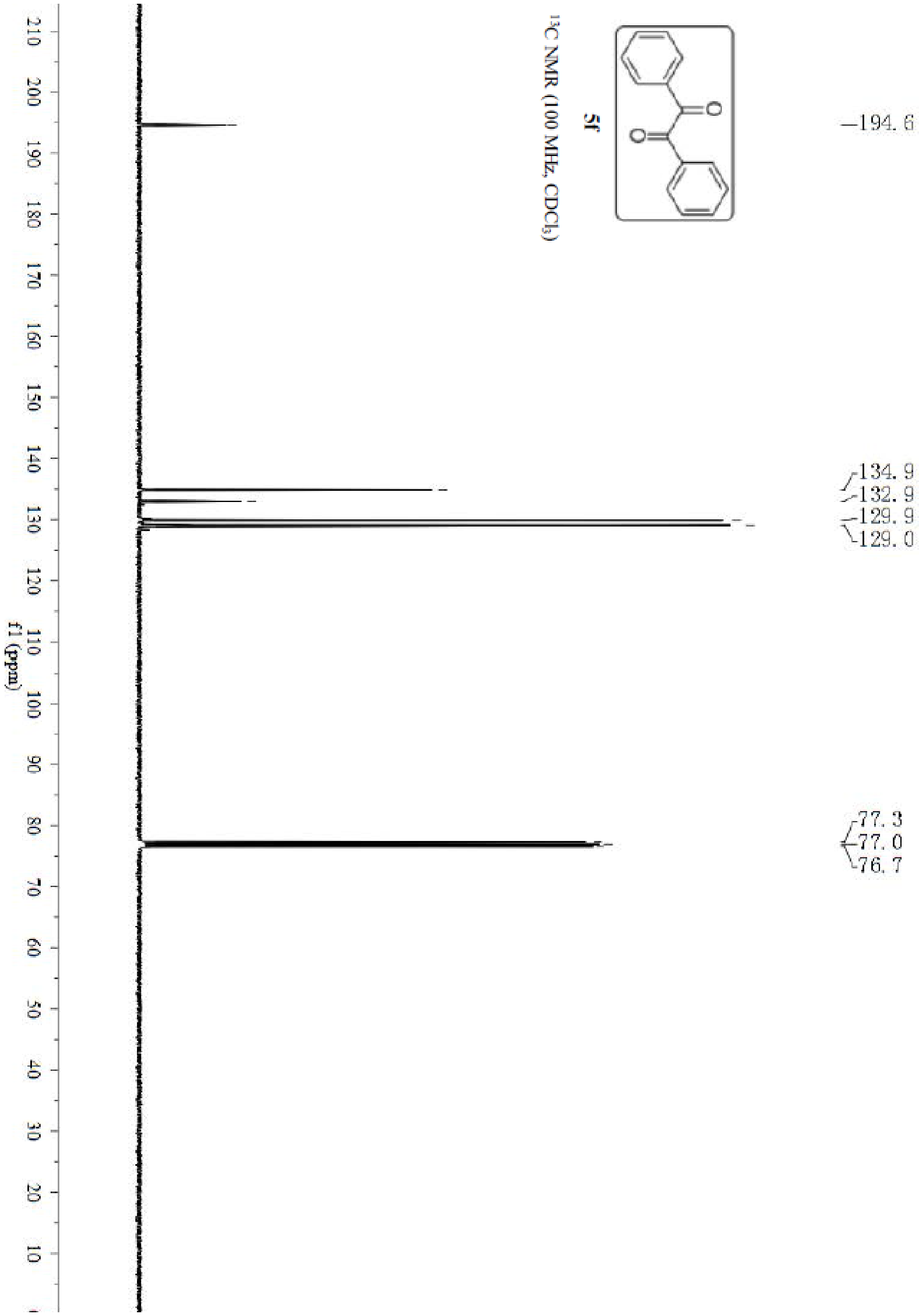

Supplementary Figure 117. ^{13}C NMR (100 MHz, $CDCl_3$) spectra of compound **5f**.

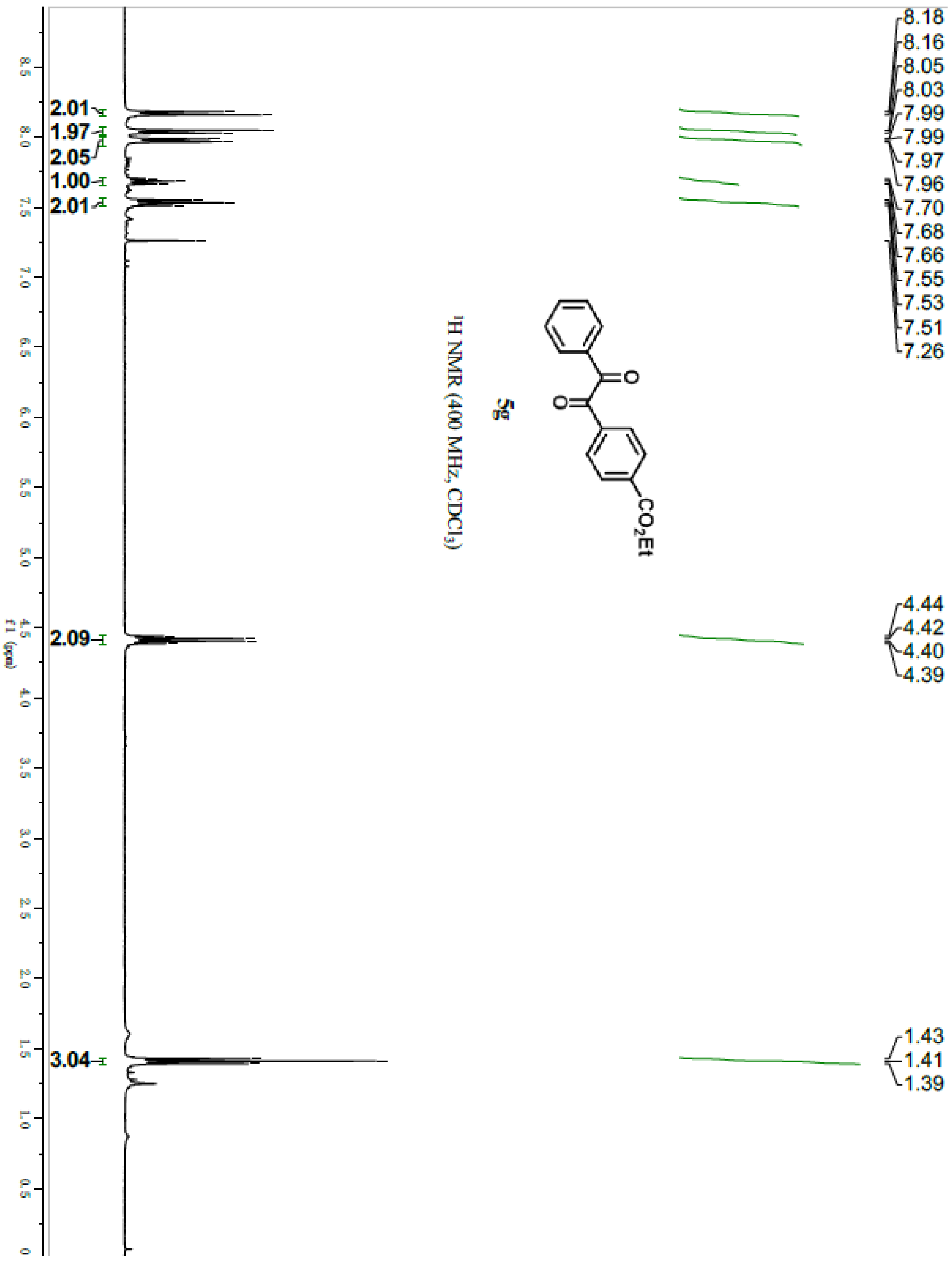

Supplementary Figure 118. ^{1}H NMR (400 MHz, $CDCl_3$) spectra of compound **5g**.

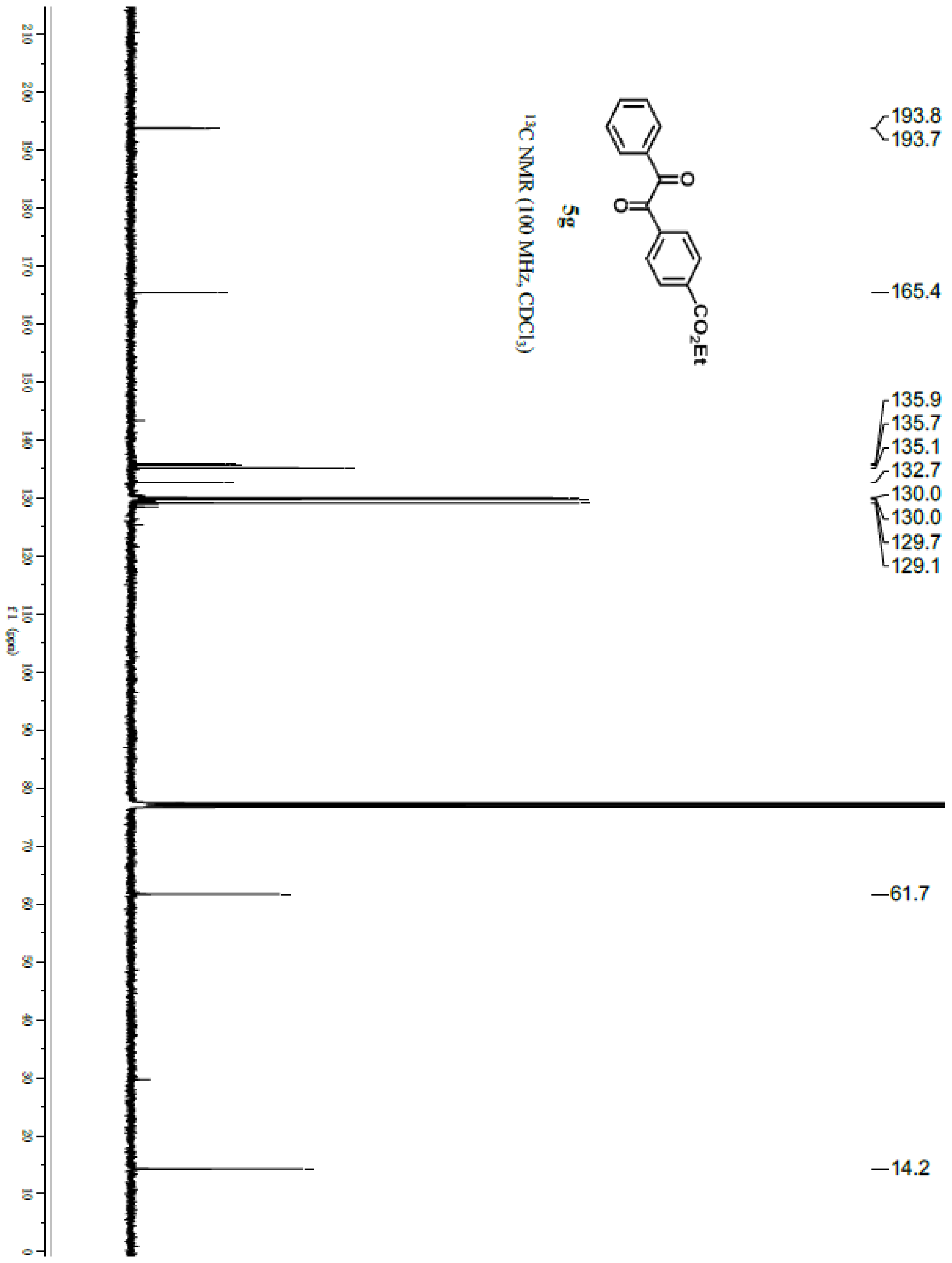

Supplementary Figure 119. ^{13}C NMR (100 MHz, $CDCl_3$) spectra of compound **5g**.

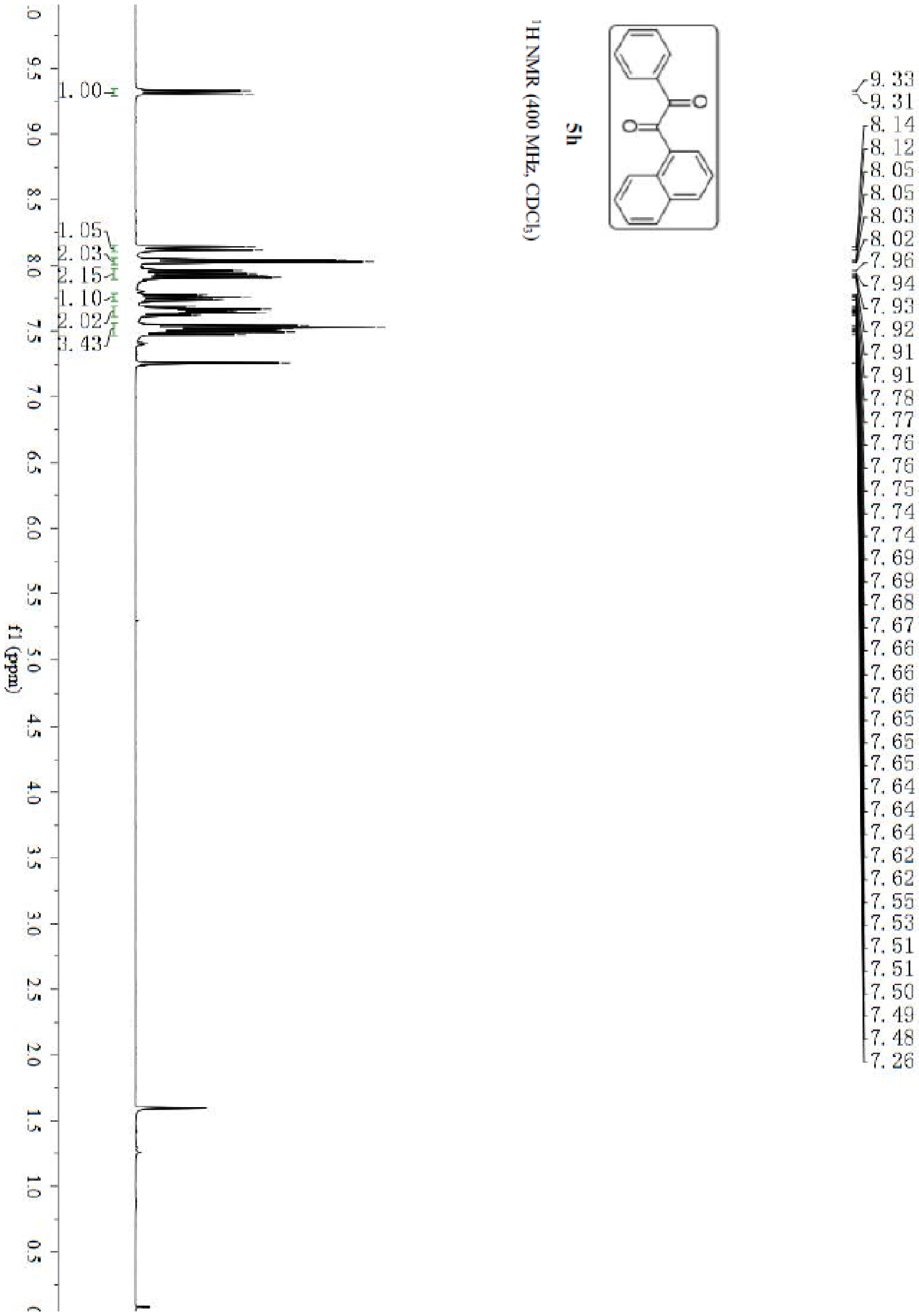

Supplementary Figure 120. ^{1}H NMR (400 MHz, $CDCl_3$) spectra of compound **5h**.

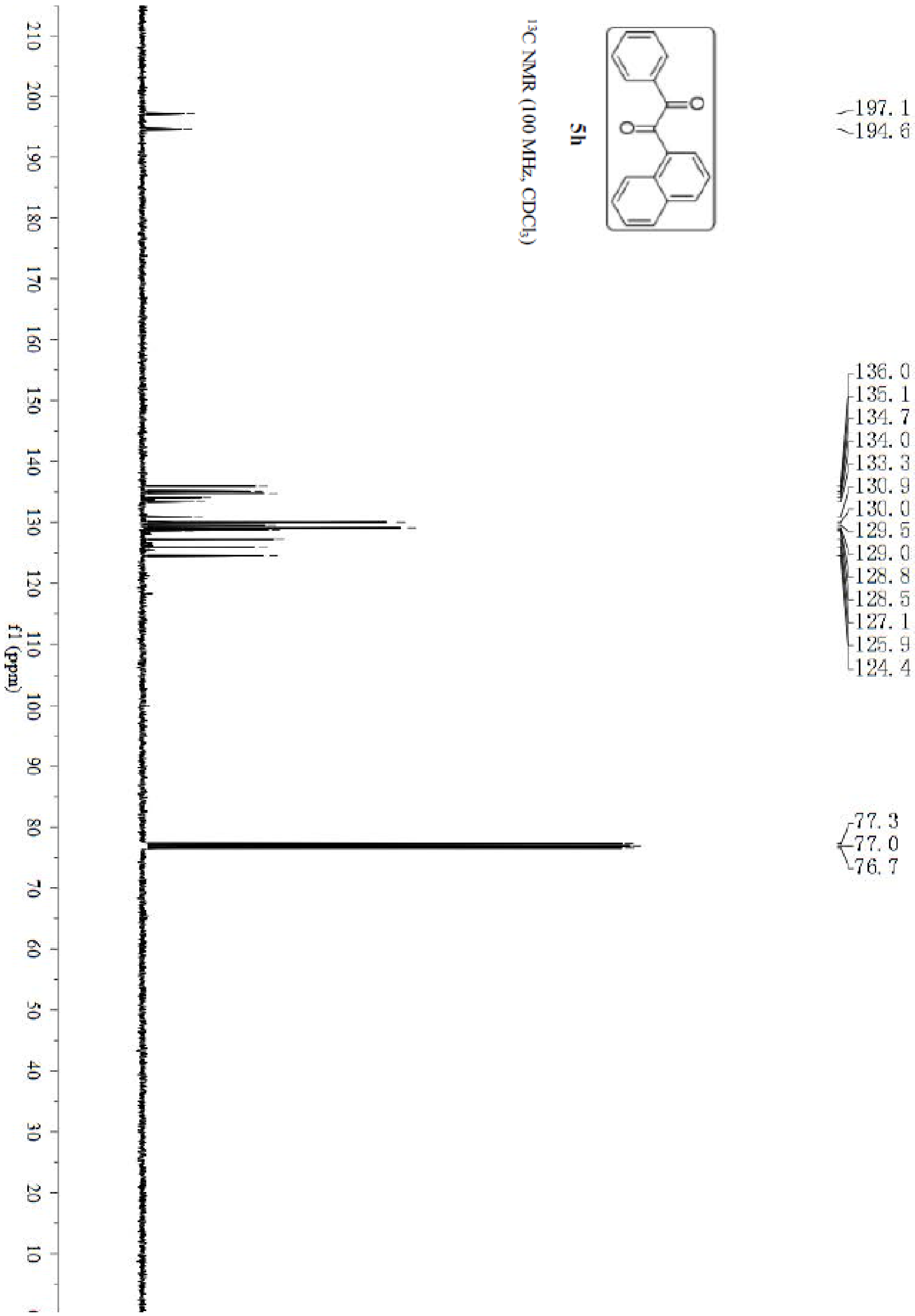

Supplementary Figure 121. ^{13}C NMR (100 MHz, $CDCl_3$) spectra of compound **5h**.

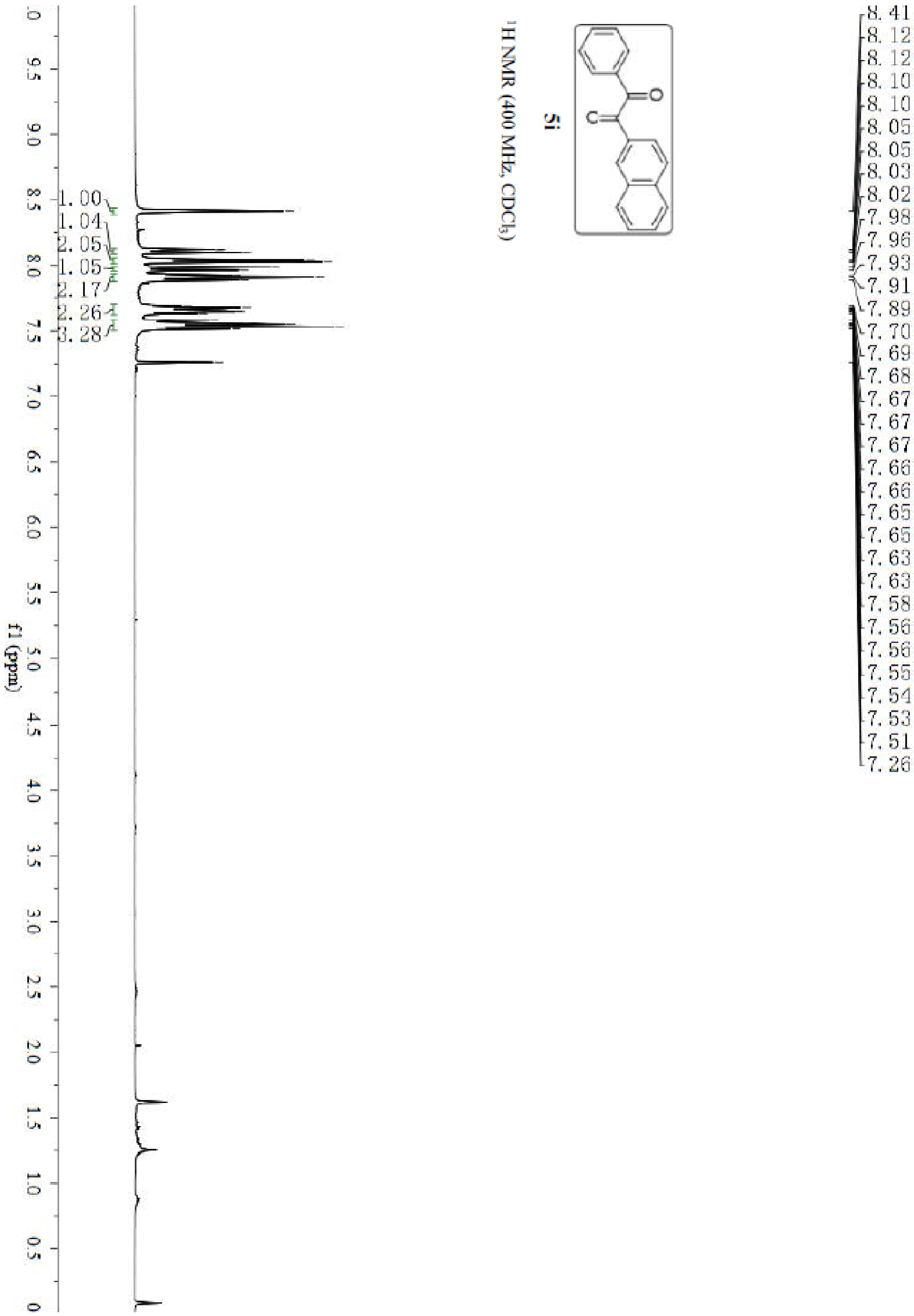

Supplementary Figure 122. ^{1}H NMR (400 MHz, $CDCl_3$) spectra of compound **5i**.

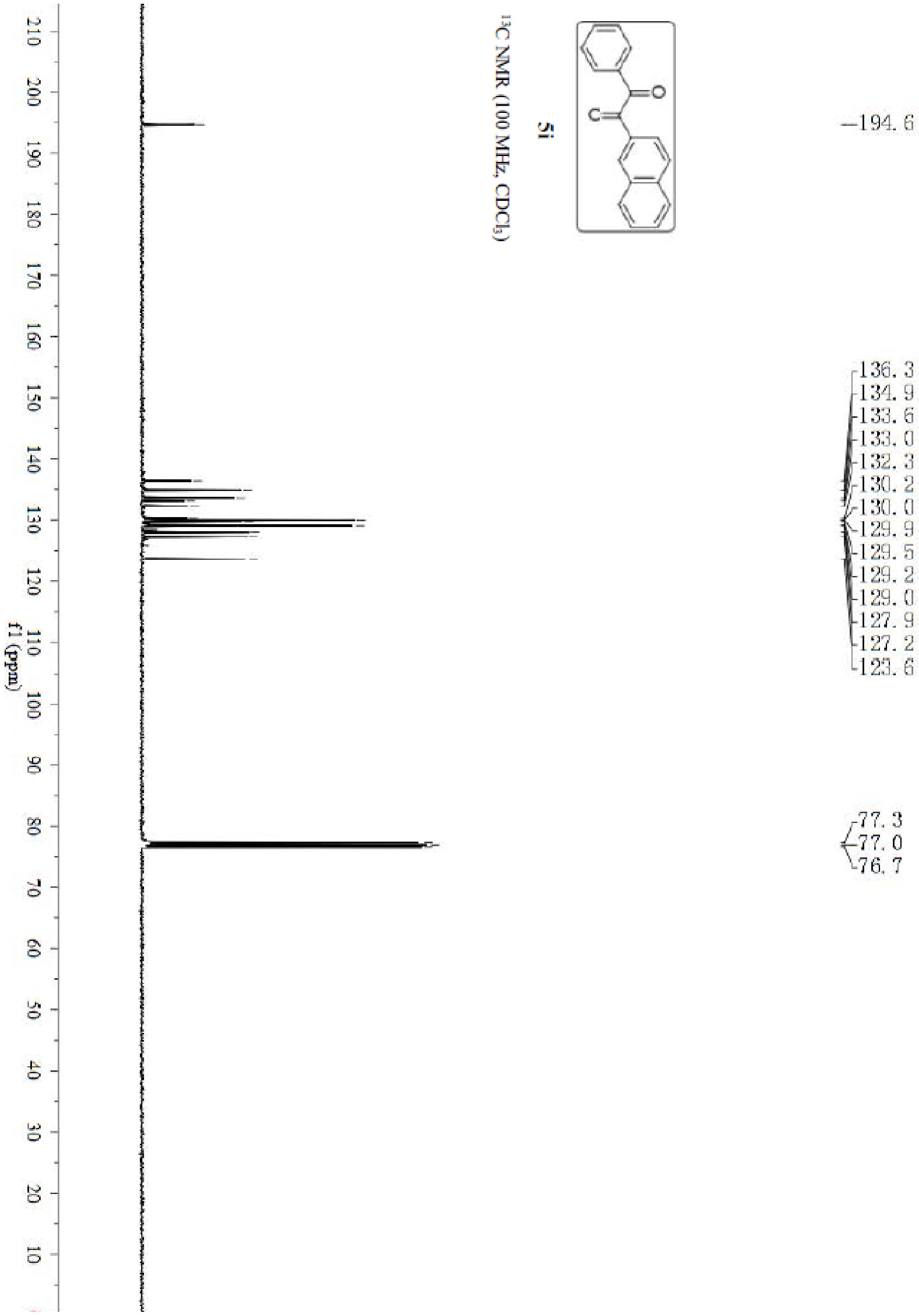

Supplementary Figure 123. ^{13}C NMR (100 MHz, $CDCl_3$) spectra of compound **5i**.

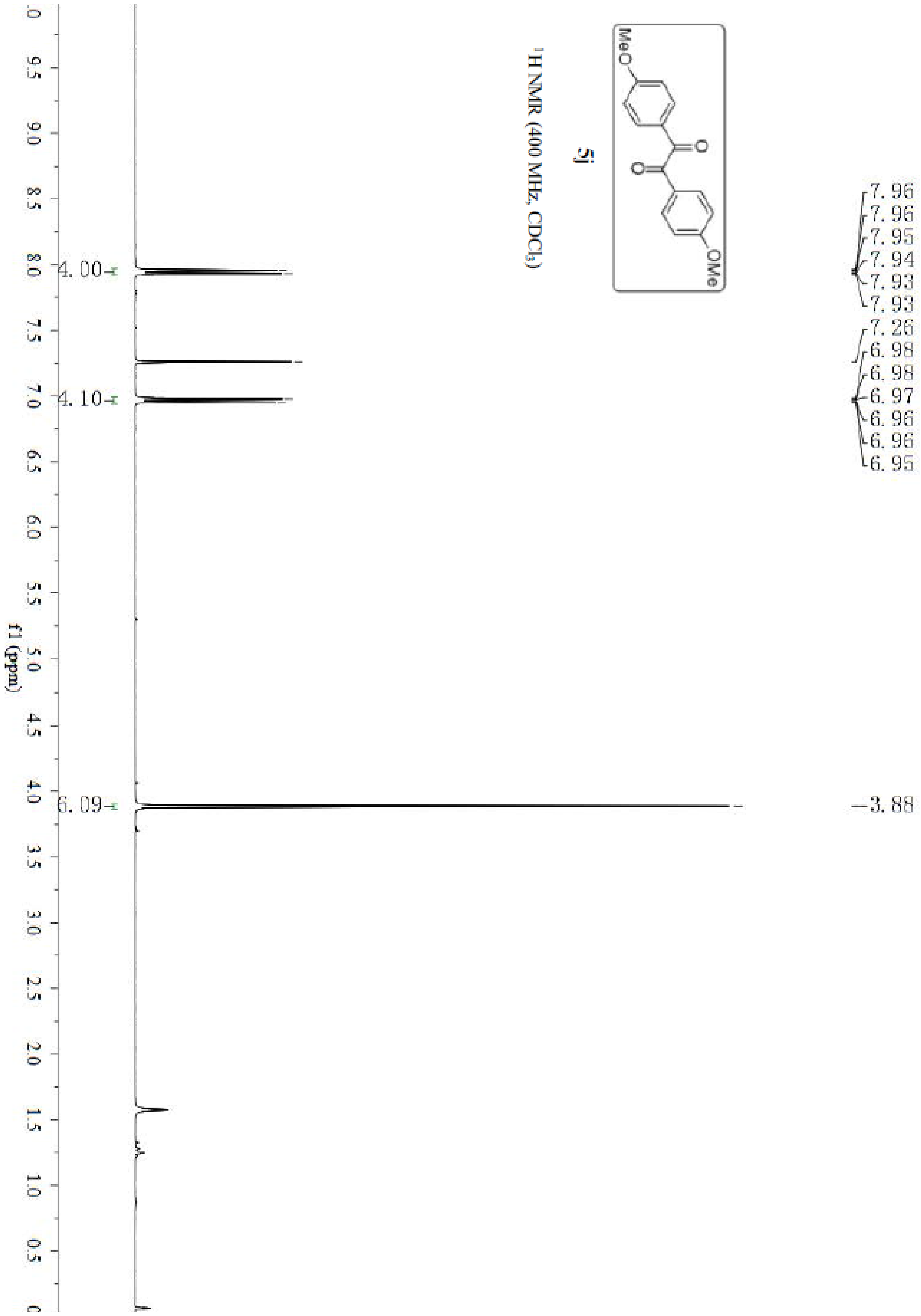

Supplementary Figure 124. ^{1}H NMR (400 MHz, $CDCl_3$) spectra of compound **5j**.

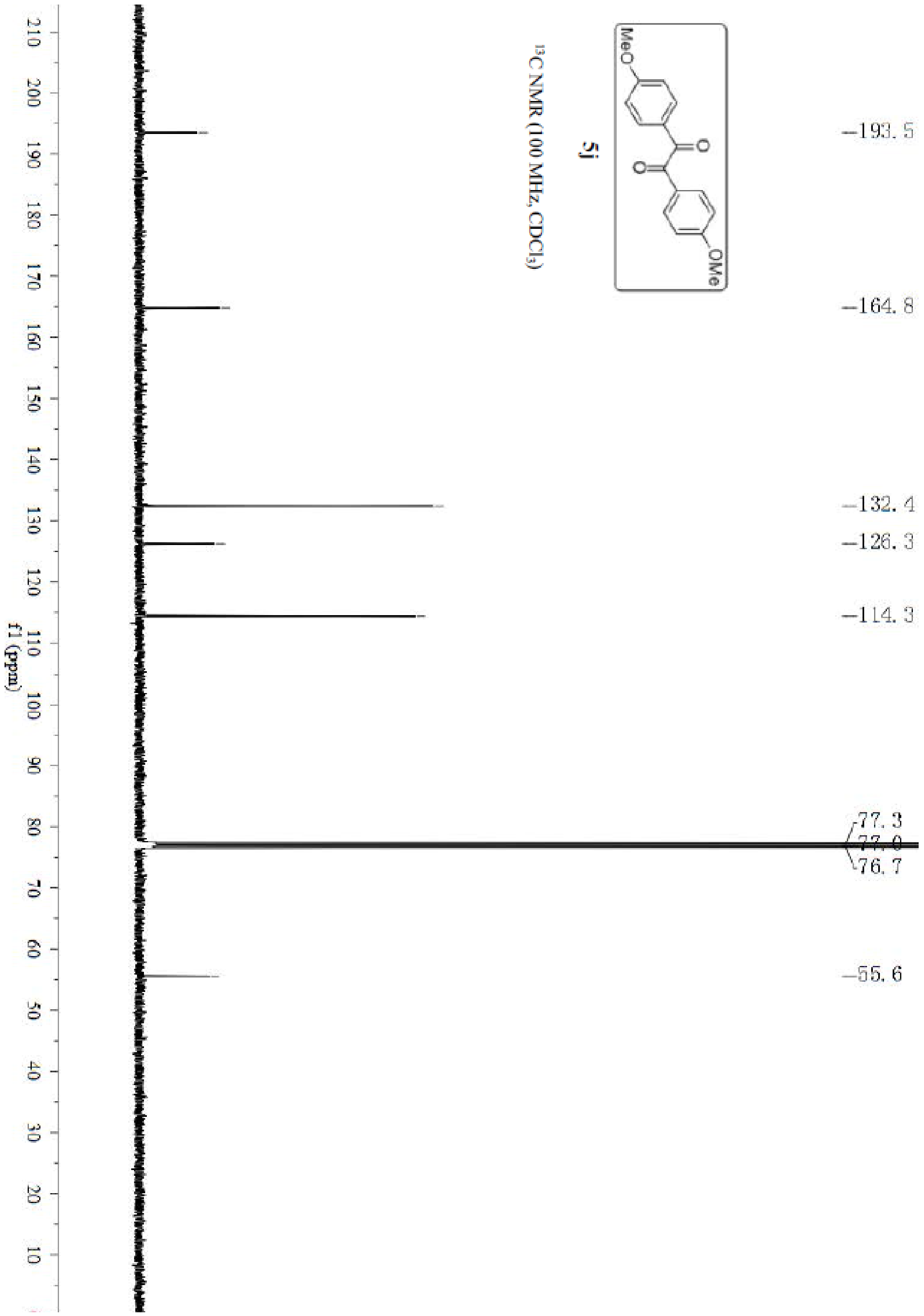

Supplementary Figure 125. ^{13}C NMR (100 MHz, $CDCl_3$) spectra of compound **5j**.

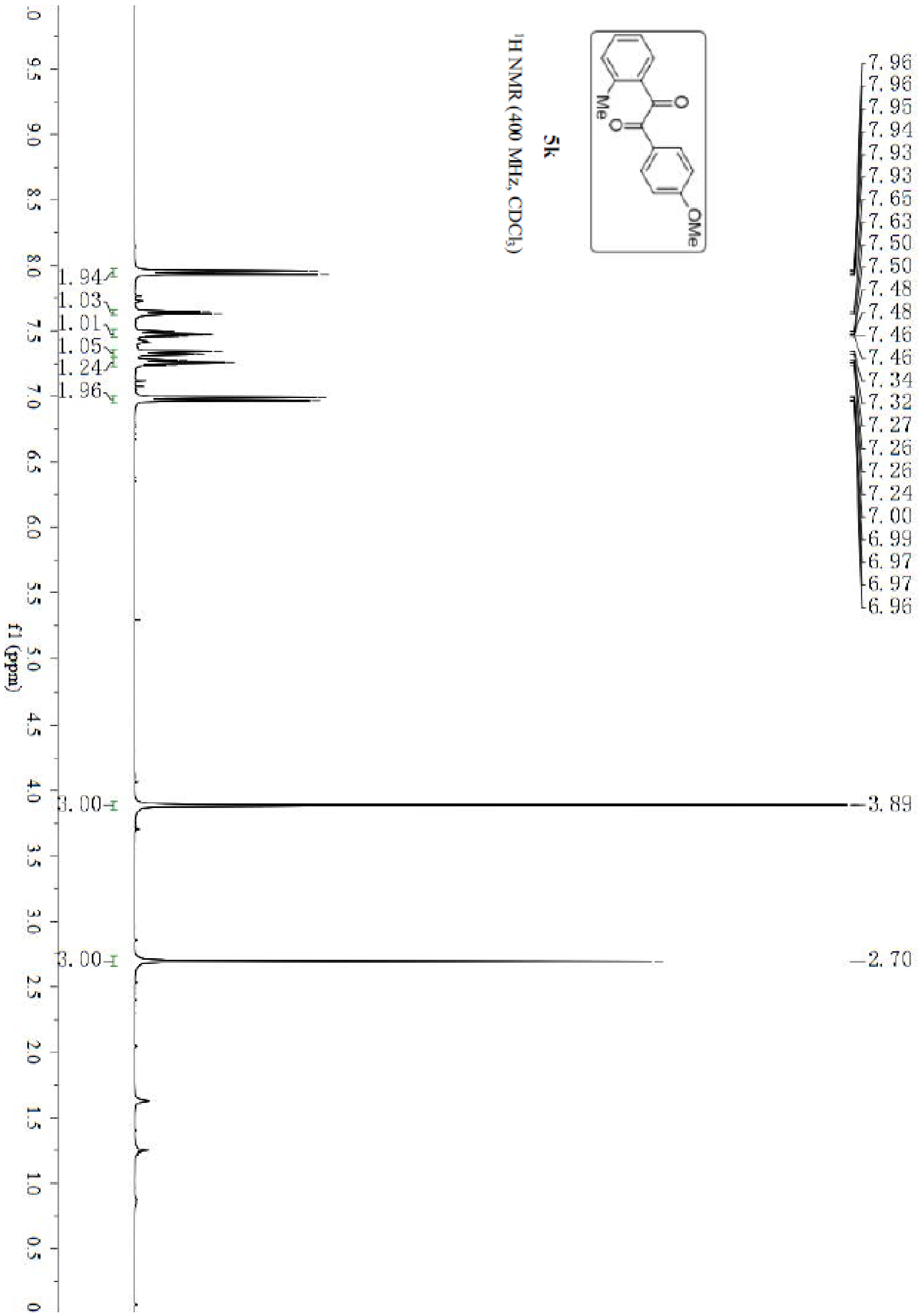

Supplementary Figure 126. ^{1}H NMR (400 MHz, $CDCl_3$) spectra of compound **5k**.

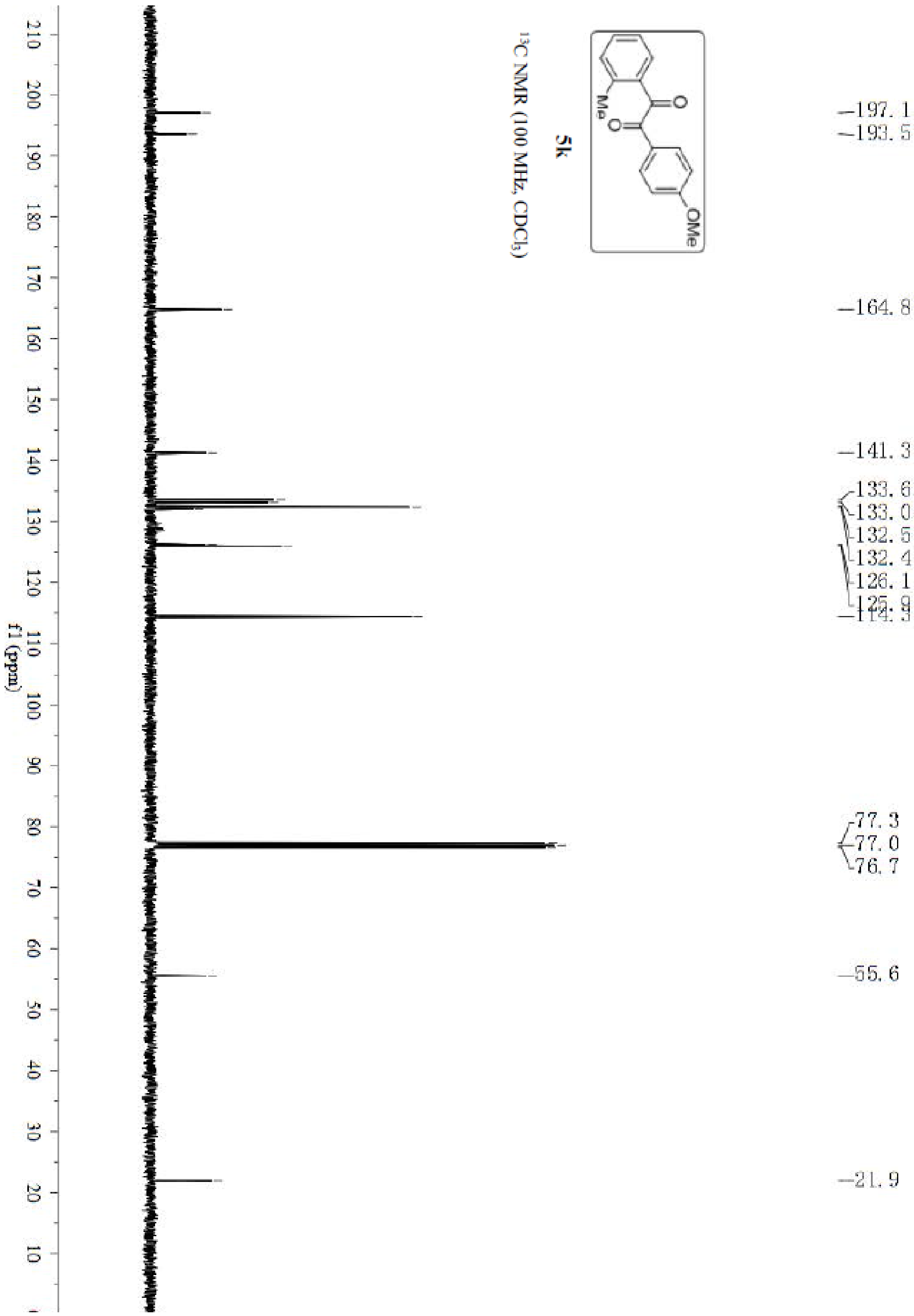

Supplementary Figure 127. ^{13}C NMR (100 MHz, $CDCl_3$) spectra of compound **5k**.

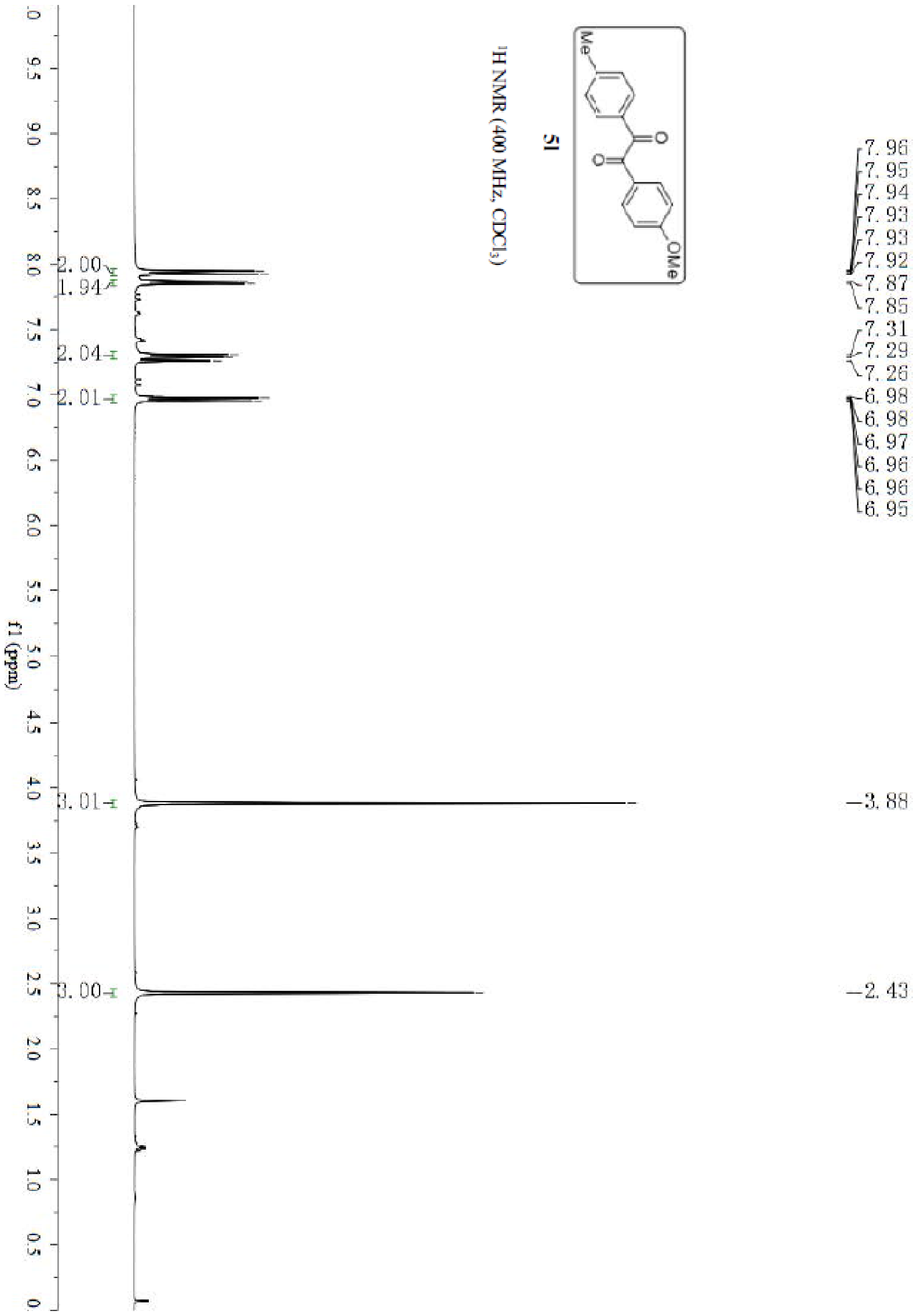

Supplementary Figure 128. ^{1}H NMR (400 MHz, $CDCl_3$) spectra of compound **5l**.

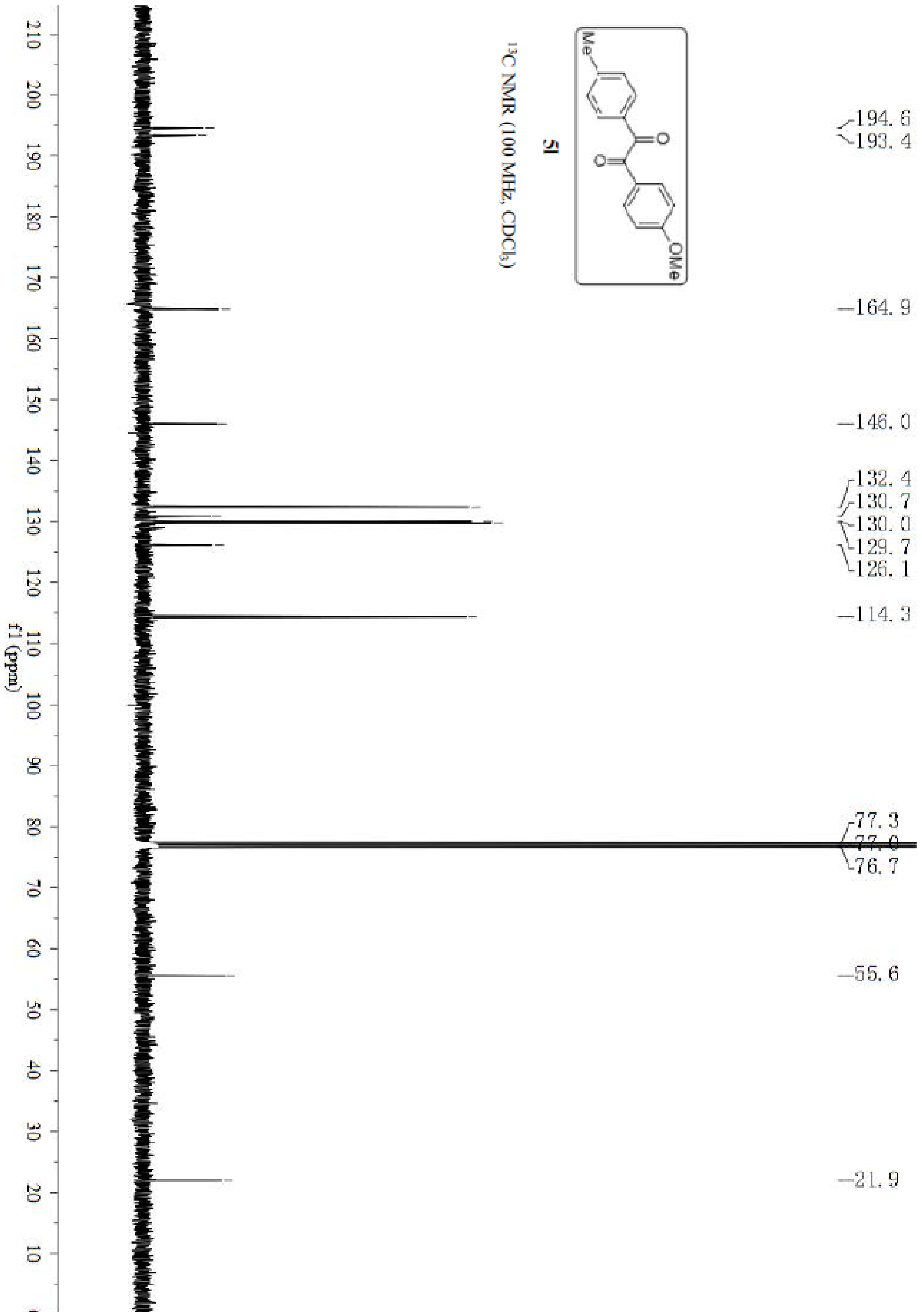

Supplementary Figure 129. ^{13}C NMR (100 MHz, $CDCl_3$) spectra of compound **5l**.

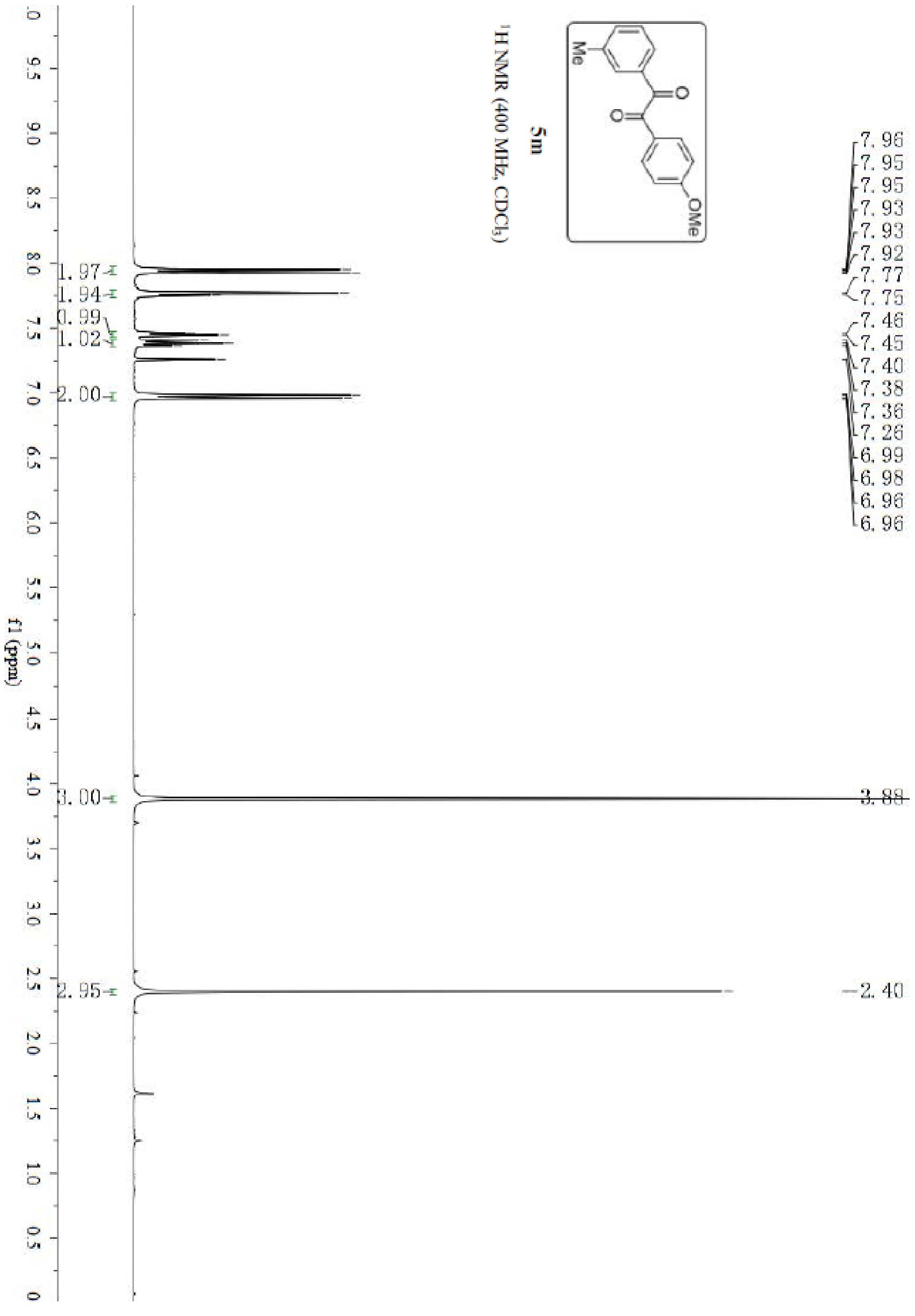

Supplementary Figure 130. ^{1}H NMR (400 MHz, $CDCl_3$) spectra of compound **5m**.

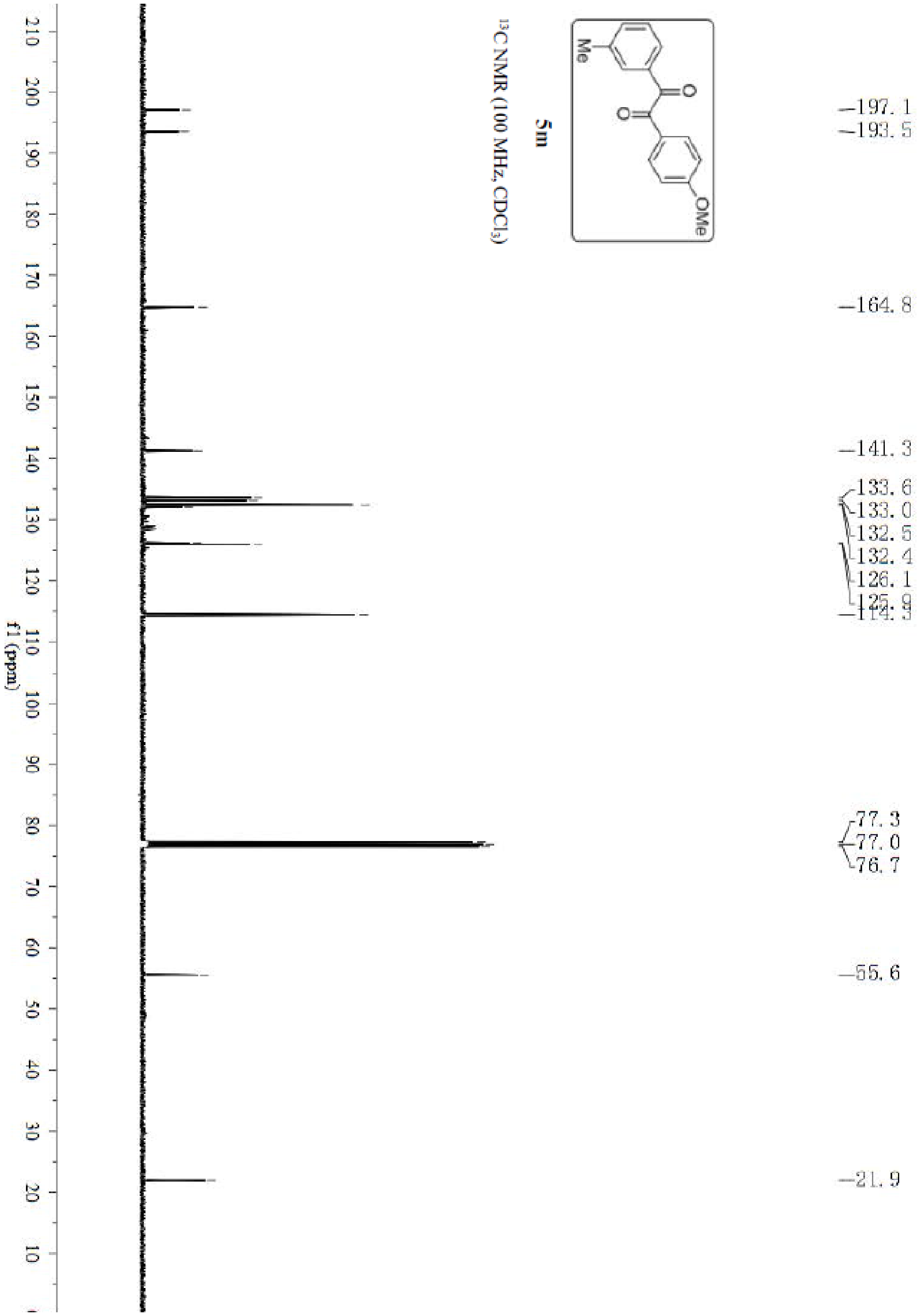

Supplementary Figure 131. ^{13}C NMR (100 MHz, CDCl$_3$) spectra of compound **5m**.

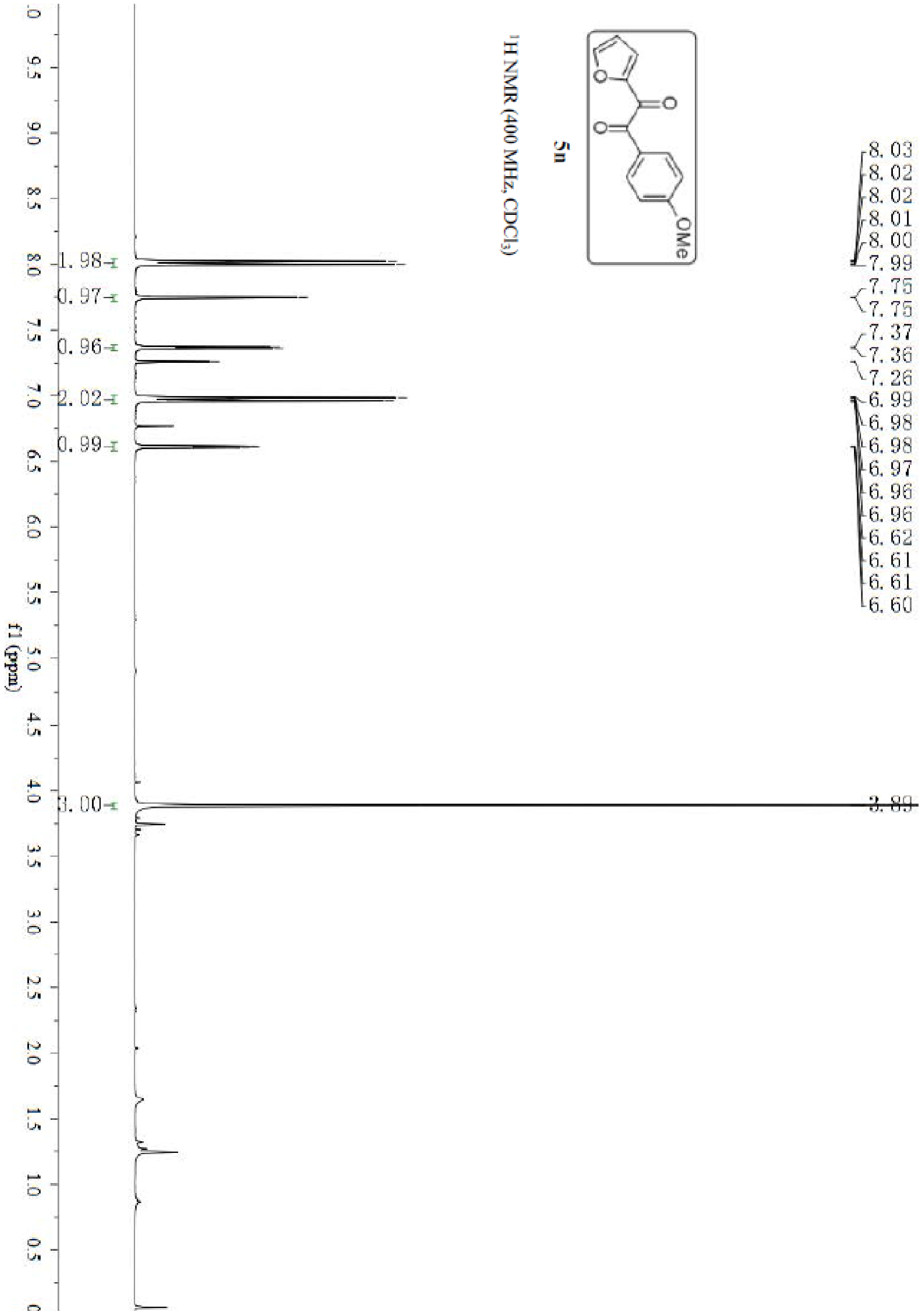

Supplementary Figure 132. ^{1}H NMR (400 MHz, $CDCl_3$) spectra of compound **5n**.

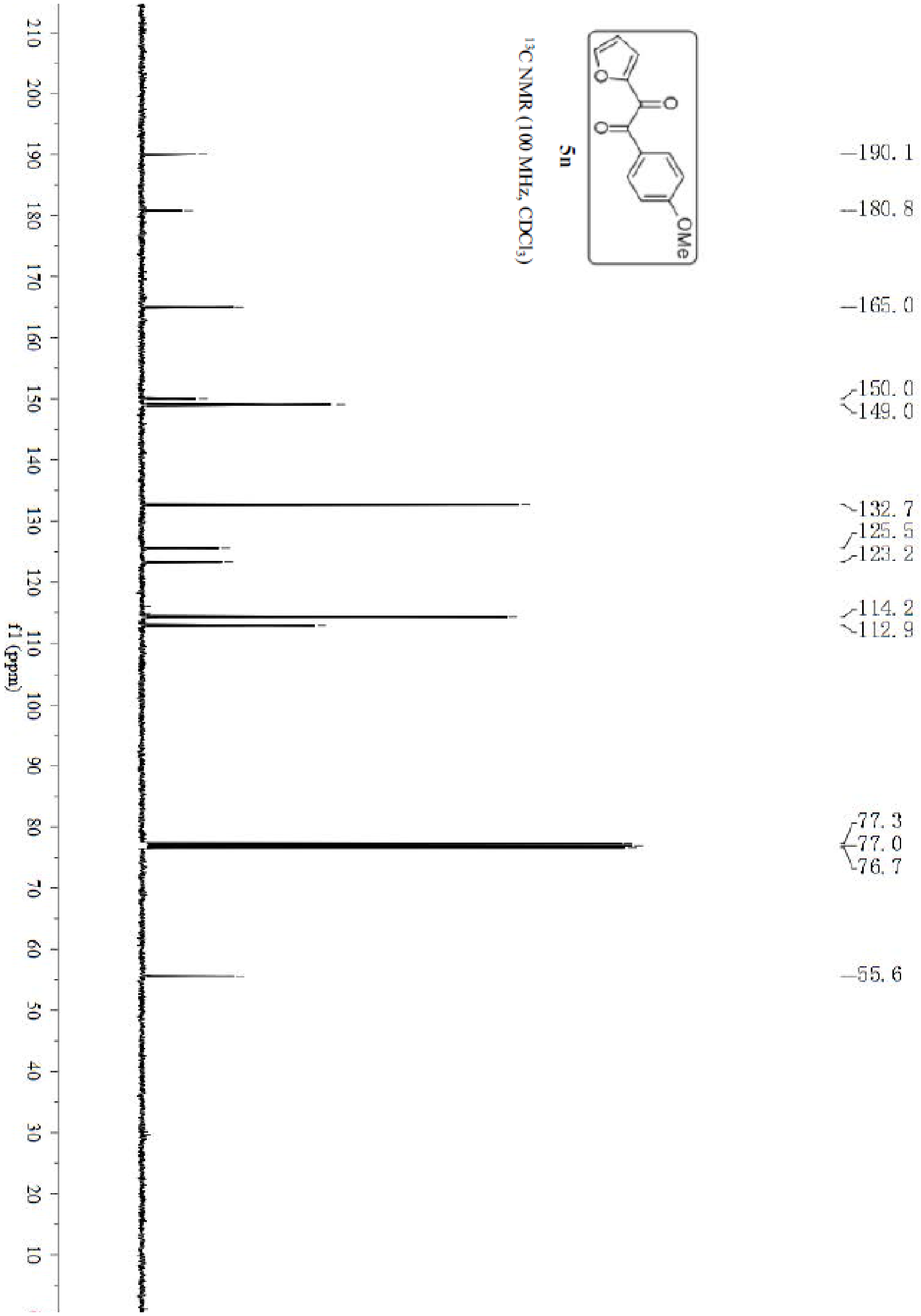

Supplementary Figure 133. ^{13}C NMR (100 MHz, $CDCl_3$) spectra of compound **5n**.

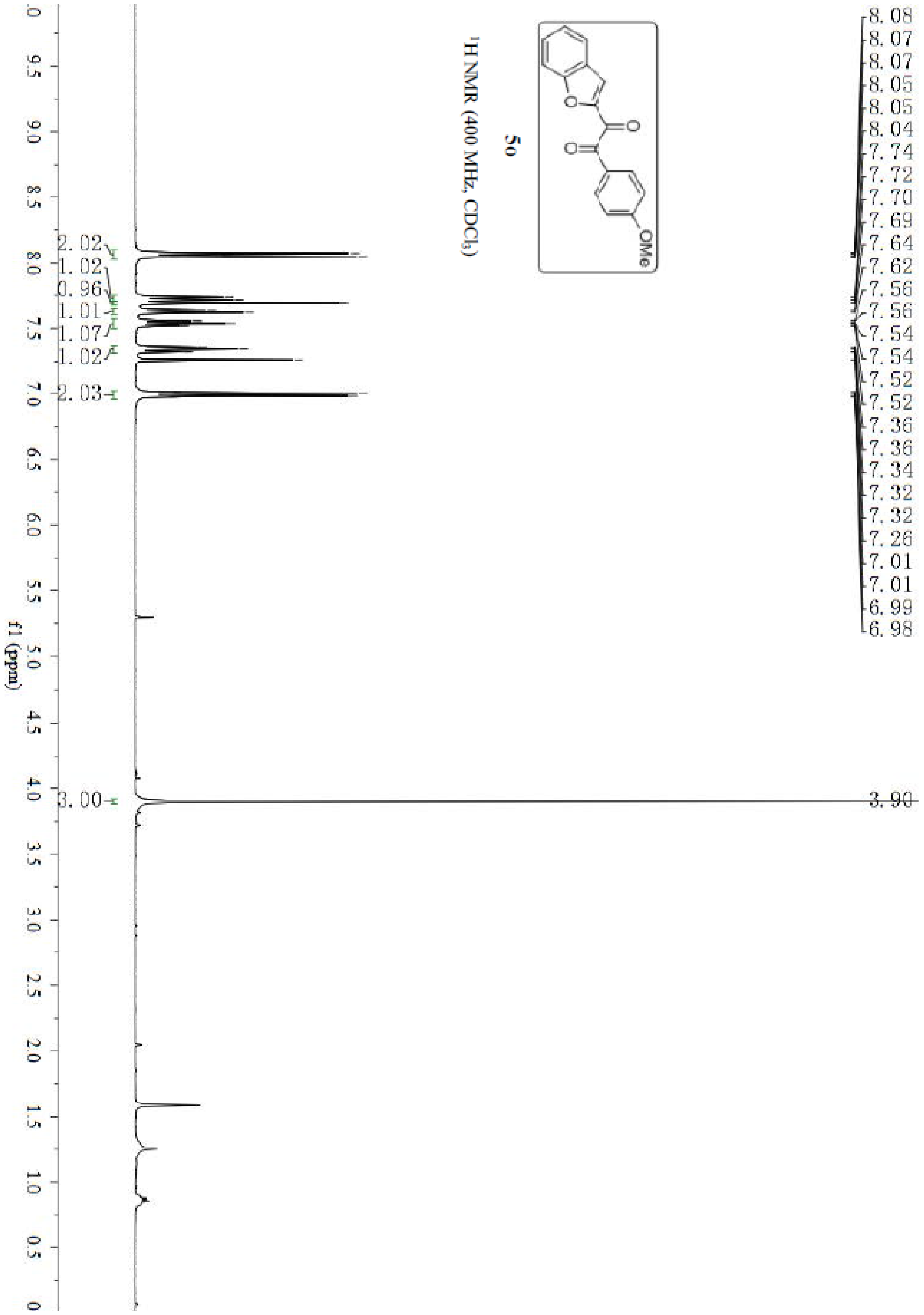

Supplementary Figure 134. ^{1}H NMR (400 MHz, $CDCl_3$) spectra of compound **5o**.

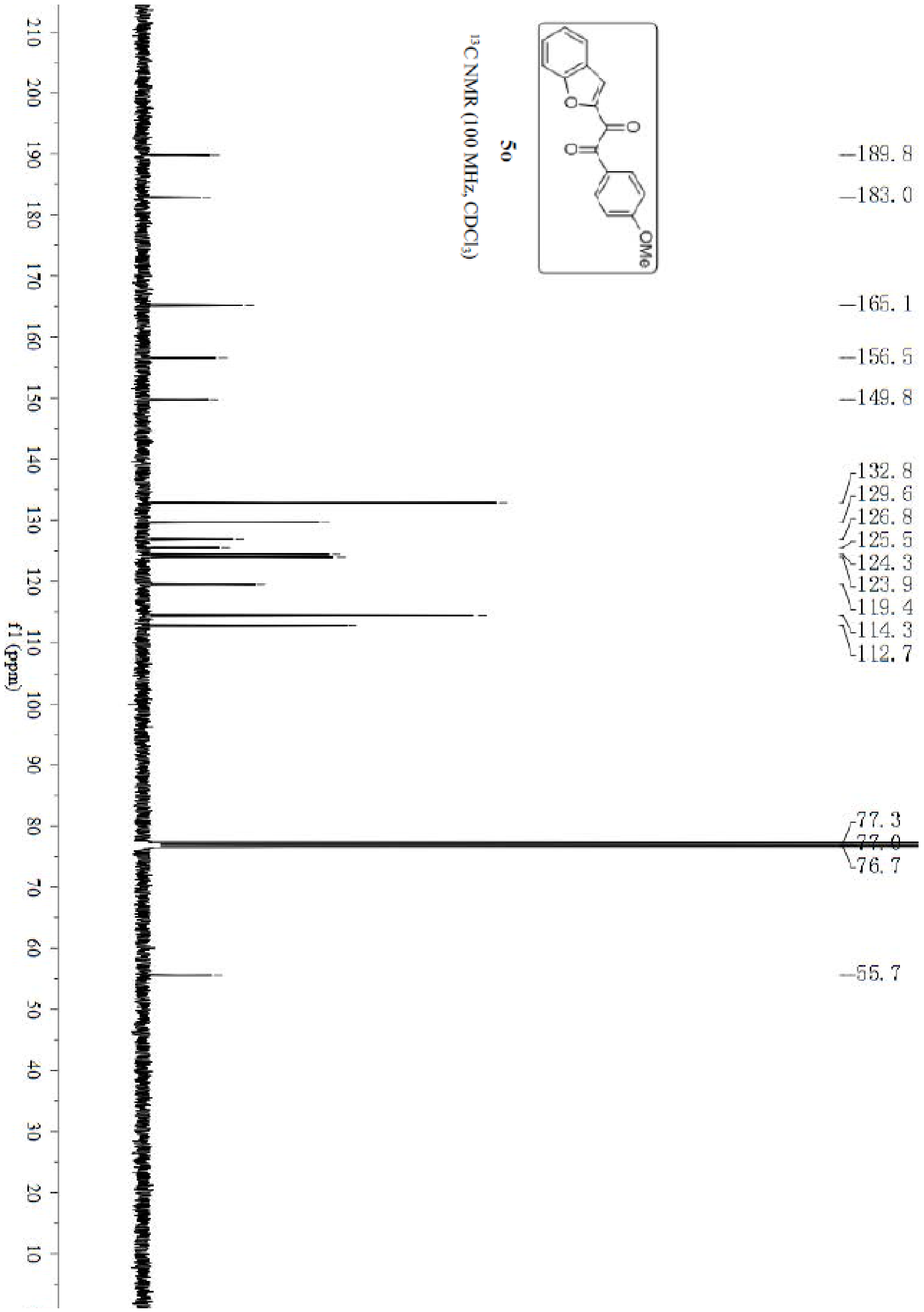

Supplementary Figure 135. ^{13}C NMR (100 MHz, $CDCl_3$) spectra of compound **5o**.

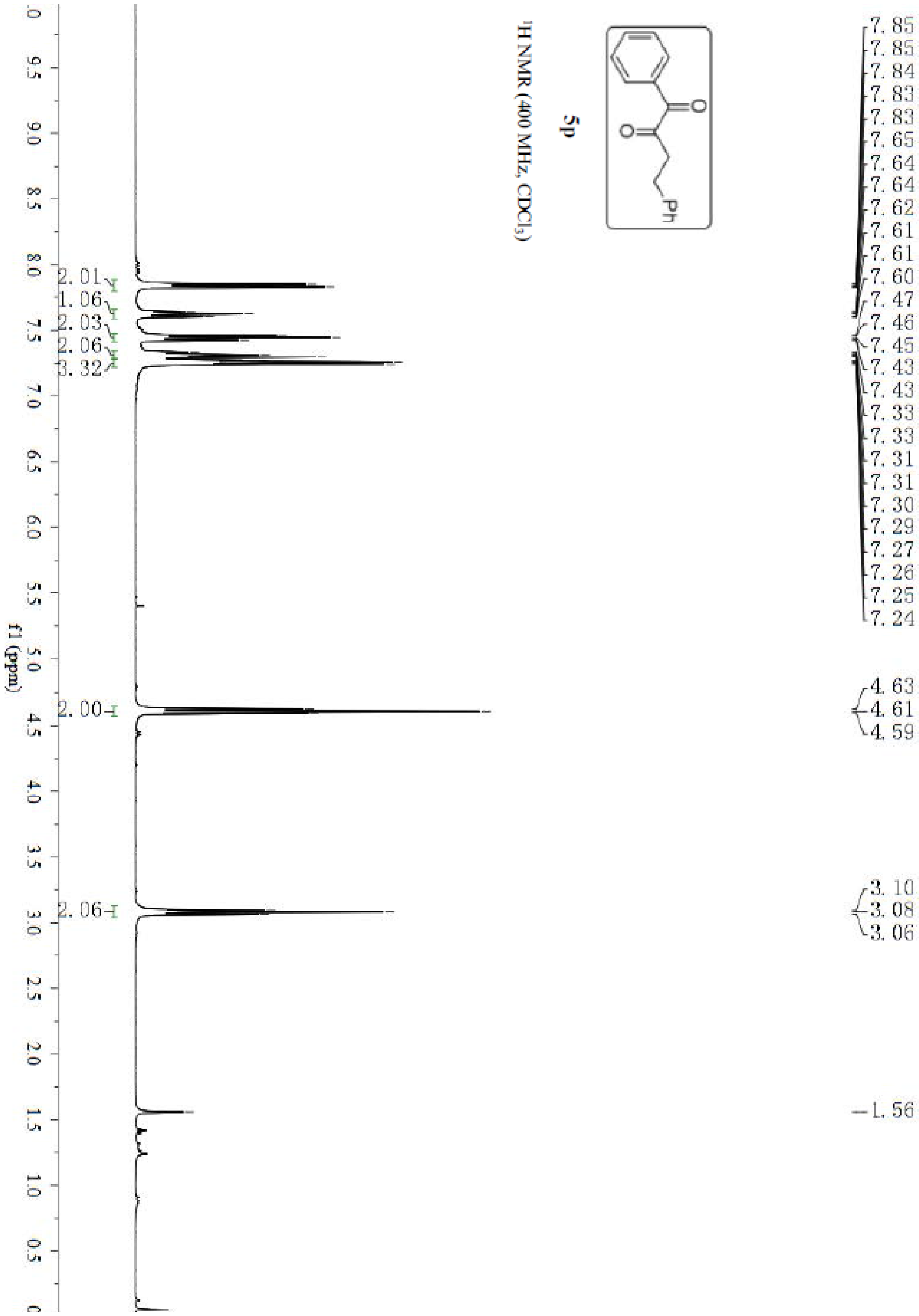

Supplementary Figure 136. ^{1}H NMR (400 MHz, $CDCl_3$) spectra of compound **5p**.

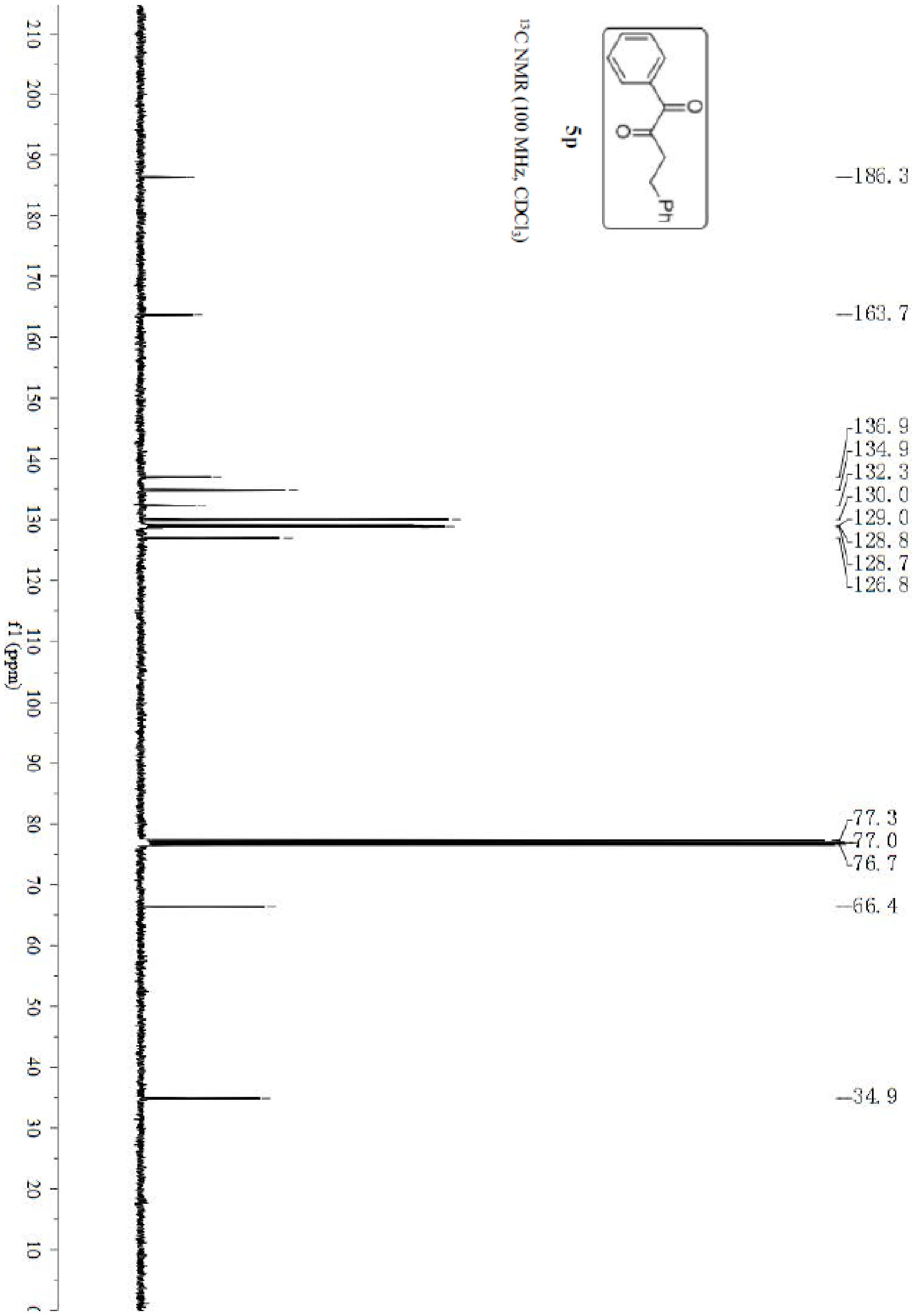

Supplementary Figure 137. ^{13}C NMR (100 MHz, $CDCl_3$) spectra of compound **5p**.

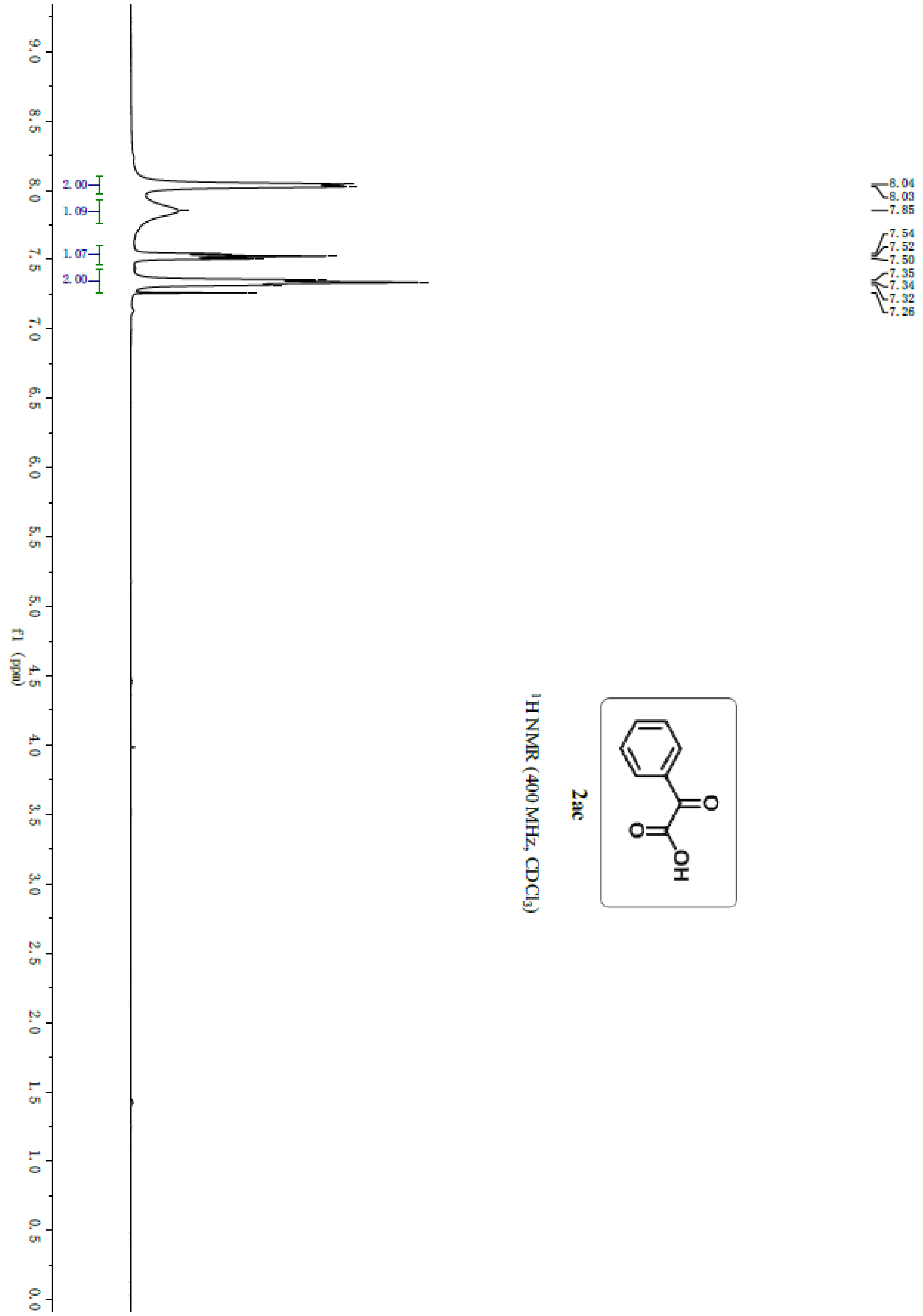

Supplementary Figure 138. ^{1}H NMR (400 MHz, $CDCl_3$) spectra of compound **2ac**.

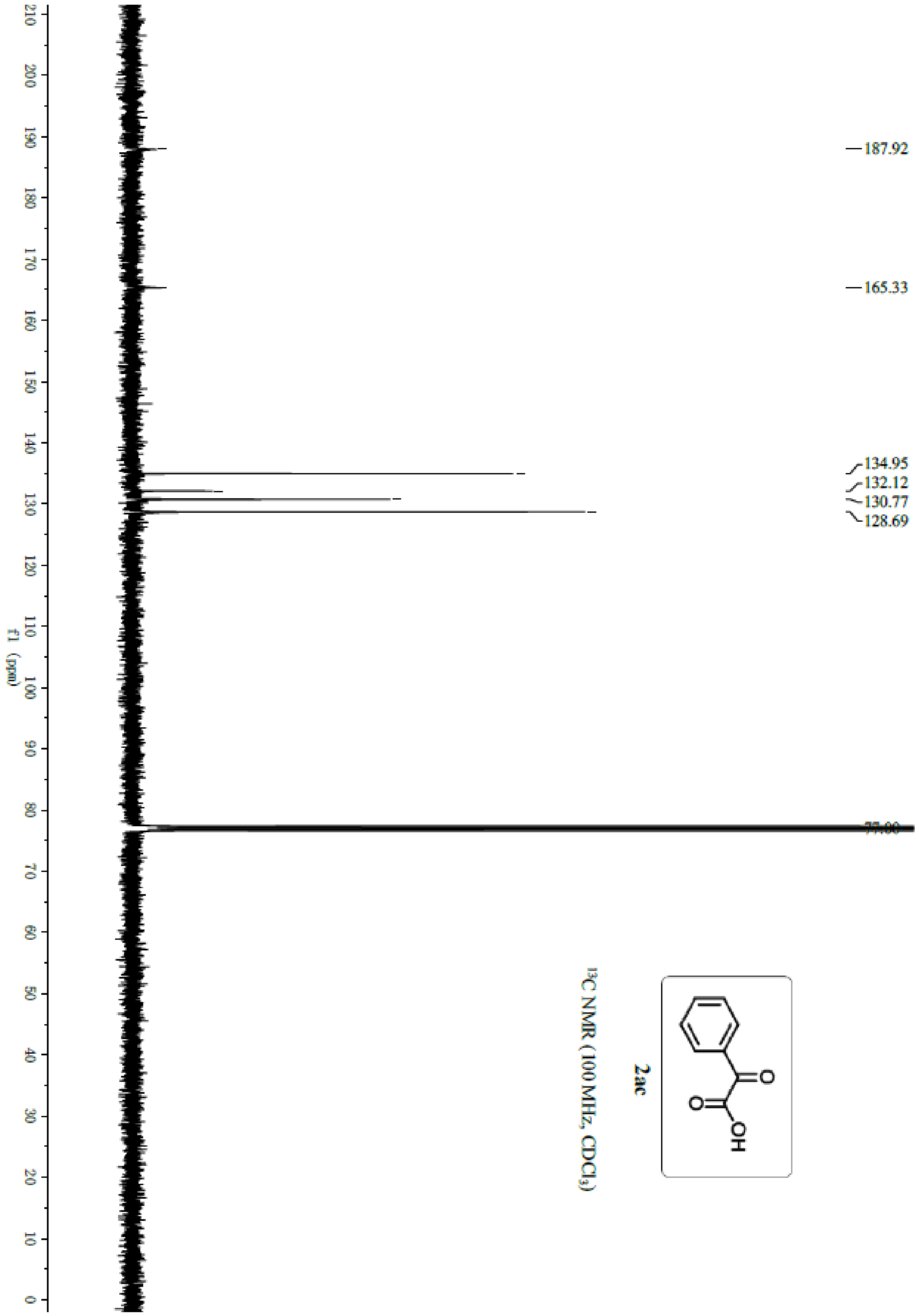

Supplementary Figure 139. ^{13}C NMR (100 MHz, $CDCl_3$) spectra of compound **2ac**.

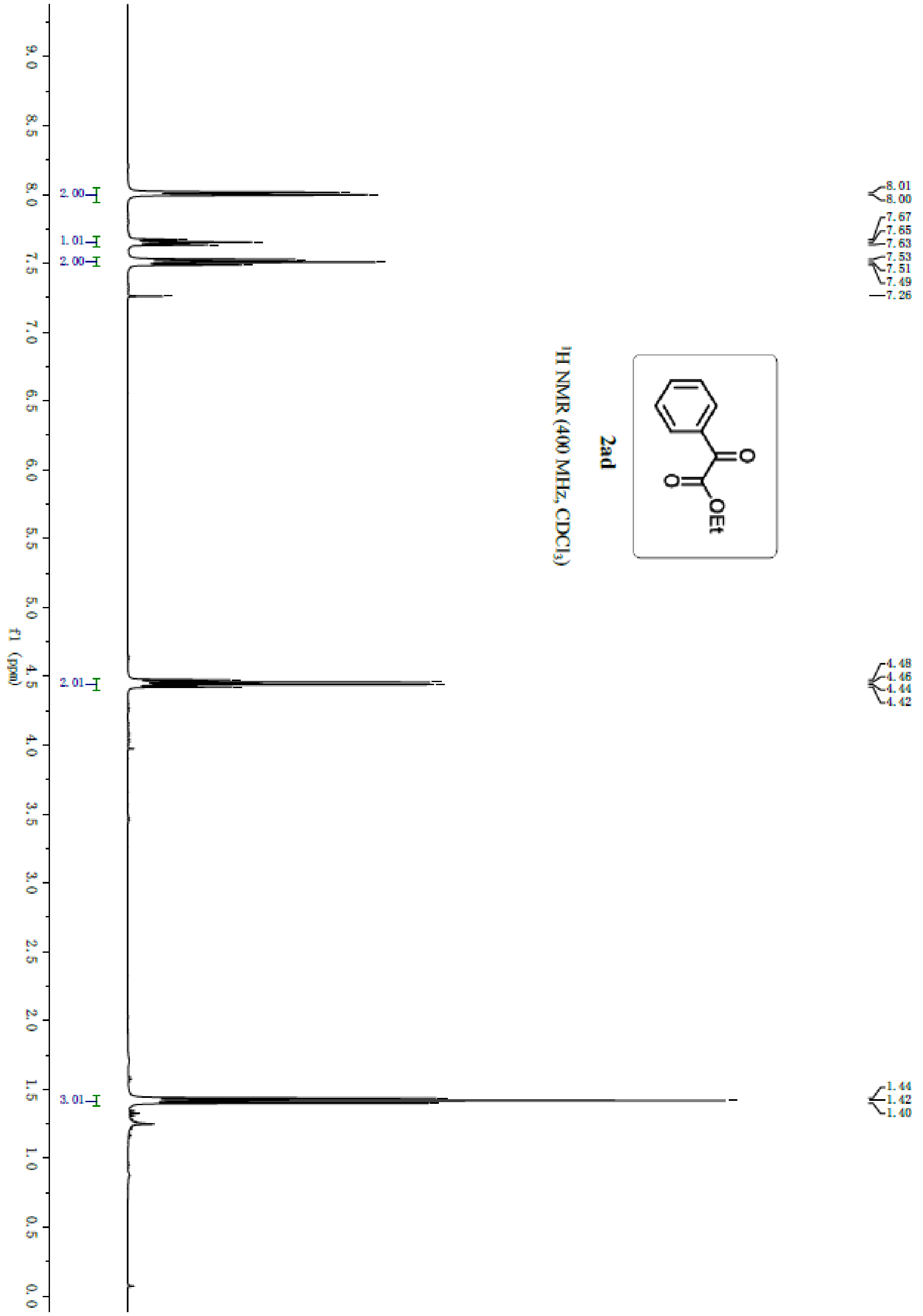

Supplementary Figure 140. ^{1}H NMR (400 MHz, $CDCl_3$) spectra of compound **2ad**.

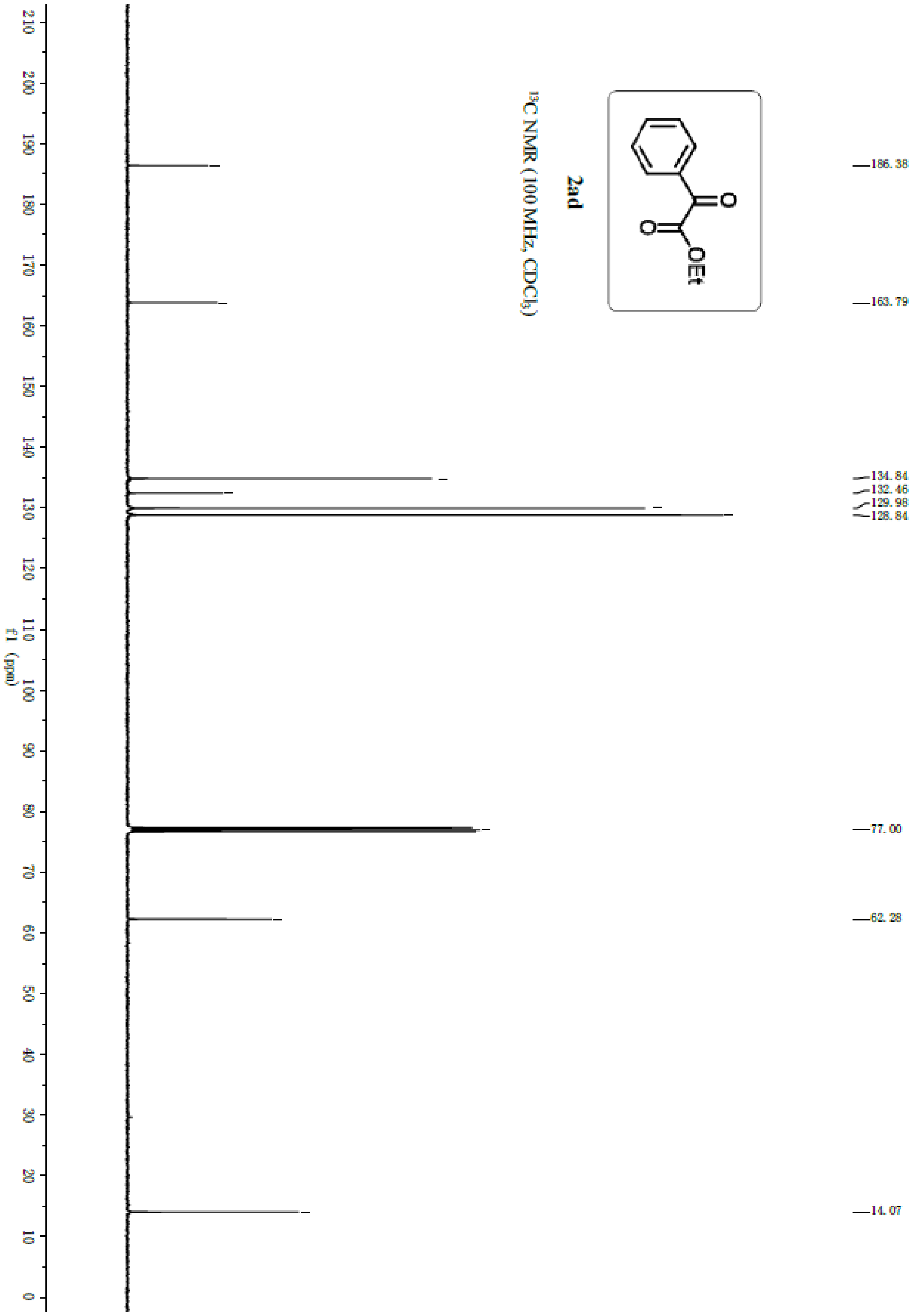

Supplementary Figure 141. ^{13}C NMR (100 MHz, $CDCl_3$) spectra of compound **2ad**.

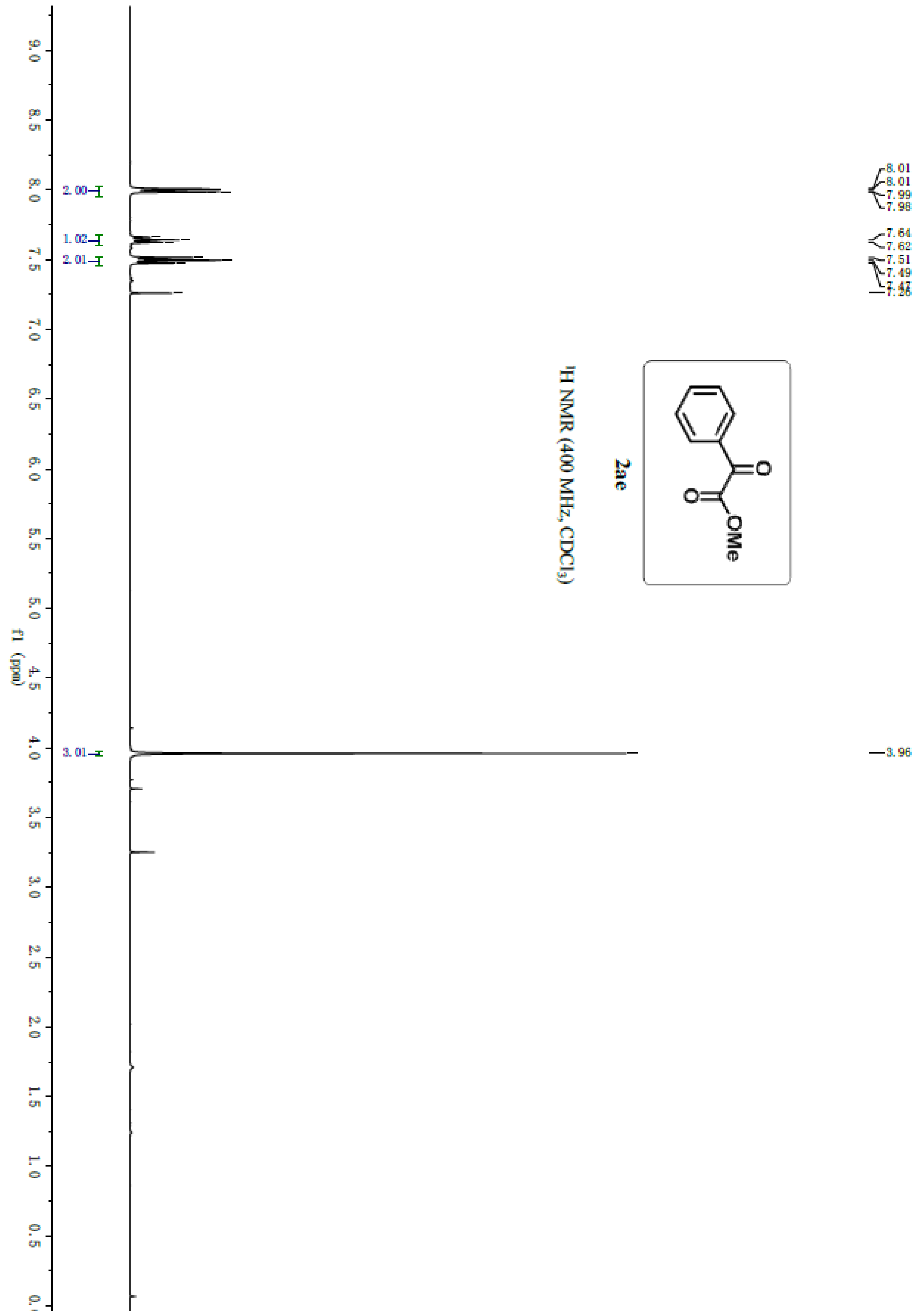

Supplementary Figure 142. ^{1}H NMR (400 MHz, $CDCl_3$) spectra of compound **2ae**.

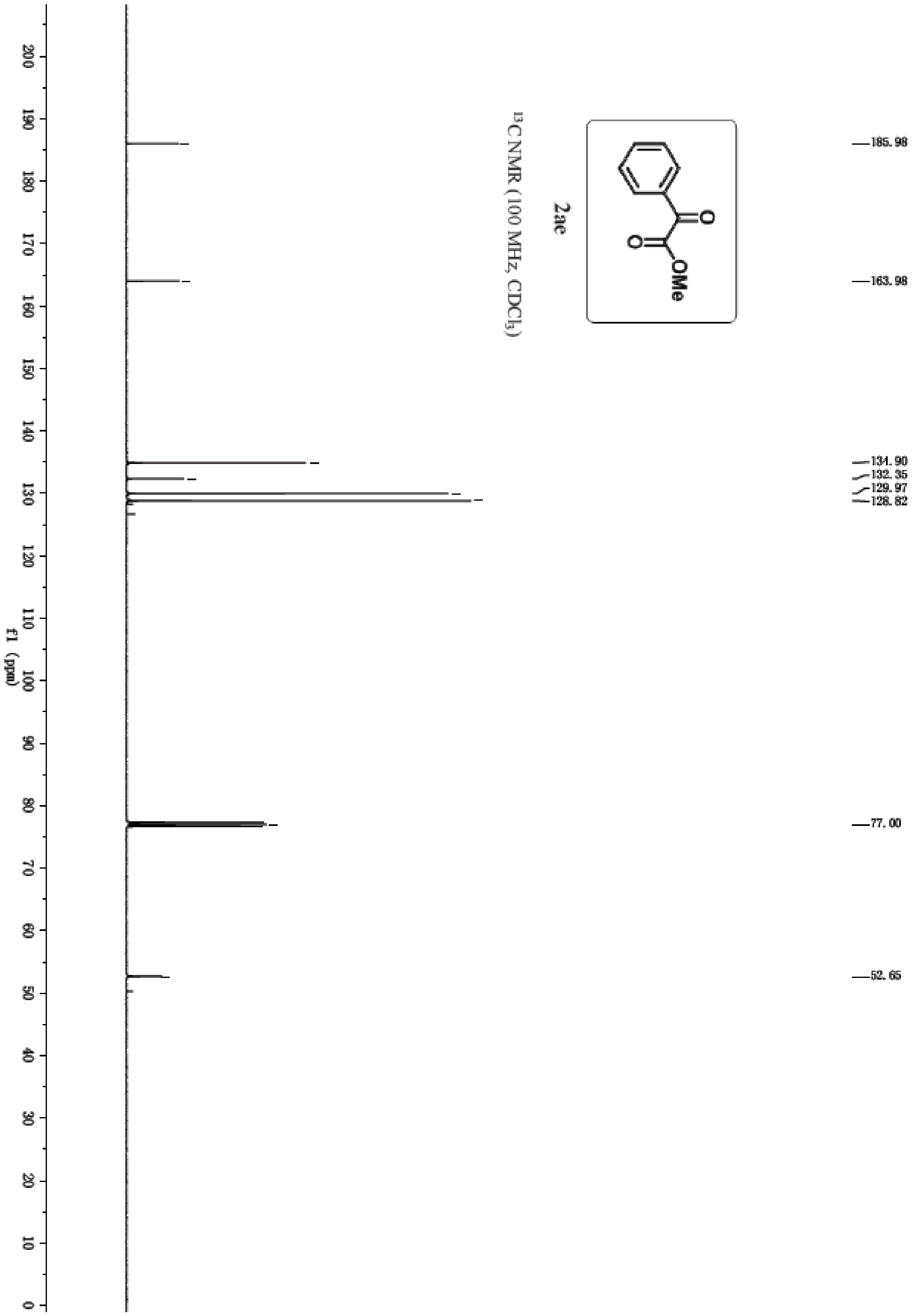

Supplementary Figure 143. ^{13}C NMR (100 MHz, $CDCl_3$) spectra of compound **2ae**.

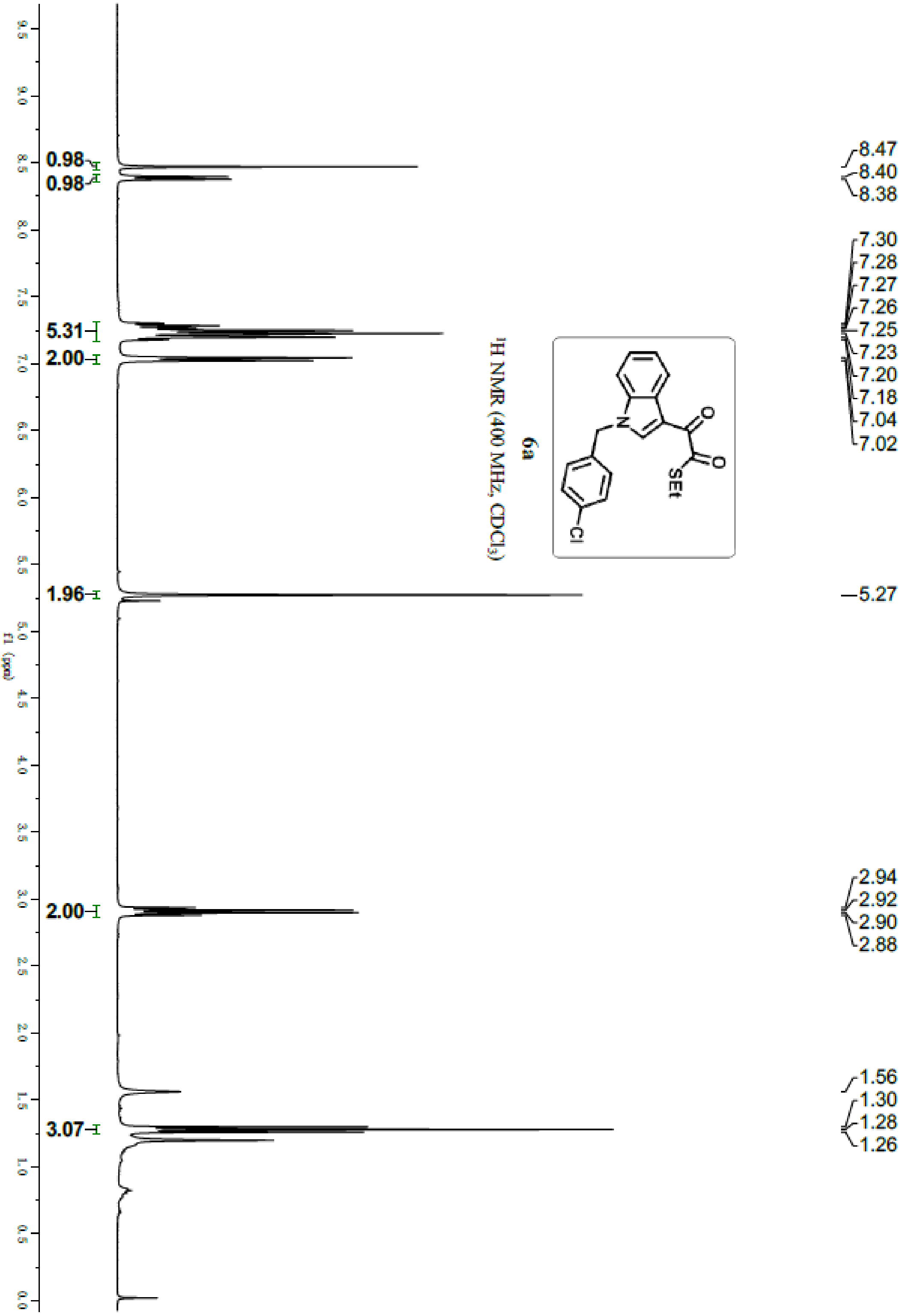

Supplementary Figure 144. ^{1}H NMR (400 MHz, $CDCl_3$) spectra of compound **6a**.

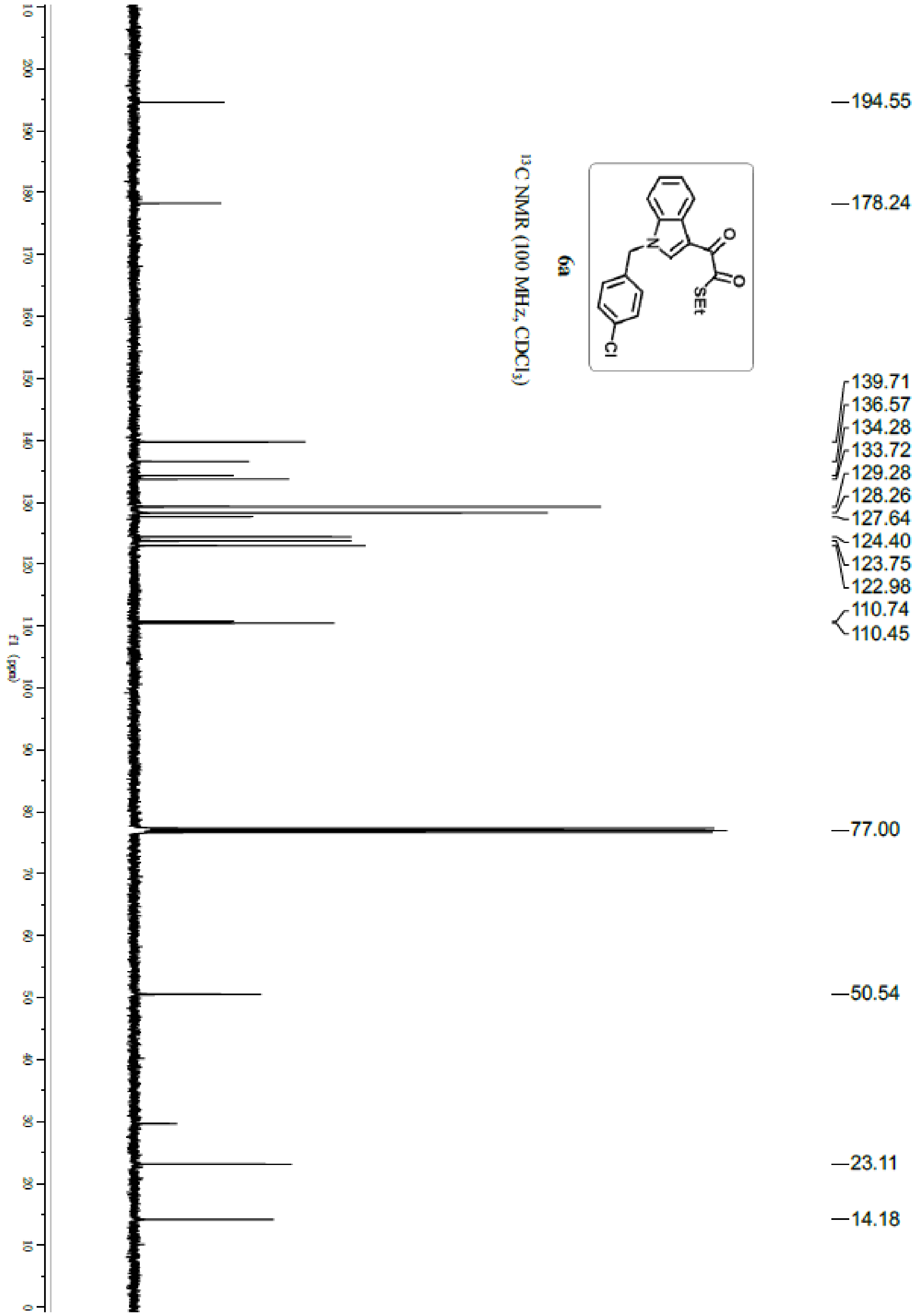

Supplementary Figure 145. ^{13}C NMR (100 MHz, $CDCl_3$) spectra of compound **6a**.

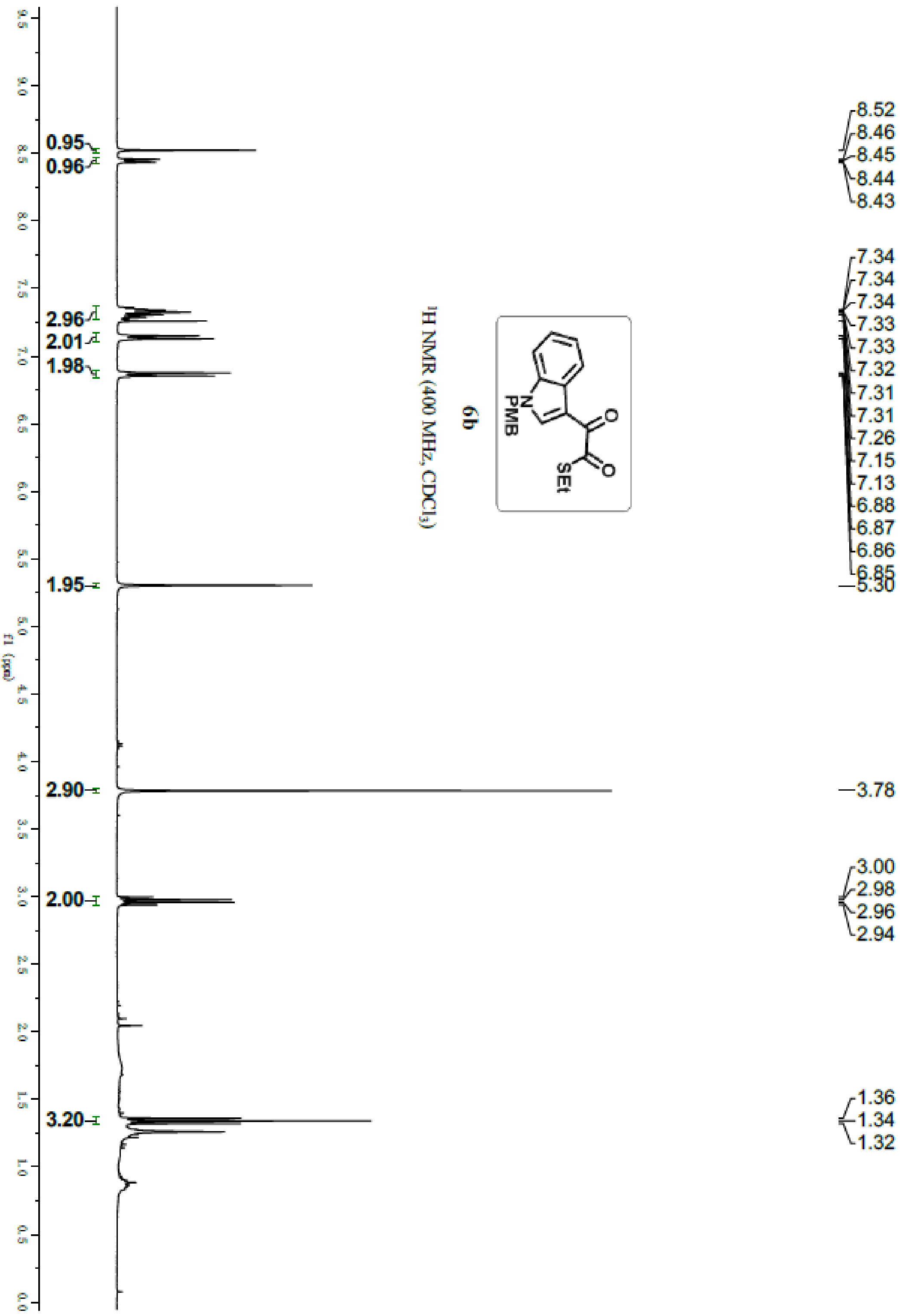

Supplementary Figure 146. ^{1}H NMR (400 MHz, $CDCl_3$) spectra of compound **6b**.

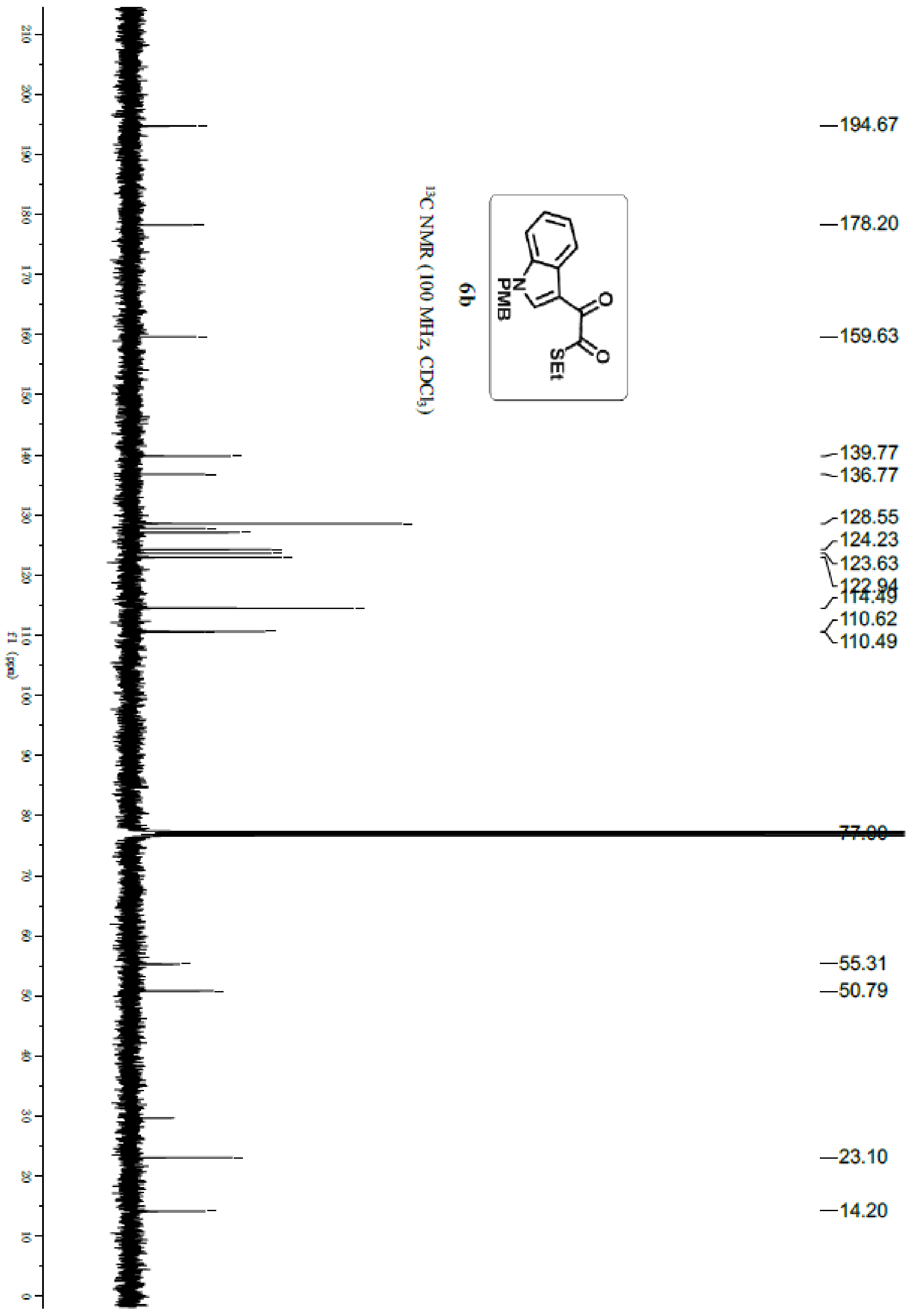

Supplementary Figure 147. ^{13}C NMR (100 MHz, $CDCl_3$) spectra of compound **6b**.

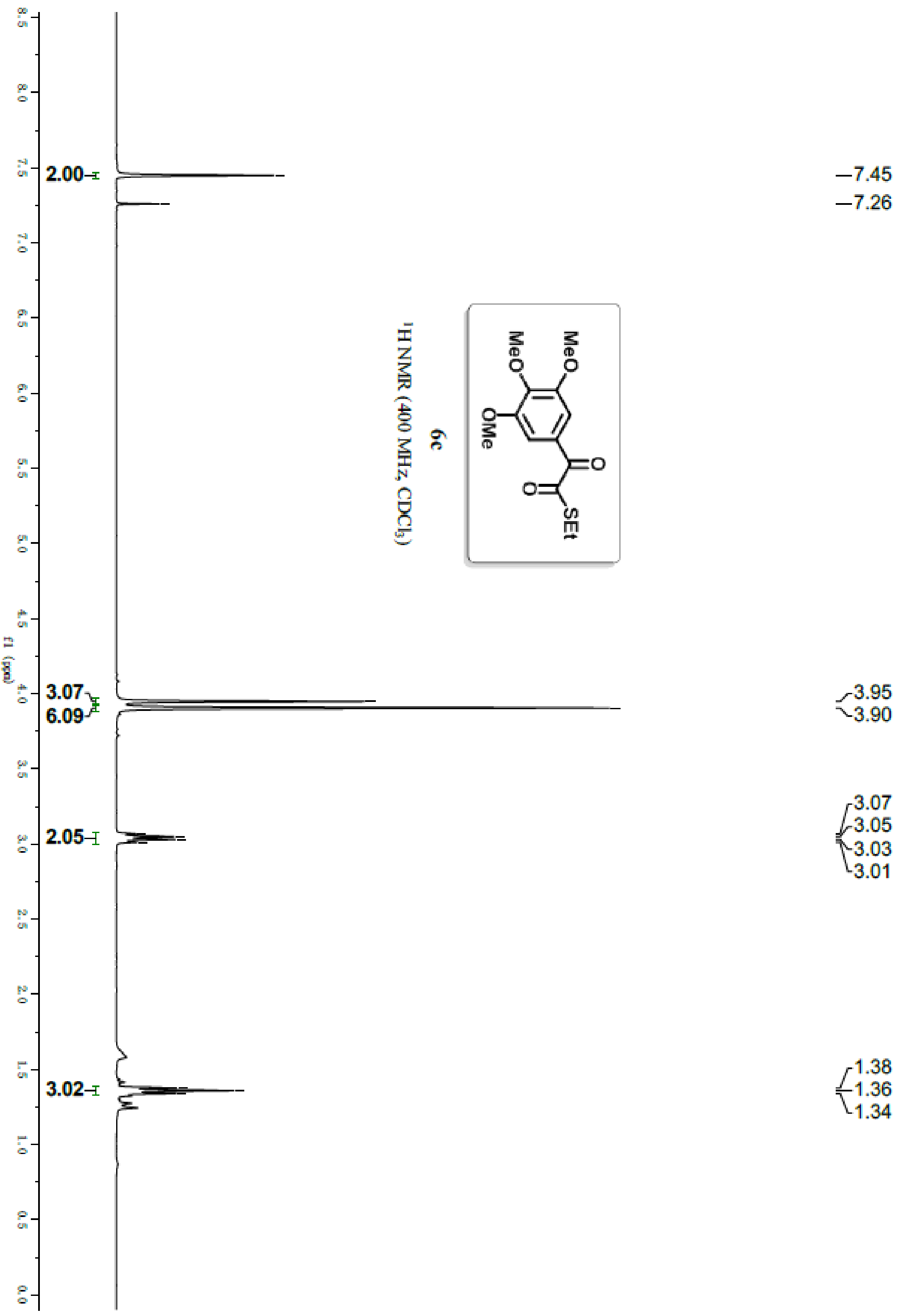

Supplementary Figure 148. ^{1}H NMR (400 MHz, $CDCl_3$) spectra of compound **6c**.

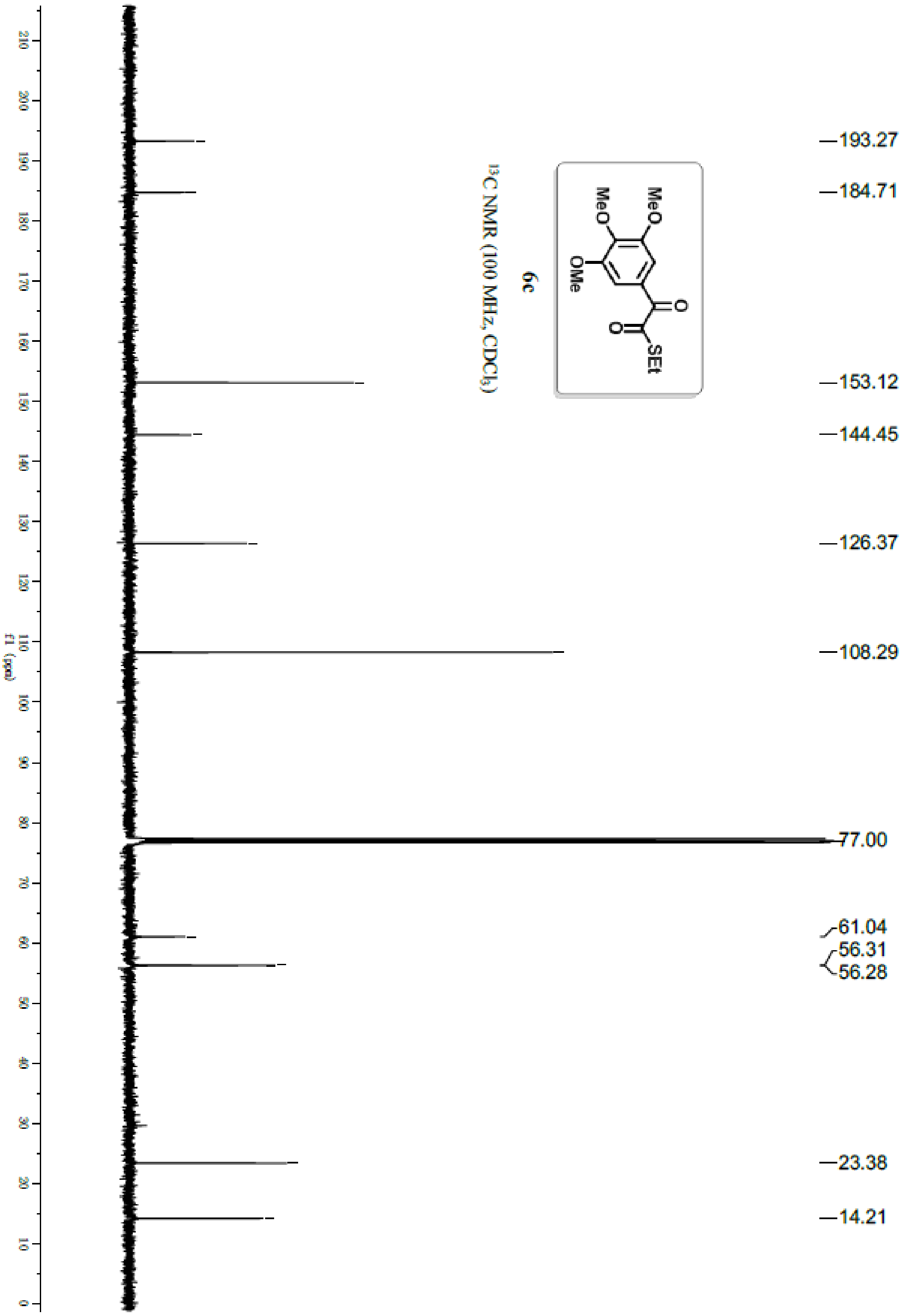

Supplementary Figure 149. ^{13}C NMR (100 MHz, $CDCl_3$) spectra of compound **6c**.

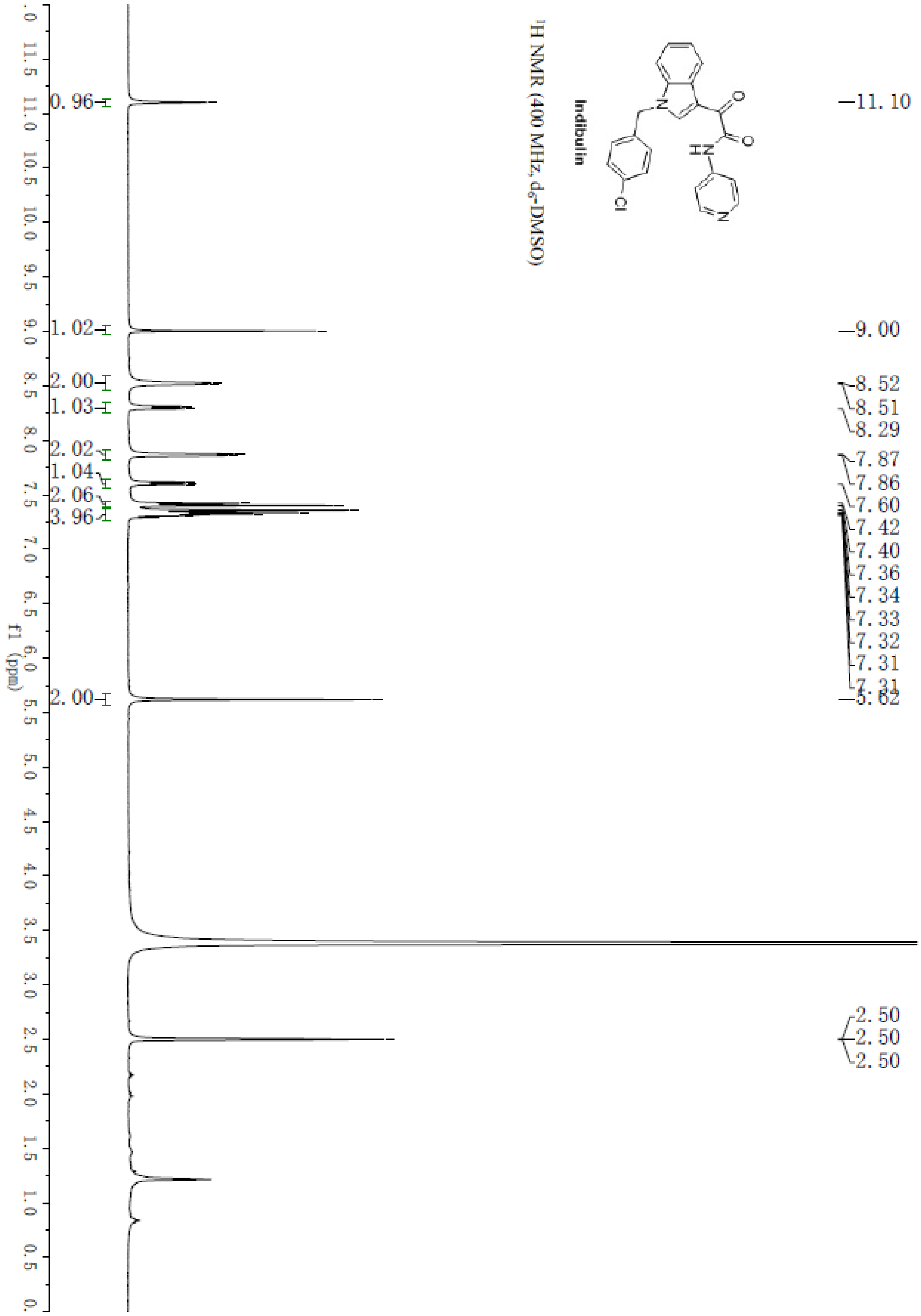

Supplementary Figure 150. ^{1}H NMR (400 MHz, d_6-DMSO) spectra of compound **7a**.

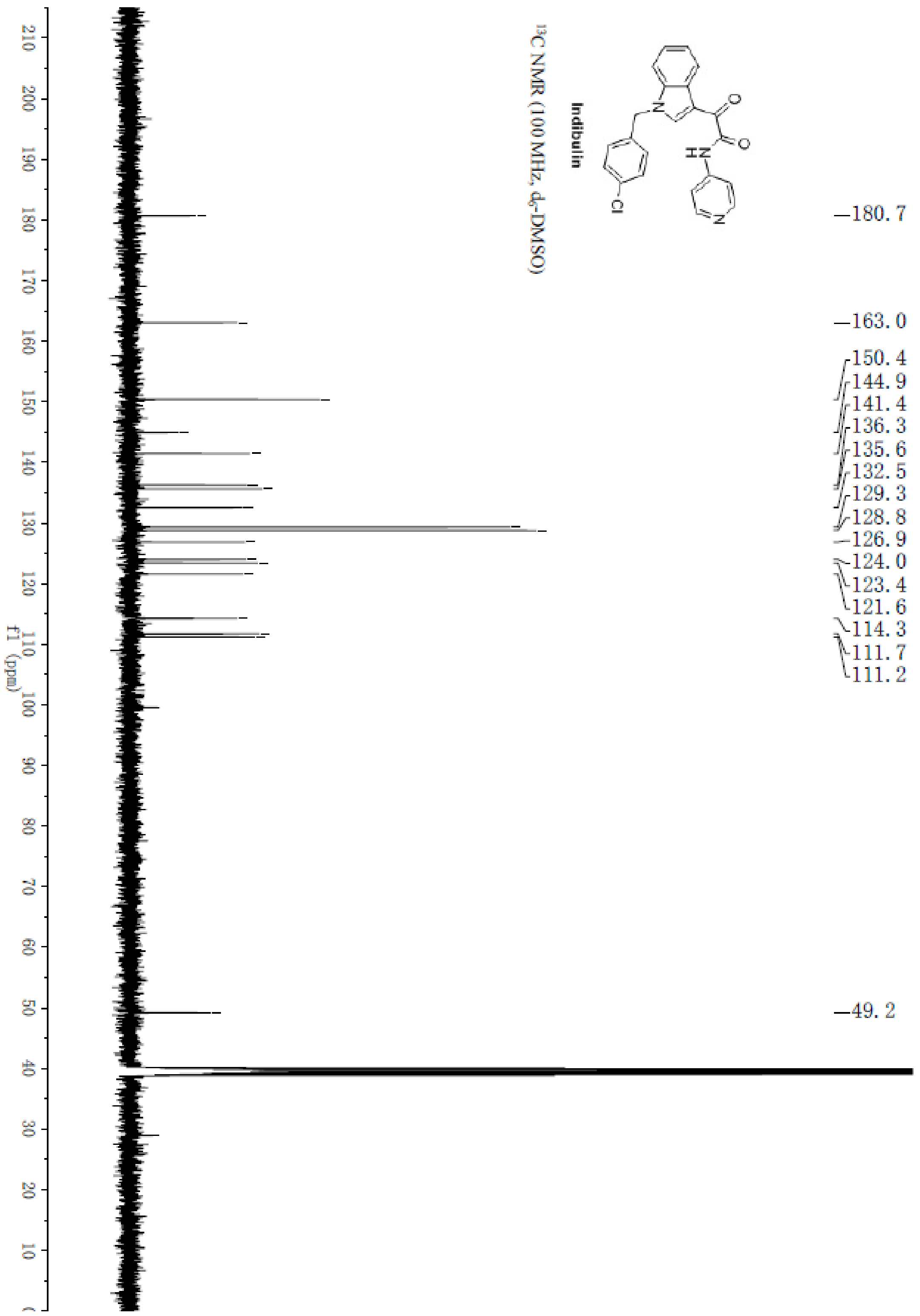

Supplementary Figure 151. ^{13}C NMR (100 MHz, d_6-DMSO) spectra of compound **7a**.

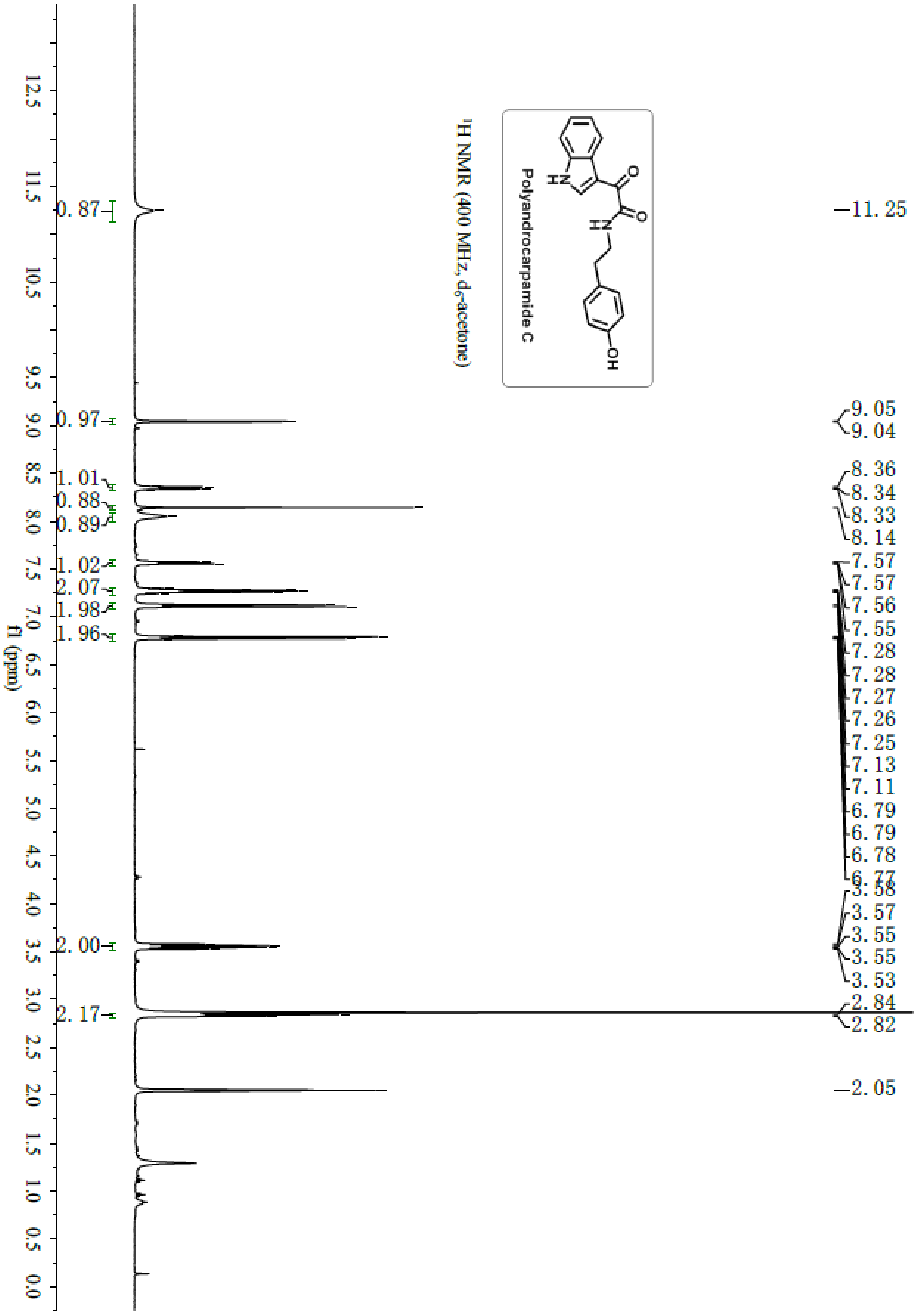

Supplementary Figure 152. ^{1}H NMR (400 MHz, d_6-acetone) spectra of compound **7b**.

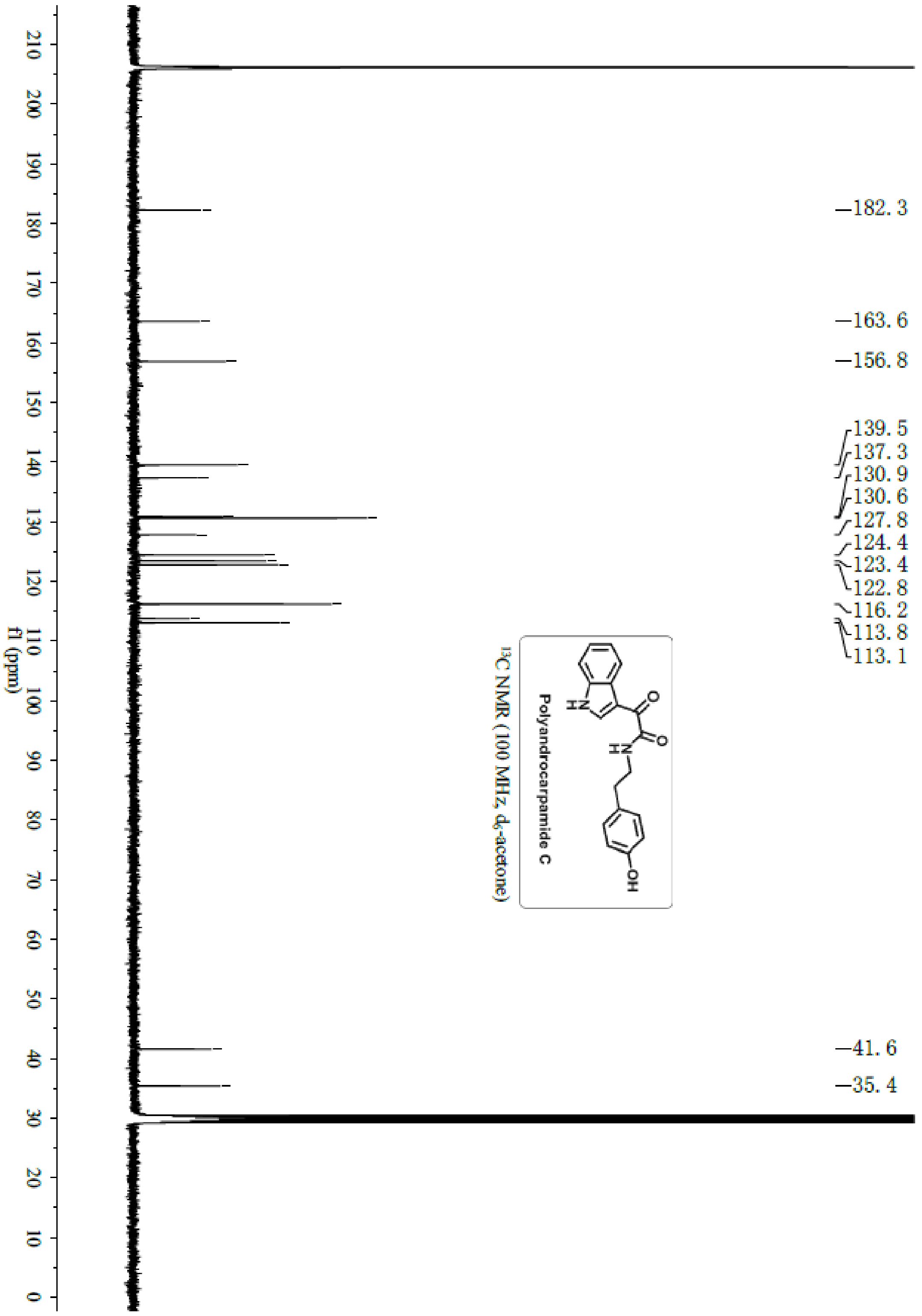

Supplementary Figure 153. ^{13}C NMR (100 MHz, d_6-acetone) spectra of compound **7b**.

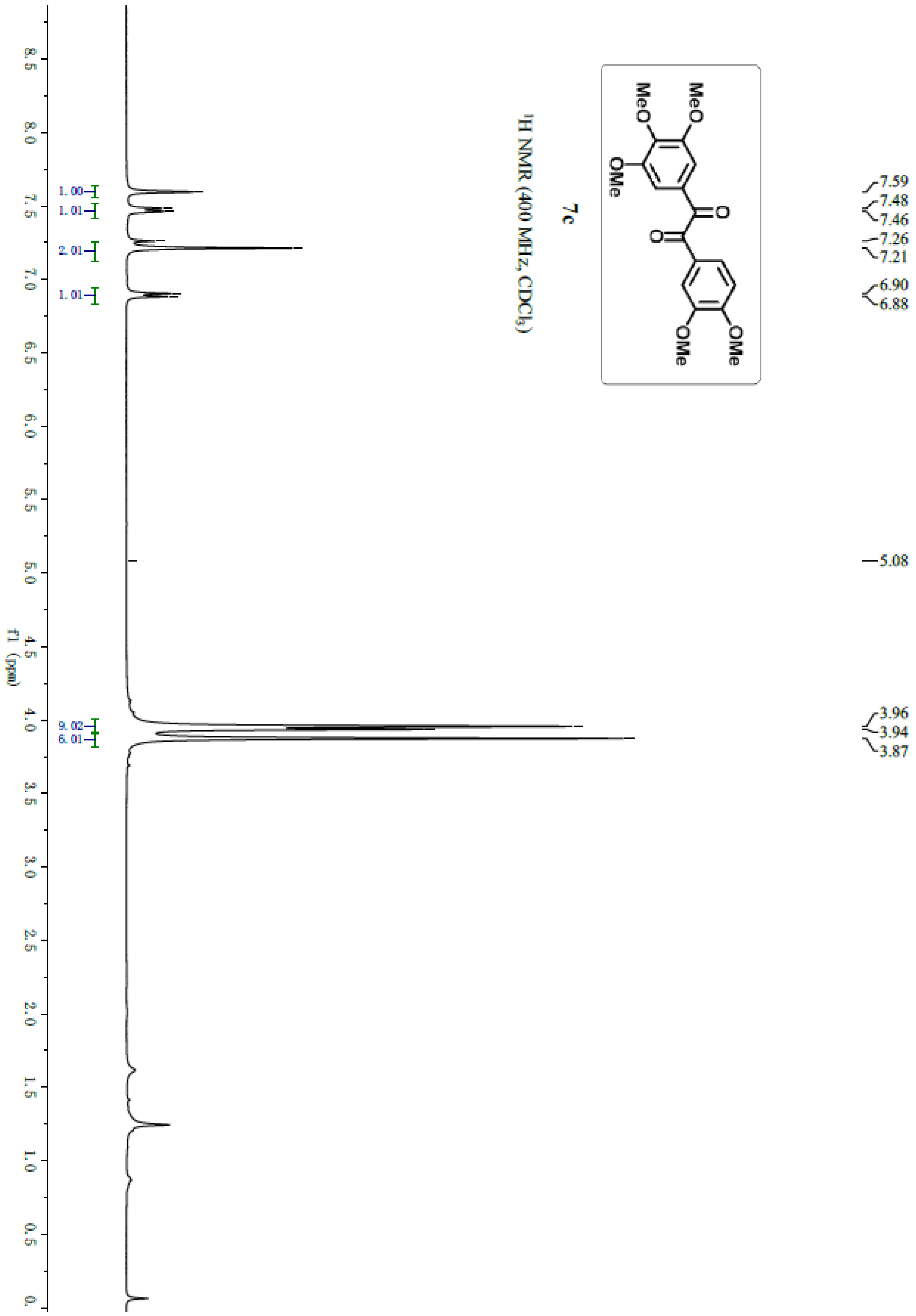

Supplementary Figure 154. ^{1}H NMR (400 MHz, $CDCl_3$) spectra of compound **7c**.

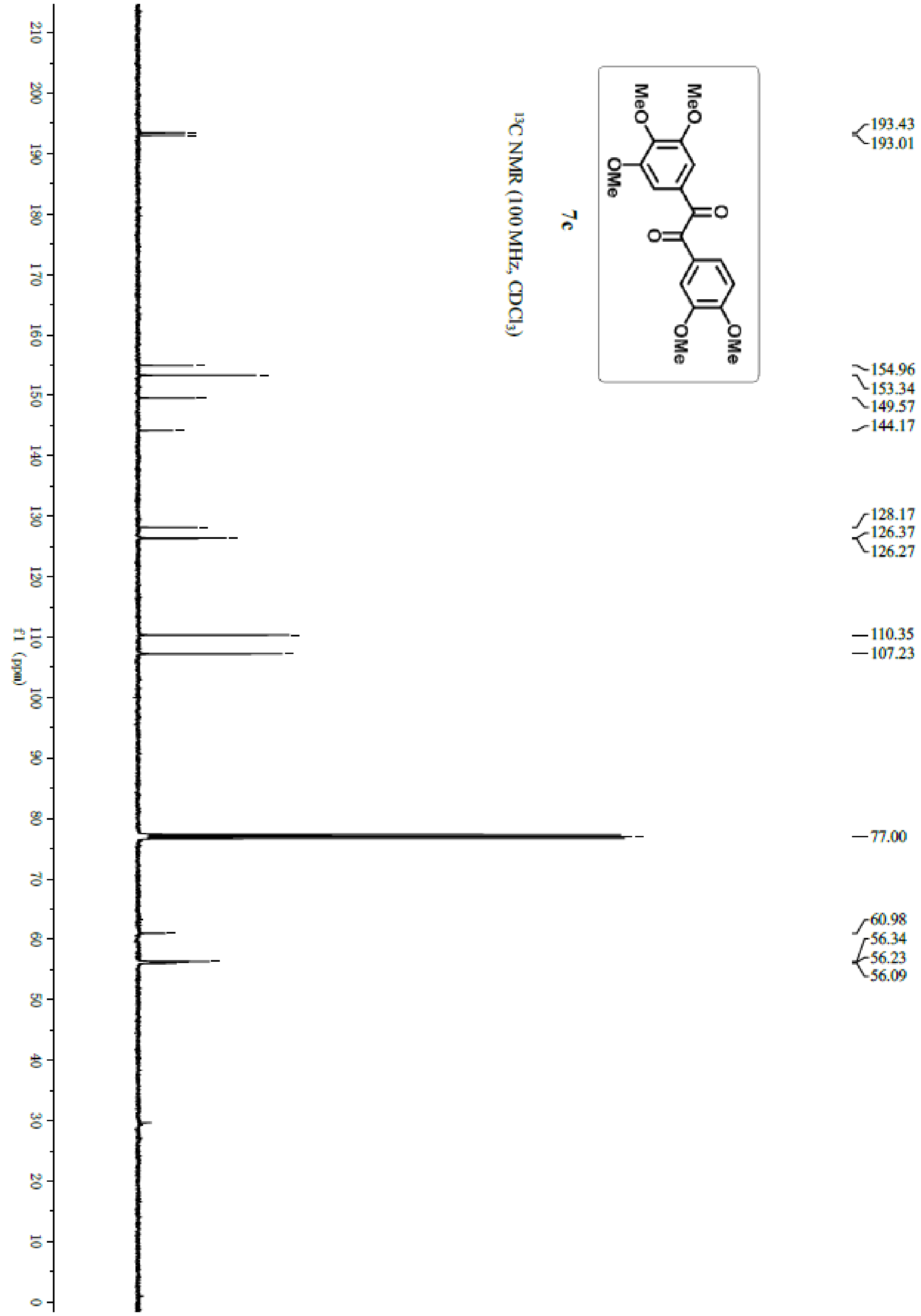

Supplementary Figure 155. ^{13}C NMR (100 MHz, $CDCl_3$) spectra of compound **7c**.

Supplementary References

1. Liu, H., Dong, C., Zhang, Z., Wu, P. & Jiang, X. Transition-metal-free aerobic oxidative cleavage of C-C bonds in α-hydroxy ketones and mechanistic insight to the reaction pathway. *Angew. Chem. Int. Ed.* **51**, 12570-12574 (2012).

2. Moriarty, R. M. Organohypervalent iodine: Development, applications, and future directions. *J. Org. Chem.* **70**, 2893-2903 (2005).
